# Aruncin B: Synthetic Studies, Structural Reassignment and Biological Evaluation

**DOI:** 10.1002/chem.201702949

**Published:** 2017-10-30

**Authors:** Aubert Ribaucourt, Christopher Towers, Laia Josa‐Culleré, Frances Willenbrock, Amber L. Thompson, David M. Hodgson

**Affiliations:** ^1^ Department of Chemistry University of Oxford, Chemistry Research Laboratory Mansfield Road Oxford OX1 3TA UK; ^2^ Department of Oncology University of Oxford, Old Road Campus Research Building Oxford OX3 7DQ UK

**Keywords:** anticancer, butenolide, cytotoxin, natural products, structure elucidation

## Abstract

A ring‐closing alkene metathesis (RCM)/ oxyselenation‐selenoxide elimination sequence was established to the sodium salts *E*‐ and *Z*‐**25** of the originally proposed structure for the recently isolated cytotoxin aruncin B (**1**), as well as to the sodium salt *Z*‐**34** of a related ethyl ether regioisomer; however, none of their corresponding free acids could be obtained. Their acid sensitivity, together with detailed analysis of the spectroscopic data indicated that profound structural revision was necessary. This led to reassignment of aruncin B as a *Z*‐γ‐alkylidenebutenolide *Z*‐**36**. Although a related RCM/ oxyselenation‐selenoxide elimination sequence was used to confirm the γ‐alkylidenebutenolide motif, a β‐iodo Morita‐Baylis–Hillman reaction/ Sonogashira cross‐coupling‐5‐*exo‐dig* lactonisation sequence was subsequently developed, due to its brevity and flexibility for diversification. Aruncin B (**36**), together with 14 γ‐alkylidenebutenolide analogues, were generated for biological evaluation.

## Introduction

**Figure 1:**
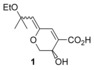
Originally reported structure of aruncin B (**1**).^2^

Inhibitors of antiapoptotic proteins from the Bcl‐2 family are currently considered to be promising leads for drug discovery programs, especially for the treatment of drug‐resistant tumours.^1^ In 2011, Woo and co‐workers reported the isolation of several previously unknown cytotoxins from the aerial parts extract of *Aruncus dioicus* var. *kamtschaticus*, a plant found on the Korean island Ulleungdo.^2^ Among these, aruncin B (**1**, Figure 1) stood out: early biological studies showed inhibitive properties towards Bcl‐2 family proteins, with some selectivity for malignant over human cells.^3^ The structure of aruncin B (**1**) was originally assigned using spectroscopic methods, as a monoterpenoid containing a dihydropyranol core.^2^ It features an unusual cross‐conjugated exocyclic enol ether and, as originally reported, a potentially acid‐labile tertiary allylic ethyl ether and carboxylic acid functionality within the same structure. The olefin geometry and absolute configuration remained undetermined. Its unusual structural features and promising biological activity made aruncin B (**1**) an interesting target for synthetic studies. In this Full Paper, we detail our synthetic studies towards the originally reported structure of aruncin B (**1**), as well as two closely related isomeric structures, its structural revision and synthetic routes leading to the correct structure.^4^ The conciseness and convergent nature of the latter enabled access to a library of analogues for biological evaluation.

**Scheme 1:**
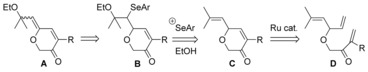
Original retrosynthesis.

Retrosynthetically, aruncin B (**1**) could be derived from a dihydropyranone core (**A**, Scheme 1), with installation of the key enol ether being achieved through an ethoxyselenation‐selenoxide elimination sequence (**C**→**B**→**A**).^5^ Diene **C** would be obtained by ring‐closing alkene metathesis (RCM) of an appropriate precursor triene (**D**).^6^

## Results and Discussion

### Synthesis of a model dihydropyranone 8

**Scheme 2:**
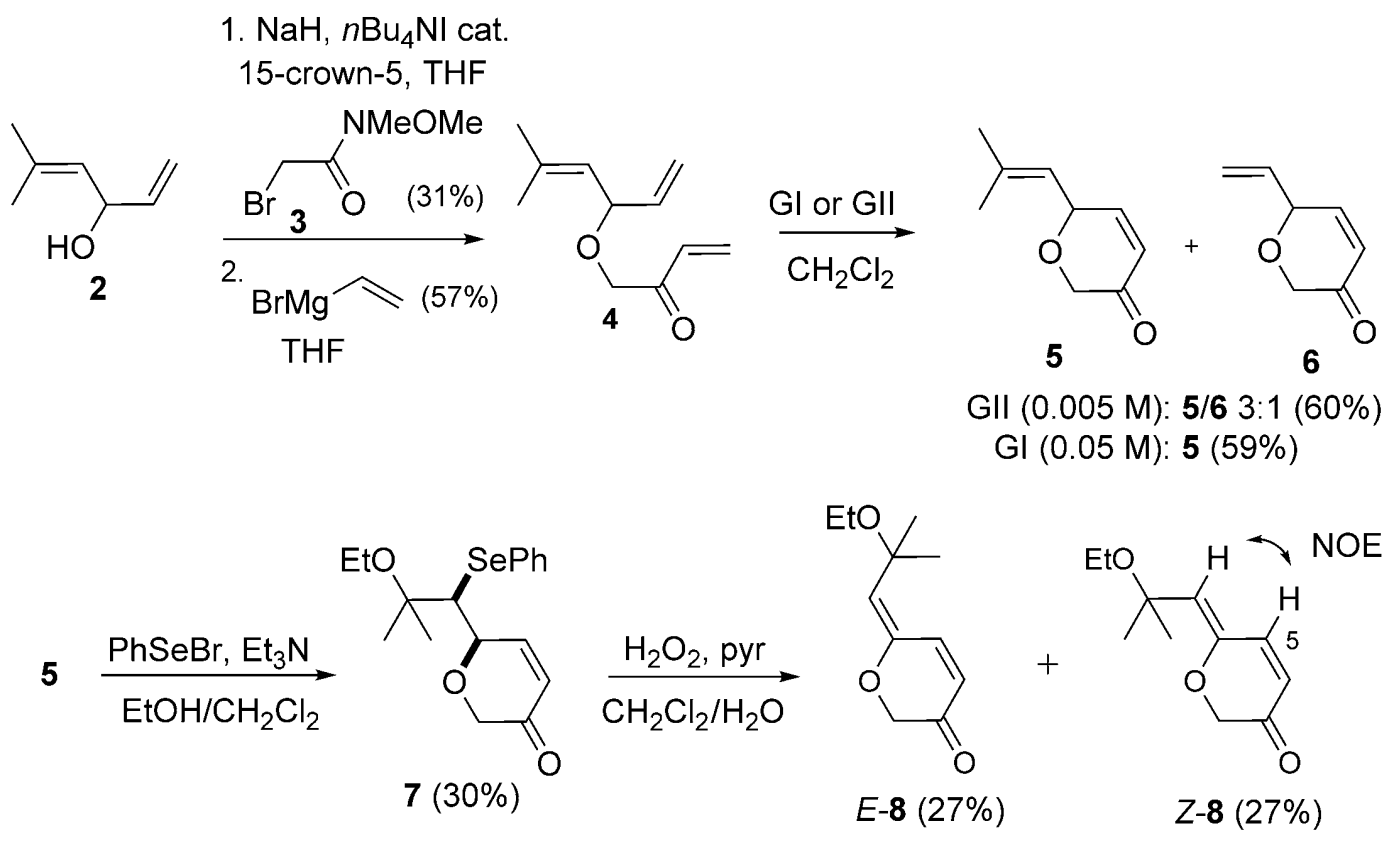
Synthesis of dihydropyran **8**.

So as to establish the viability of the above strategy (Scheme 1), we first examined a simplified system that lacked the α‐side‐chain (i.e., R=H). *O*‐Alkylation^7^ of the bis allylic alcohol **2** (Scheme 2, obtained by addition of vinylmagnesium bromide to 3‐methyl‐2‐butenal),^8^ using the α‐bromo Weinreb amide **3** (prepared from bromoacetyl bromide and *N*,*O*‐dimethylhydroxylamine),^9^ proceeded in modest yield. Subsequent addition of vinylmagnesium bromide gave the acid‐labile enone **4**.^10^ RCM using Grubbs II cat. (GII) produced an inseparable 3:1 mixture of desired dihydropyranone **5** and terminal olefin‐containing dihydropyranone **6**; the latter likely being generated from enone **4** by competing initiation at the *gem*‐dimethyl‐substituted olefin. The less reactive Grubbs I cat. (GI) prevented metathetical involvement of the more sterically demanding *gem*‐dimethyl‐substituted olefin, and delivered exclusively the desired dihydropyranone **5**.

**Scheme 3:**
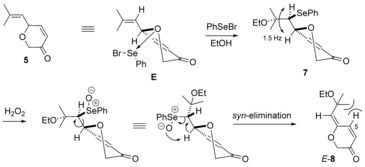
Rationale for stereochemical outcome of ethoxyselenation‐selenoxide elimination sequence.

Ethoxyselenation of dihydropyranone **5** using PhSeBr/ EtOH in the presence of Et_3_N5c delivered ethoxyselenide **7**, isolated as a single diastereoisomer. The same level and sense of diastereoselectivity (typically ^3^ *J*_OCH→SeCH_ 1.5 Hz, Scheme 3) was also found in the ethoxyselenation of all dihydropyranones examined later in this work, including selenide **23** (Scheme 7) where the relative configuration was established by single crystal X‐ray diffraction studies.^11^ Selenation is likely to occur from a conformer (**E**, Scheme 3) of dihydropyranone **5**, where the isobutenyl group is pseudo‐equatorial, and allylic strain is minimised. The cyclic ether oxygen atom may direct approach of the electrophilic selenium species by coordination12a (as shown in **E**); this coordination would also enhance the e‐σ^*^ acceptor ability of the ring C−O bond, thus increasing the relative e‐σ donor ability of the allylic ring C−C bond (vs. the ring C−O bond), which already preferentially directs the attack *anti* to itself. This diastereoselectivity is also found in methoxyhalogenation of α‐isobutenyl‐substituted 5‐ and 6‐membered cyclic ethers.^12^

Hydrogen peroxide‐induced oxidation^13^ of ethoxyselenide **7**, followed by *syn*‐elimination of the in situ formed selenoxide, gave enol ether **8** as a separable mixture of *E*‐/*Z*‐isomers (Scheme 2). Starting from the single diastereoisomer of ethoxyselenide **7**, this sequence was expected to deliver only the *E*‐isomer (Scheme 3). TLC monitoring of the reaction showed initial formation of the *E*‐isomer, which isomerises under the reaction conditions and on standing.^14^ One possible mode of isomerisation is via reversible conjugate addition to the δ‐position of the dienone. The configurational lability of the *E*‐isomer could be explained by A^(1,3)^ strain: C(5)*H*‐ (vs. ring *O*‐) interaction with CMe_2_OEt (see *E*‐**8**, Scheme 3). Configurational lability of the exocyclic olefin was also found in the other γ‐alkylidenedihydropyrans subsequently synthesised. *E*‐/*Z*‐Configurations were assigned by comparison of the C(5)*H* chemical shifts (>8.0 ppm and 7.0–7.5 ppm, respectively).

The above model study showed that a RCM/ ethoxyselenation‐selenoxide elimination strategy could be used to form the cross‐conjugated pyranone motif. The ethoxyselenation‐selenoxide elimination sequence proved to be a mild method to access the sensitive enol ether functionality. In addition, the configurational lability of the *E*‐isomer suggested that the natural product was more likely to possess a *Z*‐configured exocyclic enol ether.

### Studies towards the originally reported structure

#### Synthesis of dihydropyranone **14**

**Scheme 4:**
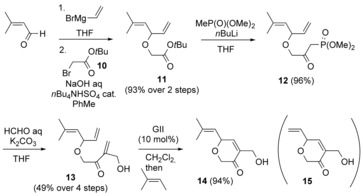
Synthesis of key intermediate, dihydropyranone **14**.

The viability of the above model study encouraged investigation of a system bearing an α‐side‐chain appropriate for aruncin B. *tert*‐Butyl α‐bromoacetate (**10**, Scheme 4) proved a more effective partner than the Weinreb amide derivative **3** (Scheme 2) for the *O*‐alkylation of 3‐methyl‐2‐butenal‐derived crude alcohol **2**; the latter being achieved under mild phase‐transfer catalysis (Scheme 4).^15^ After conversion of the resulting ester **11** to its corresponding β‐keto phosphonate **12**,^16^ Villiéras modification^17^ of the Horner–Wadsworth–Emmons reaction provided the RCM precursor, triene **13**, while installing the required extra carbon atom at the α‐position (missing in model triene **4**). Triene **13** also possessed a greater (acid) stability than triene **4**. This sequence could be carried out with either diethyl or dimethyl methylphosphonate in similar yields (50–60 % over 2 steps); however, dimethyl methylphosphonate was preferred due to its higher water solubility, enabling removal of excess during aqueous workup, rather than by distillation. In contrast to the model study, RCM of triene **13** using GI did not give hydroxy dihydropyranone **14** in a useful yield (≈25 %), probably due to the slightly more sterically demanding nature of the α‐hydroxymethyl‐bearing olefin partner. GII proved more promising, giving dihydropyranone **14** along with dihydropyranone **15** (ca. 9:1 mixture, inseparable); however, the latter could be converted to **14** by subsequent addition of excess 2‐methyl‐2‐butene.^18^ Dihydropyranone **14** possesses all the carbon atoms of the originally assigned core structure of aruncin B (**1**).^2^

#### Original late‐stage function group interconversion (FGI) attempt

**Scheme 5:**
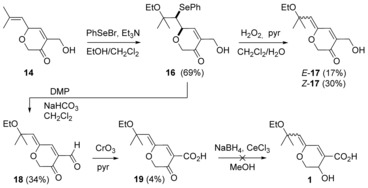
First attempt at late‐stage FGI of dihydropyranone **14**.

The ethoxyselenation‐selenoxide elimination sequence previously described (Scheme 2) was carried out on dihydropyranone **14** (Scheme 5) to give, via ethoxyselenide **16**, a (separable) mixture of *Z*‐/*E*‐enol ethers **17**. Unfortunately, these intermediates undergo rapid decomposition on standing and could not be used in a subsequent (oxidation) step. However, oxidation of the ethoxyselenide **16** using excess Dess–Martin periodinane (DMP) resulted in simultaneous selenide oxidation‐selenoxide elimination and oxidation of the primary alcohol to the corresponding aldehyde,^19^ resulting in the more stable *Z*‐ketoaldehyde **18**. Oxidation to the ketoacid **19**, proved very challenging, proceeding at best in only 4 % yield using CrO_3_ in pyridine;^20^ Luche reduction^21^ of ketoacid **19** failed to deliver aruncin B (**1**).

#### Redox interchange and FGI endgame

**Scheme 6:**
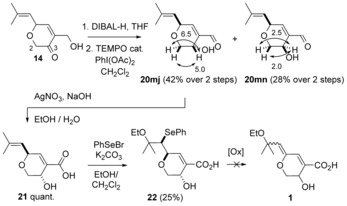
Redox interchange from dihydropyranone **14** with conformational analysis (^3^ *J*_H→H_ in Hz).

The above results suggested that it might be necessary to have the correct ring and α‐side‐chain oxidation levels in place prior to installation of the sensitive enol ether functionality; an initial redox interchange was therefore considered. 1,2‐Reduction of dihydropyranone **14** using DIBAL‐H,^22^ followed by selective TEMPO‐catalysed oxidation of the primary alcohol^23^ delivered hydroxyaldehyde **20** (3:1 d.r., Scheme 6). For ease of spectral analysis diastereoisomer separation was carried out at this point and the rest of the sequences presented were carried out on the major diastereoisomer (**20 mj**). Relative stereochemistry was originally assigned by comparison of ^3^ *J*_2→3_ values of the major contributing half‐chair conformer of each diastereoisomer. In both diastereoisomers, the isobutenyl group is expected to be pseudo‐equatorial; for the major diastereoisomer, the higher ^3^ *J*_2→3_ values (≥5 Hz) observed are characteristic^24^ for the oxygen substituent residing pseudo‐equatorial, corresponding to the *trans* isomer **20 mj**. For the minor diastereoisomer **20 mn**, lower ^3^ *J*_2→3_ values (<3 Hz) are characteristic for the oxygen substituent being pseudo‐axial. This preference for the formation of the *trans* isomer has also been found in the 1,2‐reduction of a 4‐substituted α‐(hydroxymethyl)cyclohexanone.^25^ Oxidation of the hydroxyaldehyde **20 mj** using alkaline AgNO_3_,^26^ delivered the hydroxyacid **21**. Reaction of the latter with PhSeBr in the presence of an inorganic base was used to obtain ethoxyselenide acid **22**. Various oxidation conditions (H_2_O_2_, NaIO_4_, NaIO_4/_ NaHCO_3_, *m*‐CPBA/ *i*Pr_2_NH or O_3_) were examined with acid **22** to lastly install the enol ether, but all failed to generate aruncin B (**1**); only decomposition was observed.

**Scheme 7:**
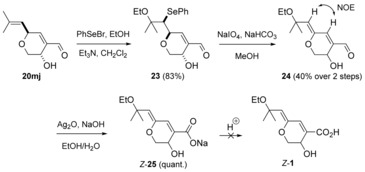
Synthesis of sodium salt *Z*‐**25** of the postulated structure of aruncin B.

The efficiency and chemoselectivity of the above aldehyde to acid oxidation (**20 mj** → **21**) encouraged consideration of leaving such an oxidation to the last step. Initial ethoxyselenation of hydroxyaldehyde **20 mj** proceeded again diastereoselectively (Scheme 7). The relative stereochemistry was confirmed by single crystal X‐ray diffraction studies on the 3,5‐dinitrobenzoate derivative of ethoxyselenide **23** 11 (see Supporting Information). This analysis served not only to confirm the ethoxyselenation diastereoselectivity rationalised earlier (Scheme 3), but also the diastereoselectivity in the DIBAL‐H‐induced 1,2‐reduction of dihydropyranone **14** (Scheme 6). Subsequent oxidation of selenide **23** using NaIO_4_ resulted in the *Z*‐enol ether **24**. Oxidation to the acid, using silver(I) oxide in the presence of NaOH^27^ gave the sodium salt **25** which corresponded to the conjugate base of the originally reported structure of aruncin B (**1**). Various mild acidification conditions were then examined (workup using pH 6 buffer solution, low temperature addition (−78 °C) of accurately measured 1.0 equiv of HCl, carboxylic acid‐supported ion‐exchange resin (amberlite IR‐50)), but only led to decomposition.

Since the geometry of the enol ether was not unambiguously assigned in the original isolation report of aruncin B (**1**, Figure 1), we were interested in establishing whether the *E*‐isomer would present a higher stability towards acidic conditions. As noted earlier, the *E*‐ → *Z*‐isomerisation (Scheme 2) was suspected to occur in part by reversible conjugate addition to the δ‐position of the dienone **17**, **18** and dienal **24**; we therefore considered a slightly less electron‐withdrawing system, such as a dienoate, might retard the rate of isomerisation and enable access to the *E*‐enol ether configuration.

**Scheme 8:**
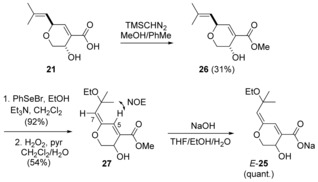
Synthesis of sodium salt *E*‐**25** of the postulated structure of aruncin B.

TMSCHN_2_‐induced esterification of acid **21** gave hydroxyester **26** (Scheme 8). Ethoxyselenation, followed by oxidation resulted in the formation of the *E*‐enol ether **27**, which showed a much lower rate of isomerisation compared with dienal **24**. Subsequent saponification gave the sodium salt *E*‐**25**, which however, similarly to its *Z*‐isomer, could not be acidified.

The above results (attempted conversion of **19**, **22**, *Z*‐**25** and *E*‐**25** to **1**) suggested that structure **1** could not exist (as a free acid) nor correspond to aruncin B: it presents intrinsic elements of instability. Although we were unable to isolate any decomposition product(s) that may have helped to clarify the decomposition pathway(s), we consider the carboxylic acid functionality likely initiates decomposition via ethoxy protonation leading to facile loss of EtOH from the tertiary allylic position assisted by the enol ether moiety.

#### Synthesis of regioisomeric ether

**Figure 2:**
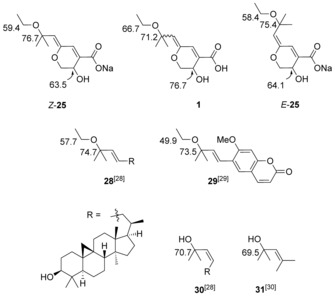
Selected ^13^C NMR data for **1**, *E*‐/*Z*‐**25** and previously reported tertiary allylic ethyl ethers and alcohols.

Comparison of ^13^C NMR data for *Z*‐**25** and *E*‐**25** with reported values for aruncin B (**1**) indicated notable inconsistencies in the C(3), C(8), and C(10) chemical shifts (Figure 2), and we therefore considered that the site of etherification could be misassigned.

Further detailed comparison with ^13^C NMR data of other known tertiary allylic ethyl ethers (**28** 28 and (*E*)‐*O*‐ethylsuberenol (**29**),^29^ Figure 2) and alcohols (**30** 28 and **31** 30), suggested that while the C(8) and C(10) chemical shifts observed in Na salts *Z‐* and *E*‐**25** are not anomalous,^28^, ^29^ the shift observed and originally assigned in the natural isolate is more consistent with the tertiary allylic position being an alcohol.^29^, ^30^

**Scheme 9:**
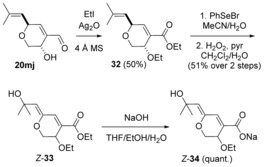
Synthesis of the regioisomeric structure **34**.

In addition to the above analytical considerations, the presence of a free hydroxy group at the tertiary allylic position might provide a lower acid sensitivity, due to its lower basicity compared with the ethoxy group, thus limiting initial protonation of the oxygen atom, suspected to trigger decomposition by loss of H_2_O or EtOH, respectively. We therefore focused on synthesising an alternative regioisomeric structure (**34**, Scheme 9) featuring the ethyl ether at C(3).

*O*‐Ethylation of hydroxyaldehyde **20 mj** in neat EtI in the presence of Ag_2_O^31^ and 4 Å MS, was accompanied by oxidation of the aldehyde to the corresponding acid and subsequent esterification to give the diethyl ether ester **32**. In a similar fashion to ethoxyselenation described previously, hydroxyselenation of ester **32** could be carried out using PhSeBr in MeCN/H_2_O,^32^ followed by oxidation with hydrogen peroxide to give *Z*‐enol ether **33**. Obtention of the *Z*‐isomer contrasts with the results observed with the dieneoate **27**. ^1^H NMR analysis of the crude mixture following hydroxyselenation‐oxidative elimination of ester **32** indicated traces of *E*‐**33**: chemical shifts for C(5)*H* (and C(7)*H*) 8.18 (and 5.51 ppm) (CDCl_3_), respectively; these values are higher than those seen for dienoate *E*‐**27** (8.03 (and 5.35 ppm) (CDCl_3_), respectively). This difference may indicate slightly higher electron deficiency in dieneoate *E*‐**33** compared with *E*‐**27**, which might account for the greater *E*‐→Z‐isomerisation of dieneoate **33**. Saponification of dienoate *Z*‐**33** delivered the corresponding sodium salt **34**, which however featured similar acid sensitivity to sodium salts **25**.

The acid instability of the dihydropyranol sodium salt derivatives and the inconsistencies observed in the analytical data led us to conclude that the originally assigned structure of aruncin B or the *O*‐ethyl variant could not be correct, and a more profound structural revision was necessary.

### Studies towards a newly postulated structure

#### The Z‐γ‐alkylidenebutenolide hypothesis

**Figure 3:**
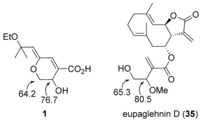
Comparison of selected ^13^C NMR data between **1** and eupaglehnin D (**35**).^35^

Reported UV data for aruncin B are consistent with an γ‐alkoxy‐α,β,γ,δ‐unsaturated carboxyl motif being present in the molecule.^33^, ^34^ However, acid sensitivity of the tertiary allylic ether (alcohol) motif cast doubt on its coexistence with the carboxylic acid functionality, and we therefore considered that the carboxyl motif could be esterified. The ^13^C NMR shift for the ring CH_2_ (in the original assignment) suggested a better match for a primary alcohol rather than an ether (Figure 3).^35^ As discussed earlier (Figure 2), the tertiary allylic position most likely corresponds to a free alcohol, with the ethyl group being borne by a secondary alcohol and the reported ^13^C shift for C(3) is more consistent for an ether rather than a free alcohol (Figure 3).

**Figure 4:**
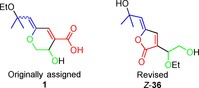
Alternative structure **36**.

These considerations led us to postulate a γ‐alkylidenebutenolide *Z*‐**36** as an alternative structure for aruncin B (Figure 4), with the ester being part of the ring and the dioxygenated moiety being a side‐chain featuring a free primary alcohol and an ethyl ether of a secondary alcohol.

**Figure 5:**
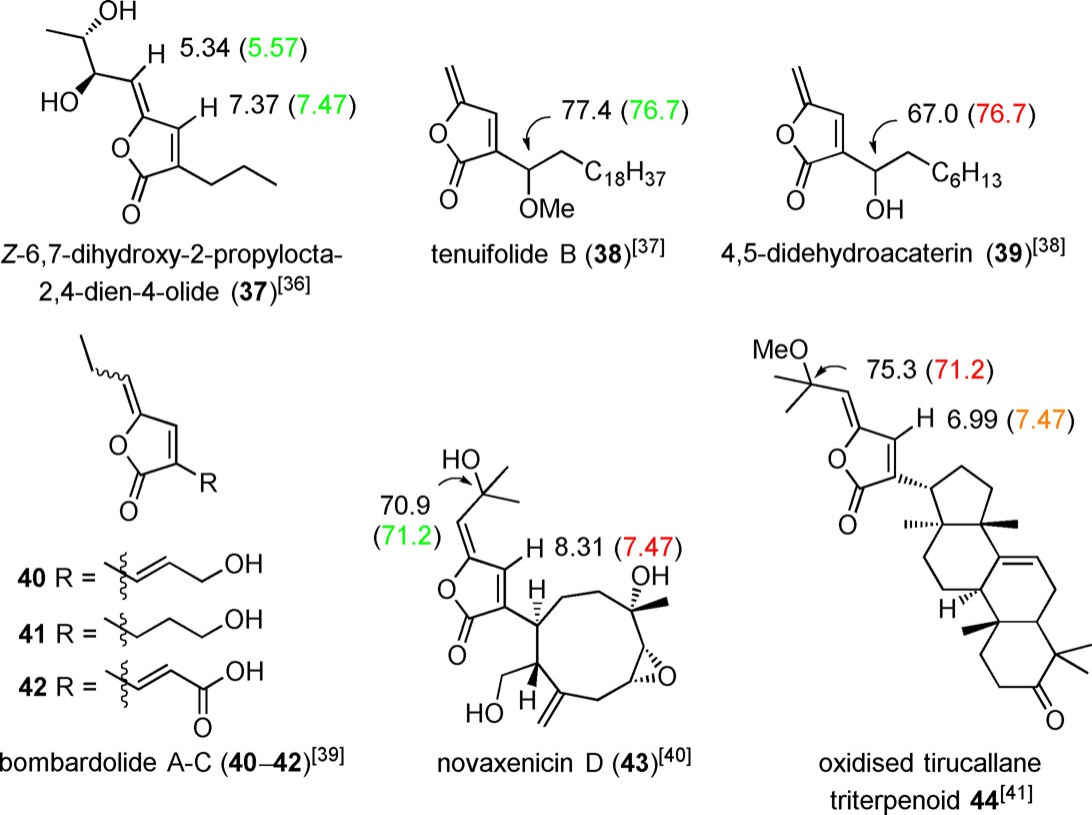
Naturally occuring γ‐alkylidenebutenolides, with selected NMR data compared against related reported values for aruncin B (in parentheses).

The γ‐alkylidenebutenolide motif is found in various previously reported natural products (**37**–**44**, Figure 5),^36^, ^37^, ^38^, ^39^, ^40^, ^41^ including the more complex novaxenicin D (**43**)^40^ and an oxidised tirucallane triterpenoid **44**.^41^ Comparison of ^1^H NMR shifts with butenolide **37**,^36^ is indicative of a *Z*‐enol ether. Moreover, ^13^C NMR values of the secondary alcohol (ether) centre observed in compounds **38** 37 and **39**,^38^ are also consistent with the site of etherification being at the secondary alcohol. Comparison between **43** and **44**, is also consistent with the tertiary allylic position being an alcohol. The revised assignment of aruncin B as **36** was only contradicted by IR data, with an anticipated C=O stretch value for this type of butenolide of 1755 cm^−1^,^40^ and a reported value^2^ of 1724 cm^−1^ (KBr disc). However, as we were informed that the original IR spectrum could not be located and none of the natural isolate was available for further analysis,^42^ synthetic studies towards the revised structure were undertaken.

### Model study

**Scheme 10:**
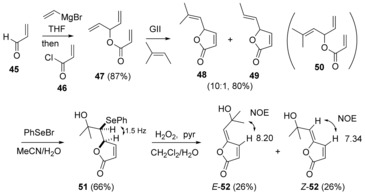
Synthesis of model butenolide **52** with ^1^H NMR δ values in ppm highlighted.

Although there are many known methods to γ‐alkylidenebutenolides,^43^ we were interested in studying whether a RCM/ hydroxyselenation‐selenoxide elimination sequence, which proved successful to the γ‐ethylidenedihydropyranoate compounds, would also enable access to the γ‐ethylidenebutenolide motif. We first examined a synthesis of the model butenolide **52** (Scheme 10), lacking the α‐dioxygenated side chain. Addition of vinylmagnesium bromide to acrolein,^44^ followed by trapping of the in situ generated magnesium alcoholate by acryloyl chloride gave the RCM precursor, triene **47** (Scheme 10). RCM‐cross‐metathesis (CM) cascade^45^ using GII in the presence of 2‐methyl‐2‐butene delivered the desired lactone **48**, as an inseparable (10:1) mixture with lactone **49**.^18^, ^46^ This RCM‐CM strategy avoids the intermediancy of triene **50** (from 3‐methyl‐2‐butenal and vinylmagnesium bromide, as described earlier), which would be required in a classical RCM approach and proved to be to prone to allylic rearrangement (during aqueous workup and chromatographic purification). As observed with the 6‐membered ring system (Scheme 3), hydroxyselenation of lactone **48** proceeded with complete diastereoselectivity; the relative stereochemistry of hydroxyselenide **51** was assigned by analogy with the 6‐membered ring system, on the basis of similar ^3^ *J*_OCH→SeCH_ values (≈1.5 Hz). The hydroxyselenide derived from lactone **49** was removed during chromatographic purification of hydroxyselenide **51**. Finally oxidation‐selenoxide elimination of **51** delivered the *Z*‐ and *E*‐isomers of **52**. As in the dihydropyranone series, initial *E*‐isomer formation and isomerisation over the time was observed, which again indicates a thermodynamic preference for *Z*‐geometry. ^1^H NMR β‐ =CH data (8.20 and 7.34 ppm for *E*‐ and *Z*‐**52**, respectively; 7.47 ppm for aruncin B (**1**)) indicates aruncin B should possess *Z*‐geometry.

#### First‐generation synthesis of butenolide **36**

**Scheme 11:**
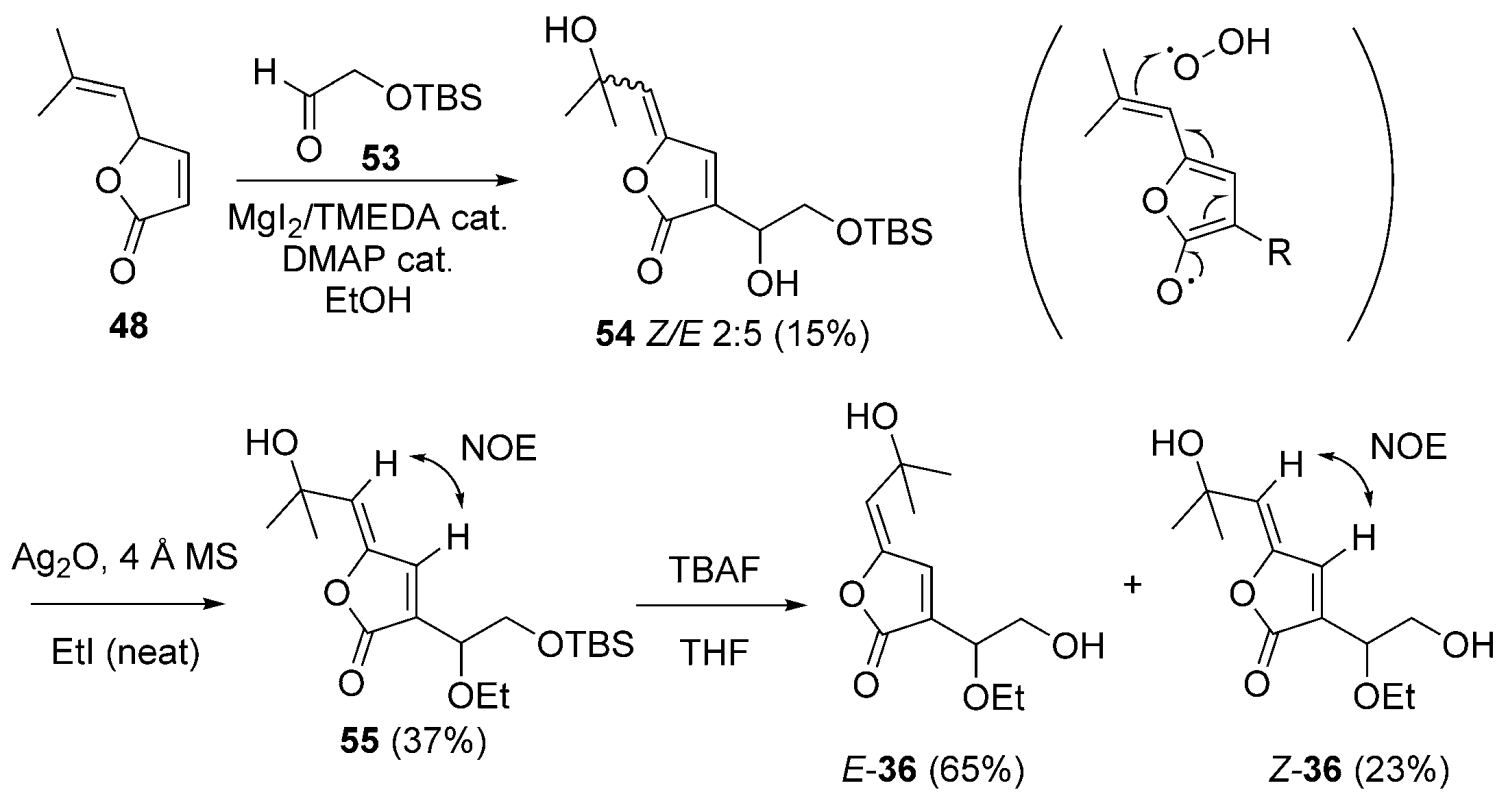
First synthesis of alcohol *Z*‐**36**, the true structure of aruncin B.

With the viability of a RCM/ hydroxyselenation‐selenoxide elimination sequence to the γ‐alkylidene butenolide motif established (Scheme 10), the synthesis of the butenolide **36** via α‐functionalisation of lactone **48** was examined (Scheme 11), using a Morita–Baylis–Hillman (MBH) reaction, followed by appropriate FGI and hydroxyselenation‐selenoxide elimination.

Remarkably, MBH reaction of lactone **48** with aldehyde **53** 47 using a MgI_2_/TMEDA complex‐DMAP catalytic system^48^ delivered butenolide **54** as an inseparable *E*‐/*Z*‐mixture. The concomitant and fortuitous allylic oxidation presumably occured via hydrogen atom abstraction at the bis‐allylic position; this aerobic oxidation was not observed on the 6‐membered ring system, and allylic hydrogen atom abstraction is probably favoured on the 5‐membered ring system by generation of the aromatic oxyfuran radical (show in parenthesis in Scheme 11). This unexpected oxidation avoids carrying out the hydroxyselenation‐selenoxide elimination steps. Reaction of butenolide **54** in neat ethyl iodide in the presence of Ag_2_O, facilitated *O*‐alkylation of the secondary alcohol, to give ether **55** as a single (*Z*‐) geometric isomer. Fluoride‐induced removal of the TBS group gave the free primary alcohol **36**. Surprisingly, the presence of fluoride apparently promotes loss of geometrical integrity of the exocyclic unsaturation, to give the alcohol **36** as a (separable) 3:1 *E*‐/*Z*‐isomeric mixture; the major product being the anticipated thermodynamically disfavored isomer. Analytical data recorded for alcohol *Z*‐**36** corresponded to those reported for aruncin B (see Supporting Information for details), except for the IR C=O stretch which had the value as discussed and anticipated earlier (1755 cm^−1^, film or KBr disc).

Although the synthetic route shown in Scheme 11 delivered sufficient material to confirm the true structure of aruncin B, it was impared by two major issues: 1) transformation of butenolide **48** to ether **54** was low yielding and poorly reproducible, and 2) loss of geometrical integrity during fluoride‐induced desilylation.

#### Second‐generation synthesis

**Scheme 12:**
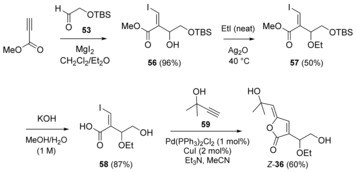
Synthesis of aruncin B (*Z*‐**36**) via β‐iodo MBH/ Sonogashira cross‐coupling‐5‐*exo‐dig* lactonisation sequence.

So as to address the above issues, we evaluated construction of the butenolide using a one‐pot Sonogashira cross‐coupling‐5‐*exo‐dig* lactonisation^49^ (Scheme 12). The dioxygenated side‐chain and the *Z*‐vinyl iodide functionality necessary for the final cross‐coupling were installed simultaneously, by way of a β‐iodo‐MBH^50^ between commercially available methyl propiolate and aldehyde **53** using in situ prepared MgI_2_ as a source of iodide.^51^ *O*‐Ethylation of the resulting iodoacrylate **56** was carried out as previously described (Scheme 11) to give ethyl ether **57** (Scheme 12), followed by hydrolysis to give the hydroxyacid **58**. Saponification was accompanied by desilylation, thus avoiding the potentially problematic use of fluoride in a subsequent step. The final step with 2‐methyl‐3‐butyn‐2‐ol (**59**) was carried out on up to 1 mmol scale, with a low catalyst loading, and provided sufficient aruncin B (*Z*‐**36**) (≈200 mg) for biological evaluation.

#### Possible origin of aruncin B

**Scheme 13:**
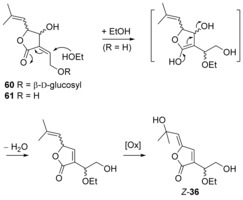
Potential origin of aruncin B.

Previously,^4^ we postulated that the low specific rotation of natural aruncin B compared with synthetic enantiopure samples,^52^ together with the unusual presence of the ethoxy group at the β′‐position of the true structure of aruncin B, indicated that the latter might not be an actual natural product. Rather, we suggested that aruncin B was an artefact of the isolation process, resulting from a poorly diastereoselective incorporation of EtOH (the solvent used for the extraction^2^) to a enantioenriched natural product precursor (Scheme 13). We originally mooted that the natural precursor of aruncin B was likely to be a α‐(2‐hydroxyethylidene)‐γ‐lactone.^4^ Following our initial disclosure of the structural revision of aruncin B, Zidorn and co‐workers, reported the isolation of natural products from the european plant *Aruncus dioicus* var. *vulgaris*.^53^ Some of the isolated monoterpenoids from the latter were identical to those isolated from *Aruncus dioicus* var. *kamtschaticus*.^2^ Based on our synthetic studies, Zidorn and co‐workers revised the structure of three of them. They also reported the isolation of γ‐lactone **60** and its aglycon **61** (Scheme 13); we consider the latter to be a reasonable precursor to aruncin B, through conjugate adddition of EtOH/ loss of H_2_O, followed by allylic oxidation—the latter resembling the oxidation observed in the generation of butenolide **54** (Scheme 11), also occurring in EtOH.

### Synthesis of analogues and biological evaluation

#### Synthesis of butenolide analogues of aruncin B

**Scheme 14:**
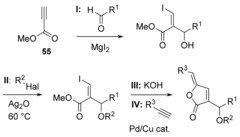
Synthesis of analogues for biological evaluation.

Taking advantage of the brevity and functional group tolerance of the second‐generation synthetic route to the correct structure of aruncin B (Scheme 12), this was applied to the synthesis of a range of analogues by varying different reaction components (Scheme 14).

Analogues of aruncin B at the α‐side‐chain were obtained using a variety of aldehyde during the β‐iodo‐MBH step. Higher yields were observed with non‐enolisable aldehydes (Table 1, entries 3–4). The rest of the sequence was followed as for aruncin B using EtI and alkynol **59**, except with R^1^=*t*Bu (Table 1, entry 3), for which the hydrolysis step was carried out using TMSOK in refluxing THF.^54^

**Table 1:** α‐Side‐chain analogues.

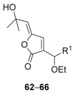						
Entry	R^1^	**I**. Yield [%]	**II**. Yield [%]	**III**. Yield [%]	**IV**. Yield [%]	Butenolide
1	CH_2_OBn	52	59	71	53	**62**
2	Me	41	44	71	54	**63**
3	*t*Bu	85	20	58	58	**64**
4	Ph	78	quant.	75	52	**65**
5	*n*C_8_H_17_	51	62	65	54	**66**

*O*‐Alkyl‐substituent‐analogues were obtained starting with iodo‐acrylate **56** using different alkyl halides in the presence of Ag_2_O (Table 2). The rest of the sequence was carried out as presented previously.

**Table 2:** *O*‐Alkyl analogues.

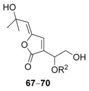					
Entry	R^2^‐Hal	**II**. Yield [%]	**III**. Yield [%]	**IV**. Yield [%]	Butenolide
1	(R^2^=H)	‐	60	42	**67**
2	MeI	77	98	67	**68**
3	*i*PrI	30	86	39	**69**
4	BnCl	46	64	52	**70**

Derivatives at the δ‐position were obtained by using different alkynes in the final cross‐coupling step with hydroxy acid **58** (Table 3).

**Table 3:** δ‐Substituent analogues.

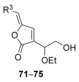			
Entry	R^3^	**IV**. Yield [%]	Butenolide
1	CMe_2_OMe	59	**71**
2	*i*Pr	51	**72**
3	CH_2_OH	50	**73**
4	Ph	60	**74**
5	*n*C_8_H_17_	39	**75**

### Biological evaluation

*E*‐ and *Z*‐**36**, and analogues **62**–**75** have been tested against Jurkat T cells, derived from acute T cell leukaemia, and primary human T cells. In the original aruncin B isolation studies,^2^, ^3^ assays were carried out with a high number of cells (5×10^5^) over 20 h.^55^ This short assay duration could potentially compromise detection of biological activity. Therefore, we tested over a longer time period (3 cell cycles), reducing the cell number (1.5–5×10^3^) to avoid confluence in control wells. The most potent compounds with our assays were also tested using the method described in the original publication, so as to enable comparison between the two methods.

*rac‐Z*‐Aruncin B, and (+)‐ and (−)‐*Z*‐aruncin B^52^ as well as *rac*‐*E*‐aruncin B, were first tested against Jurkat T cells. These aruncin B isomers showed similar activity (IC_50_ values (μm) of 16.0, 9.6, 9.7 and 9.1, respectively)^56^ and compared favourably with that originally reported for aruncin B (70 μm). The similar activity suggests that the side‐chain stereochemistry is probably not directly influential in the binding/ interaction with the target(s). Similar activity observed with the *E*‐isomer could be explained by isomerisation, once the compound is present in the cell medium.

Activity of the analogues against Jurkat T cells and human T cells was then tested. For the α‐side‐chain derivatives (Table 4, entries 1–5), although a clear trend is not discernable, modifications at this moiety strongly affect the activity. Aryl butenolide **65** (entry 4) showed good activity against Jurkat T cells but is poorly selective, whereas *n*‐octyl butenolide **66** (entry 5), showed greater activity against Jurkat T cells and a better selectivity towards the latter. Variation at the *O*‐ether substituent (Table 4, entries 6–9) does not seem to significantly affect activity or selectivity. Changes at the δ‐alkylidene substituent (Table 4, entries 10–14) appeared to be crucial; only butenolide **73** (entry 12), featuring a free alcohol showed activity. Compounds featuring a similar steric character to aruncin B on the δ‐alkylidene moiety, such as butenolides **71** and **72** (entries 10, 11), did not show significant activities. Those observations suggest that the presence of a hydrogen bond donor substituent is critical for the activity. Other substituents such as an aromatic ring or a long aliphatic chain, **74** and **75**, respectively (entries 13,14), did not show activity.

**Table 4:** IC_50_ [μm] values for aruncin B analogues.^56^

Entry	Butenolide	Derivatisation	Jurkat T cells	Human T cells
1	**62**	R^1^=CH_2_OBn	22.5	15.3
2	**63**	R^1^=Me	>50	>50
3	**64**	R^1^=*t*Bu	>50	>50
4	**65**	R^1^=Ph	11.6	9.4
5	**66**	R^1^=*n*C_8_H_17_	2.9	8.2
6	**67**	R^2^=H	26.3	23.5
7	**68**	R^2^=Me	12.5	16.5
8	**69**	R^2^=*i*Pr	23.0	>50
9	**70**	R^2^=Bn	5.6	9.1
10	**71**	R^3^=CMe_2_OMe	>50	>50
11	**72**	R^3^=*i*Pr	>50	>50
12	**73**	R^3^=CH_2_OH	3.6	1.2
13	**74**	R^3^=Ph	>50	>50
14	**75**	R^3^=*n*C_8_H_17_	>50	>50

To summarise the above observations, it appears that the exact nature of the α‐side‐chain is critical for the activity of the butenolide, although changes in the absolute configuration or the nature of the *O*‐alkyl group of the ether moiety are inconsequential. The nature of the δ‐alkylidene substituent proved crucial to the activity of the butenolide, with the presence of a free hydroxy group apparently necessary; but the exocyclic olefin geometry does not seem important (likely due to in situ isomerisation).

The most potent compounds were then retested, following the protocol described in the original aruncin B publications^2^, ^3^ (Table 5, method B). As in the original work, auraptene was used as a positive control for cytotoxicity. For all synthetic butenolides tested, method B gave much higher IC_50_ values than the protocol used in the current work (method A).

**Table 5:** Comparison of IC_50_ [μm] values between assay protocol in the current work (A) and that used in the original isolation study (B).^[a]^

Entry	Compound	Jurkat T cells		Human T cells Batch 1		Human T cells Batch 2	
		A	B	A	B	A	B
1	auraptene	83	57	>50	205	19.4	73
2	aruncin B (natural isolate)^2^, ^3^		*70* (lit.)				*>245* (lit.)
3	*rac*‐aruncin B (*Z*‐**36**)	16.0	>50	NT	NT	18.2	>50
4	**65**	11.6	32	13.3	NT	2.0	46
5	**66**	2.9	11	3.3	NT	1.1	14
6	**68**	12.5	>50	10.2	>50	12.8	NT

[a] NT=not tested.

## Conclusion

The *Z*‐ and *E*‐Na salts of the postulated structure of aruncin B were synthesised through an approach relying on formation of a 6‐membered ring intermediate by RCM followed by an oxyselenation‐selenoxide elimination sequence to install the key enol ether functionality. However, the free acids could not be obtained from these Na salts, suggesting intrinsic instability. A regioisomeric ether was first envisaged as an alternative structure for aruncin B, and its Na salt was synthesised using a related RCM/ hydroxyselenation‐selenoxide elimination sequence, but featured similar acid sensitivity. Instability together with inconsistencies in the analytical data collected from the various dihydropyran compounds suggested that aruncin B was another example of a compound of mistaken identity.^57^ A butenolide was proposed as a viable alternative structure for aruncin B, and was first synthesised using a related RCM/ hydroxyselenation‐selenoxide elimination sequence, demonstrating the versatility of the strategy for the synthesis of different ring size systems, and confirming the true structure of aruncin B. Unfortunately, installation of the α‐side‐chain proved difficult and a preferred approach was developed involving a Sonogashira cross‐coupling–5‐*exo‐dig* lactonisation strategy. This latter method also proved flexible, enabling the synthesis of 14 analogues for biological evaluation. The cytotoxic activity originally reported for aruncin B was confirmed, and some of the analogues (**66** and **70**) showed improved activity against malignant Jurkat T cells vs. normal T cells.

## Conflict of interest

The authors declare no conflict of interest.

## Supporting information

As a service to our authors and readers, this journal provides supporting information supplied by the authors. Such materials are peer reviewed and may be re‐organized for online delivery, but are not copy‐edited or typeset. Technical support issues arising from supporting information (other than missing files) should be addressed to the authors.

Supplementary
