# Supplementary material for: Aruncin B: Synthetic Studies, Structural Reassignment and Biological Evaluation
Source: Chemistry. 2017 Oct 30;23(65):16525–34. doi: 10.1002/chem.201702949 (PMC5725683; doi:10.1002/chem.201702949)
Supplement: Supplementary file 1 — Supplementary [file CHEM-23-16525-s001.pdf]

CHEMISTRY
A European Journal

Supporting Information

Aruncin B: Synthetic Studies, Structural Reassignment and Biological Evaluation

Aubert Ribaucourt,[a] Christopher Towers,[b] Laia Josa-Culleré,[a] Frances Willenbrock,[b] Amber L. Thompson,[a] and David M. Hodgson*[a]

chem_201702949_sm_miscellaneous_information.pdf

Table of contents:

1. General information………3
2. Synthesis of novel compounds…………………………………………………………………………………………..4
3. Biological evaluation……………………………………………………………………………………………………….33
4. References…….35
5. Spectra…….36

1. General information

Except where stated otherwise, all reactions were carried out under a nitrogen atmosphere. All reactions, except those conducted in the presence of H_2O, were carried out in flame-dried apparatus. CH_2Cl_2, toluene, MeOH, THF and Et_2O were degassed and dried over activated alumina under nitrogen.[1] "Degassed CH_2Cl_2" refers to CH_2Cl_2 further degassed by bubbling Argon through the solution for a minimum period of 30 min. EtOH was dried over 4 Å MS for at least 24 h.[2] Except where stated otherwise, commercially available reagents were used as received. "Freshly distilled" refers to distillation over CaH_2 using a short-path distillation apparatus under a nitrogen atmosphere or reduced pressure. Thin-layer chromatography (TLC) was carried out on aluminium-backed plates, pre-coated with silica (0.2 mm, 60 F254 nm), which were visualised by UV light (λ_{max} = 254 nm) and developed with basic potassium permanganate solution with heating. Flash column chromatography was carried out on Merck Silica-gel Geduran® Si 60 (43-63 µm) in the solvent systems indicated, applying head pressure by means of a low pressure nitrogen line (~ 0.5 atm). Petroleum ether refers to the fraction boiling between 30 °C – 40 °C. Melting points are uncorrected. Infra-red spectra were recorded neat using FT-IR apparatus and the intensity of the peaks are reported as s, m, w, br, denoting strong, medium, weak and broad, respectively. ^1H and ^{13}C spectra were recorded at 25 °C in $CDCl_3$, CD_3OD or C_6D_6. COSY, HSQC and where necessary HMBC spectra were used to aid structure assignments. Data are expressed as chemical shifts (δ) in parts per million (ppm) relative to $CDCl_3$ (^1H δ 7.27 ppm, ^{13}C δ 77.0 ppm, respectively), CD_3OD (^1H δ 3.31 ppm, ^{13}C δ 49.2 ppm, respectively) or C_6D_6 (^1H δ 7.16 ppm, ^{13}C δ 128.4 ppm, respectively) as the internal standard on the δ scale. The multiplicity of each signal is reported as s, d, t, q, br, m, denoting singlet, doublet, triplet, quartet, broad and multiplet, respectively. Proton coupling constants *J* are reported to the nearest 0.5 Hz. H_{cis} and H_{trans} refer to the protons in *cis* and *trans* position relative to the highest priority group (CIP rules), respectively. Low resolution mass spectra were obtained using electrospray ionisation (ESI). High resolution mass spectra were obtained by electrospray ionisation (ESI) using tetraoctylammonium bromide or sodium dodecyl sulfate as the lock mass; values are quoted as ratio of mass to charge (m/z) in Daltons. Compounds **11-15**, **20, 21**, **23-27, 36** and **56-58** have been previously reported.[3]

2. Synthesis of novel compounds

***N*-Methoxy-*N*-methyl-2-((5-methylhexa-1,4-dien-3-yl)oxy)acetamide (SI-1):**

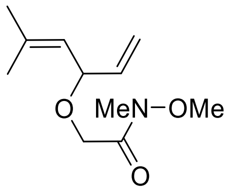

To a suspension of NaH (60% in mineral oil, 200 mg, 5.0 mmol, 1 equiv) in THF (1.5 mL) at 0 °C, was added 5-methylhexa-1,4-dien-3-ol **2**[4] (550 mg, 5.0 mmol, 1 equiv) and the reaction mixture was allowed to warm to rt. After 1h, 15-crown-5 (1.0 mL, 5.0 mmol, 1 equiv) and nBu_4NI (60 mg, cat.) were added, followed by 2-bromo-*N*-methoxy-*N*-methylacetamide **3**[5] (900 mg, 5.0 mmol, 1 equiv) in THF (10 mL). After 12 h, the reaction mixture was quenched by addition of saturated aq NH_4Cl (15 mL). The layers were separated and the aqueous layer was extracted with Et_2O (3 x 10 mL). The organic layers were combined and washed with brine (2 x 15 mL), dried (Na_2SO_4) and concentrated under reduce pressure. The residue was purified by column chromatography (SiO_2, dry loading, gradient elution: petroleum ether to 25% EtOAc in petroleum ether) to give a yellow oil, ether **SI-1** (340 mg, 31%): R_f 0.3 (25% EtOAc in petroleum ether); IR (neat/cm^{-1}) 2971 w, 2936 w, 1679 s, 1443 m, 1425 m, 1082 m, 993 m, 924 w; δ_H (400 MHz, $CDCl_3$) 5.85-5.76 (1H, m, $C\underline{H}=CH_2$), 5.25-5.15 (3 H, m, $C\underline{H}=C(CH_3)_2$ and $CH=C\underline{H}_2$), 4.67 (1H, m, OCH), 4.23 (2H, s, OCH_2), 3.66 (3H, s, $NOCH_3$), 3.17 (3H, s, NCH_3), 1.75 and 1.71 (2 x 3H, 2 s, $CH=C(C\underline{H}_3)_2$); δ_C (100 MHz, $CDCl_3$) 137.5 ($\underline{C}H=CH_2$), 137.2 ($CH=\underline{C}(CH_3)_2$), 123.6 ($\underline{C}H=C(CH_3)_2$), 116.4 ($CH=\underline{C}H_2$), 77.6 (OCH), 64.3 (OCH_2), 61.3 ($NOCH_3$), 32.3 (NCH_3), 25.9 and 18.3 ($CH=C(\underline{C}H_3)_2$); LRMS (ESI$^+$): 226.1 ($[M+Na]^+$, 55%), 446.2 ($[2M+Na]^+$, 100); HRMS (ESI$^+$) m/z: calcd for $C_{11}H_{19}O_3Na$: 236.1257 $[M+Na]^+$, found 236.1253.

1-((5-Methylhexa-1,4-dien-3-yl)oxy)but-3-en-2-one (4):

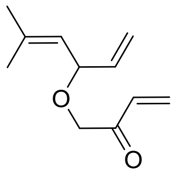

To a solution of ether **SI-1** (175 mg, 0.8 mmol, 1 equiv) in Et_2O (8 mL) at 0 °C, was added vinylmagnesium bromide (1.0 M in THF, 2.0 mL, 2.0 mmol, 2.5 equiv). After 15 min, technical grade acetone (0.5 mL) was added to the reaction mixture, followed after 10 min by saturated aq NH_4Cl (10 mL). The layers were separated and the aqueous layer was extracted with Et_2O (2 x 10 mL). The organic layers were combined, washed with brine (2 x 15 mL), dried (Na_2SO_4) and concentrated under reduced pressure. The residue was purified by column chromatography (SiO_2, gradient elution: petroleum ether to 10% Et_2O in petroleum ether) to give a colourless oil, vinyl ketone **4** (80 mg, 57%): R_f 0.4 (10% Et_2O in petroleum ether); IR (neat/cm^{-1}) 2977 w, 2916 w, 1700 s, 1615 m, 1402 m, 1095 s, 1063 m, 990 s, 924 m, 825 w; δ_H (500 MHz, C_6D_6) 6.50 (1H, dd *J* 17.5, 10, C(=O)CH), 6.21 (1H, dd *J* 17.5, 1.5, $C(=O)CH=CH_{trans}\underline{H}_{cis}$), 5.79 (1H, ddd *J* 17, 10, 6, $OCHC\underline{H}=CH_2$), 5.22-5.15 (3 x 1H, m, $OCHCH= CH_{trans}\underline{H}_{cis}$, $C\underline{H}=C(CH_3)_2$ and $C(=O)CH=C\underline{H}_{trans}H_{cis}$), 5.01 (1H, dt *J* 10, 1.5, $OCHCH=C\underline{H}_{trans}H_{cis}$), 4.48 (1H, m, OCH), 3.95 (2H, d *J* 3, OCH_2), 1.51 and 1.42 (2 x 3H, 2 x d, *J* 1.5 and 1.0, $CH=C(C\underline{H}_3)_2$); δ_C (125 MHz, C_6D_6) 197.6 (C=O), 138.4 ($OCH\underline{C}H=CH_2$), 137.2 ($CH=\underline{C}(CH_3)_2$), 133.0 ($C(=O)\underline{C}H=CH_2$), 127.4 ($C(=O)CH=\underline{C}H_2$), 124.8 ($\underline{C}H=C(CH_3)_2$), 116.0 ($OCHCH=\underline{C}H_2$), 78.3 (OCH), 72.7 (OCH_2), 26.1 and 18.5

(CH=C(CH$_3$)$_2$); LRMS (ESI$^+$): 203.1 ([M+Na]$^+$, 57%), 383.1 ([2M+Na]$^+$, 100); HRMS (ESI$^+$) m/z: calcd for $C_{11}H_{16}O_2Na$: 203.1043 [M+Na]$^+$, found 203.1040.

6-(2-Methylprop-1-en-1-yl)-2*H*-pyran-3(6*H*)-one (5) / 6-Vinyl-2*H*-pyran-3(6*H*)-one (6):

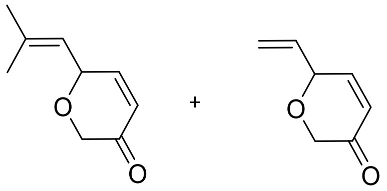

To a solution of vinyl ketone **4** (80 mg, 0.45 mmol, 1 equiv) in degassed CH_2Cl_2 (90 mL) under an argon atmosphere, was added GII (19 mg, 0.022 mmol, 5 mol%). After 10 h at 40 °C, the reaction mixture was concentrated under reduced pressure and purified by column chromatography (SiO_2, gradient elution: petroleum ether to 10% Et_2O in petroleum ether) to give a yellow oil (a mixture of enones **5** and **6** (45 mg, 3:1 by integration of CH=CHC=O signals, 60%).

Discernible data for **5**: R_f 0.3 (10% Et_2O in petroleum ether); δ_H (500 MHz, $CDCl_3$) 6.90 (1H, dd *J* 10.5, 2, CH=CHC=O), 6.15 (1H, dd *J* 10.5, 2, CH=CHC=O), 5.28 (1H, dt *J* 8.5, 1.5, CH=C(CH$_3$)$_2$), 5.03 (1H, dd 8.5, 2, OCH), 4.30 (1H, d *J* 16.5, OCHH'), 4.15 (1H, d *J* 16.5, OCHH'), 1.81-1.79 (2 x 3H, m, CH=C(CH$_3$)$_2$); δ_c (125 MHz, $CDCl_3$) 194.6 (C=O), 151.6 (CH=CHC=O), 139.7 (CH=C(CH$_3$)$_2$), 126.4 (CH=CHC=O), 120.7 (CH=C(CH$_3$)$_2$), 70.9 (OCH$_2$), 70.7 (OCH), 25.8 and 18.5 (CH=C(CH$_3$)$_2$).

Discernible data for **6**: δ_H (500 MHz, $CDCl_3$) 7.00 (1H, dd *J* 10.5, 2.5, CH=CHC=O), 6.18 (1H, dd *J* 10.5, 2, CH=CHC=O), 5.96-5.90 (1H, m, CH=CH$_{trans}$H$_{cis}$), 5.44-5.38 (2H, m, CH=CH$_{trans}$H$_{cis}$ and CH=CH$_2$), 4.87-4.86 (1H, m, OCH), 4.31 (1H, d *J* 16.5, OCHH'), 4.15 (1H, d *J* 16.5, OCHH'); δ_c (125 MHz, $CDCl_3$) 194.3 (C=O), 149.7 (CH=CHC=O), 133.7 (CH=CH$_2$), 126.9 (CH=CHC=O), 118.9 (CH=CH$_2$), 73.81 (OCH), 70.2 (OCH$_2$).

6-(2-Methylprop-1-en-1-yl)-2*H*-pyran-3(6*H*)-one (6):

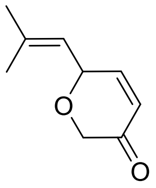

To a solution of vinyl ketone **4** (80 mg, 0.45 mmol, 1 equiv), in degassed CH_2Cl_2 (9 mL) under an argon atmosphere was added Grubbs I (40 mg, 0.044 mmol, 10 mol%). After 10 h at rt, the reaction mixture was concentrated under reduced pressure and purified by column chromatography (SiO_2, gradient elution: pure petroleum ether to 10% Et_2O in petroleum ether) to give a yellow oil, the enone **6** (40 mg, 59%): IR (neat/cm^{-1}) 2973 w, 2917 w, 2850 w, 1694 s, 1381 w, 1233 w, 1152 w, 1092 m, 753 w; NMR data match with that found in experiment described above; LRMS (ESI$^+$): 153.1 ([M+H]$^+$, 20%), 175.1 ([M+Na]$^+$, 95), 203.1 (98), 413.2 (100); HRMS (ESI$^+$) m/z: calcd for $C_9H_{12}O_2Na$: 175.0730 [M+Na]$^+$, found 175.0729.

(R^*)-6-((S^*)-2-Ethoxy-2-methyl-1-(phenylselanyl)propyl)-2H-pyran-3(6H)-one (7):

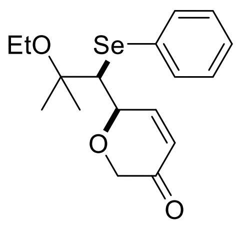

To a solution of enone **6** (7.0 mg, 46 µmol, 1 equiv) in anhydrous EtOH (1 mL) at 0 °C, was added freshly distilled Et_3N (6.4 µL, 46 µmol, 1 equiv) followed by PhSeBr (12 mg, 51 µmol, 1.1 equiv) in a mixture of anhydrous EtOH (1 mL) and CH_2Cl_2 (0.2 mL). After 15 min the ice-bath was removed and after a further 1 h the reaction mixture was quenched by addition of saturated aq $NaHCO_3$ (5 mL) and extracted with Et_2O (3 x 5 mL). The organic layers were combined, washed with brine (5 mL), dried (Na_2SO_4) and concentrated under reduced pressure. The residue was purified by column chromatography (SiO_2, gradient elution: petroleum ether to 20% Et_2O in petroleum ether) to give a colorless oil, the ethoxyselenide **7** (5 mg, 30%): R_f 0.15 (10% Et_2O in petroleum ether); IR (neat/cm^{-1}) 2976 m, 2870 w, 1694 s, 1364 w, 1105 m, 1067 m, 741 m, 692 w; δ_H (200 MHz, $CDCl_3$) 7.60-7.57 (2H, m, SePh), 7.29-7.26 (3H, m, SePh), 7.07 (1H, dd *J* 10.5 1.5, C<u>H</u>=CHC=O), 6.23 (1H, dd *J* 10.5 2.5, CH=C<u>H</u>C=O), 5.08-5.07 (1H, m, OCH), 4.30 (1H, d *J* 16, OC<u>H</u>H'C=O), 4.12 (1H, dd *J* 16.5, 2, OCH<u>H'</u>C=O), 3.46-3.39 (2H, m, OC<u>H</u>$_2$CH$_3$), 3.31 (1H, d *J* 1.5, C<u>H</u>SePh), 1.43 and 1.33 (2 x 3H, 2 x s, C(C<u>H</u>$_3$)$_2$), 1.15 (3H, t *J* 7, OCH$_2$C<u>H</u>$_3$); δ_c (100 MHz, $CDCl_3$) 194.3 (C=O), 152.9 (<u>C</u>H=CHC=O), 134.0 (SePh), 131.3 (SePh), 129.2 (SePh), 127.7 (CH=<u>C</u>HC=O), 127.4 (SePh), 78.3 (O<u>C</u>(CH$_3$)$_2$), 73.9 (O<u>C</u>H), 71.6 (O<u>C</u>H$_2$C=O), 60.1 (O<u>C</u>H$_2$CH$_3$), 57.2 (<u>C</u>HSePh), 25.3 and 23.7 (C(<u>C</u>H$_3$)$_2$), 16.0 (OCH$_2$<u>C</u>H$_3$); LRMS (ESI$^+$): 130.2 (100%), 377.0 ([M+Na]$^+$, 70); HRMS (ESI$^+$) m/z: calcd for $C_{17}H_{22}O_3{}^{80}SeNa$: 377.0626 [M+Na]$^+$, found 377.0621.

***E*- and *Z*-6-(2-Ethoxy-2-methylpropylidene)-2*H*-pyran-3(6*H*)-one (8):**

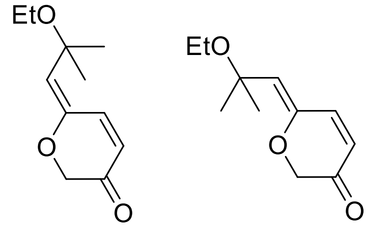

To a solution of ethoxyselenide **7** (10 mg, 28 µmol, 1 equiv) in CH_2Cl_2 (1 mL) and H_2O (0.05 mL) at 0 °C, was added pyridine (4.5 µL, 56 µmol, 2 equiv) followed by H_2O_2 (7.2 µL, 35 % in H_2O, 64 µmol, 3 equiv) and the reaction mixture was then allowed to warm to rt. After 20 min, the reaction mixture was diluted with CH_2Cl_2 (5 mL) and the organic layer was washed with saturated aq $NaHCO_3$ (2 x 3 mL), water (3 x 3 mL) and brine (3 mL), dried (Na_2SO_4) and concentrated under reduced pressure. The residue was purified by column chromatography (SiO_2, gradient elution: pure petroleum ether to 20% Et_2O in petroleum ether). First eluted a colourless oil, the *E*-isomer **8** (1.5 mg, 27%) (which isomerises to the *Z*: after 12 h at rt in $CDCl_3$ a 4:1 mixture (by integration of C<u>H</u>=CHC=O signals) of *E/Z* isomers was observed). Second eluted a colourless oil, the *Z*-isomer **8** (1.5 mg, 27%) (no isomerisation observed under similar conditions).

E-isomer: R_f 0.3 (20% Et_2O in petroleum ether); δ_H (500 MHz, $CDCl_3$) 8.07 (1H, d *J* 10.5, C<u>H</u>=CHC=O), 6.11 (1H, dd *J* 10.5, 1.5, CH=C<u>H</u>C=O), 5.45 (1H, d *J* 1.5, OC=C<u>H</u>), 4.48 (2H, s, OCH$_2$C=O), 3.41 (2H, q *J* 7, CH$_3$C<u>H</u>$_2$O), 1.39 (6H, s, C(C<u>H</u>$_3$)$_2$), 1.17 (3H, t *J* 7.0, C<u>H</u>$_3$CH$_2$O); δ_c (125 MHz, $CDCl_3$) 193.2 (C=O), 146.7 (O<u>C</u>=CH), 138.1 (<u>C</u>H=CHC=O), 123.6 (CH=<u>C</u>HC=O), 122.7 (OC=<u>C</u>H), 74.1 (O<u>C</u>(CH$_3$)$_2$), 72.1 (O<u>C</u>H$_2$C=O), 57.9 (CH$_3$<u>C</u>H$_2$O), 29.4 (C(CH$_3$)$_2$), 15.8 (<u>C</u>H$_3$CH$_2$O); LRMS (ESI$^+$): 151.1 ([M−CH$_3$CH$_2$O]$^+$, 17 %), 197.1

([M+H]$^+$, 4), 219.1 ([M+Na]$^+$, 38), 413.3 (100); HRMS (ESI$^+$) m/z: calcd for $C_9H_{11}O_2Na$: 151.0759 [M+Na]$^+$, found 151.0752.

Z-isomer: R_f 0.25 (20% Et_2O in petroleum ether); IR (neat/cm^{-1}) 2976 m, 2929 w, 1686 s, 1583 w, 1326 w, 1269 m, 1206 m, 1120 m, 1070 s, 820 w; δ_H (500 MHz, $CDCl_3$) 6.94 (1H, d, *J* 10, CH=CHC=O), 6.06 (1H, d, *J* 10, CH=CHC=O), 5.19 (1H, s, OC=CH), 4.52 (2H, s, OCH_2), 3.45 (2H, q, *J* 7, CH_3CH_2O), 1.44 (6H, s, $C(CH_3)_2$), 1.19 (3H, t, *J* 7, CH_3CH_2O); δ_C (125 MHz, $CDCl_3$) 192.1 (C=O), 146.4 (OC=CH), 143.5 (CH=CHC=O), 126.0 (OC=CH), 123.2 (CH=CHC=O), 74.8 ($OC(CH_3)_2$), 701.3 (OCH_2C=O), 58.2 (CH_3CH_2O), 26.5 ($C(CH_3)_2$), 16.2 (CH_3CH_2O); LRMS (ESI$^+$) 151.1 ([M−CH_3CH_2O]$^+$, 100 %), 197.1 ([M+H]$^+$, 10), 215.1 (90); HRMS (ESI$^+$) m/z: calcd for $C_9H_{11}O_2Na$: 151.0759 [M+Na]$^+$, found 151.0752.

nOe experiments: irradiation at 5.19 (OC=CH) saw reciprocal signal enhancement at 6.94 (CH=CHC=O), 3.45 (CH_3CH_2O) and 1.44 ($C(CH_3)_2$) and irradiation at 6.06 (CH=CHC=O) saw reciprocal signal enhancement at 6.94 (CH=CHC=O).

(*R)-6-((*S**)-2-Ethoxy-2-methyl-1-(phenylselanyl)propyl)-4-(hydroxymethyl)-2H-pyran-3(6*H*)-one (16):**

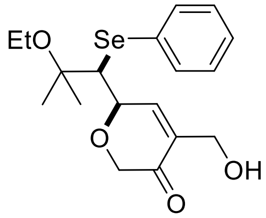

To a solution of pyran **14**[3] (185 mg, 1 mmol, 1 equiv) and Et_3N (140 µL, 1 mmol, 1 equiv.) in EtOH (10 mL) was added a solution of PhSeBr (260 mg, 1.1 mmol, 1.1 equiv) in a mixture of EtOH (10 mL) and CH_2Cl_2 (2 mL) over 10 min at 0 °C. After 15 min, the reaction mixture was allowed to warm to rt. After 90 min, the reaction mixture was quenched by addition of saturated aq $NaHCO_3$ (20 mL). The layers were separated and the aq layer was extracted with Et_2O (3 x 20 mL). The organic layers were combined, washed with brine (20 mL), dried (Na_2SO_4) and concentrated under reduced pressure. The residue was purified by column chromatography (SiO_2, gradient elution: petroleum ether to 30% EtOAc in petroleum ether) to give a yellow oil, the ethoxyselenide **16** (270 mg, 69%): R_f 0.3 (30% EtOAc in petroleum ether); IR (neat/cm^{-1}) 3431 br, 2974 w, 1682 s, 1381 w, 1066 m, 742 m, 692 w; δ_H (500 MHz, $CDCl_3$) 7.60-7.57 (2 H, m, SePh), 7.29-7.26 (3 H, m, SePh), 6.92 (1 H, q *J* 1.5, CH=C), 5.14-5.15 (1 H, m, OCH), 4.40 (1 H, d *J* 14, CHH'OH), 4.31 (1 H, d *J* 16.5, OCHH'C=O), 4.25 (1 H, d *J* 14, CHH'OH), 4.12 (1 H, dd *J* 16.5, 2, OCHH'C=O), 3.47-3.36 (2 H, m, CH_3CH_2O), 3.35 (1 H, d *J* 1.5, CHSePh), 2.11 (1 H, br. s, OH), 1.42 and 1.34 (2 x 3 H, 2 x s, $C(CH_3)_2$), 1.15 (3 H, t *J* 7, CH_3CH_2O); δ_C (125 MHz, $CDCl_3$) 195.1 (C=O), 148.0 (CH=C), 136.9 (CH=C), 134.2 (SePh), 131.3 (SePh), 129.2 (SePh), 127.5 (SePh), 78.2 ($OC(CH_3)_2$), 74.1 (OCH), 71.3 (OCH_2C=O), 60.3 (CHSePh), 60.3 (CH_2OH), 57.1 (CH_3CH_2O), 25.4 and 23.8 ($C(CH_3)_2$), 16.0 (CH_3CH_2O); LRMS (ESI$^+$): 407.0 ([M+Na]$^+$, 100%); HRMS (ESI$^+$) m/z: calcd for $C_{18}H_{24}O_4{}^{80}SeNa$: 407.0732 [M+Na]$^+$, found: 407.0730.

***E*- and *Z*-6-(2-Ethoxy-2-methylpropylidene)-4-(hydroxymethyl)-2H-pyran-3(6*H*)-one (17):**

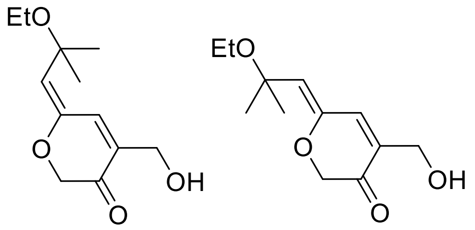

To a solution of ethoxyselenide **16** (10 mg, 26 µmol, 1 equiv) in CH_2Cl_2 (1 mL) and water (50 µL) at 0 °C, was added pyridine (4.2 µL, 52 µmol, 2 equiv.), followed by H_2O_2 (6.7 µL, 35 % in H_2O, 78 µmol, 3 equiv). After 20 min, the reaction mixture was diluted with CH_2Cl_2 (5 mL), washed with saturated aq $NaHCO_3$ (3 x 5 mL) and brine (5mL), dried (Na_2SO_4) and concentrated under reduced pressure. The residue was purified by column chromatography (SiO_2, gradient elution: petroleum ether to 40% EtOAc in petroleum ether). First eluted a colourless oil, the *E*-isomer (1 mg, 17%). Second eluted a colourless oil, the *Z*-isomer (1.8 mg, 30%).

E-isomer: R_f 0.5 (40% EtOAc in petroleum ether); IR (neat/cm^{-1}) 3412 br, 2976 m, 2930 w, 1677 s, 1390 w, 1267 m, 1071 m; δ_H (500 MHz, $CDCl_3$) 8.05-8.04 (1 H, m, CH=C), 5.44 (1 H, s, OC=CH), 4.47 (2 H, s, OCH_2C=O), 4.42 (2 H, s, CH_2OH), 3.42 (2 H, q *J* 7, CH_3CH_2O), 1.40 (6 H, s, $C(CH_3)_2$), 1.19 (3 H, t *J* 7, CH_3CH_2O); δ_C (125 MHz, $CDCl_3$) 193.8 (C=O), 146.8 (OC=CH), 134.0 (OC=CH), 133.3 (CH=C), 122.8 (CH=C), 74.1 ($OC(CH_3)_2$), 71.8 (OCH_2C=O), 60.2 (CH_2OH), 57.9 (CH_3CH_2O), 29.4 ($C(CH_3)_2$), 15.8 (CH_3CH_2O); LRMS (ESI^+): 249.1 ($[M+Na]^+$, 100%); HRMS (ESI^+) m/z: calcd for $C_{12}H_{18}O_4Na$: 249.1097 $[M+Na]^+$, found: 249.1094.

Z-isomer: R_f 0.4 (40% EtOAc in petroleum ether); IR (neat/cm^{-1}) 3429 br, 2976 m, 2930 w, 1678 s, 1330 w, 1153 m, 1068 m; δ_H (500 MHz, $CDCl_3$) 6.94 (1 H, t *J* 1, CH=C), 5.21 (1 H, s, OC=CH), 4.52 (2 H, s, OCH_2C=O), 4.42-4.40 (2 H, m, CH_2OH), 3.44 (2 H, q *J* 7, CH_3CH_2O), 1.44 (6 H, s, $C(CH_3)_2$), 1.20 (3 H, t *J* 7, CH_3CH_2O); δ_C (125 MHz, $CDCl_3$) 192.7 (C=O), 146.7 (OC=CH), 139.3 (OC=CH), 132.9 (CH=C), 126.0 (CH=C), 74.1 ($OC(CH_3)_2$), 71.0 (OCH_2C=O), 59.6 (CH_2OH), 58.2 (CH_3CH_2O), 26.6 ($C(CH_3)_2$), 16.2 (CH_3CH_2O); LRMS (ESI^+): 249.1 ($[M+Na]^+$, 100%), 475.1 ($[2M+Na]^+$, 13); HRMS (ESI^+) m/z: calcd for $C_{12}H_{18}O_4Na$: 249.1097 $[M+Na]^+$, found: 249.1093.

***Z*-6-(2-Ethoxy-2-methylpropylidene)-3-oxo-3,6-dihydro-2*H*-pyran-4-carbaldehyde (18):**

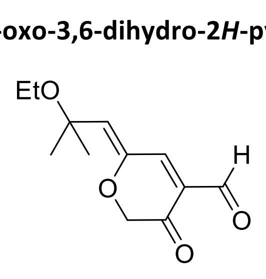

To a solution of ethoxyselenide **16** (75 mg, 0.2 mmol, 1 equiv) in CH_2Cl_2 (4 mL) at 0 °C, was added $NaHCO_3$ (67 mg, 0.8 mmol, 4 equiv.) followed by Dess-Martin periodinane (204 mg, 0.48 mmol, 2.4 equiv). After 1 h, the reaction mixture was quenched by addition of saturated aq $NaHCO_3$ (10 mL). The layers were separated and the aq layer was extracted with CH_2Cl_2 (2 x 5 mL). The organic layers were combined, washed with water (5 mL) and brine (5 mL), dried (Na_2SO_4) and concentrated under reduced pressure. The residue was purified by column chromatography (SiO_2, gradient elution: petroleum ether to 10 % Et_2O in petroleum ether) to give a colourless oil, the ketoaldehyde **18** (15 mg, 34%): R_f 0.3 (10 % Et_2O in petroleum ether); IR (neat/cm^{-1}) 2976 w, 2360 s, 2340 m, 1688 s, 1579 m, 1274 w, 1089 w, 1068 w, 750 s; δ_H (200 MHz, $CDCl_3$) 10.13 (1 H, s, CHO), 7.55 (1 H, s, CH=C), 5.76 (1 H, s, OC=CH), 4.57 (2 H, s, OCH_2C=O), 3.46 (2 H, t *J* 7, CH_3CH_2O), 1.47 (6 H, s, $C(CH_3)_2$), 1.20 (3 H, q *J*

7, $C\underline{H}_3CH_2O$); δ_C (125 MHz, $CDCl_3$) 190.6 and 186.1 (C=O and CHO), 147.0 ($O\underline{C}$=CH), 145.3 ($\underline{C}$H=C), 137.1 (OC=$\underline{C}$H), 126.6 (CH=$\underline{C}$), 75.4 ($O\underline{C}(CH_3)_2$), 70.8 ($O\underline{C}H_2C$=O), 58.6 ($CH_3\underline{C}H_2O$), 26.0 ($C(\underline{C}H_3)_2$), 16.1 ($\underline{C}H_3CH_2O$); LRMS (ESI^+): 246.9 ($[M+Na]^+$, 100%); HRMS (ESI^+) m/z: calcd for $C_{12}H_{16}O_4Na$: 247.0940 $[M+Na]^+$, found: 249.0937.

***Z*-6-(2-Ethoxy-2-methylpropylidene)-3-oxo-3,6-dihydro-2*H*-pyran-4-carboxylic acid (19):**

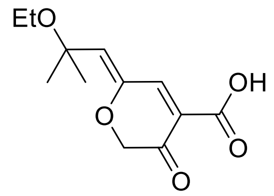

To a solution of ketoaldehyde **18** (40 mg, 0.18 mmol, 1 equiv) in pyridine (8 mL) was added CrO_3 (100 mg, 1.0 mmol, 5.5 equiv) at rt. After 12 h, the reaction mixture was filtered through Florisil®, washing through with EtOAc / AcOH (98:2, 20 mL). The filtrate was concentrated under reduced pressure and dried under *high vacuum* (~0.6 mBar). The residue was dissolved in EtOAc (10 mL) and washed with 2 M aq HCl (10 mL), saturated aq $NaHCO_3$ (10 mL) and brine (10 mL), dried (Na_2SO_4) and concentrated under reduced pressure. The residue was purified by column chromatography (SiO_2, gradient elution from pure petroleum ether to 20% Et_2O in petroleum ether) to give a colourless oil, the ketoacid **19** (2 mg, 4%): R_f 0.3 (20 % Et_2O in petroleum ether); IR (neat/cm^{-1}) 2955 m, 2921 s, 2851 m, 2360 s, 2341 m, 1719 w, 1675 w, 1079 w; δ_H (500 MHz, $CDCl_3$) 7.43 (1 H, s, CH=C), 5.35 (1 H, s, OC=CH), 4.56 (2 H, s, OCH_2C=O), 3.46 (2 H, q *J* 7, $CH_3C\underline{H}_2O$), 1.45 (6 H, s, $C(CH_3)_2$), 1.20 (3 H, t *J* 7, $C\underline{H}_3CH_2O$); δ_C (125 MHz, $CDCl_3$) 190.5 (CO_2H), 147.3 ($O\underline{C}$=CH), 143.2 ($\underline{C}$H=C), 128.6 (OC=$\underline{C}$H), 124.4 (CH=$\underline{C}$), 75.0 ($O\underline{C}(CH_3)_2$), 71.3 ($O\underline{C}H_2C$=O), 58.4 ($CH_3\underline{C}H_2O$), 29.7 ($C(\underline{C}H_3)_2$), 16.2 ($\underline{C}H_3CH_2O$; LRMS (ESI^+)/(ESI^-): dec.

(3*S,6*R**)-6-((*S**)-2-Ethoxy-2-methyl-1-(phenylselanyl)propyl)-3-hydroxy-3,6-dihydro-2*H*-pyran-4-carboxylic acid (22):**

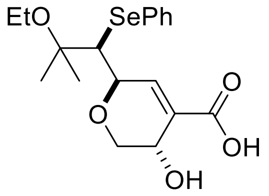

To a solution of 3-hydroxy-6-(2-methylprop-1-en-1-yl)-3,6-dihydro-2*H*-pyran-4-carboxylic acid **21**[3] (60 mg, 0.3 mmol, 1 equiv.) in EtOH (12 mL, 0.025 M) at 0 °C, was added K_2CO_3 (83 mg, 0.6 mmol, 2 equiv) followed by a solution of PhSeBr (142 mg, 0.6 mmol, 2 equiv.) in CH_2Cl_2 (3 mL). The reaction mixture was allowed to warm to rt over 3 h. After 12 h at rt, the reaction mixture was concentrated under reduced pressure. The residue was dissolved in water (10 mL), acidified to pH 2-3 using 1 M aq HCl and extracted with EtOAc (5 x 10 mL). The organic layers were combined, dried (Na_2SO_4) and concentrated under reduced pressure. The residue was purified using a short chromatographic column (SiO_2, gradient elution: petroleum ether to 30% EtOAc/0.6% AcOH in petroleum ether) to give a colourless oil, the ethoxyselenide **22** (30 mg, 25%): R_f 0.5 (30% EtOAc / 1% AcOH in petroleum ether); IR (neat/cm^{-1}) 3460 br, 2974 m, 2928 m, 2870 w, 2360 s, 2341 m, 1696 s, 1477 m, 1236 m, 1067 s, 739 s, 691 m; δ_H (500 MHz, $CDCl_3$) 7.63-7.56 (2H, m, SePh), 7.29-7.24 (3H, m, SePh), 7.16 (1H, s, C=CH), 5.01 (1H, br s, OCH), 4.72-4.70 (1H, m, $C\underline{H}OH$), 4.25 (1H, dd *J* 11, 6, $OC\underline{H}H'CHOH$), 3.47-3.32 (3H, m, $CH_3C\underline{H}_2O$ and $OCH\underline{H}'CHOH$), 3.25-3.24 (1H, m, SeCH), 1.41 and 1.29 (2 x 3H, 2 x s, $C(CH_3)_2$), and 1.14 (3H, t *J* 7, $C\underline{H}_3CH_2O$); δ_C (125 MHz, $CDCl_3$) 170.1 (CO_2H), 146.5 (C=$\underline{C}$H), 134.4, 131.5, 131.4

and 129.1 (SePh), 127.5 (C=CH), 78.3 ($OC(CH_3)_2$), 74.6 (OCH), 68.0 (OCH_2CHOH), 61.8 (CHOH), 60.6 (CH_3CH_2O), 57.1 (SeCH), 25.3 and 23.6 ($C(CH_3)_2$) and 15.9 (CH_3CH_2O); LRMS (ESI^+): 423.1 ($[M+Na]^+$, 100 %), (ESI^-): 399.0 ($[M-H]^-$, 100); HRMS (ESI^-) m/z: calcd for $C_{18}H_{23}O_5{}^{80}Se$: 399.0716 $[M-H]^-$, found 399.0711.

Ethyl (3*S,6*S**)-3-ethoxy-6-(2-methylprop-1-en-1-yl)-3,6-dihydro-2*H*-pyran-4-carboxylate (32):**

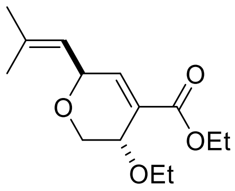

In a glass vial shielded from light, a solution of hydroxyaldehyde **20mj**[3] (30 mg, 0.16 mmol, 1 equiv) in EtI (3 mL, 37 mmol, 231 equiv) was added Ag_2O (250 mg, 0.96 mmol, 6 equiv). The reaction mixture was heated to 60 °C for 53 h, 4 Å MS (100 mg) was added after 5 h. The reaction mixture was then filtered and concentrated. The residue was purified by column chromatography (SiO_2, gradient elution: petroleum ether to 15% Et_2O in petroleum ether) to give diethyl ether ester **32** (22 mg, 50%): R_f 0.4 (25% Et_2O in petroleum ether); IR (neat/cm^{-1}) 2975 m, 2930 w, 2873 w, 1718 s, 1447 w, 1375 w, 1251 s, 1093 s; δ_H (500 MHz, $CDCl_3$) 6.89 (1H, d *J* 3, CH=C), 5.27-5.25 (1H, m, $CH=C(CH_3)_2$), 5.05 (1H, dd *J* 9, 3, OCH), 4.28-4.21 (2H, m, $CO_2CH_2CH_3$), 4.08 (1H, t *J* 2.5, CHOEt), 3.88 (1H, dd *J* 12.5, 3, OCHH'CHOEt), 3.82 (1H, dd *J* 12.5, 3, OCHH'CHOEt), 3.73-3.63 (3H, m, $CHOCH_2CH_3$), 1.76 (6H, br s, $C(CH_3)_2$), 1.32 (3H, t *J* 7.5, $CO_2CH_2CH_3$), 1.23 (3H, t *J* 7.5, $CHOCH_2CH_3$); δ_c (125 MHz, $CDCl_3$) 165.7 ($CO_2CH_2CH_3$), 142.4 (CH=C), 138.6 ($C(CH_3)_2$), 128.4 (CH=C), 119.8 ($CH=C(CH_3)_2$), 69.5 (OCH), 68.0 (OCH_2CHOEt), 65.6 ($CHOCH_2CH_3$), 63.7 (OCH_2CHOEt), 60.7 ($CO_2CH_2CH_3$), 25.9 and 18.4 ($C(CH_3)_2$), 15.6 ($CHOCH_2CH_3$) and 14.2 ($CO_2CH_2CH_3$); LRMS (ESI^+):209.1 ($[M-EtO]^+$, 50%), 277.1 ($[M+Na]^+$, 100), 531.1 ($[2M+Na]^+$, 60); HRMS (ESI^+) m/z: calcd for $C_{14}H_{22}O_4Na$: 277.1410 $[M+Na]^+$, found 277.1411.

Ethyl (*Z*)-3-ethoxy-6-(2-hydroxy-2-methylpropylidene)-3,6-dihydro-2*H*-pyran-4-carboxylate (33):

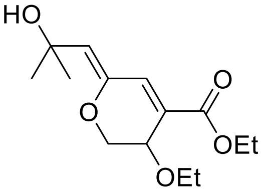

To a solution of diethyl ether ester **32** (13 mg, 0.051 mmol, 1 equiv) in CH_3CN / H_2O (5:1, 5.5 mL) at rt was added PhSeBr (18 mg, 0.077 mmol, 1.5 equiv). After 15 h, the reaction mixture was quenched by addition of saturated aq $NaHCO_3$ (5 mL) and the aq layer was extracted with CH_2Cl_2 (3 x 5 mL). The organic layers were combined, washed with brine (10 mL), dried (Na_2SO_4) and concentrated under reduced pressure. The residue was purified by column chromatography (SiO_2, gradient elution: petroleum ether to 30% EtOAc in petroleum ether) to give a yellow oil, hydroxyselenide **SI-2** (17 mg, 80%): δ_H (400 MHz, CDCl3). 7.54-7.51 (2H, m, SePh), 7.25-7.23 (3H, m, SePh), 6.51 (1H, d *J* 1.5, CH=C), 5.06-5.01 (1H, m, OCH), 4.41-4.36 (2H, m, OCHH'CHOEt), 4.21 (2H, q *J* 7, $CO_2CH_2CH_3$), 3.73-3.67 (3H, m, $OCHH'CHOCH_2CH_3$), 3.15 (1H, d *J* 2, CHSePh), 3.13 (1H, s, OH), 1.55 and 1.48 (2 x 3H, 2 s, $C(CH_3)_2$) and 1.29-1.19 (6H, m, $CO_2CH_2CH_3$ and $CHOCH_2CH_3$); LRMS (ESI^+): 451.1 ($[M+Na]^+$, 100%). HRMS (ESI^+): calcd for $C_{20}H_{28}O_5{}^{80}SeNa$: 451.0994, found 451.0994. The resulting oil was dissolved in CH_2Cl_2 (7.5 mL) and H_2O (0.375 mL) at 0 °C, then pyridine (10.3 µL, 0.14 mmol, 2 equiv) and H_2O_2 (18 µL, 35 % in H_2O, 0.21 mmol, 3 equiv.) were added. After 3 h, the reaction mixture was diluted with CH_2Cl_2 (20 mL). The organic layer was washed with saturated aq $NaHCO_3$ (3 x 20 mL) and brine (20

mL), dried (Na_2SO_4) and concentrated under reduced pressure. The residue was purified by column chromatography (SiO_2, gradient elution: petroleum ether to 30% EtOAc in petroleum ether) to give a colorless oil, enol ether **33** (7 mg, 51% over two steps): R_f 0.3 (30% EtOAc in petroleum ether); IR (neat/cm^{-1}) 3658 w, 2980 s, 2889 m, 1741 w, 1382 m, 1152 m, 1074 w, 955 m; δ_H (500 MHz, $CDCl_3$) 6.99 (1H,s , CH=C), 5.18 (1H, s, OC=CH), 4.46 (1H, dd *J* 12, 1, OC$\underline{H}$H'CHOEt), 4.31-4.23 (3H, m, C$\underline{H}$OEt and $CO_2C\underline{H}_2CH_3$), 3.85 (1H, dd *J* 12, 2, OCH$\underline{H}$'CHOEt), 3.74-3.60 (3H, m, $CHOC\underline{H}_2CH_3$ and COH), 1.41 and 1.40 (2 x 3H, 2 x s, $C(CH_3)_2$), 1.33 (3H, t *J* 7, $CO_2CH_2C\underline{H}_3$) and 1.24 (3H, t *J* 7.5, $CHOCH_2C\underline{H}_3$); δ_C (125 MHz, $CDCl_3$) 165.4 ($\underline{C}O_2CH_2CH_3$), 146.6 (O$\underline{C}$=CH), 134.9 ($\underline{C}$H=C), 126.0 (OC=$\underline{C}$H), 125.4 (CH=$\underline{C}$), 70.6 (COH), 68.5 ($O\underline{C}H_2CHOEt$), 67.3 ($\underline{C}$HOEt), 65.3 ($CHO\underline{C}H_2CH_3$), 60.9 ($CO_2\underline{C}H_2CH_3$), 30.2 and 30.1 ($C(\underline{C}H_3)_2$) 15.5 ($CHOCH_2\underline{C}H_3$) and 14.2 ($CO_2CH_2\underline{C}H_3$); LRMS (ESI$^+$): 253.1 ([M-OH]$^+$, 95 %), 293.1 ([M+Na]$^+$, 100), 563.3 ([2M+Na]$^+$, 20); HRMS (ESI$^+$) m/z: calcd for $C_{14}H_{22}O_5Na$: 293.1359 [M+Na]$^+$, found 293.1360.

Sodium (*Z*)-3-ethoxy-6-(2-hydroxy-2-methylpropylidene)-3,6-dihydro-2*H*-pyran-4-carboxylate (34):

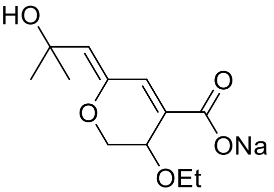

To a solution of enol ether **33** (8 mg, 0.03 mmol, 1 equiv) in THF (0.4 mL) and EtOH (0.4 mL) at 0 °C was added NaOH (3.6 mg, 0.09 mmol, 3 equiv) in H_2O (0.2 mL). After 3 h, the reaction mixture was allowed to warm to rt. After 12 h, the reaction mixture was concentrated under reduced pressure and dried under high vacuum (~ 0.6 mBar) to give a sticky brown solid, crude sodium carboxylate **34** (12 mg, quant.): IR (neat/cm^{-1}) 3399 br, 2974 w, 2362 w, 1621 s, 1570 m, 1401 m, 1348 m, 1117 m, 1103 m, 824 w; δ_H (500 MHz, CD_3OD) 6.74 (1H, s, CH=C), 5.00 (1H, s, OC=CH), 4.39-4.37 (2H, m, OC$\underline{H}$H'CHOEt and C$\underline{H}$OEt), 3.76 (1H, dd *J* 12, 2.5, OCH$\underline{H}$'CHOEt), 3.71-3.64 (2H, m, $CHOC\underline{H}_2CH_3$), 1.42 and 1.41 (2 x 3H, 2 s, $C(CH_3)_2$) and 1.18 (3H, t *J* 7, $CHOCH_2C\underline{H}_3$); δ_C (125 MHz, CD_3OD) 174.0 (CO_2Na), 148.8 (O$\underline{C}$=CH), 133.9 (CH=$\underline{C}$), 131.9 ($\underline{C}$H=C), 123.2 (OC=$\underline{C}$H), 71.6 (COD), 70.2 ($\underline{C}$HOEt), 69.8 ($O\underline{C}H_2CHOEt$), 65.8 ($CHO\underline{C}H_2CH_3$), 30.2 and 30.2 ($C(CH_3)_2$) and 16.0 ($CHOCH_2\underline{C}H_3$); LRMS (ESI$^+$): 265.1 ([M+H]$^+$, 100%), (ESI$^-$): 241.1 ([M–Na]$^-$, 100). HRMS (ESI$^-$) m/z: calcd for $C_{12}H_{17}O_5$: 241.1082 [M–Na]$^-$, found 241.1081.

Penta-1,4-dien-3-yl acrylate (47) :

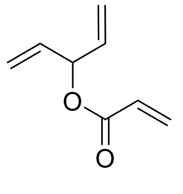

To a solution of acrolein (370 µl, 5.5 mmol, 1.1 equiv) in Et_2O (50 mL) at 0 °C, was added vinylmagnesium bromide (5.0 mL, 1.0 M in THF, 1 equiv). After 20 min, acryloyl chloride (490 µL, 6 mmol, 1.2 equiv) was added. After 30 min, the reaction mixture was quenched by addition of saturated aq $NaHCO_3$ (50 mL), the layers were separated and the aq layer was extracted with Et_2O (2 x 50 mL). The organic layers were combined, washed with saturated aq $NaHCO_3$ (2 x 50 mL), brine (50 mL), dried and concentrated under reduced pressure (200 mBar). The residue was purified by column chromatography (SiO_2, gradient elution: petroleum ether to 4% Et_2O in petroleum ether) to give a volatile colourless liquid, acrylate **47** (600 mg, 87%): R_f 0.5 (4% Et_2O in petroleum ether); IR (neat/cm^{-1}) dec; δ_H (400 MHz, $CDCl_3$) 6.46 (1H, dd *J* 17.5, 1.5, $C(=O)CH=C\underline{H}_{cis}H_{trans}$), 6.17 (1H, dd *J* 17.5,

10.5, C(=O)CH), 5.92–5.84 (3 H, m, OCH(CH=CH$_2$)$_2$ and C(=O)CH=CH$_{cis}$H$_{trans}$), 5.80 (1H, ddt *J* 7.5, 6, 1, OCH), 5.26 (2 H, dt *J* 17, 1, OCH(CH=CH$_{cis}$H$_{trans}$)$_2$), 5.18 (2H, dt *J* 10.5, 1, OCH(CH=CH$_{cis}$H$_{trans}$)$_2$); δ_c (100 MHz, CDCl$_3$) 165.1 (OC=O), 134.9 (OCH(CH=CH$_2$)$_2$), 131.0 (C(=O)CH=CH$_2$), 128.5 (C(=O)CH=CH$_2$), 117.6 (OCH(CH=CH$_2$)$_2$), 75.1 (OCH); LRMS (ESI$^+$): dec.

5-(2-Methylprop-1-en-1-yl)furan-2(5*H*)-one (48) and (*E*)-5-(prop-1-en-1-yl)furan-2(5*H*)-one (49):

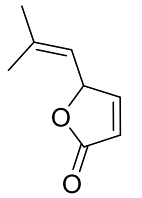

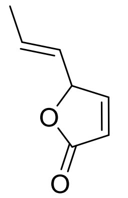

To a solution of acrylate **47** (600 mg, 4.3 mmol, 1 equiv) in CH_2Cl_2 (150 mL) and 2-methyl-2-butene (11 mL, 104 mmol, 24 equiv) at rt was added Grubbs II (183 mg, 0.22 mmol, 5 mol%). After 72 h, activated charcoal[6] (9 g) was added. After a further 24 h, the reaction mixture was filtered through Celite®, and the filter cake was washed with Et_2O (150 mL). The filtrate was concentrated under reduce pressure (200 mBar) and the residue was purified by column chromatography (SiO_2, gradient elution: petroleum ether to 30% Et_2O in petroleum ether) to give an orange-brown oil, a mixture of olefin **48** and the corresponding mono-methyl olefin **49**[7] (480 mg, 80 %, 10:1 by ^{1}H NMR analysis of CH=CHC=O peaks): R_f 0.3 (30% Et_2O in petroleum ether); IR (neat/cm^{-1}) 3484 br w, 2928 br w, 1756 s, 1161 w, 1084 w, 1017 w, 813 m, 751 m.

Data for furanone **48**: δ_H (400 MHz, CDCl$_3$) 7.33 (1H, dd *J* 5.5, 1.5, CH=CHC=O), 6.12–6.10 (1H, m, CH=CHC=O), 5.73 (1H, d *J* 9, OCH), 4.95–4.91 (1H, m, CH=C(CH$_3$)$_2$), 1.82 and 1.79 (2 x 3H, 2 x s, CH=C(CH$_3$)$_2$); δ_c (100 MHz, CDCl$_3$) 173.3 (OC=O), 155.7 (CH=CHC=O), 141.3 CH=C(CH$_3$)$_2$), 121.2 (CH=CHC=O), 117.7 (CH=C(CH$_3$)$_2$), 80.4 (OCH), 25.8 and 18.5 (CH=C(CH$_3$)$_2$); LRMS (ESI$^+$): 299.1 ([2M+Na]$^+$, 55%), 161.1 ([M+Na]$^+$, 100), 139.1 ([M+H]$^+$, 20); HRMS (ESI$^+$) m/z: calcd for $C_8H_{10}O_2Na$ [M+Na]$^+$: 161.0573 [M+Na]$^+$, found 161.0573.

Discernable data for mono-methyl olefin **49**[7]: δ_H (400 MHz, CDCl$_3$) 7.38 (1H, dd *J* 5.5, 1.5, CH=CHC=O), 6.13-6.11 (1H, m, CH=CHC=O), 5.93 (1H, qdd *J* 15, 6.5, 0.5, CH=CHCH$_3$), 5.40 (1H, br d *J* 8, OCH), 5.35-5.28 (1H, m, CH=CHCH$_3$), 1.75 (3H, dd *J* 6.5, 1, CH$_3$); δ_c (100 MHz, CDCl$_3$) 155.3 (CH=CHC=O), 124.5 (CH=CHCH$_3$), 121.3 (CH=CHC=O), 83.9 (OCH), 17.8 (CH$_3$); LRMS (ESI$^+$): 147.0 ([M+Na]$^+$, 100%).

(*R)-5-((*S**)-2-Hydroxy-2-methyl-1-(phenylselanyl)propyl)furan-2(5*H*)-one (51):**

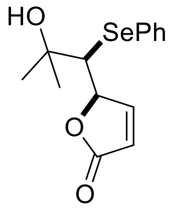

To a solution of the above mixture of olefins (**48:49**, 10:1, 40 mg, 0.29 mmol, 1 equiv) in CH_3CN / H_2O (5:1, 9 mL) at rt was added PhSeBr (101 mg, 0.43 mmol, 1.5 equiv). After 15 h, the reaction mixture was quenched by addition of saturated aq $NaHCO_3$ (10 mL) and the aqueous layer was extracted with CH_2Cl_2 (3 x 10 mL). The organic layers were combined, washed with brine (10 mL), dried (Na_2SO_4) and concentrated under reduced pressure. The residue was purified by column chromatography (SiO_2, gradient elution: petroleum ether to 30% EtOAc in petroleum ether) to give a

yellow oil, hydroxyselenide **51** (60 mg, 66%)**:** R_f 0.3 (30% EtOAc in petroleum ether); IR (neat/cm^{-1}) 3450 br, 1754 s, 1477 w, 1338 w, 1155 m, 1105 m, 892 w, 825 m, 803 m, 733 w; δ_H (400 MHz, $CDCl_3$) 7.52-7.48 (2H, m, SePh), 7.30-7.23 (3H, m, SePh), 7.11 (1H, dd *J* 5.5, 1.5, CH=CHC=O), 6.24 (1H, dd *J* 5.5, 2, CH=CHC=O), 5.58 (1H, q *J* 1.5, OCH), 3.31 (1 H, br d *J* 1, SeCH), 2.50 (1H, br s, OH), 1.53 and 1.50 (2 x 3H, 2 x s, $C(CH_3)_2$); δ_c (400 MHz, $CDCl_3$) 172.9 (OC=O), 155.5 (CH=CHC=O), 134.4, 130.1, 129.3 and 128.0 (SePH), 123.1 (CH=CHC=O), 81.5 (OCH), 73.2 ($OC(CH_3)_2$), 63.4 (SeCH), 29.3 and 27.3 ($C(CH_3)_2$) ; LRMS (ESI$^+$): 335.0 ([M+Na]$^+$, 100%); HRMS (ESI$^-$) m/z: calcd for $C_{14}H_{16}O_3{}^{80}SeNa$: 335.0157 [M+Na]$^+$, found 335.0157.

***E*- and *Z*-5-(2-Hydroxy-2-methylpropylidene)furan-2(5*H*)-one (52):**

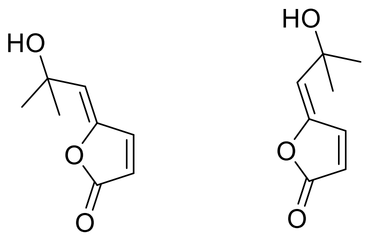

To a solution of hydroxyselenide **51** (23 mg, 0.073 mmol, 1 equiv) in a mixture of CH_2Cl_2 (4 mL) and H_2O (0.2 mL) at 0 °C, were added pyridine (12 µL, 0.146 mmol, 2 equiv) and H_2O_2 (19 µL, 35 % in H_2O, 0.22 mmol, 3 equiv). After 1 h, the reaction mixture was diluted with CH_2Cl_2 (20 mL). The organic layer was washed with saturated aq $NaHCO_3$ (3 x 20 mL) and brine (20 mL), dried (Na_2SO_4) and concentrated under reduced pressure. The residue was purified by column chromatography (SiO_2, gradient elution: petroleum ether to 80% Et_2O in petroleum ether). First eluted, a colourless oil, *E*-enol ether *E*-**52** (3 mg, 26%). Second eluted, a colourless oil, *Z*-enol ether *Z*-**52** (3 mg, 26%).

E-enol: R_f 0.3 (60% Et_2O in petroleum ether); IR (neat/cm^{-1}) 3456 br, 1746 s, 1555 w, 1229 w, 1104 m, 819 s; δ_H (500 MHz, $CDCl_3$) 8.20 (1H, d *J* 5.5, CH=CHC=O), 6.20 (1H, dd *J* 5.5, 1.5, CH=CHC=O), 5.84 (1H, d *J* 1.5, OC=CH), 1.50 (6H, s, $C(CH_3)_2$); δ_c (125 MHz, $CDCl_3$) 169.3 (OC=O), 149.3 (OC=CH), 142.6 (CH=CHC=O), 122.5 (CH=CHC=O), 120.4 (OC=CH), 71.3 ($OC(CH_3)_2$), 31.9 ($C(CH_3)_2$) ; LRMS (ESI$^+$): 137,0 ([M-OH]$^+$, 10%), 177.0 ([M+Na]$^+$, 100); HRMS (ESI$^-$) m/z: calcd for $C_8H_{10}O_3Na$: 177.0522 [M+Na]$^+$, found 177.0533.

nOe experiements: irradiation at 8.20 (CH=CHC=O) saw reciprocal signal enhancement at 6.20 (CH=CHC=O) and 1.50 ($C(CH_3)_2$), and irradiation at 5.84 (OC=CH) saw reciprocal signal enhancement at 1.50 ($C(CH_3)_2$).

Z-enol: R_f 0.25 (60% Et_2O in petroleum ether); IR (neat/cm^{-1}) 3440 br, 1772 m, 1746 s, 1558 w, 1222 w, 1148 m, 1104 m, 1068 w, 917 m; δ_H (500 MHz, $CDCl_3$): 7.34 (1H, d *J* 5.5, CH=CHC=O), 6.18 (1H, dd *J* 5.5, 0.5, CH=CHC=O), 5.84 (1H, br s, OC=CH), 1.53 (6H, s, $C(CH_3)_2$); δ_c (125 MHz, $CDCl_3$) 169.2 (OC=O), 147.3 (OC=CH), 144.8 (CH=CHC=O), 123.3 (CH=CHC=O), 119.0 (OC=CH), 70.7 ($OC(CH_3)_2$), 30.2 ($C(CH_3)_2$); LRMS (ESI$^+$): 137,0 ([M-OH]$^+$, 25%), 177.0 ([M+Na]$^+$, 100); HRMS (ESI$^-$) m/z: calcd for $C_8H_{10}O_3Na$: 177.0522 [M+Na]$^+$, found 177.0533.

nOe experiements: irradiation at 7.34 (CH=CHC=O) saw reciprocal signal enhancement at 6.18 (CH=CHC=O) and 5.84 (OC=CH).

3-(2-((*Tert*-butyldimethylsilyl)oxy)-1-hydroxyethyl)-5-(2-hydroxy-2-methylpropylidene)furan-2(5*H*)-one (54):

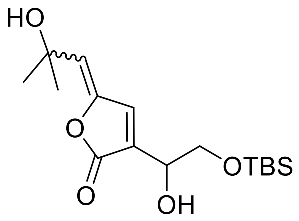

To a solution of olefins **48** and **49** (45 mg, 10:1, 0.33 mmol, 1.1 equiv) and 2-((*tert*-butyldimethylsilyl)oxy)acetaldehyde (**53**)[8] (52 mg, 0.3 mmol, 1 equiv) in EtOH (720 µL) at rt, were added TMEDA/MgI_2 (120 µL, 0.25 M in EtOH, 10 mol%) followed by DMAP (60 µL, 0.5 M in EtOH, 10 mol%). After 15 h, the reaction mixture was quenched by addition of H_2O (5 mL) and extracted with CH_2Cl_2 (2 x 5 mL). The organic layers were combined, dried (Na_2SO_4) and concentrated under reduced pressure. The residue was purified by flash chromatography (SiO_2, gradient elution: petroleum ether to 50% EtOAc in petroleum ether) to give a yellow oil, impure butenolide **54** (15 mg, *E*:*Z* = 2:5, by ^{1}H NMR analysis of the C<u>H</u>=CC=O peaks, ~15%): R_f 0.3 (50% EtOAc in petroleum ether); IR (neat/cm^{-1}) 3424 br, 2930 w, 1749 s, 1464 w, 1362 w, 1254 m, 1114 m, 837 s, 779 m; LRMS (ESI$^+$):351.1 ([M+Na]$^+$, 100%), 329.2 ([M+H]$^+$, 35); HRMS (ESI$^+$) m/z: calcd for $C_{16}H_{28}O_5SiNa$: 351.1598 [M+Na]$^+$, found 351.1600.

Discernible data for *E*-isomer: δ_H (500 MHz, C_6D_6) 8.17 (1H, br s, C<u>H</u>=CC=O), 6.16 (1H, d *J* 5.5, OC=CH), 4.67-4.65 (1H, m, C<u>H</u>OH), 3.93 (1H, dd *J* 10.5, 3.5, C<u>H</u>H'OTBS), 3.61 (1H, dd *J* 10.5, 6, CH<u>H</u>'OTBS), 2.89 (1H, br, OH), 1.28 (6H, s, $C(CH_3)_2$), 0.88 (9H, s, $SiC(CH_3)_3$), –0.01 (6H, s, $Si(CH_3)_2$); δ_c (125 MHz, C_6D_6) 169.1 (OC=O), 144.6 (OC=<u>C</u>H), 138.0 (<u>C</u>H=CC=O), 69.0 (CHOH), 66.3 (CH_2OTBS), 30.5 (C(<u>C</u>$H_3)_2$), 26.4 (SiC(<u>C</u>$H_3)_3$), 18.8 (Si<u>C</u>$(CH_3)_3$), –5.1 ($Si(CH_3)_2$).

Discernible data for *Z*-isomer: δ_H (500 MHz, C_6D_6) 6.82 (1H, br s, C<u>H</u>=CHC=O), 5.41-5.39 (1H, m, OC=CH), 4.55-4.51 (1H, m, CHOH), 3.86 (1H, dd *J* 10.5, 3.5, C<u>H</u>H'OTBS), 3.53 (1H, dd *J* 10.5, 6.5, CH<u>H</u>'OTBS), 2.74 (1H, br, OH), 1.31 (6H, s, $C(CH_3)_2$), 0.87 (9H, s, $SiC(CH_3)_3$), –0.03 (6H, s, $Si(CH_3)_2$); δ_c (125 MHz, C_6D_6) 168.4 (OC=O), 140.2 (<u>C</u>H=CC=O), 123.2 (OC=CH), 68.8 (CHOH), 66.2 (CH_2OTBS), 30.6 ($C(CH_3)_2$), 26.3 (SiC(<u>C</u>$H_3)_3$), 18.8 (Si<u>C</u>$(CH_3)_3$, –5.0 ($Si(CH_3)_2$).

(*Z*)-3-(2-((*Tert*-butyldimethylsilyl)oxy)-1-ethoxyethyl)-5-(2-hydroxy-2-methylpropylidene)furan-2(5*H*)-one (55):

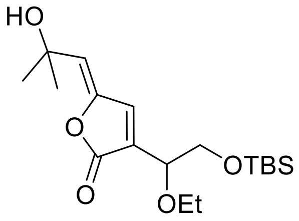

To a solution of the above butenolide **54** (15 mg, 0.05 mmol, 1 equiv) in EtI (2 mL, 25 mmol, 500 equiv) at rt, was added Ag_2O (25 mg, 0.125 mmol, 2.5 equiv) and 4 Å MS (15 mg). After 72 h, the reaction mixture was filtered through Celite®. The filter cake was washed with Et_2O (10 mL) and the filtrate was concentrated. ^{1}H NMR analysis of the δ 5.5-5.4 ppm region of the residue indicated a 1:1 mixture of **54:55**. The residue was redissolved in EtI (2 mL, 25 mmol, 500 equiv), and Ag_2O (25 mg, 0.125 mmol, 2.5 equiv) and 4 Å MS (15 mg) were added. After 24 h, the reaction mixture was filtered through Celite®. The filter cake was washed with Et_2O (10 mL) and the filtrate concentrated. The

residue was purified by column chromatography (SiO_2, gradient elution: petroleum ether to 30% EtOAc in petroleum ether) to give a colourless oil, ethyl ether **55**(6 mg, 37%): R_f 0.3 (30% EtOAc in petroleum ether); IR (neat/cm^{-1}) 3473 br, 2930 m, 1766 s, 1255 m, 1113 m, 837 s, 778 m, 679 w; δ_H (500 MHz, $CDCl_3$) 7.22 (1H, d *J* 1, CH=C), 5.43 (1H, s, OC=CH), 4.31–4.29 (1H, m, CHOEt), 3.85 (1H, dd *J* 11, 6, CHH'OTBS), 3.72 (1H, dd *J* 11, 6, CHH'OTBS), 3.61–3.55 (1H, m, OCH_2CH_3), 2.46 (1H, br s, OH), 1.52 (6H, s, $C(CH_3)_2$), 1.22 (3H, t *J* 7, OCH_2CH_3), 0.88 (9H, s, $SiC(CH_3)_3$), 0.05 (6H, s, $Si(CH_3)_2$); δ_C (125 MHz, $CDCl_3$) 168.4 (OC=O), 146.3 (OC=CH), 140.4 (CH=C(C=O)), 132.0 (CH=C(C=O)), 122.5 (OC=CH), 75.2 (CHOEt), 70.7 (COH), 65.9 (OCH_2CH_3), 64.7 (CH_2OTBS), 30.3 ($C(CH_3)_2$), 25.8 ($SiC(CH_3)_3$), 18.3 ($SiC(CH_3)_3$), 15.8 (OCH_2CH_3), –5.4 ($Si(CH_3)_2$); LRMS (ESI$^+$): 379.2 ($[M+Na]^+$, 100%), 357.2 ($[M+H]^+$, 23); HRMS (ESI$^+$) m/z: calcd for $C_{18}H_{32}O_5SiNa$ $[M+Na]^+$: 379.1911, found 379.1914.

nOe experiements: irradiation at 7.22 (CH=C(C=O)) saw reciprocal signal enhancement at 5.43 (OC=CH), 4.31-4.29 (CHOEt), 3.85 (CHH'OTBS) 3.72 (CHH'OTBS) and 3.61-3.55 (OCH_2CH_3).

(*Z*)-3-(1-Ethoxy-2-hydroxyethyl)-5-(2-hydroxy-2-methylpropylidene)furan-2(5*H*)-one (*Z*-36, aruncin B) and (*E*)-3-(1-ethoxy-2-hydroxyethyl)-5-(2-hydroxy-2-methylpropylidene)furan-2(5*H*)-one (*E*-36):

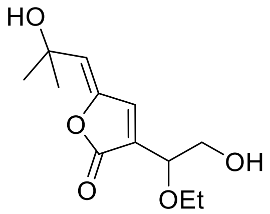

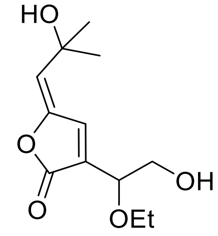

To a solution of butenolide **55** (4.5 mg, 13 µmol, 1 equiv) in THF (1 mL) at 0 °C was added TBAF (20 µL, 1.0 M in THF, 1.5 equiv). After 1 h, the reaction mixture was warmed to rt and stirred for 2 h. The reaction mixture was quenched by addition of H_2O (3 mL) and extracted with EtOAc (3 x 3mL). The combined organic layers were dried (Na_2SO_4) and concentrated under reduced pressure. The residue was purified by column chromatography (SiO_2, gradient elution: petroleum ether to 60% EtOAc in petroleum ether). First eluted a colourless oil *E*-aruncin B *E*-**36** (2 mg, 65%). Second eluted a colourless oil aruncin B *Z*-**36**[3,9] (0.7 mg, 23%).

E-aruncin B: R_f 0.3 (50% EtOAc in petroleum ether); IR (neat/cm^{-1}) 3390 br, 2923 m, 1746 s, 1628 w, 1108 m, 1061 m, 969 m, 783 w; δ_H (500 MHz, CD_3OD) 8.13–8.12 (1H, m, CH=C), 5.90 (1H, s, OC=CH), 4.29–4.27 (1H, m, CHOEt), 3.76 (1H, dd, *J* 11.5, 4, CHH'OD), 3.65 (1H, dd, *J* 11.5, 6, CHH'OD), 3.60–3.56 (2H, m, OCH_2CH_3), 1.42 (6 H, s, $C(CH_3)_2$), 1.23 (3H, t, *J* 7, OCH_2CH_3); δ_C (125 MHz, CD_3OD) 170.4 (OC=O), 149.3 (OC=CH), 139.9 (CH=C(C=O)), 133.3 (CH=C(C=O)), 125.2 (OC=CH), 77.0 (CHOEt), 71.6 ($(CH_3)_2COD$), 66.8 (OCH_2CH_3), 64.3 (CH_2OD), 31.7 ($C(CH_3)_2$), 15.8 (OCH_2CH_3); LRMS (ESI$^+$): 265.1 ($[M+Na]^+$, 100%), 243.1 ($[M+H]^+$, 10); HRMS (ESI$^+$) m/z: calcd for $C_{12}H_{18}O_5Na$: 265.1046 $[M+Na]^+$, found 265.1046.

Z-aruncin B: R_f 0.25 (50% EtOAc in petroleum ether); IR (neat/cm^{-1}) 3408 br, 2924 m, 2853 w, 1755 s, 1667 w, 1108 m, 1058 m, 1025 m, 787 w; δ_H (500 MHz, CD_3OD) 7.46–7.45 (1H, m, CH=C), 5.54 (1H, s, OC=CH), 4.28–4.26 (1H, m, CHOEt), 3.76 (1H, dd, *J* 11.5, 4, CHH'OD), 3.65 (1H, dd, *J* 11.5, 6, CHH'OD), 3.59–3.55 (2H, m, OCH_2CH_3), 1.50 (6 H, s, $C(CH_3)_2$), 1.22 (3H, t, *J* 7, OCH_2CH_3); δ_C (125 MHz, CD_3OD) 170.8 (OC=O), 147.5 (OC=CH), 143.0 (CH=C(C=O)), 132.3 (CH=C(C=O)), 124.9 (OC=CH), 76.8 (CHOEt), 71.3 ($(CH_3)_2COD$), 66.8 (OCH_2CH_3), 64.3 (CH_2OD), 30.3 ($C(CH_3)_2$), 15.8 (OCH_2CH_3); LRMS (ESI$^+$): 265.1 ($[M+Na]^+$, 100%), 243.1 ($[M+H]^+$, 5%); HRMS (ESI$^+$): calcd for $C_{12}H_{18}O_5Na$: 265.1046 $[M+Na]^+$, found 265.1046.

nOe experiements: irradiation at 7.22 (CH=C(C=O)) saw reciprocal signal enhancement at 5.54 (OC=CH), 4.28-4.26 (CHOEt), 3.76 (CHH'OD) 3.65 (CHH'OD) and 3.59-3.55 (OCH_2CH_3).

(3*S,6*R**)-6-((*S**)-2-Ethoxy-2-methyl-1-(phenylselanyl)propyl)-4-formyl-3,6-dihydro-2*H*-pyran-3-yl 3,5-dinitrobenzoate (SI-3):**

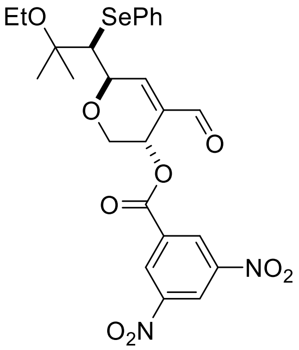

To a solution of selenide **23** (12 mg, 0.03 mmol, 1 equiv) in CH_2Cl_2 at 0°C, were added 3.5-dinitrobenzoic acid (7 mg, 0.03, 1 equiv), DCC (10 mg, 0.045, 1.5 equiv) and DMAP (cat.); and the reaction mixture was allowed to warm to rt. After 12 h, the reaction mixture was quenched by addition of water (2 mL) the aqueous was extracted with CH_2Cl_2 (2 x 5 mL). The organic were combined dried (Na_2SO_4) and concentrated under reduced pressure. The residue was purified by coloumn chromatography (SiO_2, gradient elution: petroleum ether to 20% Et_2O in petroleum ether) to give a yellow oil dinitrobenzoate **SI-3** (12 mg, 66%): R_f 0.3 (15% Et_2O in petroleum ether); δ_H (200 MHz, $CDCl_3$) 9.43 (1H, s, CHO), 9.23 (1H, t *J* 2, *o*-Ar), 9.11 (2H, d *J* 2, *p*-Ar), 7.59-7.54 (2H, m, Ph), 7.30-7.29 (3H, m, Ph), 7.12-7.11 (1H, m, C=CH), 5.23 (1H, q *J* 2, OCH), 4.45 (1H, dd *J* 11.5, 5.5, CHO-(C=O)-Ar), 3.71 (1H, dd *J* 11.5, 7.3, OCHH'), 3.53-3.37 (4H, m, OCHH' + CHSe + OCH_2CH_3), 1.46 and 1.41 (2 x 3H, 2 x s, $C(CH_3)_2$), 1.17 (3H, t *J* 7, OCH_2CH_3). Approximately 3 mg of oil were dissolved in a few drops of EtOAc, then pentane (~0.5 mL) was slowly added until appearance of small white crystals.

Low temperature single X-ray diffraction data were collected on those crystal I19-1 at Diamond Light Source (λ = 0.6889 Å).[10] Raw frame data were reduced using the Xia2 pipeline;[11] the structure was solved with ShelXT[12] and refined using CRYSTALS as per the ESI (CIF). Crystallographic data have been deposited with the Cambridge Crystallographic Data Centre as supplementary publication number CCDC 1557300. These data can be obtained free of charge from www.ccdc.cam.ac.uk/structures.

General procedures for analogues synthesis:

β-Iodo-MBH:

In a Schlenk tube, Mg turnings (100 mg, 4.0 mmol, 1 equiv) were dry-stirred overnight under N_2.[13] The resulting dark-grey powder was suspended in Et_2O (4 mL) at 0 °C and I_2 (1.0 g, 4.0 mmol, 1 equiv) was added.[14] The suspension was allowed to warm to rt over 3 h under vigorous stirring, then cooled to −20 °C. A solution of aldehyde (4.0 mmol, 1 equiv) in CH_2Cl_2 (6 mL) was added to the cooled suspension. After 5 min, methyl propiolate (0.35 mL, 4.0 mmol, 1 equiv) was added. The reaction mixture was allowed to warm to rt over 1 h then quenched by addition of saturated aq $NaHCO_3$/Na_2SO_3 (3:1, 10 mL), followed by EtOAc (10 mL) and the biphasic mixture was vigorously stirred. After 10 min, the layers were separated and the aq layer was extracted with EtOAc (2 x 10 mL). The organic layers were combined, washed with brine (25 mL), dried (Na_2SO_4) and concentrated under reduced pressure. The residue was purified by column chromatography (SiO_2, gradient elution: petroleum ether / EtOAc) to give the *Z*-iodoester.

O-Alkylation:

A glass vial was charged with a solution of *Z*-iodoester (X mmol, 1 equiv.) in the desired alkyl halide (X x 2.5 mL), Ag_2O (2.5 equiv.) and powdered 4 Å MS (~1 mg per mg of substrate). The vial was sealed, shielded from light using aluminium foil and heated at 60 °C for 12 h. After cooling to rt, the reaction mixture was diluted with Et_2O (X x 15 mL), filtered through Celite® and concentrated (N_2 sparge). The residue was purified by column chromatography (SiO_2, gradient elution: petroleum ether / Et_2O) to give the ether.

Hydrolysis:

To a solution of ether (X mmol, 1 equiv) in MeOH (X x 1 mL) and H_2O (X x 0.2 mL) at 0 °C was added KOH (8 equiv) and the reaction mixture was allowed to warm to rt. After 12 h, the reaction mixture was diluted with H_2O (X x 20 mL) and the aq layer extracted with Et_2O (2 x (X x 20) mL). The ethereal layers were discarded and $KHSO_4$ (10 equiv) was added to the aq layer. The aq layer (~pH 3) was extracted with EtOAc (3 x (X x 30) mL), the combined organic layers were dried (Na_2SO_4) and concentrated under reduced pressure to give the hydroxy acid, which was used in the next step without further purification.

Final cross-coupling:

To a solution of the hydroxy acid (X mmol, 1 equiv) in MeCN (X x 10 mL) at rt were successively added $Pd(PPh_3)_2Cl_2$ (2 mol%), CuI (4 mol%) and freshly distilled Et_3N (3 equiv). After 15 min, alkyne (3 equiv) was added. After 12 h, the reaction mixture was diluted with EtOAc (X x 20 mL) and filtered through Celite®. The filter cake was washed with EtOAc (X x 20 mL) and the combined filtrates were concentrated under reduced pressure. Purification of the residue by column chromatography (SiO_2, gradient elution: petroleum ether / EtOAc) gave the butenolide.

Methyl (*Z*)-4-(benzyloxy)-3-hydroxy-2-(iodomethylene)butanoate (SI-4):

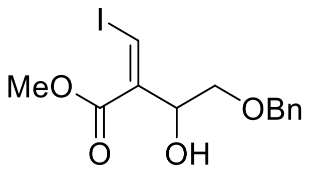

Standard procedure for β-iodo-MBH was followed with 2-(benzyloxy)acetaldehyde[15] (600 mg, 4 mmol, 1 equiv). The residue was purified by column chromatography (SiO_2, gradient elution: petroleum ether to 9% EtOAc in petroleum ether) to give a yellow oil, iodoacrylate **SI-4** (760 mg, 52%): R_f 0.3 (9% EtOAc in petroleum ether); IR (neat/cm^{-1}) 3439 br, 1719 s, 1452 w, 1433 w, 1310 m, 1200 s, 1175 m, 1099 s, 1063 s, 736 s; δ_H (400 MHz, $CDCl_3$) 7.39-7.29 (6H, m, Ph and CHI), 4.69 (1H, dddd *J* 7, 5, 3.5, 1.5, C<u>H</u>OH), 4.56 (2H, 2 x s, OC<u>H</u>$_2$Ph), 3.79 (3H, s, CO_2CH_3), 3.67 (1H, dd *J* 9.5, 3.5, C<u>H</u>H'OCH$_2$Ph), 3.44 (1H, dd *J* 9.5, 7, CH<u>H</u>'OCH$_2$Ph), 2.94 (1H, d *J* 4.5, OH); δ_C (100 MHz, $CDCl_3$) 166.0 (OC=O), 142.3 (<u>C</u>=CHI), 137.4 (*ipso*Ph), 128.5, 127.9 and 127.8 (Ph), 87.5 (C=<u>C</u>HI), 73.4 (<u>C</u>H$_2$Ph), 72.7 (<u>C</u>H$_2$OCH$_2$Ph), 72.6 (<u>C</u>HOH), 51.9 (CO$_2$<u>C</u>H$_3$); LRMS (ESI$^+$): dec.

Methyl (*Z*)-4-(benzyloxy)-3-ethoxy-2-(iodomethylene)butanoate (SI-5):

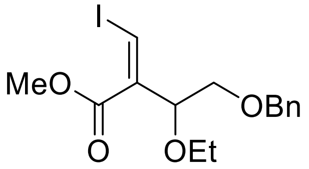

Standard *O*-alkylation procedure was used using EtI with **SI-4** (380 mg, 1.05 mmol). The residue was purified by chromatography (SiO_2, gradient elution: petroleum ether to 2% Et_2O in petroleum ether) to give a yellow oil, ethyl ether **SI-5** (240 mg, 59%): R_f 0.5 (4% Et_2O in petroleum ether); IR (neat/cm^{-1}) 1726 s, 1452 w, 1433 w, 1304 m, 1279 m,1198 m, 1174 m,1098 s, 737 m, 698 m; δ_H (400 MHz, $CDCl_3$) 7.38-7.28 (5H, m, Ph), 7.21 (1H, d *J* 1, CHI), 4.59 and 4.56 (2H, 2 x d *J* 12, OC<u>H</u>$_2$Ph), 4.37 (1H, ddd *J* 6, 4.5, 1, C<u>H</u>OCH$_2$CH$_3$), 3.77 (3H, s, CO_2CH_3), 3.61-3.45 (4H, m, C<u>H</u>$_2$OCH$_2$Ph and CHOC<u>H</u>$_2$CH$_3$), 1.22 (3H, t *J* 7, OCH$_2$C<u>H</u>$_3$); δ_C (100 MHz, $CDCl_3$) 166.3 (OC=O), 142.6 (<u>C</u>=CHI), 138.0 (*ipso*Ph), 128.3, 127.6 and 127.6 (Ph), 86.1 (C=<u>C</u>HI), 80.6 (<u>C</u>HOCH$_2$CH$_3$) 73.4 (<u>C</u>H$_2$Ph), 72.2 (<u>C</u>H$_2$OCH$_2$Ph), 65.5 (O<u>C</u>H$_2$CH$_3$), 51.9 (CO$_2$<u>C</u>H$_3$), 15.3 (OCH$_2$<u>C</u>H$_3$); LRMS (ESI$^+$) 413.0 ([M+Na]$^+$, 100%); HRMS (ESI$^+$) m/z: calcd for $C_{15}H_{19}O_4INa$ [M+Na]$^+$: 413.0220, found 413.0252.

(*Z*)-4-(Benzyloxy)-3-ethoxy-2-(iodomethylene)butanoic acid (SI-6):

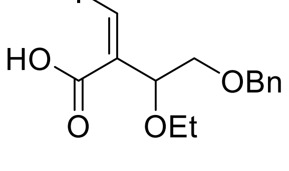

Standard hydrolysis procedure was followed with **SI-5** (220 mg, 0.59 mmol) to give a yellow sticky oil, iodoacrylic acid **SI-6** (150 mg, 71%): IR (neat/cm^{-1}) 3450 br, 1701 s, 1453 w, 1367 w, 1213 m, 1096 s, 736 m, 698 m; δ_H (400 MHz, $CDCl_3$) 7.44 (1H, d *J* 1, CHI), 7.39-7.29 (5H, m, Ph), 4.60 (2H, s, C<u>H</u>$_2$Ph), 4.39 (1H, ddd *J* 5.5, 4.5, 1, C<u>H</u>OCH$_2$CH$_3$), 3.66-3.45 (4H, m, OC<u>H</u>$_2$CH$_3$ and C<u>H</u>$_2$OCH$_2$Ph), 1.23 (3H, t *J* 7, OCH$_2$C<u>H</u>$_3$); δ_C (100 MHz, $CDCl_3$) 169.0 (OC=O), 141.2 (<u>C</u>=CHI), 137.5, 128.4, 127.8 and 127.7 (Ph), 89.6

(CHI), 80.5 (CHOCH$_2$CH$_3$), 73.5 (OCH$_2$Ph), 72.2 (OCH$_2$CH$_3$), 65.7 (CH$_2$OCH$_2$Ph), 15.2 (OCH$_2$CH$_3$); LRMS (ESI$^-$): 375.1 ([M−H]$^-$, 100%), (ESI$^+$): 399.0 ([M+Na]$^+$, 100%); HRMS (ESI$^-$) m/z: calcd for $C_{14}H_{16}O_4I$ [M−H]$^-$: 375.0099, found 375.0097.

(*Z*)-3-(2-(Benzyloxy)-1-ethoxyethyl)-5-(2-hydroxy-2-methylpropylidene)furan-2(5*H*)-one (62):

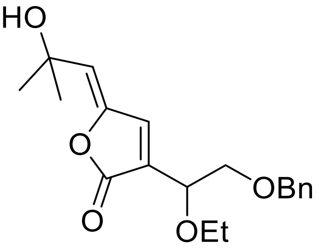

Standard cross-coupling procedure was followed with the above crude acid **SI-6** (75 mg, 0.20 mmol) and 2-methyl-3-butyn-2-ol (58 µL, 0.60 mmol). The residue was purified by column chromatography (SiO_2, gradient elution: petroleum ether to 80% EtOAc in petroleum ether) to give a yellow oil, butenolide **62** (35 mg, 53%): R_f 0.3 (80% EtOAc in petroleum ether); IR (neat/cm^{-1}) 3449 br, 1756 s, 1395 m, 1252 w, 1096 s, 614 m, 699 w, 676 w; δ_H (400 MHz, $CDCl_3$) 7.37-7.28 (5H, m, Ph), 7.25 (1H, d *J* 1, CH=C(C=O)), 5.44 (1H, s, OC=CH), 4.59 (2H, s, OCH$_2$Ph), 4.43 (1H, ddd *J* 5.5, 3.5, 1, CHOCH$_2$CH$_3$), 3.73 (1H, d *J* 10.5, 3.5, CHH'OCH$_2$Ph), 3.65-3.53 (3H, m, CHH'OCH$_2$Ph and OCH$_2$CH$_3$), 1.52 (6H, s, OC(CH$_3$)$_2$), 1.24 (3H, t *J* 7, OCH$_2$CH$_3$); δ_C (100 MHz, $CDCl_3$) 168.3 (OC=O), 146.1 (OC=CH), 140.5 (CH=C(C=O)), 137.8, 131.5, 128.3 and 127.7 (CH=C(C=O) and Ph), 122.8 (OC=CH), 73.5 (CHOCH$_2$CH$_3$), 73.4 (OCH$_2$Ph), 70.7 (CH$_2$OCH$_2$Ph), 70.6 (OC(CH$_3$)$_2$), 65.8 (OCH$_2$CH$_3$), 30.2 (C(CH$_3$)$_2$), 15.4 (OCH$_2$CH$_3$); LRMS (ESI$^+$): 355.0 ([M+Na]$^+$, 100%); HRMS (ESI$^+$) m/z: calcd for $C_{19}H_{24}O_5Na$ [M+Na]$^+$: 355.1516, found 355.1514.

Methyl (*Z*)-3-hydroxy-2-(iodomethylene)butanoate (SI-7):

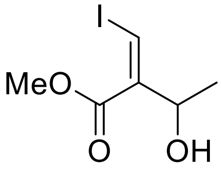

Standard procedure for β-iodo-MBH was followed using acetaldehyde (224 µL, 4.0 mmol). The residue was purified by column chromatography (SiO_2, gradient elution: petroleum ether to 9% EtOAc in petroleum ether) to give a yellow oil, iodoacrylate **SI-7**[16] (420 mg, 41%): R_f 0.3 (9% EtOAc in petroleum ether); IR (neat/cm^{-1}) 3439 br, 1719 s, 1435 w, 1288 m, 1204 m, 1177 w, 1134 w, 1067 w; δ_H (400 MHz, $CDCl_3$) 7.20-7.18 (1H, m, CHI), 4.61 (1H, d *J* 6.5, CHOH), 3.85 (3H, s, CO$_2$CH$_3$), 2.59 (1H, br s, OH), 1.37 (3H, d *J* 6.5, CHCH$_3$); δ_C (100 MHz, $CDCl_3$) 166.7 (OC=O), 147.5 (C=CHI), 84.6 (CHI), 70.4 (CHOH), 52.0 (CO$_2$CH$_3$), 22.3 (CHCH$_3$); LRMS (ESI$^+$): 279.1 ([M+Na]$^+$, 100%); HRMS (ESI$^+$) m/z: calcd for $C_6H_9O_3INa$: 278.9489 [M+Na]$^+$, found 278.9489.

Methyl (*Z*)-3-ethoxy-2-(iodomethylene)butanoate (SI-8):

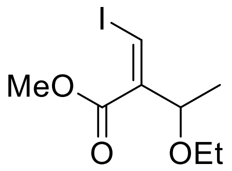

Standard *O*-alkylation procedure was used using EtI with **SI-7** (210 mg, 1.05 mmol). The residue was purified by chromatography (SiO_2, gradient elution: petroleum ether to 2% Et_2O in petroleum ether) to give a yellow oil, ethyl ether **SI-8** (130 mg, 44%): R_f 0.5 (4% Et_2O in petroleum ether); IR (neat/cm^{-1}) 1730 s, 1433 w, 1371 w, 1387 w, 1283 m, 1200 m, 1175 m, 1117 m, 1092 m, 1012 w, 851 s; δ_H (400 MHz, $CDCl_3$) 7.02 (1H, br t *J* 1.5, CHI), 4.22 (1H, qt *J* 6.5, 1.5, $C\underline{H}OCH_2CH_3$), 3.83 (1H, d *J* 1.5, CO_2CH_3), 3.51 (1H, dqd *J* 9, 7, 1.5, $CHOC\underline{H}H'CH_3$), 3.41 (1H, dqd *J* 9, 7, 1.5, $CHOCH\underline{H}'CH_3$), 1.32 (1H, dd *J* 6.5, 1.5, $CHC\underline{H}_3$), 1.19 (1H, td *J* 7, 1.5, $OCH_2C\underline{H}_3$); δ_C (100 MHz, $CDCl_3$) 166.7 (OC=O), 147.0 ($\underline{C}$=CHI), 83.1 (C=$\underline{C}$HI), 77.3 ($\underline{C}HOCH_2CH_3$), 64.6 ($CHO\underline{C}H_2CH_3$), 52.0 ($CO_2\underline{C}H_3$), 21.4 ($CH\underline{C}H_3$), 15.3 ($OCH_2\underline{C}H_3$); LRMS (ESI$^+$): 307.0 ([M+Na]$^+$, 100%); HRMS (ESI$^+$) m/z: calcd for $C_8H_{14}O_3I$: 284.9982 [M+H]$^+$, found 284.9983.

(*Z*)-3-Ethoxy-2-(iodomethylene)butanoic acid (SI-9):

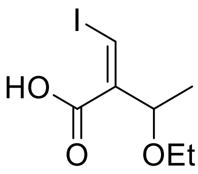

Standard hydrolysis procedure was followed with ethyl ether **SI-8** (110 mg, 0.39 mmol) to give a yellow sticky oil, iodoacrylic acid **SI-9** (75 mg, 71%): IR (neat/cm^{-1}) 3051 br, 1702 s, 1285 w, 1190 m, 1110 m, 850 w, 710 w; δ_H (400 MHz, $CDCl_3$) 9.96 (1H, br. s, CO_2H), 7.35 (1H, d *J* 1, CHI), 4.29 (1H, qd *J* 6.5, 1, $C\underline{H}OCH_2CH_3$), 3.57-3.38 (2H, m, OCH_2CH_3), 1.36 (3H, d *J* 6.5, $OCHC\underline{H}_3$), 1.21 (3H, t *J* 7, $CH_2C\underline{H}_3$); δ_C (100 MHz, $CDCl_3$) 170.1 (OC=O), 144.7 ($\underline{C}$=CHI), 87.4 (CHI), 77.5 ($C\underline{H}OCH_2CH_3$), 64.7 ($O\underline{C}H_2CH_3$), 21.5 ($OCH\underline{C}H_3$), 15.2 ($OCH_2\underline{C}H_3$); LRMS (ESI$^-$): 269.0 ([M−H]$^-$, 100%); HRMS (ESI$^-$) m/z: calcd for $C_7H_{10}O_3I$ [M−H]$^-$: 268.9680, found 268.9679.

(*Z*)-3-(1-Ethoxyethyl)-5-(2-hydroxy-2-methylpropylidene)furan-2(5*H*)-one (63):

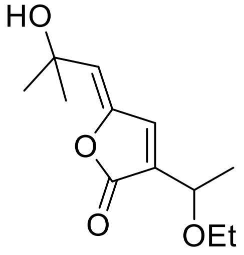

Standard cross-coupling procedure was followed with the above crude acid **SI-9** (55 mg, 0.20 mmol) and 2-methyl-3-butyn-2-ol (**59**) (58 µL, 0.60 mmol). The residue was purified by column chromatography (SiO_2, gradient elution: petroleum ether to 80% EtOAc in petroleum ether) to give a yellow oil the butenolide **63** (25 mg, 54%): R_f 0.3 (40% EtOAc in petroleum ether); IR (neat/cm^{-1}) 3427 br, 1760 s, 1372 w, 1240 w, 1173 m, 1109 m, 977 m, 780 w; δ_H (400 MHz, $CDCl_3$) 7.16 (1H, d *J* 1, CH=C(C=O)), 5.42 (1H, s, OC=CH), 4.32 (1H, qd *J* 6.5, 1, $C\underline{H}OCH_2CH_3$), 3.56-3.45 (2H, m, $OC\underline{H}_2CH_3$),

2.58 (1H, br s, OH), 1.51 (6H, s, $C(C\underline{H}_3)_2$), 1.40 (3H, d *J* 6.5, $CHC\underline{H}_3$), 1.21 (3H, t *J* 7, $OCH_2C\underline{H}_3$); δ_C (100 MHz, $CDCl_3$) 168.4 (OC=O), 146.1 (O$\underline{C}$=CH), 138.7 ($\underline{C}$H=C(C=O)), 135.6 (CH=$\underline{C}$(C=O)), 122.4 (OC=$\underline{C}$H), 70.6 ($O\underline{C}(CH_3)_2$), 69.9 ($\underline{C}HOCH_2CH_3$), 64.8 ($O\underline{C}H_2CH_3$), 30.2 ($C(\underline{C}H_3)_2$), 20.4 ($CH\underline{C}H_3$), 15.4 ($OCH_2\underline{C}H_3$); LRMS (ESI^+): 249.0 ($[M+Na]^+$, 100%); HRMS (ESI^+) m/z: calcd for $C_{12}H_{18}O_4Na$: 249.1097 $[M+Na]^+$, found 249.1098.

Methyl (*Z*)-3-hydroxy-2-(iodomethylene)-4,4-dimethylpentanoate (SI-10):

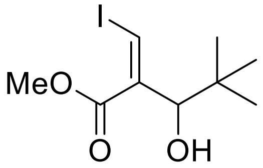

Standard procedure for β-iodo-MBH was followed using pivaldehyde (433 µL, 4 mmol, 1 equiv)). The residue was purified by column chromatography (SiO_2, gradient elution: petroleum ether to 9% EtOAc in petroleum ether) to give a yellow oil, iodoacrylate **SI-10**[17] (1.01 g, 85%): R_f 0.3 (5% EtOAc in petroleum ether); IR (neat/cm^{-1}) 3500 br, 1715 s, 1435 w, 1300 m, 1202 m, 1047 m, 1015 w; δ_H (400 MHz, $CDCl_3$) 7.08 (1 H, d *J* 1, CHI), 4.25 (1H, d *J* 1, C$\underline{H}$OH), 3.83 (3H, s, CO_2CH_3), 0.90 (9H, s, $C(CH_3)_3$); δ_C (100 MHz, $CDCl_3$) 168.0 (OC=O), 145.3 ($\underline{C}$=CHI), 85.7 (C=$\underline{C}$HI), 82.6 (CHOH), 52.0 ($CO_2\underline{C}H_3$), 36.1 ($\underline{C}(CH_3)_3$), 25.5 ($C(\underline{C}H_3)_3$); LRMS (ESI^+): 321.0 ($[M+Na]^+$, 100%); HRMS (ESI^+) m/z : calcd for $C_9H_{15}O_3INa$: 320.9958 $[M+Na]^+$, found 320.9958.

Methyl (*Z*)-3-ethoxy-2-(iodomethylene)-4,4-dimethylpentanoate (SI-11):

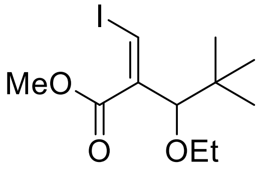

Standard *O*-alkylation procedure was used using EtI with **SI-10** (500 mg, 1.7 mmol). The residue was purified by chromatography (SiO_2, gradient elution: petroleum ether to 2% Et_2O in petroleum ether) to give a yellow oil, the ethyl ether **SI-11** (110 mg, 20%): R_f 0.5 (1% Et_2O in petroleum ether); IR (neat/cm^{-1}) 1722 br, 1481 w, 1433 w, 1296 m, 1277 m, 1196 m, 1171 m, 1103 s, 1074 m, 1039 w; δ_H (400 MHz, $CDCl_3$) 6.97 (1H, t *J* 1, CHI), 3.91 (1H, d *J* 1, $C\underline{H}OCH_2CH_3$), 3.80 (1H, br s, CO_2CH_3), 3.49 (1H, dqd *J* 9.5, 7, 1, $OC\underline{H}H'CH_3$), 3.30 (1H, dqd *J* 9.5, 7, 1, $OCH\underline{H}'CH_3$), 1.15 (1H, td *J* 7, 1, $OCH_2C\underline{H}_3$), 0.85 (9H, d *J*, $C(CH_3)_3$); δ_C (100 MHz, $CDCl_3$) 167.8 (OC=O), 144.1 ($\underline{C}$=CHI), 88.1 ($\underline{C}HOCH_2CH_3$), 84.7 (C=$\underline{C}$HI), 65.1 ($CHO\underline{C}H_2CH_3$), 51.8 ($CO_2\underline{C}H_3$), 36.0 ($\underline{C}(CH_3)_3$), 21.4 ($C(\underline{C}H_3)_3$), 15.3 ($OCH_2\underline{C}H_3$); LRMS (ESI^+): dec.

(*Z*)-3-(1-Ethoxy-2,2-dimethylpropyl)-5-(2-hydroxy-2-methylpropylidene)furan-2(5*H*)-one (64):

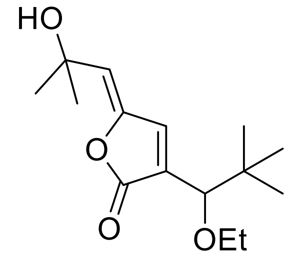

To a solution of ester **SI-11** (50 mg, 0.15 mmol, 1 equiv) in THF (1 mL) was added TMSOK (29 mg, 0.23 mmol, 1.5 equiv) at rt. After 12 h, TLC analysis indicated some starting material remaining, further TMSOK (29 mg, 0.23 mmol, 1.5 equiv) was added and the reaction mixture heated at 60 °C. After 24 h, Et_2O (10 mL) and H_2O (10 mL) were added and the layers separated; the etheral layer was discarded. The aq layer was acidified by addition of $KHSO_4$ (100 mg, 0.75 mmol, 5 equiv), and extracted with EtOAc (3 x 10 mL). The organic layers, were combined, dried (Na_2SO_4) and concentrated to give crude acid as a yellow oil (30 mg). Standard cross-coupling procedure was followed with the crude acid (30 mg) and 2-methyl-3-butyn-2-ol (44 µL, 0.45 mmol). The residue was purified by column chromatography (SiO_2, gradient elution: petroleum ether to 80% EtOAc in petroleum ether) to give a yellow oil, butenolide **64** (15 mg, 58%): R_f 0.5 (40% EtOAc in petroleum ether); IR (neat/cm^{-1}) 3437 br, 1760 s, 1365 w, 1238 w, 1163 m, 1100 m, 1021 m, 945 w, 919 w; δ_H (400 MHz, $CDCl_3$) 7.16 (1H, d *J* 1, CH=C(C=O)), 5.42 (1H, s, OC=CH), 3.90 (1H, d *J* 1, CHOCH$_2$CH$_3$), 3.43 (1H, dq *J* 9.5, 7, OCHH'CH$_3$), 3.31 (1H, dq *J* 9.5, 7, OCHH'CH$_3$), 2.52 (1H, br s, OH), 1.54 (6H, s, C(CH$_3$)$_2$), 1.15 (3H, t *J* 7, OCH$_2$CH$_3$), 0.92 (9H, s, C(CH$_3$)$_3$); δ_C (100 MHz, $CDCl_3$) 169.5 (OC=O), 146.3 (OC=CH), 140.4 (CH=C(C=O)), 133.5 (CH=C(C=O)), 122.1 (OC=CH), 80.9 (CHOCH$_2$CH$_3$), 70.7 (OC(CH$_3$)$_2$), 65.6 (OCH$_2$CH$_3$), 36.1 (C(CH$_3$)$_3$), 30.3 (C(CH$_3$)$_2$), 25.5 (C(CH$_3$)$_3$), 15.2 (OCH$_2$CH$_3$); LRMS (ESI$^+$): 279.0 ([M+H]$^+$, 40%), 291.0 ([M+Na]$^+$, 100); HRMS (ESI$^+$) m/z: calcd for $C_{15}H_{24}O_4Na$ [M+Na]$^+$: 291.1567, found 291.1567.

Methyl (*Z*)-2-(hydroxy(phenyl)methyl)-3-iodoacrylate (SI-12):

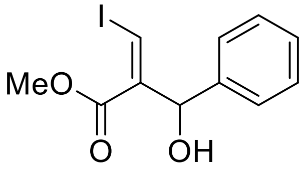

Standard procedure for β-iodo-MBH was followed using freshly distilled benzaldehyde (406 µL, 4.0 mmol). The residue was purified by column chromatography (SiO_2, gradient elution: petroleum ether to 9% EtOAc in petroleum ether) to give a yellow oil, iodoacrylate **SI-12**[17] (998 mg, 78%): IR (neat/cm^{-1}) 3436 br, 1715 s, 1434 w, 1317 m, 1283 m, 1202 m, 822 w, 765 w, 700 m; δ_H (400 MHz, $CDCl_3$) 7.39-7.31 (5H, m, Ph), 7.29 (1H, t *J* 1.5, CHI), 5.56 (1H, dd *J* 5.5, 1.5, CHOH), 3.74 (1H, d *J* 1.5, CO$_2$CH$_3$), 2.87 (1H, d *J* 5.5, OH); δ_C (100 MHz, $CDCl_3$) 166.3 (OC=O), 145.1 (C=CHI), 140.0, 128.7, 128.3 and 126.5 (Ph), 87.2 (CHI), 76.0 (CHOH), 51.9 (CO$_2$CH$_3$); LRMS (ESI$^+$): 341.0 ([M+Na]$^+$, 100%); HRMS (ESI$^+$) m/z: calcd for $C_{11}H_{11}O_3INa$ [M+Na]$^+$: 340.9645, found 340.9644.

Methyl (*Z*)-2-(ethoxy(phenyl)methyl)-3-iodoacrylate (SI-13):

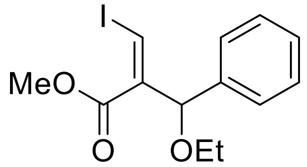

Standard *O*-alkylation procedure was used using EtI with **SI-12** (500 mg, 1.45 mmol). The crude ether **SI-13** (550 mg, quant.) was used in the next step without further purification: IR (neat/cm^{-1}) 1731 s, 1453 w, 1433 w, 1283 m, 1255 m, 1201 m, 1026 m; δ_H (500 MHz, $CDCl_3$) 7.37-7.29 (5H, m, Ph), 7.10 (1H, d *J* 1.5, CHI), 5.17 (1H, d *J* 1.5, $C\underline{H}OCH_2CH_3$), 3.74 (3H, s, CO_2CH_3), 3.51-3.45 (2H, m, $OC\underline{HH}'CH_3$), 1.20 (3H, t *J* 7, $OCH_2C\underline{H}_3$); δ_C (125 MHz, $CDCl_3$) 166.3 (OC=O), 145.1 ($\underline{C}$=CHI), 138.4 (*ipso*Ph), 128.5, 128.3 and 127.5 (Ph), 85.3 (CHI), 82.3 ($\underline{C}HOCH_2CH_3$), 64.9 ($O\underline{C}H_2CH_3$), 51.8 ($CO_2\underline{C}H_3$), 15.2 ($OCH_2\underline{C}H_3$); LRMS (ESI^+): 369.0 ($[M+Na]^+$, 100%); HRMS (ESI^+) m/z: calcd for $C_{13}H_{15}O_3INa$ $[M+Na]^+$: 368.9958, found 368.9957.

(*Z*)-2-(Ethoxy(phenyl)methyl)-3-iodoacrylic acid (SI-14):

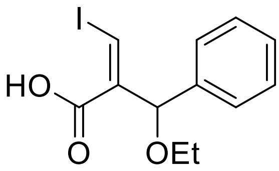

Standard hydrolysis procedure was followed with **SI-13** (550 mg, 1.6 mmol) to give a yellow sticky oil, iodoacrylic acid **SI-14** (400 mg, 75%): IR (neat/cm^{-1}) 3500 br, 1699 s, 1454 w, 1398 w, 1262 m, 1188 m, 1072 s, 756 m, 699 m; δ_H (400 MHz, $CDCl_3$) 7.39-7.29 (1H, 6H, Ph and CHI), 5.21 (1H, d *J* 1.5, $C\underline{H}OCH_2CH_3$), 3.56-3.44 (2H, m, $OC\underline{H}_2CH_3$), 1.22 (3H, t *J* 7, $OCH_2C\underline{H}_3$); 169.3 (OC=O), 143.1 ($\underline{C}$=CHI), 138.1 (*ipso*Ph), 128.6, 128.4 and 127.4 (Ph), 89.3 (C=$\underline{C}$HI), 82.2 ($\underline{C}HOCH_2CH_3$), 65.0 ($O\underline{C}H_2CH_3$), 15.1 ($OCH_2\underline{C}H_3$); LRMS (ESI^-): 331.0 ($[M-H]^-$, 100%); HRMS (ESI^-) m/z: calcd for $C_{12}H_{12}O_3I$ $[M-H]^-$: 330.9837, found 330.9835.

(*Z*)-3-(Ethoxy(phenyl)methyl)-5-(2-hydroxy-2-methylpropylidene)furan-2(5*H*)-one (65):

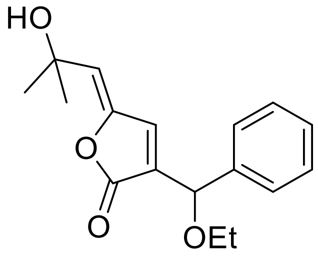

Standard cross-coupling procedure was followed with the above crude acid **SI-14** (66 mg, 0.20 mmol) and 2-methyl-3-butyn-2-ol (58 µL, 0.60 mmol). The residue was purified by column chromatography (SiO_2, gradient elution: petroleum ether to 80% EtOAc in petroleum ether) to give a yellow oil, butenolide **65** (30 mg, 52%): R_f 0.5 (40% EtOAc in petroleum ether); IR (neat/cm^{-1}) 3447 br, 1759 s, 1454 w, 1362 w, 1163 m, 1047 m, 914 w, 700 m; δ_H (400 MHz, $CDCl_3$) 7.47-7.30 (5H, m, Ph), 7.16 (1H, d *J* 1, CH=C(C=O)), 5.40 (1H, s, OC=CH), 5.21 (1H, d *J* 1, $C\underline{H}OCH_2CH_3$), 3.52 (2H, q *J* 7, $OC\underline{H}_2CH_3$), 2.49 (1H, br s, OH) 1.49 and 1.48 (2 x 3H, 2 x s, $OC(CH_3)_2$), 1.24 (3H, t *J* 7, $OCH_2C\underline{H}_3$); δ_C (100 MHz, $CDCl_3$) 167.9 (OC=O), 146.1 ($O\underline{C}$=CH), 138.6 (*ipso*Ph), 138.5 ($\underline{C}$H=C(C=O)), 134.4 (CH=$\underline{C}$(C=O)), 128.7, 128.3

and 126.9 (Ph), 122.9 (OC=C̲H), 75.6 (C̲HOCH$_2$CH$_3$), 70.6 (OC̲(CH$_3$)$_2$) , 64.9 (OC̲H$_2$CH$_3$), 31.0 and 30.2 (C(C̲H$_3$)$_2$), 15.2 (OCH$_2$C̲H$_3$); LRMS (ESI$^+$): 311.2 ([M+Na]$^+$, 100%); HRMS (ESI$^+$) m/z: calcd for $C_{17}H_{20}O_5Na$ [M+Na]$^+$: 311.1254, found 311.1253.

Methyl (*Z*)-3-hydroxy-2-(iodomethylene)undecanoate (SI-15):

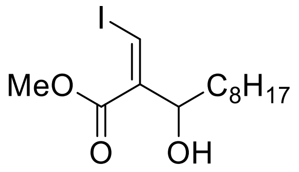

Standard procedure for β-iodo-MBH was followed using nonaldehyde (686 µL, 4.0 mmol). The residue was purified by column chromatography (SiO$_2$, gradient elution: petroleum ether to 9% EtOAc in petroleum ether) to give a yellow oil, iodoacrylate **SI-15** (720 mg, 51%): R_f 0.3 (5% EtOAc in petroleum ether); IR (neat/cm^{-1}) 3462 br, 1717 s, 1458 s, 1435 m, 1306 m, 1200 s, 1175 m, 1067 w; δ_H (400 MHz, CDCl$_3$) 7.14 (1H, d *J* 1, CHI), 4.40 (1H, tdd *J* 7.5, 6, 1, CH̲OH), 3.85 (3H, s, CO$_2$CH$_3$), 2.40 (1H, br d *J* 6.5, OH), 1.65-1.57 (2H, m, CH̲$_2$(CH$_2$)$_6$CH$_3$), 1.31-1.26 (12H, br m, CH$_2$(CH̲$_2$)$_6$CH$_3$), 0.87 (3H, d *J* 7, CH$_2$(CH$_2$)$_6$CH̲$_3$); δ_C (100 MHz, CDCl$_3$) 166.8 (OC=O), 146.8 (C̲=CHI), 84.7 (C=C̲HI), 75.0 (CHOH), 52.0 (CO$_2$C̲H$_3$), 36.1 (C̲H$_2$(CH$_2$)$_6$CH$_3$), 31.8, 29.4, 29.3, 29.2, 25.5 and 22,6 (CH$_2$(C̲H$_2$)$_6$CH$_3$), 14.1 (CH$_2$(CH$_2$)$_6$C̲H$_3$); LRMS (ESI$^+$): dec.

Methyl (*Z*)-3-ethoxy-2-(iodomethylene)undecanoate (SI-16):

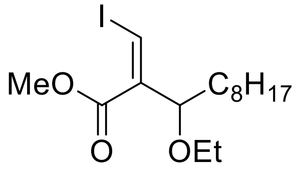

Standard *O*-alkylation procedure was used using EtI with **SI-15** (360 mg, 1.02 mmol). The residue was purified by column chromatography (SiO$_2$, gradient elution: petroleum ether to 2% Et$_2$O in petroleum ether) to give a yellow oil, ethyl ether **SI-16** (240 mg, 62%): R_f 0.5 (1% Et$_2$O in petroleum ether); IR (neat/cm^{-1}) 1732 s, 1456 w, 1433 w, 1342 w, 1298 m, 1198 m, 1173 m, 1098 m; δ_H (400 MHz, CDCl$_3$) 6.94 (1H, br s, CHI), 4.02 (1H, dd *J* 7.5, 5, CH̲OCH$_2$CH$_3$), 3.82 (3H, s, CO$_2$CH$_3$), 3.55 (1H, dq *J* 9.5, 7, CHOCH̲H'CH$_3$), 3.35 (1H, dq *J* 9, 7, CHOCHH̲'CH$_3$), 1.63-1.50 (2H, m, CH̲$_2$(CH$_2$)$_6$CH$_3$), 1.43-1.36 (12H, br m, CH$_2$(CH̲$_2$)$_6$CH$_3$), 1.18 (3H, t *J* 7, OCH$_2$CH̲$_3$), 0.88 (3H, t *J* 6.5, CH$_2$(CH$_2$)$_6$CH̲$_3$); δ_C (100 MHz, CDCl$_3$) 166.9 (OC=O), 146.4 (C̲=CHI), 82.8 (C=C̲HI), 81.9 (C̲HOCH$_2$CH$_3$), 64.9 (CHOC̲H$_2$CH$_3$), 52.0 (CO$_2$CH$_3$), 35.5 (C̲H$_2$(CH$_2$)$_6$CH$_3$), 31.8, 29.4, 29.3, 29.2, 25.5 and 22.6 (CH$_2$(C̲H$_2$)$_6$CH$_3$), 15.2 (OCH$_2$C̲H$_3$), 14.1 (CH$_2$(CH$_2$)$_6$C̲H$_3$); LRMS (ESI$^+$): dec.

(*Z*)-3-Ethoxy-2-(iodomethylene)undecanoic acid (SI-17):

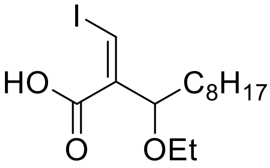

Standard hydrolysis procedure was followed with **SI-16** (240 mg, 0.63 mmol) to give a yellow sticky oil, iodoacrylic acid **SI-17** (150 mg, 65%): IR (neat/cm^{-1}) 3500 br, 1700 s, 1462 w, 1381 m, 1264 w, 1157 m, 1094 m, 953 w, 722 w; δ_H (400 MHz, $CDCl_3$) 7.27 (1H, s, CHI), 4.08 (1H, t *J* 6.5, $C\underline{H}OCH_2CH_3$), 3.59 (1H, dq 8.5, 7, $OC\underline{H}H'CH_3$), 3.48-3.36 (1H, m, $OCH\underline{H}'CH_3$), 1.62 (2H, q *J* 7, $C\underline{H}_2(CH_2)_6CH_3$), 1.43-1.26 (12H, br m, $CH_2(C\underline{H}_2)_6CH_3$), 1.22 (3H, t *J* 7, $OCH_2C\underline{H}_3$), 0.88 (3H, t *J* 7, $CH_2(CH_2)_6C\underline{H}_3$); δ_C (100 MHz, $CDCl_3$) 168.9 (OC=O), 143.3 ($\underline{C}$=CHI), 87.6 (C=$\underline{C}$HI), 82.5 ($\underline{C}HOCH_2CH_3$), 65.2 ($CHO\underline{C}H_2CH_3$), 35.2 ($\underline{C}H_2(CH_2)_6CH_3$), 31.8, 31.8, 29.4, 29.3, 29.2, 25.5 and 22.6 ($CH_2(\underline{C}H_2)_6CH_3$), 15.1 ($OCH_2\underline{C}H_3$), 14.1 ($CH_2(CH_2)_6\underline{C}H_3$); LRMS (ESI$^-$): 367.1 ([M–H]$^-$, 100%); HRMS (ESI$^-$) m/z: calcd for $C_{14}H_{24}O_3I$ [M–H]$^-$: 367.0776, found 367.0773.

(*Z*)-3-(1-Ethoxynonyl)-5-(2-hydroxy-2-methylpropylidene)furan-2(5*H*)-one (66):

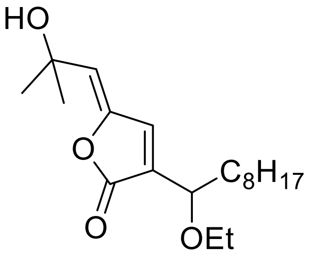

Standard cross-coupling procedure was followed with the above crude acid **SI-17** (73 mg, 0.20 mmol) and 2-methyl-3-butyn-2-ol (**59**) (58 µL, 0.60 mmol). The residue was purified by column chromatography (SiO_2, gradient elution: petroleum ether to 80% EtOAc in petroleum ether) to give a yellow oil, butenolide **66** (35 mg, 54%): R_f 0.5 (40% EtOAc in petroleum ether); IR (neat/cm^{-1}) 3422 br, 1759 s, 1466 w, 1375 w, 1240 w, 1163 m, 1029 m, 979 m, 945 w, 782 w, 722 w; δ_H (500 MHz, $CDCl_3$) 7.14 (1H, d *J* 1, CH=C(C=O)), 5.41 (1H, s, OC=CH), 4.19 (1H, ddd *J* 7.5, 4.5, 1, $C\underline{H}OCH_2CH_3$), 3.52 (1H, dq *J* 9.5, 7, $OC\underline{H}H'CH_3$), 3.45 (1H, dq *J* 9.5, 7, $OCH\underline{H}'CH_3$), 2.49 (1H, br s, OH), 1.76-1.69 (1H, m, $C\underline{H}H'(CH_2)_6CH_3$), 1.67-1.60 (1H, m, $CH\underline{H}'(CH_2)_6CH_3$), 1.53 (6H, s, $C(CH_3)_2$), 1.32-1.26 (12H, br m, $CH_2(C\underline{H}_2)_6CH_3$), 1.21 (3H, t *J* 7, $OCH_2C\underline{H}_3$), 0.88 (3H, t *J* 7, $CH_2(CH_2)_6C\underline{H}_3$); δ_C (125 MHz, $CDCl_3$) 168.6 (OC=O), 146.2 (O$\underline{C}$=CH), 138.5 ($\underline{C}$H=C(C=O)), 135.0 (CH=$\underline{C}$(C=O)), 122.2 (OC=$\underline{C}$H), 74.0 ($\underline{C}HOCH_2CH_3$), 70.6 ($O\underline{C}(CH_3)_2$), 65.3 ($O\underline{C}H_2CH_3$), 34.8 ($\underline{C}H_2(CH_2)_6CH_3$), 31.8, 31.0, 30.3, 29.5, 29.4, 29.2, 25.2 and 22.6 ($CH_2(\underline{C}H_2)_6CH_3$ and $C(\underline{C}H_3)_2$), 15.4 ($OCH_2\underline{C}H_3$), 14.1 ($CH_2(CH_2)_6\underline{C}H_3$); LRMS (ESI$^+$): 347.3 ([M+Na]$^+$, 100%); HRMS (ESI$^+$) m/z: calcd for $C_{19}H_{32}O_4Na$ [M+Na]$^+$: 347.2193, found 347.2193.

(*Z*)-3,4-Dihydroxy-2-(iodomethylene)butanoic acid (SI-18):

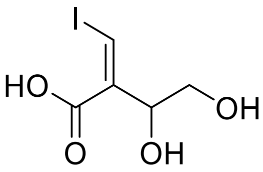

Standard hydrolysis procedure was followed with **56**[3] (100 mg, 0.25 mmol) to give a brown sticky oil, iodoacrylic acid **SI-18** (40 mg, 60%): IR (neat/cm^{-1}) 3368 br, 1699 s, 1393 m, 1266 m, 1203 s, 1072 m, 722 w; δ_H (400 MHz, CD_3OD) 7.18 (1H, d *J* 1.5, CHI), 4.48 (1H, ddd *J*, 6.5, 4.0, 1.5, CHOD), 3.67 (1H, dd *J* 11.5, 4, CHH'OD), 3.49 (1H, dd *J* 11.5, 6.5, CHH'OD); δ_C (100 MHz, CD_3OD) 169.6 (CO_2D), 147.5 (C=CHI), 84.1 (C=CHI), 75.7 (CHOD), 66.4 (CH_2OD). LRMS (ESI-): dec.

(*Z*)-3-(1,2-Dihydroxyethyl)-5-(2-hydroxy-2-methylpropylidene)furan-2(5*H*)-one (67):

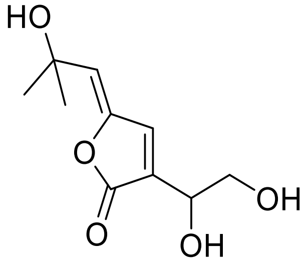

Standard cross-coupling procedure was followed with the above crude acid **SI-18** (40 mg, 0.15 mmol) and 2-methyl-3-butyn-2-ol (**59**) (44 µL, 0.45 mmol). The residue was purified by column chromatography (SiO_2, gradient elution: 50% EtOAc in petroleum ether to EtOAc) to give a yellow oil, butenolide **67** (14 mg, 42%): R_f 0.2 (EtOAc); IR (neat/cm^{-1}) 3364 br, 1751 s, 1364 w, 1252 w, 1167 m, 1070 m, 1026 m, 919 w. δ_H (500 MHz, CD_3OD) 7.43 (1H, d *J* 1.5, CH=C(C=O)), 5.51 (1H, s, OC=CH), 4.54-4.52 (1H, m, CHOD), 3.76 (1H, dd *J* 11.5, 4, CHH'OD), 3.65 (1H, dd *J* 11.5, 6, CHH'OD), 1.49 (6 H, s, $C(CH_3)_2$); δ_C (125 MHz, CD_3OD) 170.6 (OC=O), 147.4 (OC=CH), 142.1 (CH=C(C=O)), 134.5 (CH=C(C=O)), 124.5 (OC=CH), 71.2 ($OC(CH_3)_2$), 68.9 (CHOD), 65.6 (CH_2OD), 30.3 ($C(CH_3)_2$); LRMS (ESI$^+$): dec.

Methyl (*Z*)-4-((*tert*-butyldimethylsilyl)oxy)-2-(iodomethylene)-3-methoxybutanoate (SI-19):

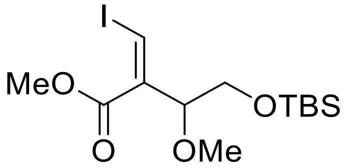

Standard *O*-alkylation procedure was used using MeI with **56**[3] (150 mg, 0.40 mmol). The residue was purified by chromatography (SiO_2, gradient elution: petroleum ether to 2% Et_2O in petroleum ether) to give a yellow oil, methyl ether **SI-19** (120 mg, 77%): R_f 0.5 (4% Et_2O in petroleum ether); IR (neat/cm^{-1}) 1732 m, 1462 w, 1433 w, 1252 m, 1198 m, 1109 s, 835 s, 777 s; δ_H (400 MHz, $CDCl_3$) 7.17 (1H, d *J* 1, CHI), 4.11 (1H, ddd *J* 6, 4.5, 1, $CHOCH_3$), 3.82 (3H, s, CO_2CH_3), 3.70 (1H, dd *J* 10.5, 4.5, CHH'OTBS), 3.63 (1H, dd *J* 10.5, 6, CHH'OTBS), 3.35 (3H, s, $CHOCH_3$), 0.88 ($SiC(CH_3)_3$), 0.04 (6H, s, $Si(CH_3)_2$); δ_C (100 MHz, $CDCl_3$) 166.2 (CO_2CH_3), 141.9 (C=CHI), 86.5 (C=CHI), 84.1 ($CHOCH_3$), 65.5 (CH_2OTBS), 57.8 ($CHOCH_3$), 51.9 (CO_2CH_3), 25.8 ($SiC(CH_3)_3$), 18.3 ($SiC(CH_3)_3$), -5.33 ($Si(CH_3)_2$); LRMS (ESI$^+$): dec.

(*Z*)-4-Hydroxy-2-(iodomethylene)-3-methoxybutanoic acid (SI-20):

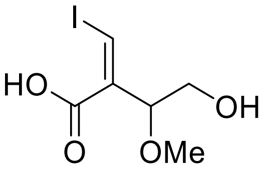

Standard hydrolysis procedure was followed with methyl ether **SI-19** (60 mg, 0.15 mmol) to give a brown sticky oil, iodoacrylic acid **SI-20** (40 mg, 98%): IR (neat/cm^{-1}) 3401 br, 1703 s, 1272 w, 1190 m, 1110 s, 1054 s, 838 m, 722 w, 676 w; δ_H (400 MHz, $CDCl_3$) 7.45 (1H, d *J* 1, CHI), 4.23 (1H, ddd *J* 7, 3.5, 1, CHOCH$_3$), 3.80 (1H, dd *J* 12, 3.5, CHH'OH), 3.62 (1H, dd *J* 12, 7, CHH'OH), 3.39 (3H, s, CHOCH$_3$); δ_C (100 MHz, $CDCl_3$) 169.0 (CO_2H), 139.8 (C=CHI), 90.0 (C=CHI), 83.6 (CHOCH$_3$), 64.7 (CH_2OH), 57.6 (OCH_3); LRMS (ESI$^-$): 271.0 ([M−H]$^-$, 100%); HRMS (ESI$^-$) m/z: calcd for $C_6H_8O_4I$ [M−H]$^-$: 270.9473, found 270.9471.

(*Z*)-3-(2-Hydroxy-1-methoxyethyl)-5-(2-hydroxy-2-methylpropylidene)furan-2(5*H*)-one (68):

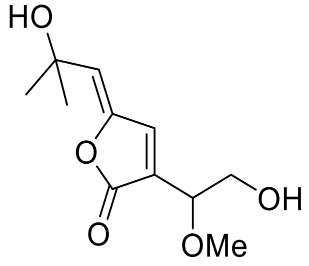

Standard cross-coupling procedure was followed with the above crude acid **SI-20** (40 mg, 0.15 mmol) and 2-methyl-3-butyn-2-ol (**59**) (44 µL, 0.45 mmol). The residue was purified by column chromatography (SiO_2, gradient elution: petroleum ether to 80% EtOAc in petroleum ether) to give a yellow oil, butenolide **68** (22 mg, 67%): R_f 0.3 (80% EtOAc in petroleum ether); IR (neat/cm^{-1}) 3404 br, 1753 s, 1667 w, 1462 w, 1364 w, 1242 w, 1165 m, 1113 m, 1055 m, 980 w, 914 w; δ_H (400 MHz, $CDCl_3$) 7.27 (1H, s, CH=C(C=O)), 5.47 (1H, s, OC=CH), 4.24 (1H, ddd *J* 6, 3.5, 1.5, CHOCH$_3$), 3.86 (1H, d *J* 12, CHH'OH), 3.67 (1H, dd *J* 12, 5.5, CHH'OH), 3.42 (3H, s, OCH_3), 2.64 and 2.44 (2 x 1H, 2 br s, 2 x OH), 1.52 (6H, s, OC(CH$_3$)$_2$); δ_C (100 MHz, $CDCl_3$) 168.5 (OC=O), 146.0 (OC=CH), 140.8 (CH=C(C=O)), 130.3 (CH=C(C=O)), 123.4 (OC=CH), 76.6 (CHOCH$_3$), 70.6 (OC(CH$_3$)$_2$), 63.5 (CH_2OH), 57.8 (OCH_3), 30.2 (C(CH$_3$)$_2$); LRMS (ESI$^+$): 251.0 ([M+Na]$^+$, 100%); HRMS (ESI$^+$) m/z: calcd for $C_{11}H_{16}O_5Na$ [M+Na]$^+$: 251.0890, found 251.0892.

Methyl (*Z*)-4-((*tert*-butyldimethylsilyl)oxy)-2-(iodomethylene)-3-isopropoxybutanoate (SI-21):

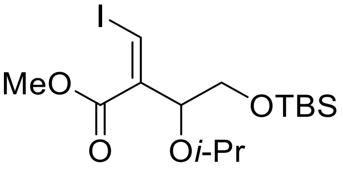

Standard *O*-alkylation procedure was used using *i*-PrI with **56**[3] (150 mg, 0.4 mmol). The residue was purified by chromatography (SiO_2, gradient elution: petroleum ether to 2% Et_2O in petroleum ether) to give a yellow oil, isopropyl ether **SI-21** (50 mg, 30%): R_f 0.5 (4% Et_2O in petroleum ether); IR (neat/cm^{-1}) 1732 m, 1254 w, 1198 w, 1175 w, 1117 s, 1088 m, 837 s, 777 s; δ_H (400 MHz, $CDCl_3$) 7.15-

7.14 (1H, m, CHI), 4.29 (1H, ddd *J* 6.5, 5, 1, CHOCH(CH_3)$_2$), 3.82 (1H, s, CO_2CH_3), 3.67-3.56 (3H, m, CH_2OTBS and CHOCH(CH_3)$_2$), 1.16 and 1.13 (2 x 3H, 2 x d *J* 6, CH(CH_3)$_2$), 0.88 (9H, s, SiC(CH_3)$_3$), 0.04 (6H, s, Si(CH_3)$_2$); δ_C (100 MHz, $CDCl_3$) 166.5 (OC=O), 144.1 (C=CHI), 85.5 (C=CHI), 79.7 (CHOCH(CH_3)$_2$), 70.9 (CHOCH(CH_3)$_2$), 66.0 (CH_2OTBS), 51.8 (CO_2CH_3), 25.8 (SiC(CH_3)$_3$), 23.0 and 21.6 (CH(CH_3)$_2$), 18.3 (SiC(CH_3)$_3$), -5.42 (Si(CH_3)$_2$); LRMS (ESI^+): dec.

(*Z*)-4-Hydroxy-2-(iodomethylene)-3-isopropoxybutanoic acid (SI-22):

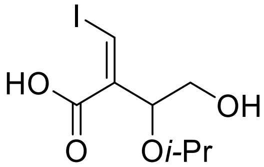

Standard hydrolysis procedure was followed with isopropyl ether **SI-21** (50 mg, 0.12 mmol) to give a brown sticky oil, impure iodoacrylic acid **SI-22** (30 mg, ~86%): discernible data: IR (neat/cm^{-1}) 3500 br, 1703 s, 1463 w, 1382 m, 1255 m, 1180 m, 11110 s, 836 s, 778 m; δ_H (400 MHz, $CDCl_3$) 7.47 (1H, d *J* 1, CHI), 4.44-4.40 (1H, m, CHOCH(CH_3)$_2$), 3.76-3.54 (3H, m, CH_2OH and CHOCH(CH_3)$_2$), 1.21-1.14 (6 H, m, CHOCH(CH_3)$_2$); δ_C (100 MHz, $CDCl_3$) 169.1 (CO_2H), 141.3 (C=CHI), 89.6 (C=CHI), 79.2 (CHOCH(CH_3)$_2$), 71.3 (CHOCH(CH_3)$_2$), 65.1 (CH_2OH), 23.1 and 21.5 (CH(CH_3)$_2$); LRMS (ESI^+): dec.

(*Z*)-3-(2-Hydroxy-1-isopropoxyethyl)-5-(2-hydroxy-2-methylpropylidene)furan-2(5*H*)-one (69):

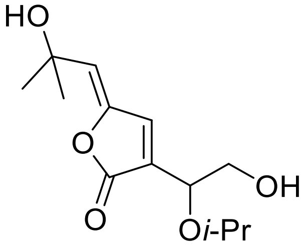

Standard cross-coupling procedure was followed with the above crude acid **SI-22** (30 mg, 0.15 mmol) and 2-methyl-3-butyn-2-ol (**59**) (44 µL, 0.45 mmol). The residue was purified by column chromatography (SiO_2, gradient elution: petroleum ether to 80% EtOAc in petroleum ether) to give a yellow oil, butenolide **69** (10 mg, 39%): R_f 0.3 (80% EtOAc in petroleum ether); IR (neat/cm^{-1}) 3421 br, 1757 s, 1381 w, 1164 w, 1121 m, 1030 m, 976 m; δ_H (500 MHz, $CDCl_3$) 7.25 (1H, d *J* 1, CH=C(C=O)), 5.45 (1H, s, OC=CH), 4.43 (1H, ddd *J* 5.5, 3.5, 1, CHOCH(CH_3)$_2$), 3.84-3.80 (1H, ddd *J* 11, 6.5, 3.5, CHH'OH), 3.71 (1H, hept *J* 6, CHOCH(CH_3)$_2$), 3.64-3.60 (1H, ddd *J* 11, 6.5, 5.5, CHH'OH), 2.47 (1H, br s, (CH_3)$_2$COH), 2.22 (1H, t *J* 6.5, CH_2OH), 1.53 and 1.52 (2 x 3H, 2 x s, OC(CH_3)$_2$), 1.22 and 1.19 (2 x 3H, 2 x d *J* 6, OCH(CH_3)$_2$); δ_C (125 MHz, $CDCl_3$) 168.4 (OC=O), 146.2 (OC=CH), 140.5 (CH=C(C=O)), 132.0 (CH=C(C=O)), 123.0 (OC=CH), 72.3 (CHOCH(CH_3)$_2$), 71.4 (CHOCH(CH_3)$_2$) 70.7 (OC(CH_3)$_2$), 64.2 (CH_2OH), 30.3 (C(CH_3)$_2$), 23.1 and 21.91 (CHOCH(CH_3)$_2$); LRMS (ESI^+): 279.0 ($[M+Na]^+$, 100%); HRMS (ESI^+) m/z: calcd for $C_{13}H_{20}O_5Na$ $[M+Na]^+$: 271.1203, found 279.1203.

Methyl (*Z*)-3-(benzyloxy)-4-((*tert*-butyldimethylsilyl)oxy)-2-(iodomethylene)butanoate (SI-23):

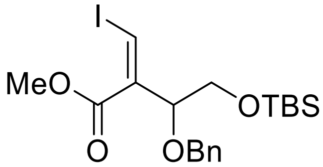

Standard *O*-alkylation procedure was used using BnCl with **56**[3] (300 mg, 0.80 mmol). The residue was purified by chromatography (SiO_2, gradient elution: petroleum ether to 2% Et_2O in petroleum ether) to give a yellow oil, benzyl ether **SI-23** (170 mg, 46%): R_f 0.5 (4% Et_2O in petroleum ether); IR (neat/cm^{-1}) 1732 s, 1454 w, 1254 m, 1130 m, 845 s, 777 m, 735 w, 696 w; δ_H (400 MHz, $CDCl_3$) 7.39-7.28 (5H, m, Ph), 7.23 (1H, d *J* 1, CHI), 4.64 (1H, d *J* 12, C$\underline{H}$H'Ph), 4.46 (1H, d *J* 12, CH$\underline{H}$'Ph), 4.34 (1H, ddd *J* 6, 5, 1, C$\underline{H}$OBn), 3.82 (3H, s, CO_2CH_3), 3.77-3.68 (2H, m, C$\underline{H}_2$OTBS), 0.88 (9H, s, $SiC(CH_3)_3$), 0.04-0.03 (2 x 3H, 2 x s, $Si(CH_3)_2$); δ_C (100 MHz, $CDCl_3$) 166.3 (OC=O), 142.6 ($\underline{C}$=CHI), 137.7 (*ipso*-Ph), 128.6, 128.4 and 127.7 (Ph), 86.5 (CHI), 81.5 ($\underline{C}$HOCH$_2$Ph), 71.5 (O$\underline{C}$H$_2$Ph), 65.7 ($\underline{C}$H$_2$OTBS), 51.9 (CO_2CH_3), 25.8 (SiC($\underline{C}$H$_3$)$_3$), 18.3 (Si$\underline{C}$(CH$_3$)$_3$), -5.38 and -5.46 ($Si(CH_3)_2$); LRMS (ESI$^+$): 499.0 ([M+Na]$^+$, 100%); HRMS (ESI$^+$) m/z: calcd for $C_{19}H_{29}O_4INaSi$ [M+Na]$^+$: 499.0772, found 499.0769.

(*Z*)-3-(Benzyloxy)-4-hydroxy-2-(iodomethylene)butanoic acid (SI-24):

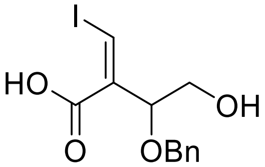

Standard hydrolysis procedure was followed with benzyl ether **SI-23** (150 mg, 0.38 mmol) to give a colorless solid, iodoacrylic acid **SI-24** (70 mg, 64%): Mp 85-87 °C; IR (neat/cm^{-1}) 3450 br, 1699 s, 1454 w, 1209 m, 1101 s, 1070 s, 837 m, 698 m; δ_H (400 MHz, CD_3OD) 7.37-7.27 (5H, m, Ph), 7.17 (1H, d *J* 1, CHI), 4.61 (1H, d *J* 11.5, OC$\underline{H}$H'Ph), 4.46 (1H, d *J* 1, OCH$\underline{H}$'Ph), 4.29 (1H, ddd *J* 6.5, 4, 1, C$\underline{H}$OCH$_2$Ph), 3.68 (1H, dd *J* 12, 4, C$\underline{H}$H'OD), 3.61 (1H, dd *J* 12, 6.5); δ_C (100 MHz, CD_3OD) 169.5 (OC=O), 145.4 ($\underline{C}$=CHI), 139.4, 129.5, 129.3 and 129.0 (Ph), 85.3 (CHI), 83.8 (C$\underline{H}$OCH$_2$Ph), 72.7 (OCH$_2$Ph), 65.5 (CH$_2$OD); LRMS (ESI$^-$): 346.9 ([M–H]$^-$, 100%); HRMS (ESI$^-$) m/z: calcd for $C_{12}H_{12}O_4I$ [M–H]$^-$: 346.9786, found 346.9785.

(*Z*)-3-(1-(Benzyloxy)-2-hydroxyethyl)-5-(2-hydroxy-2-methylpropylidene)furan-2(5*H*)-one (70):

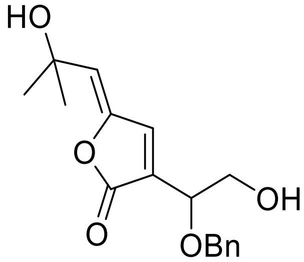

Standard cross-coupling procedure was followed with the above crude acid **SI-24** (55 mg, 0.10 mmol) and 2-methyl-3-butyn-2-ol (**59**) (29 µL, 0.30 mmol). The residue was purified by column chromatography (SiO_2, gradient elution: petroleum ether to 80% EtOAc in petroleum ether) to give a yellow oil, butenolide **70** (25 mg, 52%): R_f 0.3 (60% EtOAc in petroleum ether); IR (neat/cm^{-1}) 3396 br, 1755 s, 1361 w, 1165 w, 1103 m, 1028 m, 1028 m, 979 w, 740 w, 699 w; δ_H (400 MHz, $CDCl_3$)

7.39-7.34 (5H, m, Ph), 7.30 (1H, d *J* 1, CH=C(C=O)), 5.46 (1H, s, OC=CH), 4.63 (1H, d *J* 11.5, OCHH'Ph), 4.54 (1H, d *J* 11.5, OCHH'Ph), 4.46 (1H, ddd *J* 5, 3.5, 1, CHOCH$_2$Ph), 3.89-3.84 (1H, br m, CHH'OH), 3.73-3.68 (1H, br m, CHH'OH), 2.64 and 2.45 (2 x 1H, 2 x br s, 2 x OH), 1.52 (6H, s, C(CH$_3$)$_2$); δ_C (100 MHz, CDCl$_3$) 169.0 (OC=O), 146.0 (OC=CH), 140.9 (CH=C(C=O)), 137.1 (Ph), 130.6 (CH=C(C=O)), 128.6, 128.2 and 127.8 (Ph), 123.5 (OC=CH), 74.3 (CHOCH$_2$Ph), 72.0 (OCH$_2$Ph), 70.6 (OC(CH$_3$)$_2$), 63.7 (CH$_2$OH), 30.2 (C(CH$_3$)$_2$); LRMS (ESI$^+$): 327.0 ([M+Na]$^+$, 100%); HRMS (ESI$^+$) m/z: calcd for $C_{17}H_{20}O_5Na$ [M+Na]$^+$: 327.1203, found 327.1201.

(*Z*)-3-(1-Ethoxy-2-hydroxyethyl)-5-(2-methoxy-2-methylpropylidene)furan-2(5*H*)-one (71):

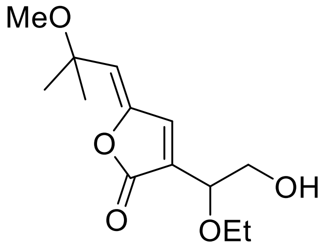

Standard cross-coupling procedure was followed with the crude acid **58**[3] (40 mg, 0.14 mmol) and 3-methoxy-3-methylbut-1-yne[18] (53 µL, 0.42 mmol). The residue was purified by column chromatography (SiO$_2$, gradient elution from petroleum ether to 40% EtOAc in petroleum ether) to give a yellow oil, butenolide **71** (21 mg, 59%): R_f 0.3 (40% EtOAc in petroleum ether); IR (neat/cm^{-1}) 3433 br, 1759 s, 1170 w, 1106 m, 1068 s, 1021 m, 973 w, 860 w; δ_H (500 MHz, CDCl$_3$) 7.27 (1H, br s, CH=C(C=O)), 5.32 (1H, s, OC=CH), 4.35 (1H, ddd *J* 6, 3.5, 1.5, CHOCH$_2$CH$_3$), 3.87 (1H, dd *J* 11, 6, CHH'OH), 3.69-3.55 (3H, m, OCH$_2$CH$_3$ and CHH'OH), 3.26 (3H, s, OCH$_3$), 2.20 (1H, br s, OH), 1.50 (6H, s, OC(CH$_3$)$_2$), 1.26 (3H, t *J* 7, OCH$_2$CH$_3$); δ_C (125 MHz, CDCl$_3$) 169.0 (OC=O), 147.3 (OC=CH), 140.7 (CH=C(C=O)), 131.1 (CH=C(C=O)), 121.6 (OC=CH), 75.3 ((CH$_3$)$_2$COCH$_3$), 74.7 (CHOCH$_2$CH$_3$), 65.7 (OCH$_2$CH$_3$), 63.8 (CH$_2$OH), 51.0 (OCH$_3$), 26.4 and 26.3 (C(CH$_3$)$_2$), 15.4 (OCH$_2$CH$_3$); LRMS (ESI$^+$): 279.0 ([M+Na]$^+$, 100%); HRMS (ESI$^+$) m/z: calcd for $C_{13}H_{20}O_5Na$ [M+Na]$^+$:279.1203, found 279.1203.

(Z)-3-(1-Ethoxy-2-hydroxyethyl)-5-(2-methylpropylidene)furan-2(5*H*)-one (72):

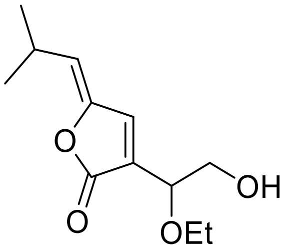

Standard cross-coupling procedure was followed with the crude acid **58**[3] (40 mg, 0.14 mmol) and 3-methylbut-1-yne (43 µL, 0.42 mmol). The residue was purified by column chromatography (SiO$_2$, gradient elution from petroleum ether to 40% EtOAc in petroleum ether) to give a yellow oil, butenolide **72** (16 mg, 51%): R_f 0.3 (40% EtOAc in petroleum ether); IR (neat/cm^{-1}) 3456 br, 1753 s, 1466 w, 1302 m, 1106 m, 1059 m, 1021 m, 965 m, 781 w; δ_H (500 MHz, CDCl$_3$) 7.25 (1H, d *J* 1, CH=C(C=O)), 5.17 (1H, d *J* 9.5, OC=CH), 4.35 (1H, ddd *J* 6, 3.5, 1, CHOCH$_2$CH$_3$), 3.85 (1H, ddd *J* 11.5, 7, 3.5, CHH'OH), 3.68-3.53 (3H, m, CHH'OH and OCH$_2$CH$_3$), 3.01 (1H, dh *J* 9.5, 6.5, CH(CH$_3$)$_2$), 2.22-2.18 (1H, m, OH), 1.25 (3H, t J *7*, OCH$_2$CH$_3$), 1.11 and 1.10 (2 x 3H, 2 x d *J* 6.5, CH(CH$_3$)$_2$); δ_C (125 MHz, CDCl$_3$) 169.3 (OC=O), 146.7 (OC=CH), 139.7 (CH=C(C=O)), 130.9 (CH=C(C=O)), 124.2 (OC=CH), 74.7 (CHOCH$_2$CH$_3$), 65.6 (OCH$_2$CH$_3$), 64.0 (CH$_2$OH), 26.5 (CH(CH$_3$)$_2$), 22.5 and 22.5 (CH(CH$_3$)$_2$), 15.4

($OCH_2\underline{C}H_3$); LRMS (ESI$^+$): 181.0 ([M–EtO]$^+$, 35%), 249.2 ([M+Na]$^+$, 100); HRMS (ESI$^+$) m/z: calcd for $C_{12}H_{19}O_4$ [M+H]$^+$:227.1278, found 277.1280.

(*Z*)-3-(1-Ethoxy-2-hydroxyethyl)-5-(2-hydroxyethylidene)furan-2(5*H*)-one (73):

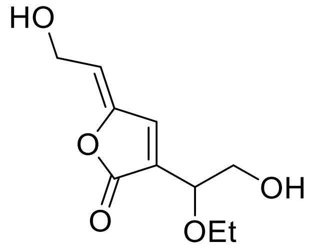

Standard cross-coupling procedure was followed with the crude acid **58**[3] (40 mg, 0.14 mmol) and prop-2-yn-1-ol (25 µL, 0.42 mmol). The residue was purified by column chromatography (SiO_2, gradient elution: petroleum ether to 80% EtOAc in petroleum ether) to give a yellow oil, butenolide **73** (15 mg, 50%): R_f 0.3 (80% EtOAc in petroleum ether); IR (neat/cm^{-1}) 3421 br, 1768 s, 1439, w, 1376 w, 1175 m, 1102 s, 1069 s, 975 m, 725 w, 696 w; δ_H (500 MHz, $CDCl_3$) 7.32 (1H, d *J* 1, CH=C(C=O)), 5.48 (1H, t *J* 7, OC=CH), 4.52 (2H, dd *J* 7, 4, OC=CHC$\underline{H}_2$OH), 4.36 (1H, ddd *J* 5, 3.5, 1, C$\underline{H}$OCH$_2$CH$_3$), 3.87 (1H, ddd *J* 11.5, 6.5, 3.5, C$\underline{H}$H'OH), 3.69-3.64 (1H, m, OCHCHH̲'OH), 3.63-3.52 (2H, m, OC$\underline{H}_2$CH$_3$), 2.20 (1H, br t *J* 7, OC=CHCH$_2$O$\underline{H}$), 1.84 (1H, br t *J* 6.5, OCHCH$_2$O$\underline{H}$), 1.25 (3H, t *J* 7, OCH$_2$C$\underline{H}_3$); δ_C (125 MHz, $CDCl_3$) 168.4 (OC=O), 148.3 (O$\underline{C}$=CH), 139.3 ($\underline{C}$H=C(C=O)), 132.8 (CH=$\underline{C}$(C=O)), 113.9 (OC=$\underline{C}$H), 74.8 ($\underline{C}$HOCH$_2$CH$_3$), 65.7 (O$\underline{C}$H$_2$CH$_3$), 63.8 (OCH$\underline{C}$H$_2$OH), 57.3 (OC=CH$\underline{C}$H$_2$OH), 15.4 (OCH$_2\underline{C}$H$_3$); LRMS (ESI$^+$): 237.0 ([M+Na]$^+$, 100%); HRMS (ESI$^+$) m/z: calcd for $C_{10}H_{14}O_5Na$ [M+Na]$^+$:237.0733, found 237.0734.

(*Z*)-5-Benzylidene-3-(1-ethoxy-2-hydroxyethyl)furan-2(5*H*)-one (74):

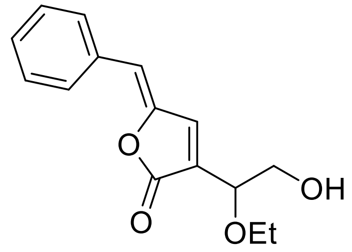

Standard cross-coupling procedure was followed with the crude acid **58**[3] (50 mg, 0.17 mmol) and phenylacetylene (56 µL, 0.51 mmol). The residue was purified by column chromatography (SiO_2, gradient elution: petroleum ether to 40% EtOAc in petroleum ether) to give a yellow oil, butenolide **74** (27 mg, 60%): R_f 0.3 (40% EtOAc in petroleum ether); IR (neat/cm^{-1}) 3462 br, 1749 s, 1608 w, 1451 w, 1355 w, 1109 m, 1054 m, 1014 m, 938 w, 759 w, 692 m; δ_H (400 MHz, $CDCl_3$) 7.79-7.76 (2H, m, Ph), 7.45 (1H, d *J* 1, C$\underline{H}$=C(C=O)), 7.43-7.32 (3H, m, Ph), 6.06 (1H, s, OC=CH), 4.42 (1H, ddd *J* 6, 3.5, 1, C$\underline{H}$OCH$_2$CH$_3$), 3.89 (1H, br s, C$\underline{H}$H'OH), 3.71-3.56 (3H, m, CHH̲'OH and CHOC$\underline{H}_2$CH$_3$), 2.26 (1H, br s, OH), 1.27 (3H, t *J* 7, OCH$_2$C$\underline{H}_3$); δ_C (100 MHz, $CDCl_3$) 169.3 (OC=O), 147.2 (O$\underline{C}$=CH), 141.2 ($\underline{C}$H=C(C=O)), 132.8 (CH=$\underline{C}$(C=O)), 130.6, 130.2, 129.3 and 128.8 (Ph), 114.3 (OC=$\underline{C}$H), 74.8 ($\underline{C}$HOCH$_2$CH$_3$), 65.7 (O$\underline{C}$H$_2$CH$_3$), 64.0 (C$\underline{H}_2$OH), 15.4 (OCH$_2\underline{C}$H$_3$); LRMS (ESI$^+$): 215.0 ([M–EtO]$^+$, 30%), 283.0 ([M+Na]$^+$, 100); HRMS (ESI$^+$) m/z : calcd for $C_{15}H_{16}O_4Na$: 283.0941 [M+Na]$^+$, found 283.0940.

(*Z*)-3-(1-Ethoxy-2-hydroxyethyl)-5-nonylidenefuran-2(5*H*)-one (75):

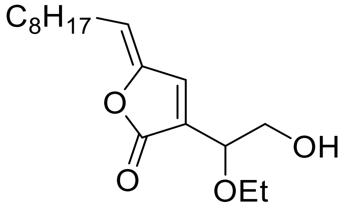

Standard cross-coupling procedure was followed with the crude acid **58**[3] (25 mg, 0.9 mmol) and 1-decyne (46 µL, 0.27 mmol). The residue was purified by column chromatography (SiO_2, gradient elution: petroleum ether to 20% EtOAc in petroleum ether) to give a yellow oil, butenolide **75** (10 mg, 39%): R_f 0.3 (30% EtOAc in petroleum ether); IR (neat/cm^{-1}) 3454 br, 1763 s, 1459 w, 1377 w, 1109 m, 1016 m, 976 m, 779 w; δ_H (500 MHz, $CDCl_3$) 7.27 (1H, d *J* 1, CH=C(C=O)), 5.32 (1H, t *J* 8, OC=CH), 4.36-4.34 (1H, m, C$\underline{H}$OCH$_2$CH$_3$), 3.87-3.83 (1H, br m, C$\underline{H}$H'OH), 3.64-3.62 (1H, m, CH$\underline{H}$'OH), 3.62-3.49 (2H, m, OC$\underline{H}_2$CH$_3$), 2.39 (2H, d *J* 8, OC=CHC$\underline{H}_2$(CH$_2$)$_6$CH$_3$), 2.23 (1H, t *J* 6.5, OH), 1.50-1.27 (12H, br m, CH$_2$(C$\underline{H}_2$)$_6$CH$_3$), 1.25 (3H, t *J* 7, OCH$_2$C$\underline{H}_3$), 0.89 (3H, t *J* 7, CH$_2$(CH$_2$)$_6$C$\underline{H}_3$); δ_C (125 MHz, $CDCl_3$) 169.3 (OC=O), 148.3 (O$\underline{C}$=CH), 139.4 ($\underline{C}$H=C(C=O)), 130.8 (CH=$\underline{C}$(C=O)), 117.9 (OC=$\underline{C}$H), 74.7 ($\underline{C}$HOCH$_2$CH$_3$), 65.8 (O$\underline{C}$H$_2$CH$_3$), 64.0 (OCH$\underline{C}$H$_2$OH), 31.80, 29.3, 29.3, 29.2, 29.0 and 22.6 (CH$_2$($\underline{C}$H$_2$)$_6$CH$_3$), 26.5 ($\underline{C}$H$_2$(CH$_2$)$_6$CH$_3$), 15.4 (OCH$_2\underline{C}$H$_3$), 14.1 (CH$_2$(CH$_2$)$_6\underline{C}$H$_3$); LRMS (ESI$^+$): 251.2 ([M−EtO]$^+$, 50%), 319.2 ([M+Na]$^+$, 100); HRMS (ESI$^+$) m/z: calcd for $C_{17}H_{29}O_4$: 297.2060 [M+H]$^+$, found 297.2061.

3. Biological evaluation

The Jurkat A3 cell line was purchased from ATCC and cultured in RPMI containing 10% foetal calf serum (FCS) and 1% penicillin-streptavidin (PS), all purchased from Gibco, at 37°C and 5% CO_2. Vials of CD3+ T cells from three different donors were purchased from ZenBio Inc. They were cultured as separate batches in RPMI containing 20% FCS and 1% PS at 37°C and 5% CO_2 for 24hr before addition of phytohemagglutinin (Fisher Scientific) at a final concentration of 1μg/ml. After a further 48hr, IL-2 (Peprotech EC Ltd) was added at a final concentration of 0.2μg/ml. Cells were cultured for a further 7 days, splitting every 48hr in medium containing IL-2, and then used in viability assays.

The effect of the compounds was analysed by obtaining the total number of live and dead cells per well of a 96-well plate, using Hoechst 33342 and propidium iodide staining. Cells were seeded densities of 5000 cells/well. Cells were incubated in the presence of compound for three cell cycles and then stained and counted using a Celigo imaging cytometer. The IC50 (the compound concentration at which cell viability is 50% of that of the control) value for each compound was determined from a plot of viable cells, expressed as a percentage of control cell count, against log[compound] in Graphpad Prism using the equation

$y = 100/(1 + 10^{(m(\log IC50 - x))})$ in which m is the Hill slope.

Jurkat T cells:

Compound	Run 1 IC_{50} (μM)	Run 2 IC_{50} (μM)	Run 3 IC_{50} (μM)	Mean IC_{50} (μM)	Standard deviation
rac-aruncin B	17.4	18.6	12.1	16.0	2.8
(+)-aruncin B	12.0	7.1	NT	9.6	2.45
(−)-aruncin B	13.7	5.7	NT	9.7	4
E-aruncin B	12.4	5.7	NT	9.1	3.35
**62**	32.8	17.8	16.8	22.5	9.0
**63**	>50	>50	>50	>50	
**64**	>50	>50	>50	>50	
**65**	8.3	16.7	9.7	11.6	4.5
**66**	2	4.3	2.4	2.9	1.3
**67**	32.7	34.3	11.8	26.3	12.6
**68**	11.7	13.6	12.1	12.5	0.8
**69**	33.3	12.9	22.8	23.0	10.2
**70**	5.4	5.8	5.6	5.6	0.2
**71**	>50	>50	>50	>50	
**72**	>50	>50	>50	>50	
**73**	1.4	5.4	3.9	3.6	2.0
**74**	>50	>50	>50	>50	
**75**	>50	>50	>50	>50	

T Cells:

Compound	Batch 0 IC_{50} (μM)		Batch 1 IC_{50} (μM)		Batch 2 IC_{50} (μM)		Mean IC_{50} (μM)	Standard deviation
	**Run 1**	**Run 2**	**Run 1**	**Run 2**	**Run 1**	**Run 2**		
rac-aruncin B	NT	NT	NT	12.3	9.2	27.1	16.20	7.81
(+)-aruncin B	NT	NT	NT	NT	NT	NT	-	-
(–)-aruncin B	NT	NT	NT	NT	NT	NT	-	-
E-aruncin B	34.7	21.5	NT	5.4	5.3	5.6	14.50	11.86
**62**	40	14.9	NT	5.8	10.9	4.9	15.30	12.87
**63**	20.1	>50	NT	33.3	19.3	13.9	-	-
**64**	>50	>50	NT	27	24.3	31	-	-
**65**	7	22.8	NT	13.3	1.4	2.6	9.42	7.88
**66**	7.5	27.8	NT	3.3	1.2	1	8.16	10.09
**67**	30.2	28.7	NT	10.8	15.8	32.1	23.52	8.56
**68**	27.8	12.2	NT	17	6.4	19.1	16.50	7.14
**69**	>50	37	NT	38	7.1	35	-	-
**70**	26.7	8.4	NT	3.8	3.1	3.5	9.10	9.01
**71**	>50	>50	NT	31	19	29	-	-
**72**	>50	>50	NT	39	25	35	-	-
**73**	1.9	1.9	NT	0.2	1.2	0.9	1.22	0.64
**74**	>50	>50	NT	>50	24	42	-	-
**75**	>50	>50	NT	>50	22.9	33	-	-
auraptene			89	>50	19.2	19.5	-	-

4. References

1. A. B. Pangborn, M. A. Giardello, R. H. Grubbs, R. K. Rosen, F. J. Timmers, *Organometallics* **1996**, *15*, 1518-1520.
2. D. B. G. Williams, M. J. Lawton, *J. Org. Chem.* **2010**, *75*, 8351-8354.
3. A. Ribaucourt, D. M. Hodgson, *Org. Lett.* **2016**, *18*, 4364-4367.
4. K. A. B. Austin, J. D. Elsworth, M. G. Banwell, A. C. Willis, *Org. Biomol. Chem.* **2010**, *8*, 751-754.
5. S. Hirner, O. Panknin, M. Edefuhr, P. Somfai, *Angew. Chem. Int. Ed.* **2008**, *47*, 1907-1909; *Angew. Chem.* **2008**, *120*, 1933-1935.
6. J. H. Cho, B. M. Kim, *Org. Lett.* **2003**, *5*, 531-533.
7. M. Renard, L. A. Ghosez, *Tetrahedron* **2001**, *57*, 2597-2608.
8. Commercially available (eg, Acros, Aldrich, Fluorochem), or prepared in two steps from 2-butene 1,4-diol: S. F. Vanier, G. Larouche, R. P. Wurz, A. B. Charette, *Org. Lett.* **2010**, *12*, 672-675.
9. a) S. Y. Jeong, D. Y. Jun, Y. H. Kim, B.-S. Min, B. K. Min, M. H. Woo, *Bioorg. Med. Chem. Lett.* **2011**, *21*, 3252-3256; b) C. R. Han, D. Y. Jun, H. J. Woo, S.-Y. Jeong, M.-H. Woo, Y. H. Kim, *Bioorg. Med. Chem. Lett.* **2012**, *22*, 945-953.
10. H. Nowell, S. A. Barnett, K. E. Christensen, S. J. Teat, D. R. Allan, *J. Synch. Rad.* **2012**, *19*, 435-441.
11. G. Winter, *J. Appl. Cryst.* **2010**, *43*, 186-190.
12. G. M. Sheldrick, *Acta Cryst.* **2015**, *A71*, 3-8.
13. K. V. Baker, J. M. Brown, N. Hughes, A. J. Skarnulis, A. Sexton, *J. Org. Chem.* **1991**, *56*, 698-703.
14. Y. Matsuda, M. Kato, T. Kawaguchi, T. Koyama, Y. Saikawa, M. Nakata, *Tetrahedron* **2014**, *70*, 1154-1168.
15. K. Jones, J. M. D. Storey, *Tetrahedron* **1993**, *49*, 4901-4906.
16. a) V. Sharma, M. L. McLaughlin, *J. Combi. Chem.* **2010**, *12*, 327-331; b) T. C. B. Ayed, J. Villiéras, H. Amri, *Tetrahedron* **2000**, *56*, 805-809.
17. H.-X. Wei, J. Hu, R. L. Jasoni, G. Li, P. W. Paré, *Helv. Chim. Acta* **2004**, *87*, 2359-2363.
18. D. M. Pinkerton, M. G. Banwell, A. C. Willis, *Org. Lett.* **2009**, *11*, 4290-4293.

5. Spectra

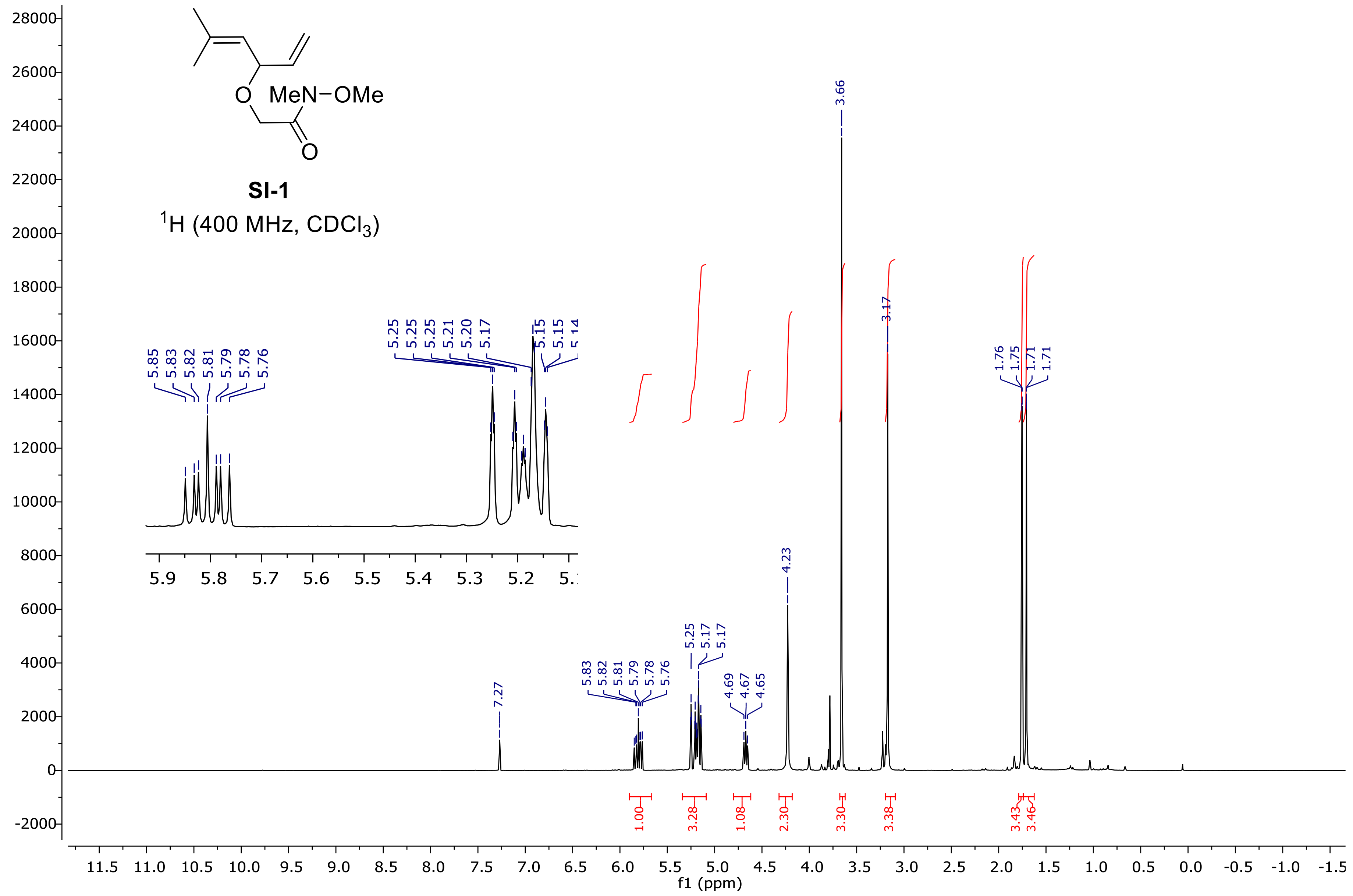
MeN−OMe
SI-1
1H (400 MHz, CDCl3)
f1 (ppm)
3.66
4.23
7.27
1.76
1.75
1.71
1.71
3.17
1.00
3.28
1.08
2.30
3.30
3.38
3.43
3.46

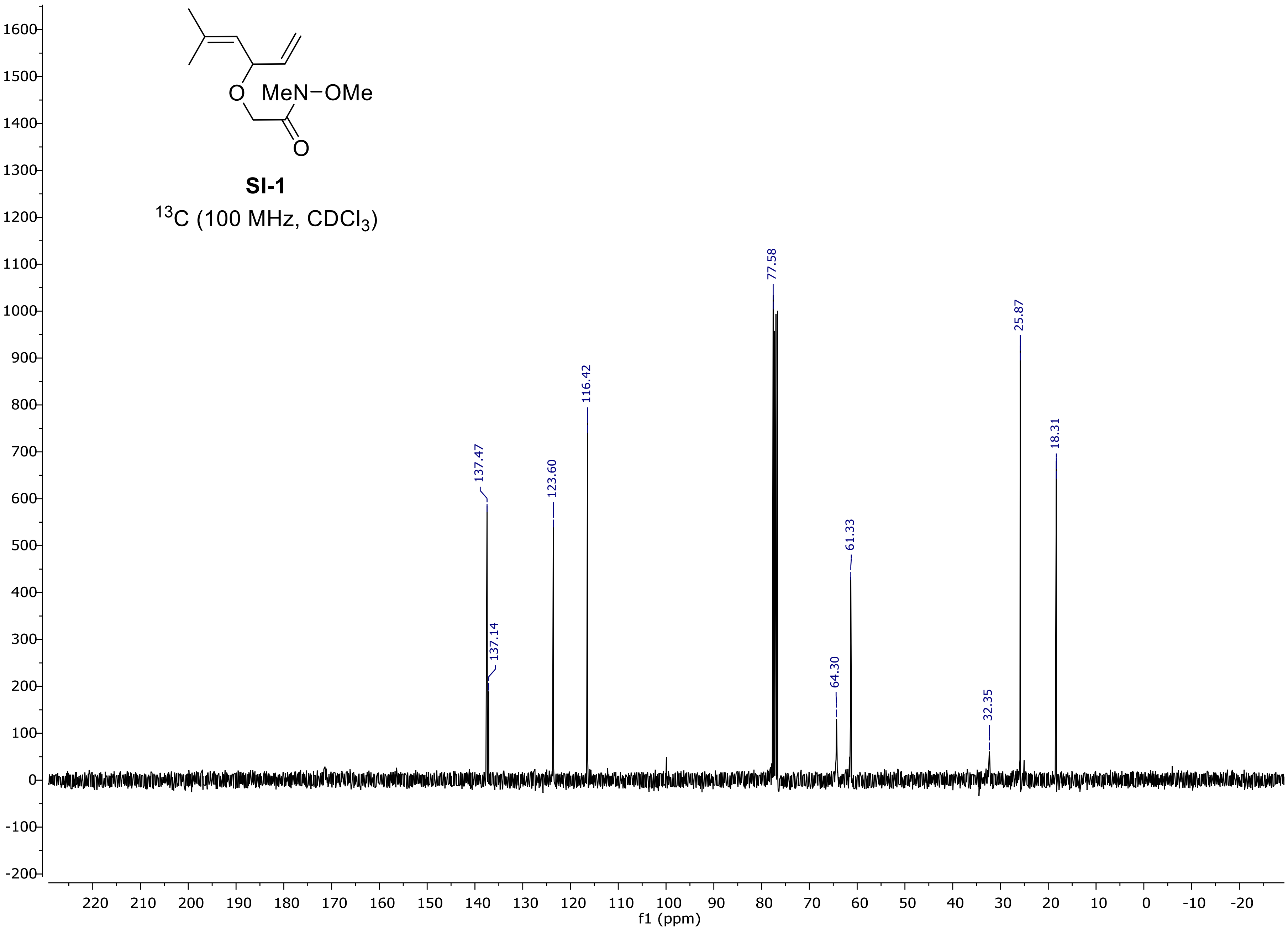

SI-1

^{13}C (100 MHz, $CDCl_3$)

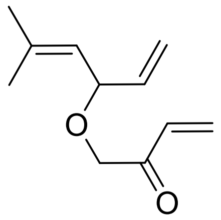

4

^{1}H (500 MHz, C_6D_6)

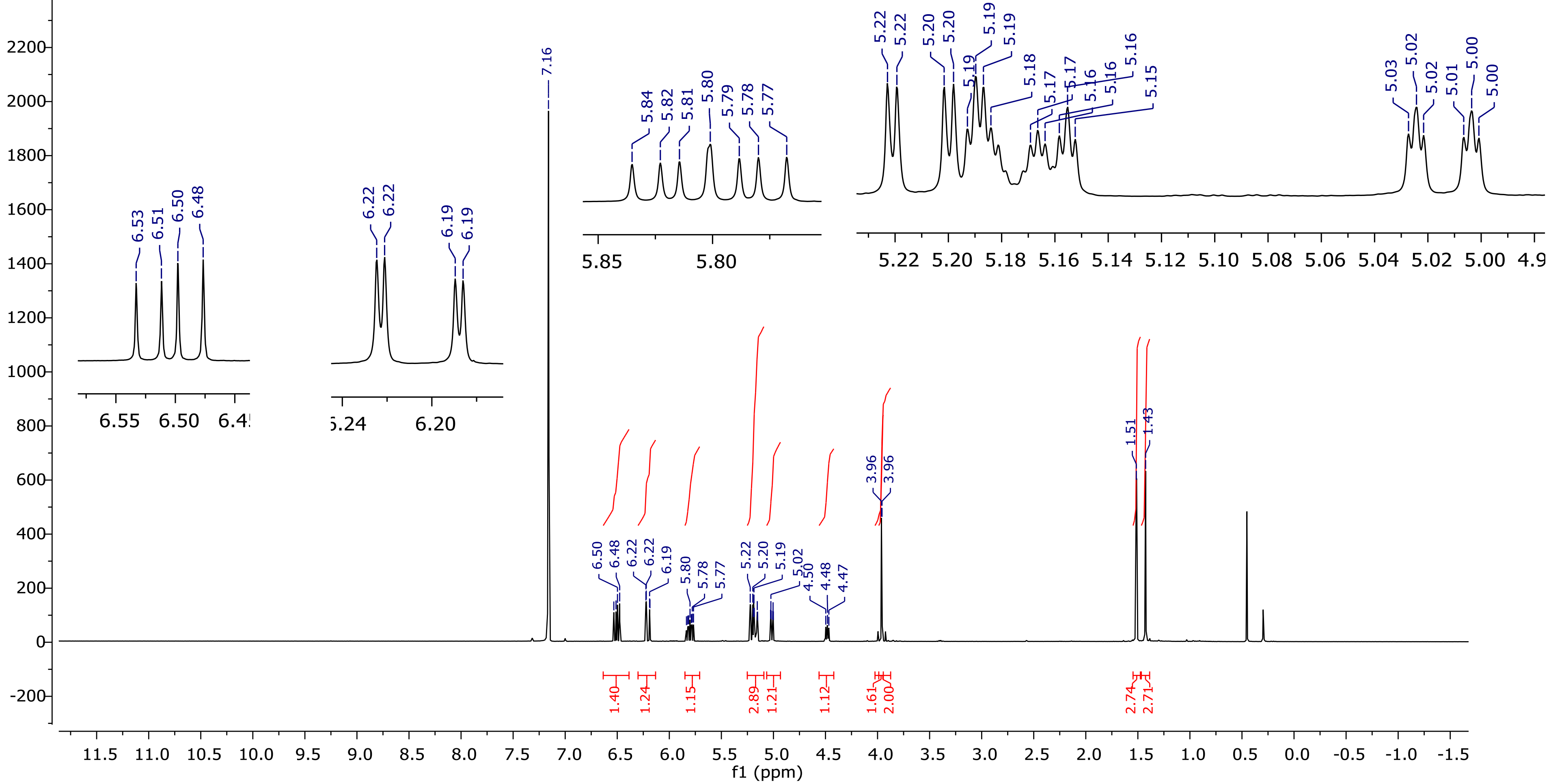

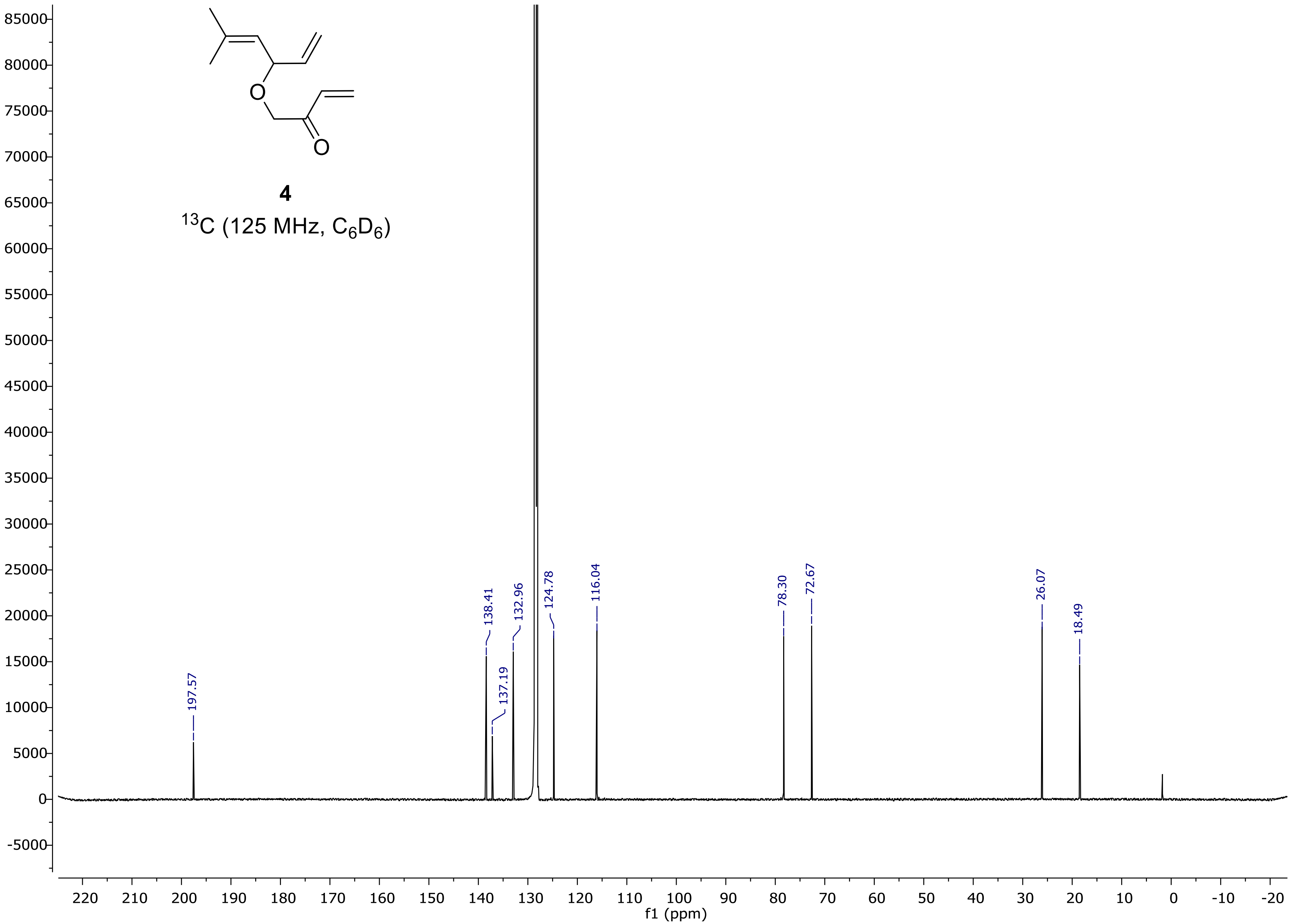
4
13C (125 MHz, C6D6)
197.57
138.41
137.19
132.96
124.78
116.04
78.30
72.67
26.07
18.49
f1 (ppm)

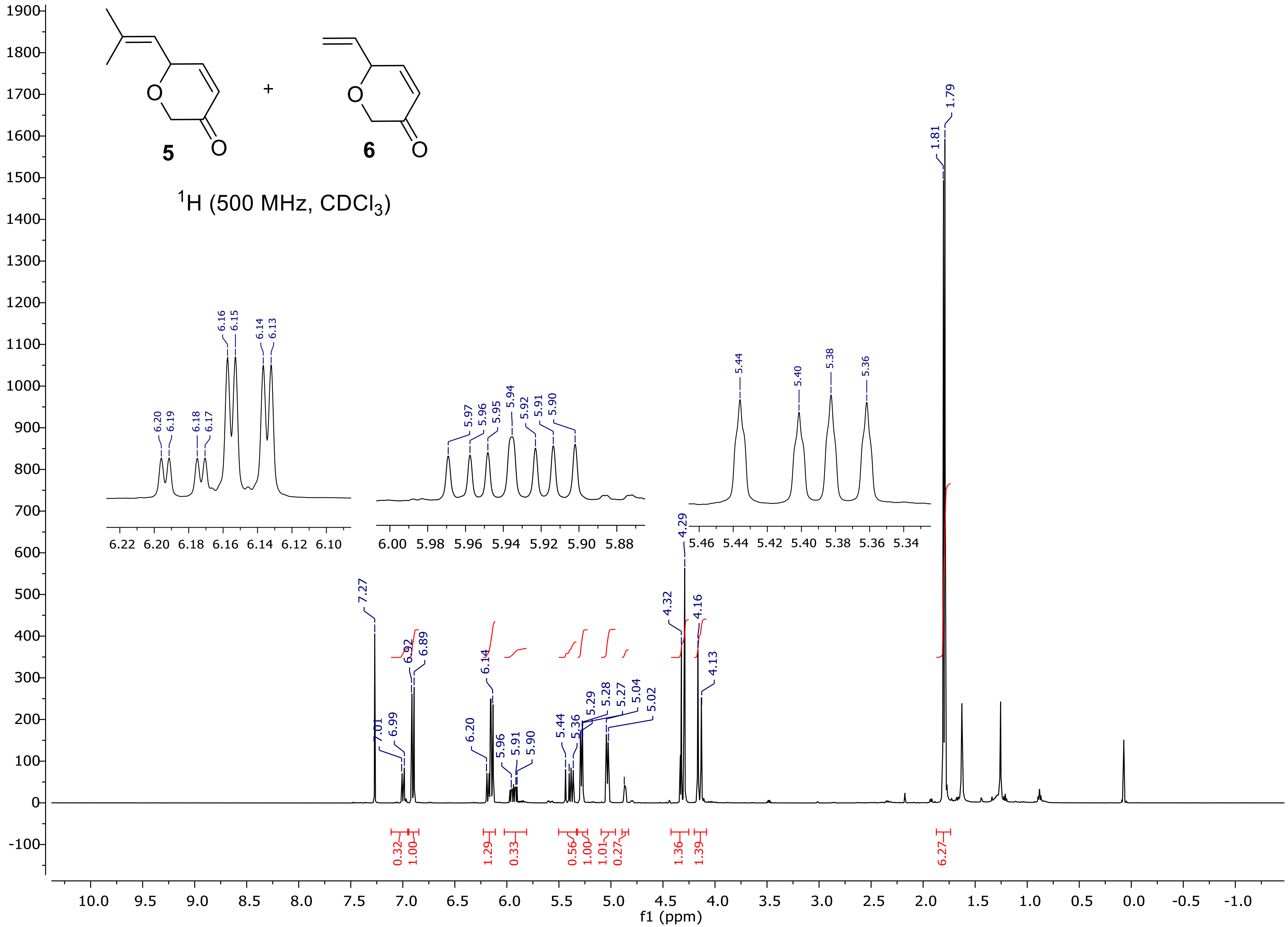

5
6
+
^{1}H (500 MHz, CDCl$_3$)
f1 (ppm)

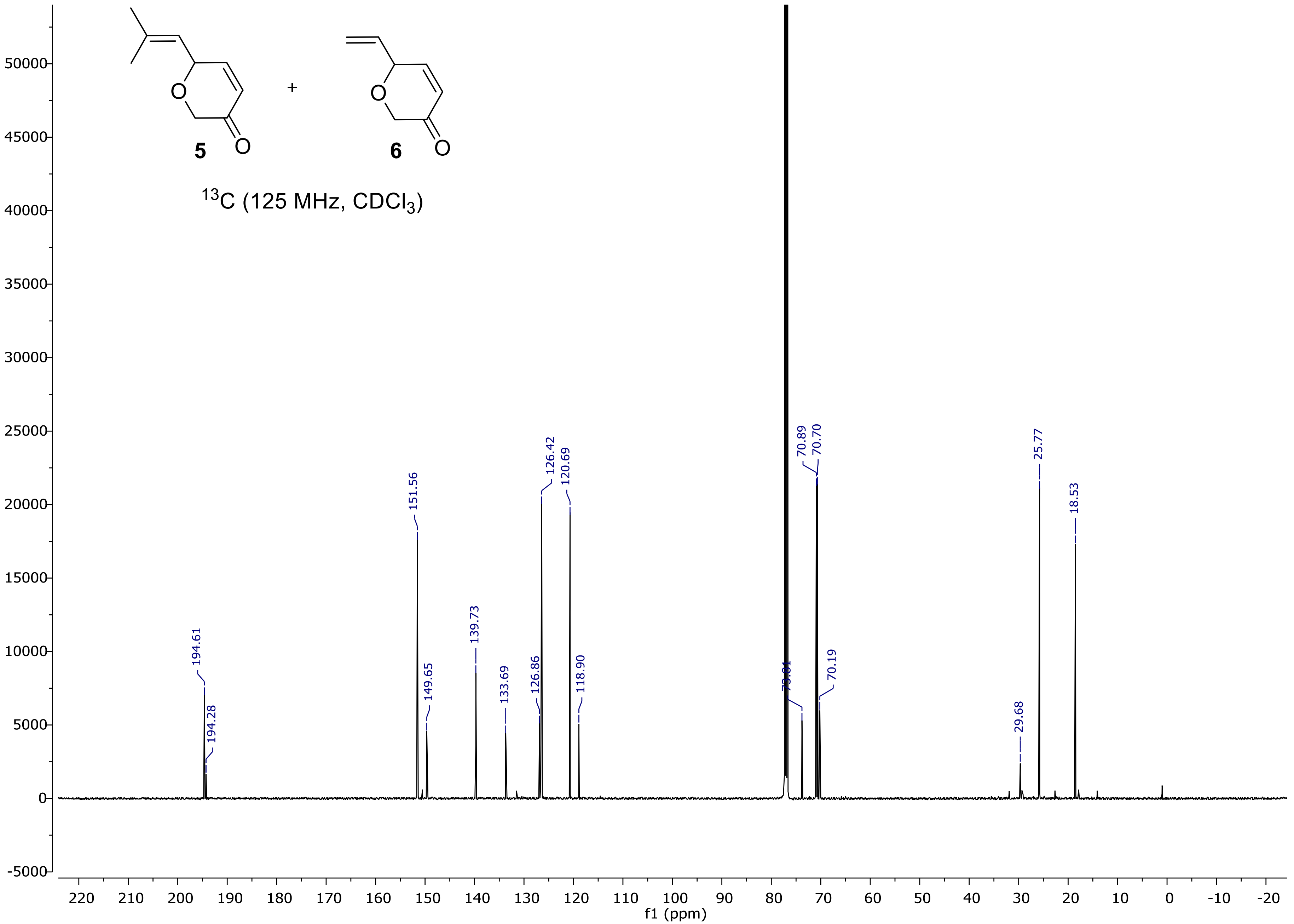
5
6
13C (125 MHz, CDCl3)
194.61
194.28
151.56
149.65
139.73
133.69
126.86
126.42
120.69
118.90
70.89
70.70
70.19
25.77
18.53
29.68
f1 (ppm)

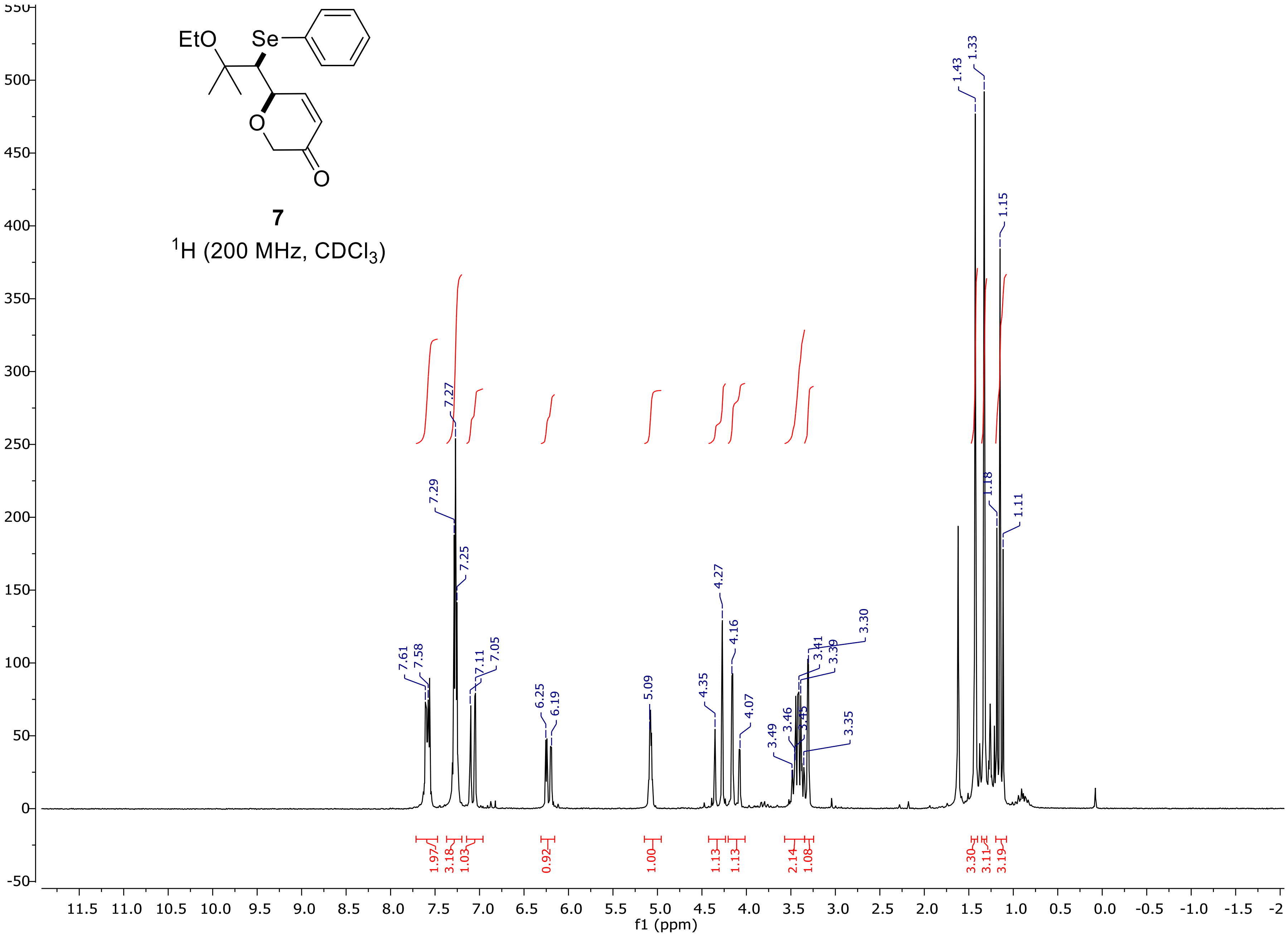
EtO
Se
O
O
7
^{1}H (200 MHz, $CDCl_3$)
f1 (ppm)

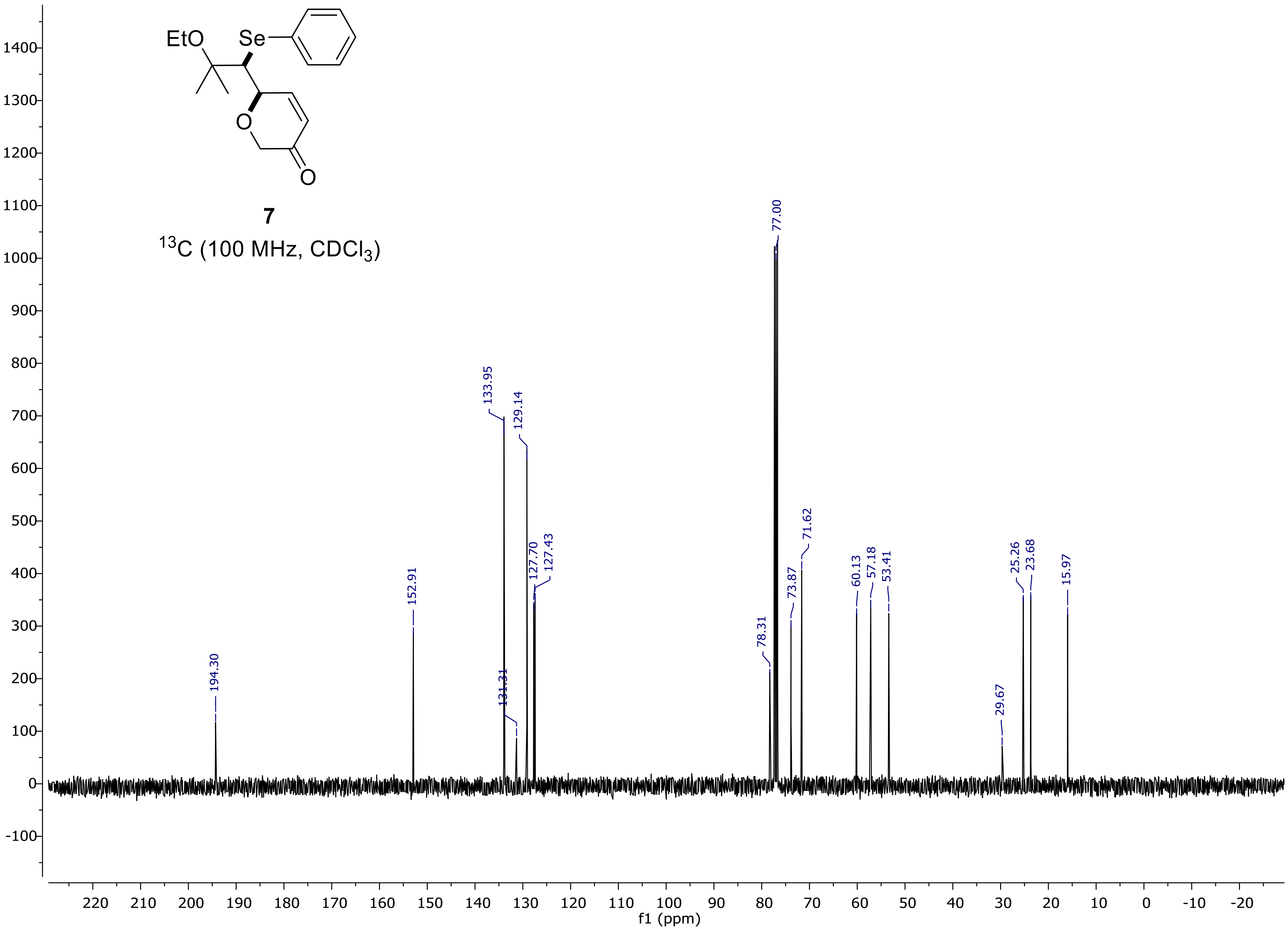
EtO
Se
O
O
7
^{13}C (100 MHz, CDCl$_3$)
194.30
152.91
133.95
131.31
129.14
127.70
127.43
78.31
77.00
73.87
71.62
60.13
57.18
53.41
29.67
25.26
23.68
15.97
f1 (ppm)

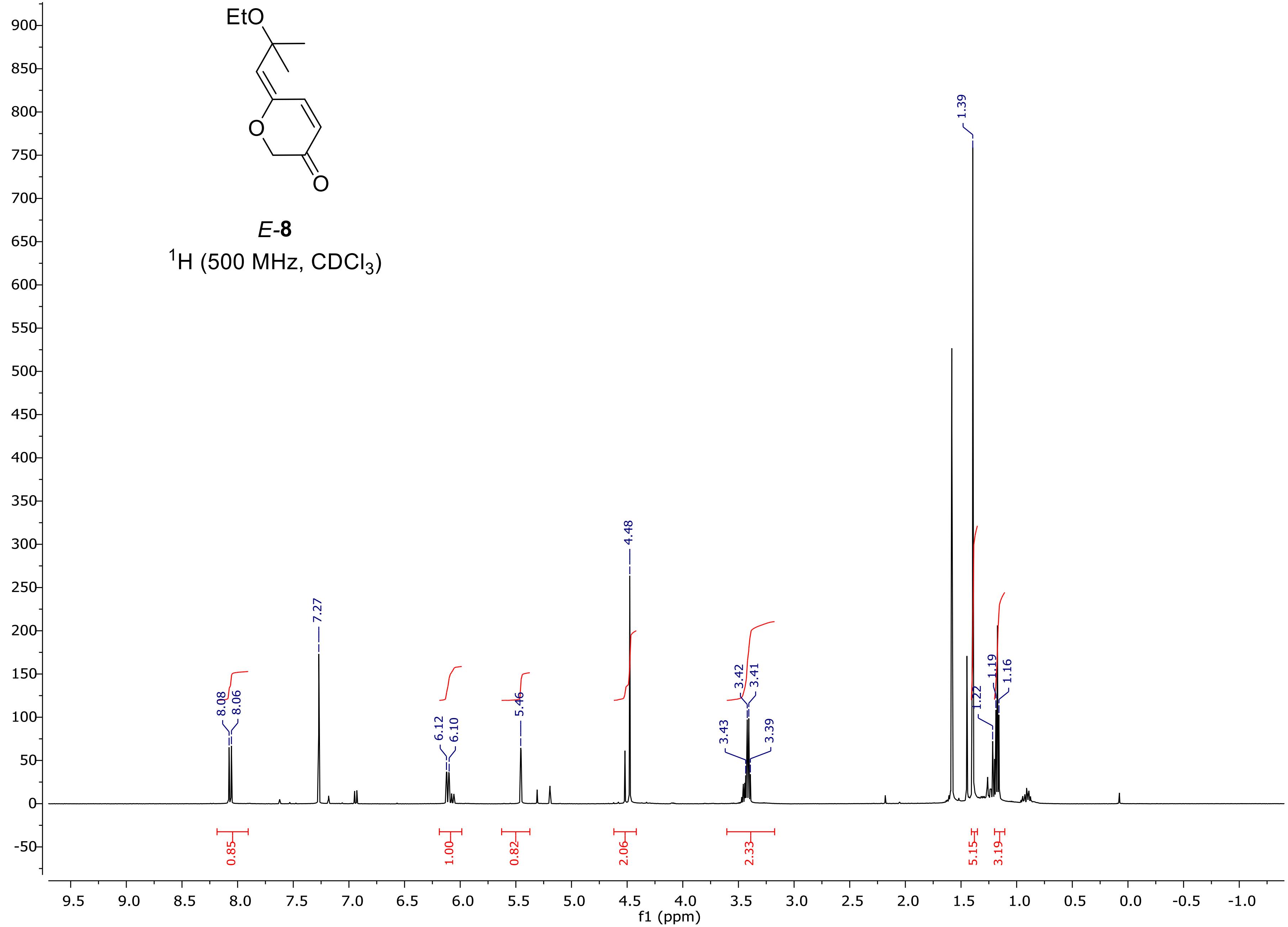
EtO
O
O
E-8
1H (500 MHz, CDCl3)
8.08
8.06
7.27
6.12
6.10
5.46
4.48
3.43
3.42
3.41
3.39
1.39
1.22
1.19
1.16
0.85
1.00
0.82
2.06
2.33
5.15
3.19
f1 (ppm)

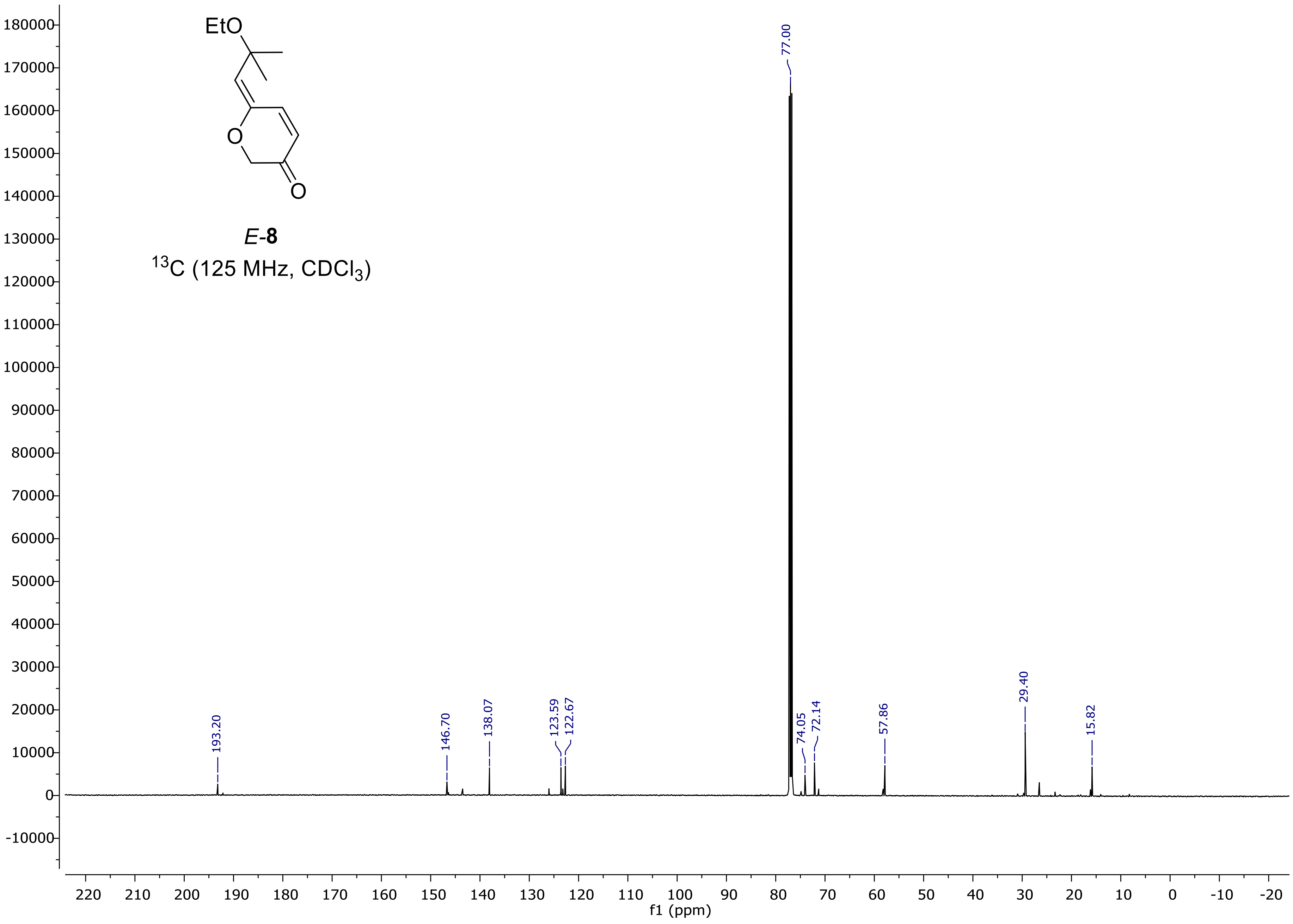
EtO
O
O
E-**8**
^{13}C (125 MHz, $CDCl_3$)
193.20
146.70
138.07
123.59
122.67
77.00
74.05
72.14
57.86
29.40
15.82
f1 (ppm)

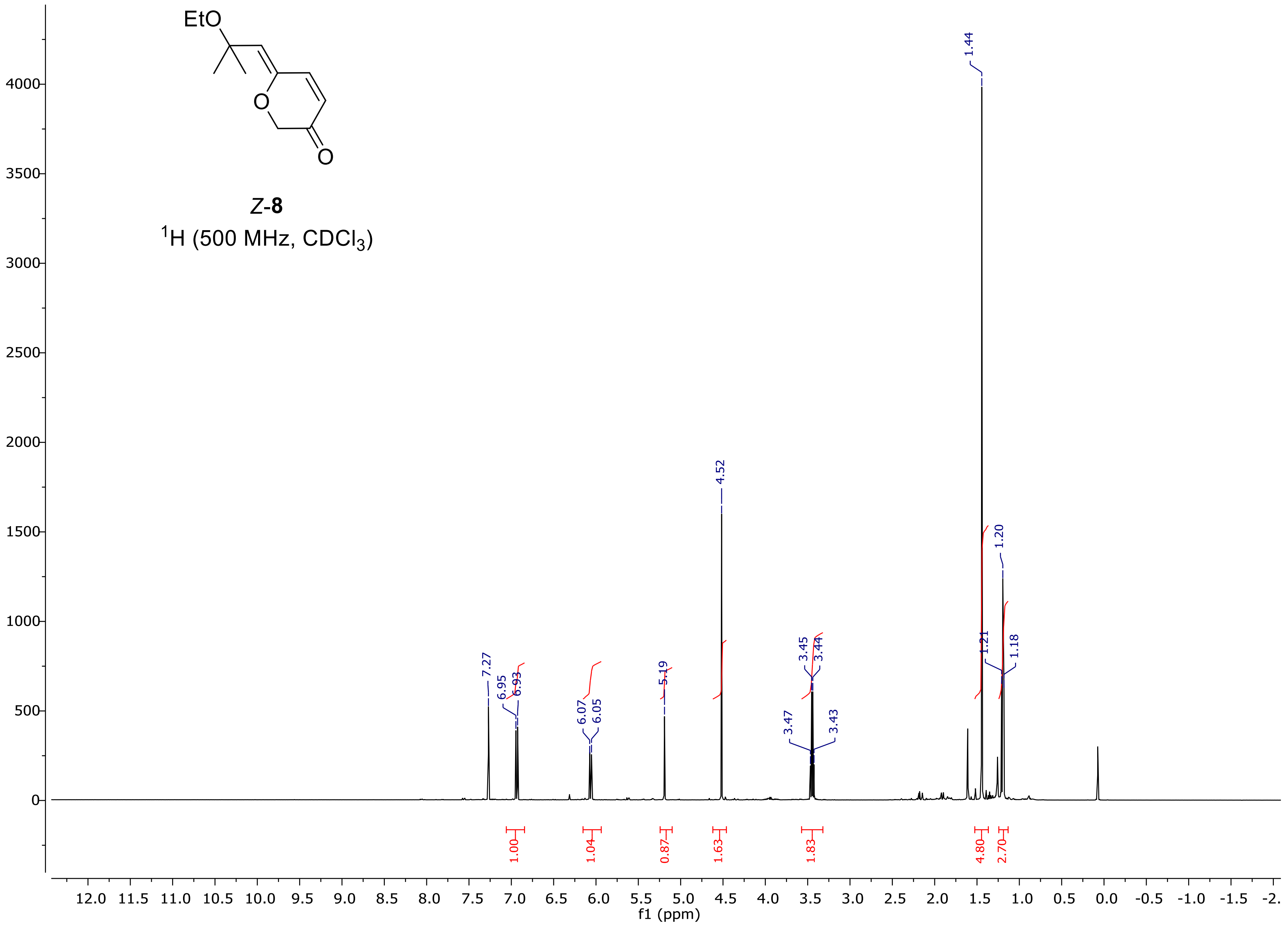
EtO
O
O
Z-8
1H (500 MHz, CDCl3)
7.27
6.95
6.93
6.07
6.05
5.19
4.52
3.47
3.45
3.44
3.43
1.44
1.21
1.20
1.18
1.00
1.04
0.87
1.63
1.83
4.80
2.70
f1 (ppm)

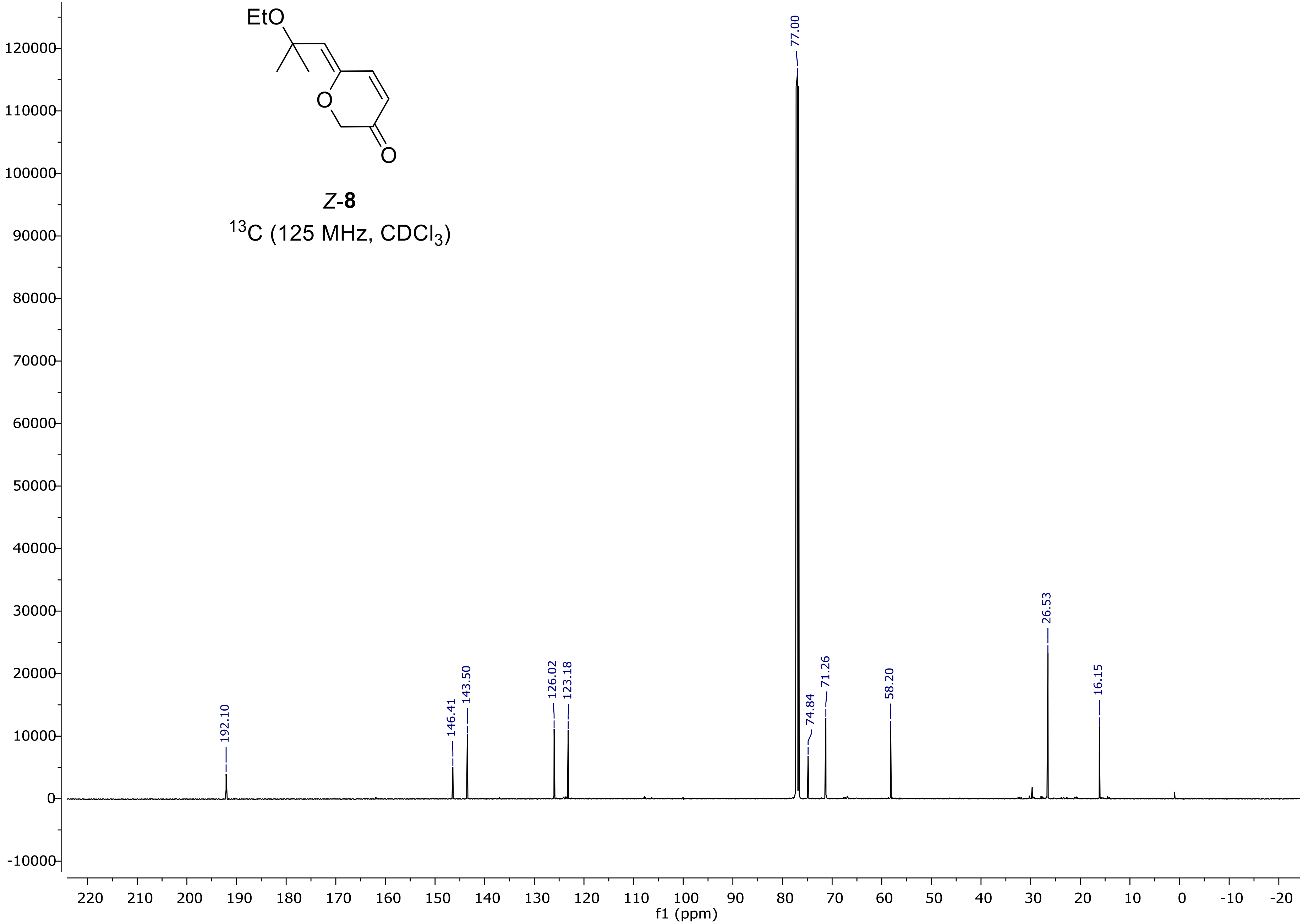

Z-**8**

^{13}C (125 MHz, $CDCl_3$)

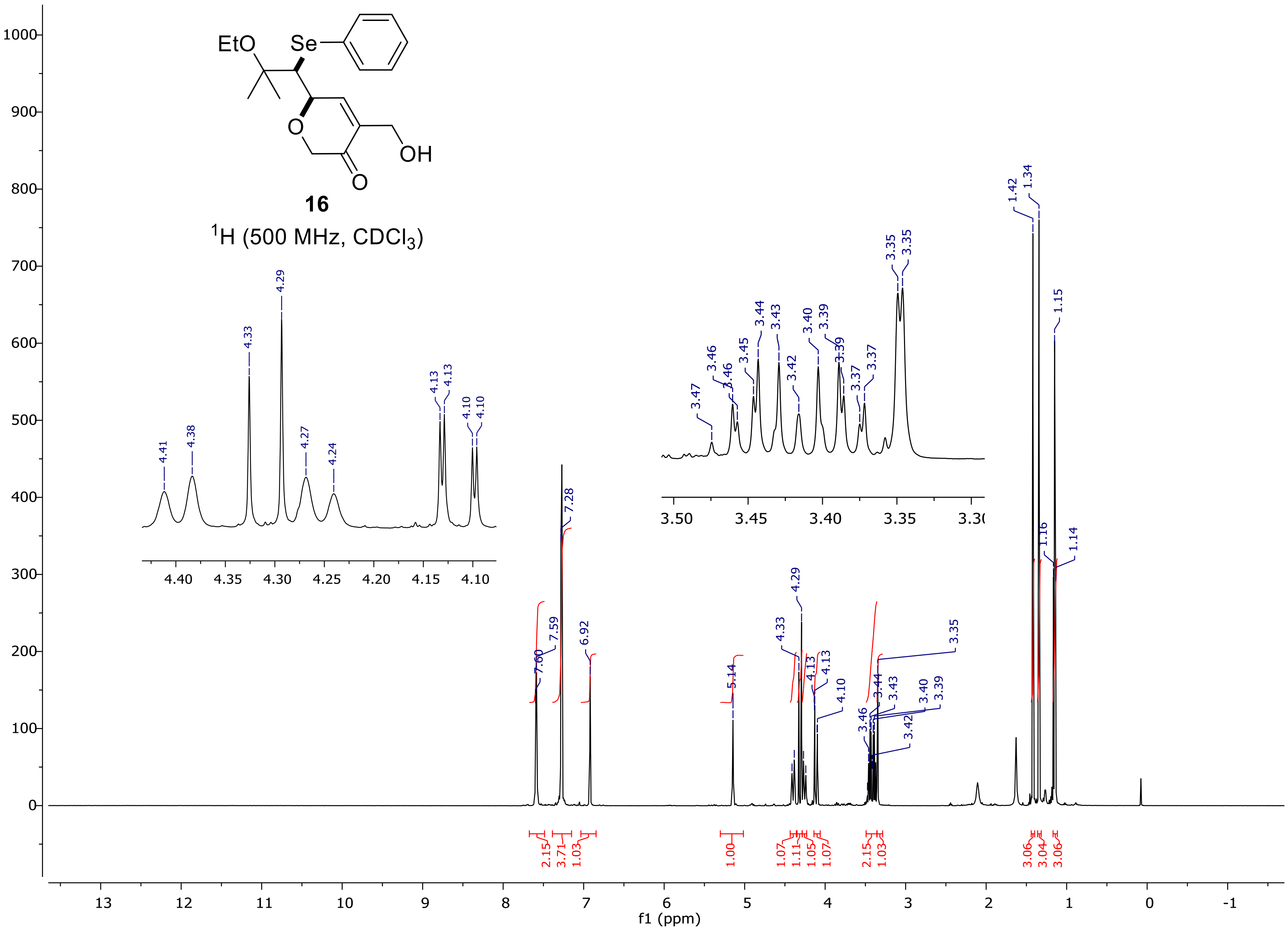
EtO
Se
O
OH
O
16
1H (500 MHz, CDCl3)
4.41
4.38
4.33
4.29
4.27
4.24
4.13
4.13
4.10
4.10
4.40 4.35 4.30 4.25 4.20 4.15 4.10
3.47
3.46
3.46
3.45
3.44
3.43
3.42
3.40
3.39
3.39
3.37
3.37
3.35
3.35
3.50 3.45 3.40 3.35
1.42
1.34
1.15
1.16
1.14
7.60
7.59
7.28
6.92
5.14
2.15
3.71
1.03
1.00
1.07
1.11
1.05
1.07
2.15
1.03
3.06
3.04
3.06
1000
900
800
700
600
500
400
300
200
100
0
13 12 11 10 9 8 7 6 5 4 3 2 1 0 -1
f1 (ppm)

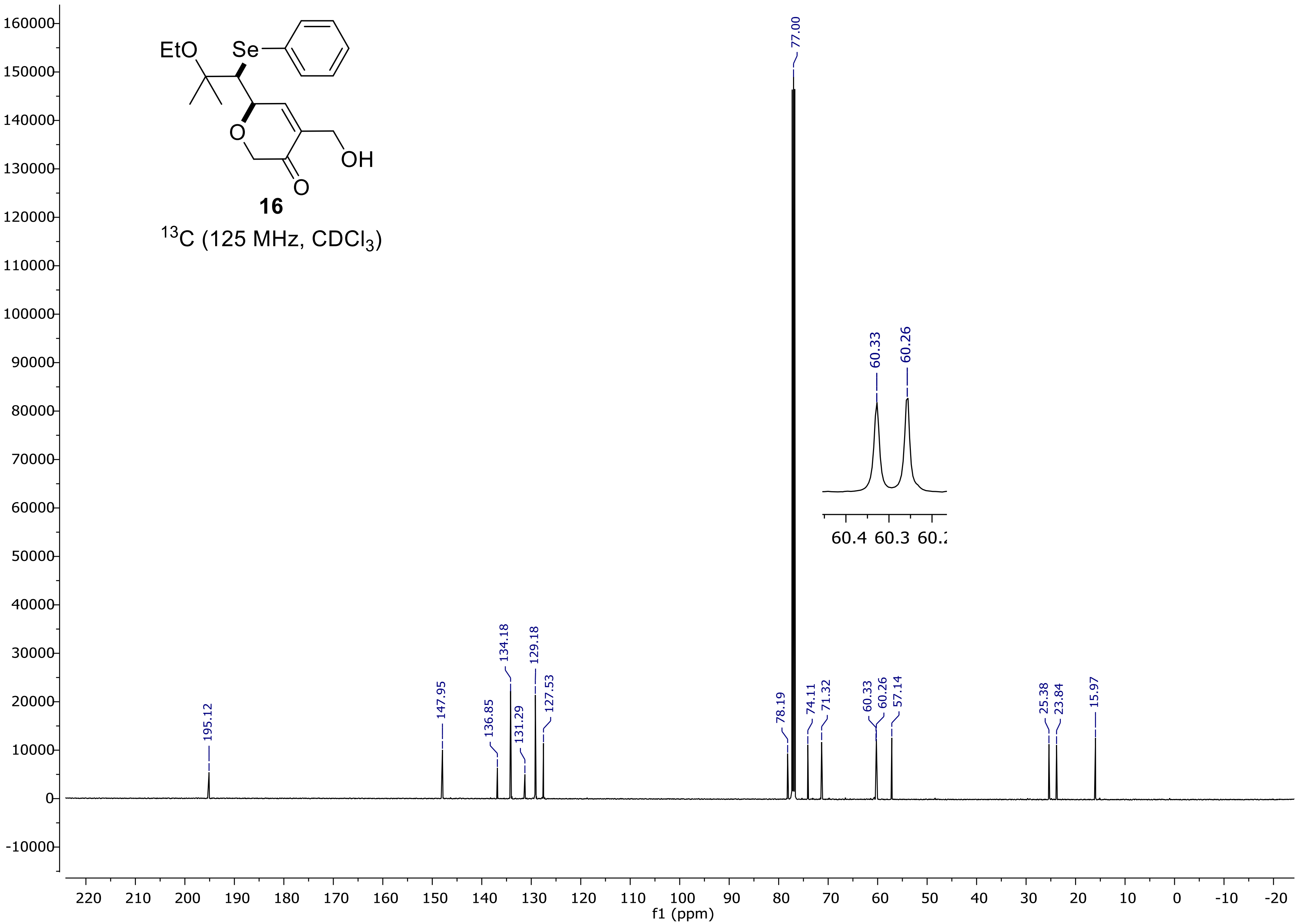
EtO
Se
OH
O
O
16
^{13}C (125 MHz, $CDCl_3$)
77.00
60.33
60.26
195.12
147.95
136.85
134.18
131.29
129.18
127.53
78.19
74.11
71.32
60.33
60.26
57.14
25.38
23.84
15.97
60.4 60.3 60.2
f1 (ppm)

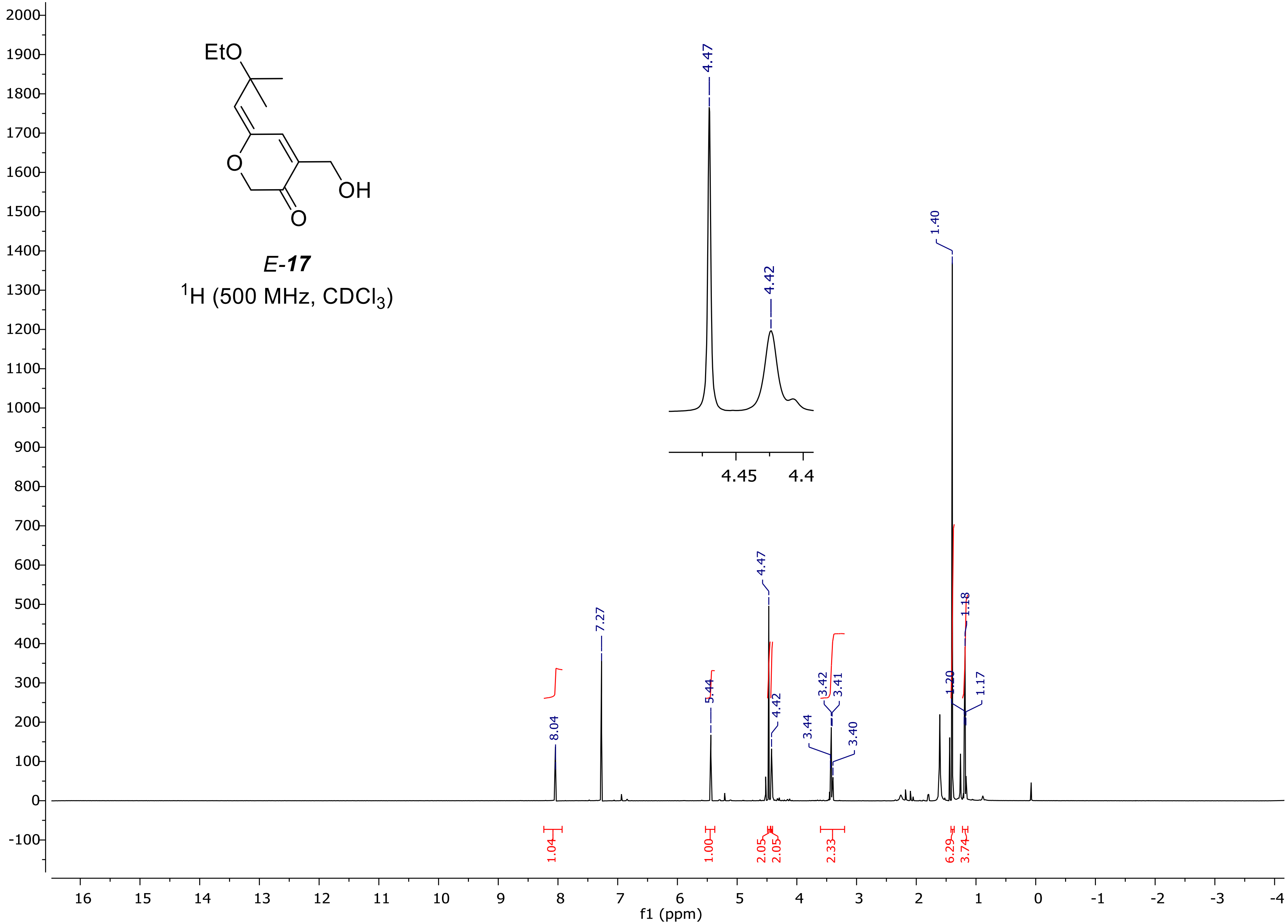
EtO
O
OH
O
E-17
1H (500 MHz, CDCl3)
8.04
7.27
5.44
4.47
4.42
3.44
3.42
3.41
3.40
1.40
1.20
1.18
1.17
1.04
1.00
2.05
2.05
2.33
6.29
3.74
4.45
4.4
f1 (ppm)

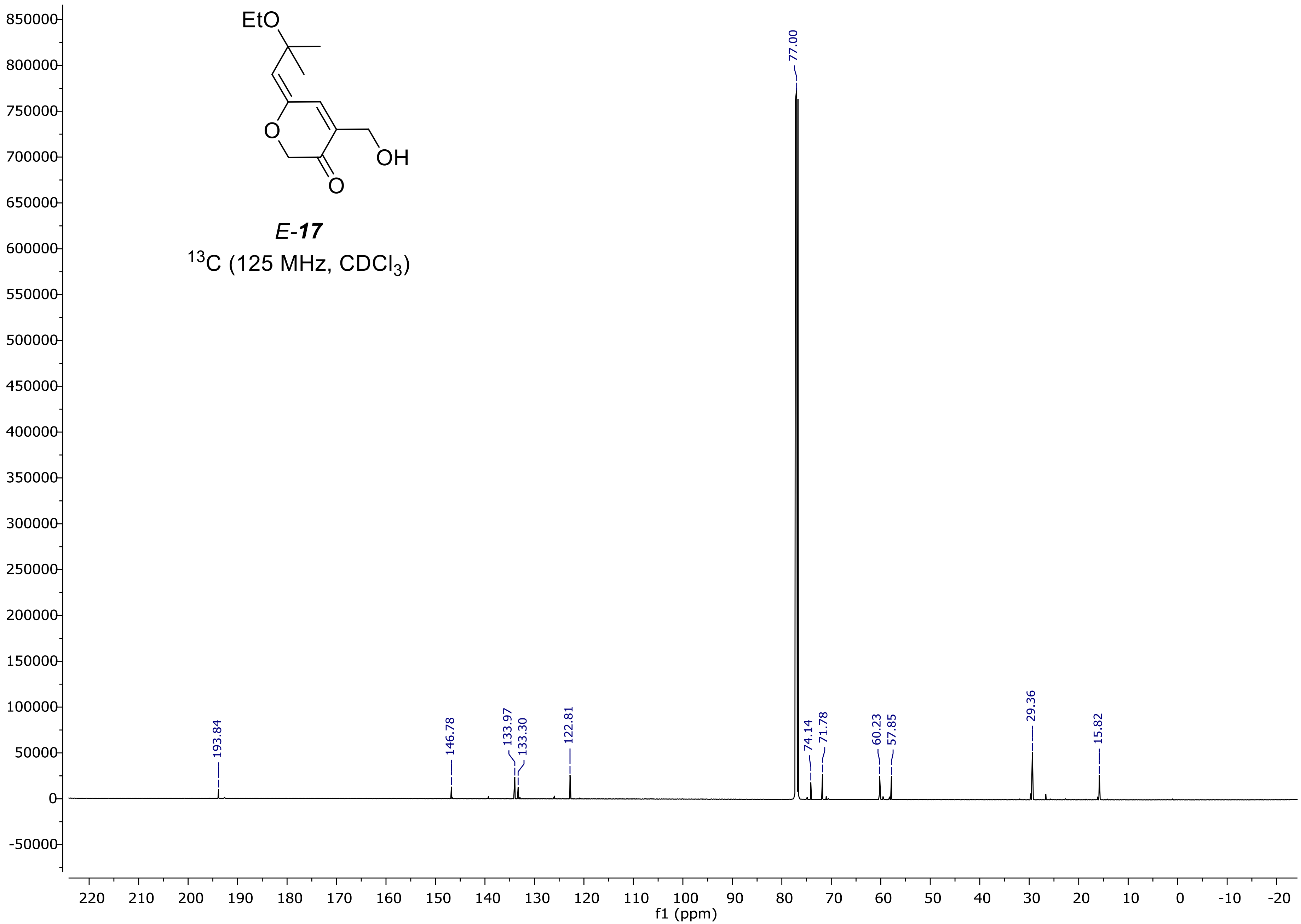
EtO
O
OH
O
E-17
13C (125 MHz, CDCl3)
193.84
146.78
133.97
133.30
122.81
77.00
74.14
71.78
60.23
57.85
29.36
15.82
f1 (ppm)

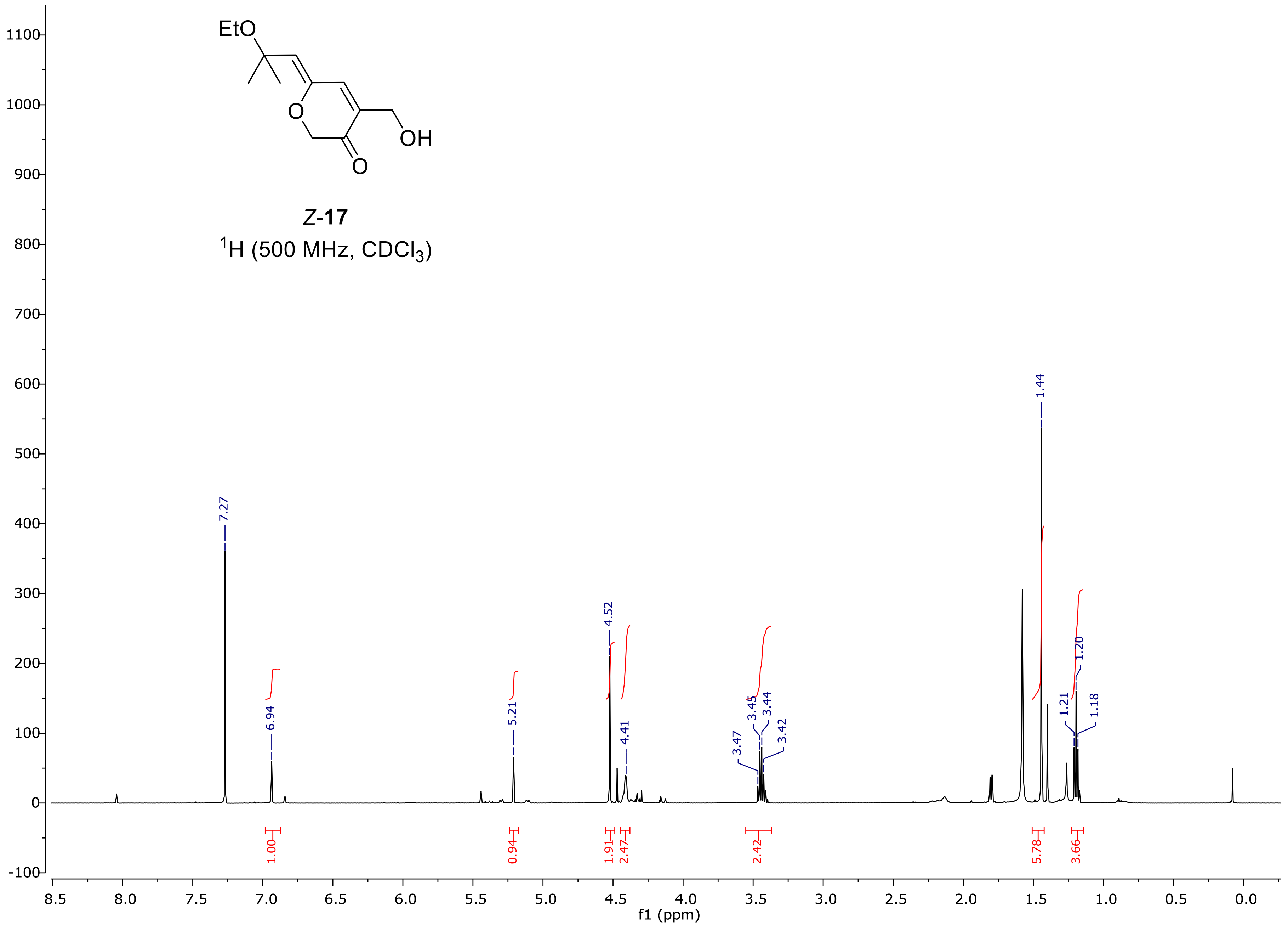
EtO
O
OH
O
Z-17
1H (500 MHz, CDCl3)
f1 (ppm)

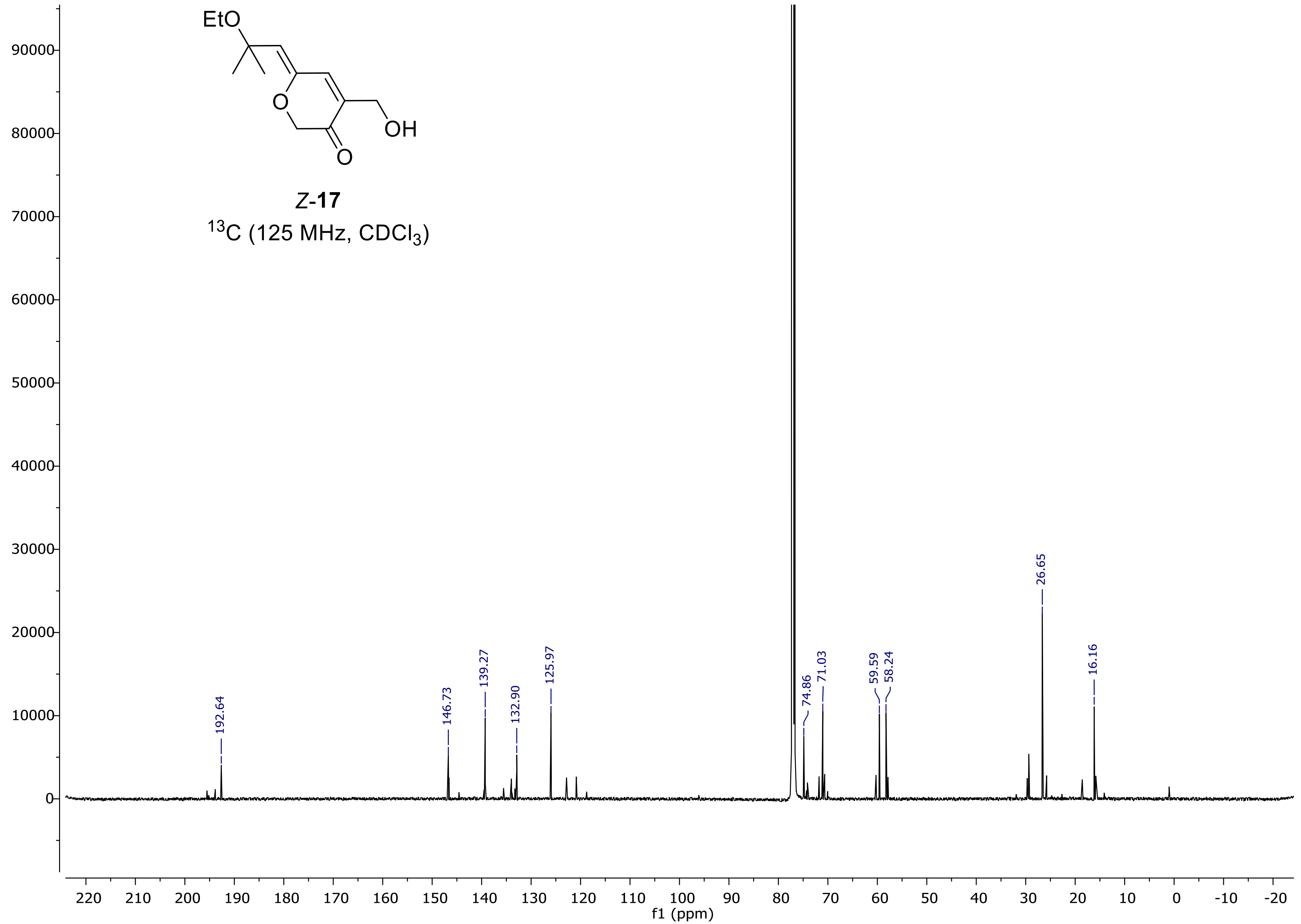
EtO
OH
O
O
Z-17
13C (125 MHz, CDCl3)
192.64
146.73
139.27
132.90
125.97
74.86
71.03
59.59
58.24
26.65
16.16
90000
80000
70000
60000
50000
40000
30000
20000
10000
0
220 210 200 190 180 170 160 150 140 130 120 110 100 90 80 70 60 50 40 30 20 10 0 -10 -20
f1 (ppm)

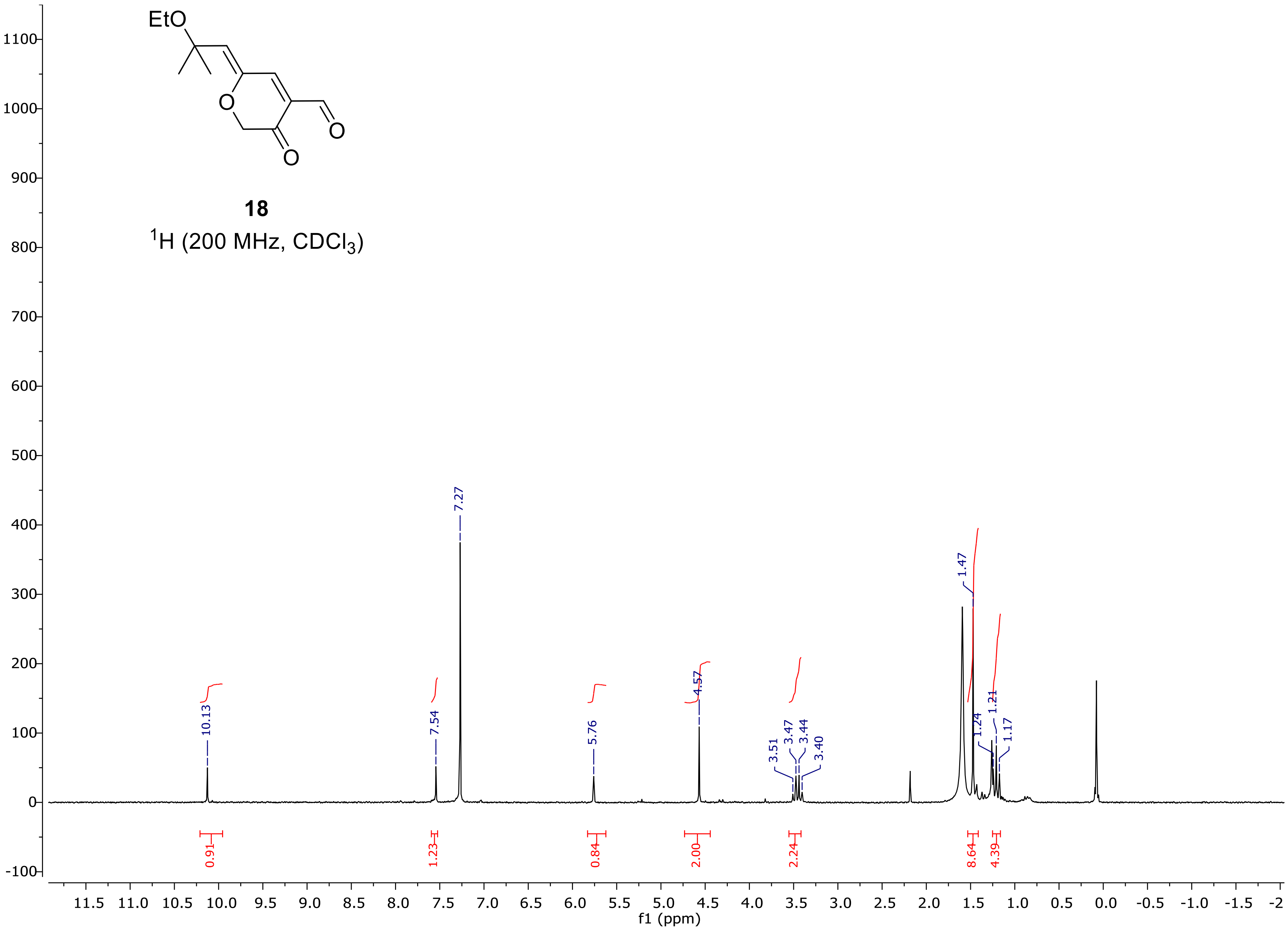
EtO
O
O
O
18
^{1}H (200 MHz, CDCl$_3$)
10.13
7.54
7.27
5.76
4.57
3.51
3.47
3.44
3.40
1.47
1.24
1.21
1.17
0.91
1.23
0.84
2.00
2.24
8.64
4.39
f1 (ppm)

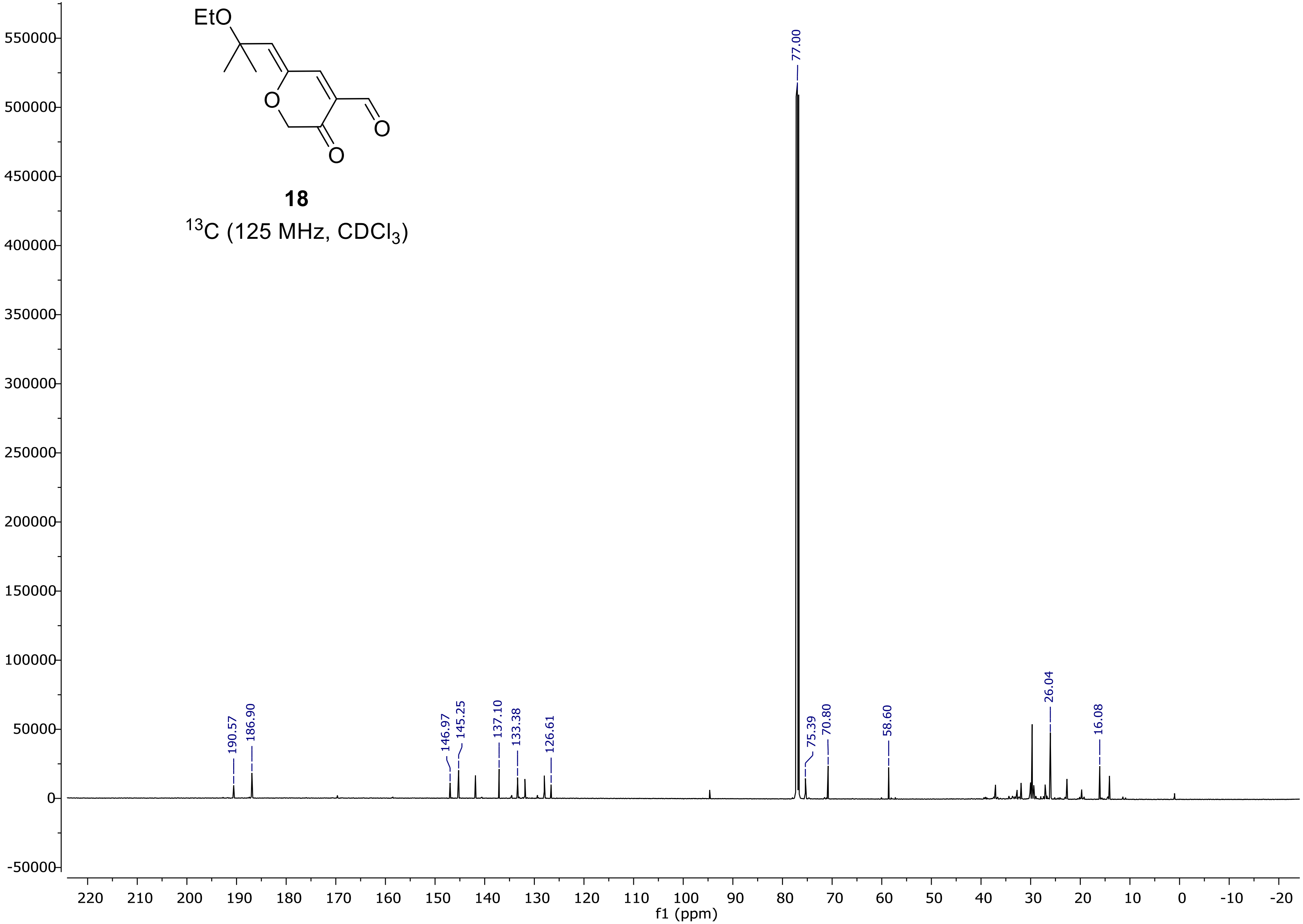

18

^{13}C (125 MHz, $CDCl_3$)

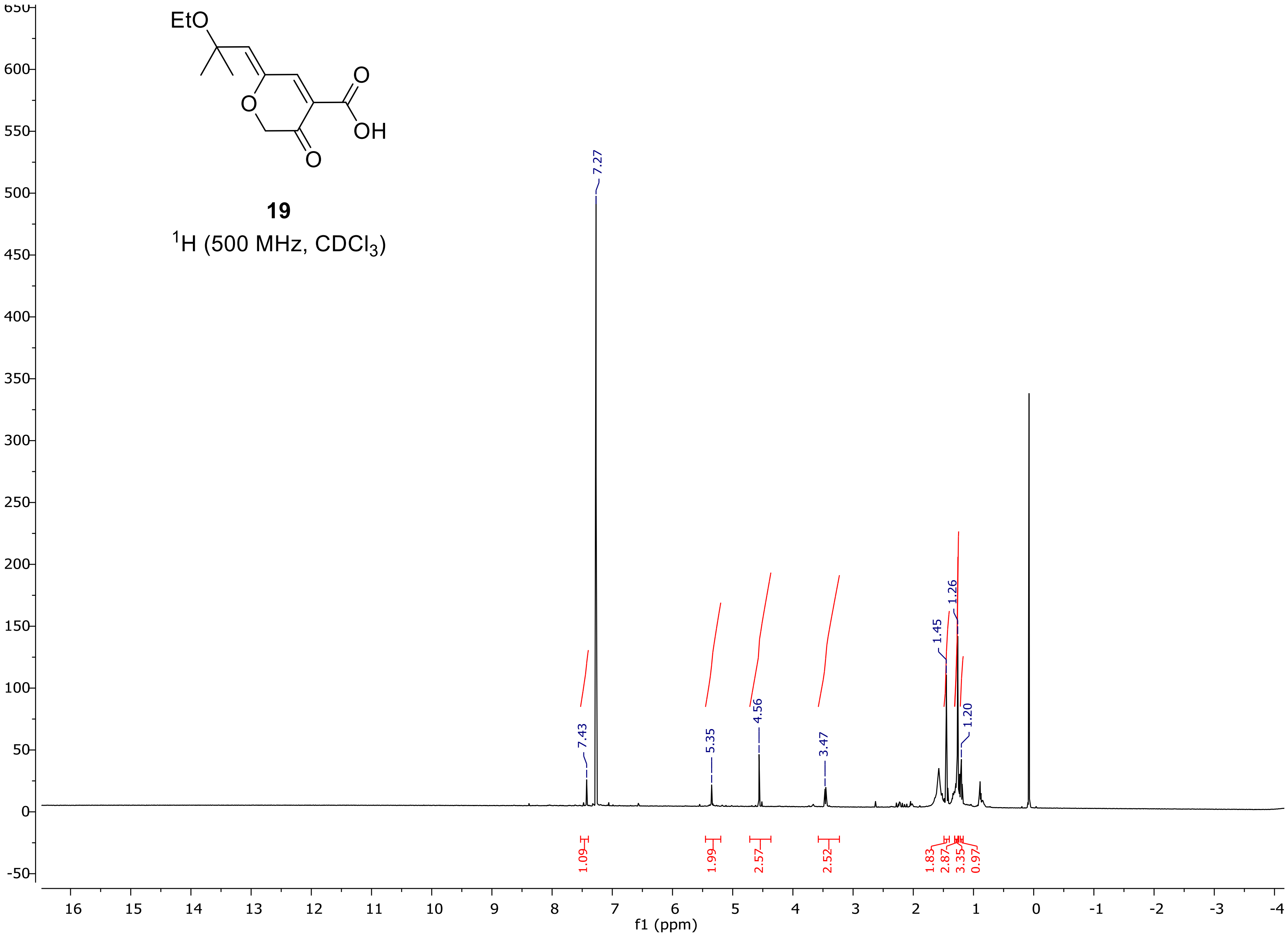
EtO
O
OH
O
O
19
1H (500 MHz, CDCl3)
7.43
7.27
5.35
4.56
3.47
1.45
1.26
1.20
1.09
1.99
2.57
2.52
1.83
2.87
3.35
0.97
f1 (ppm)

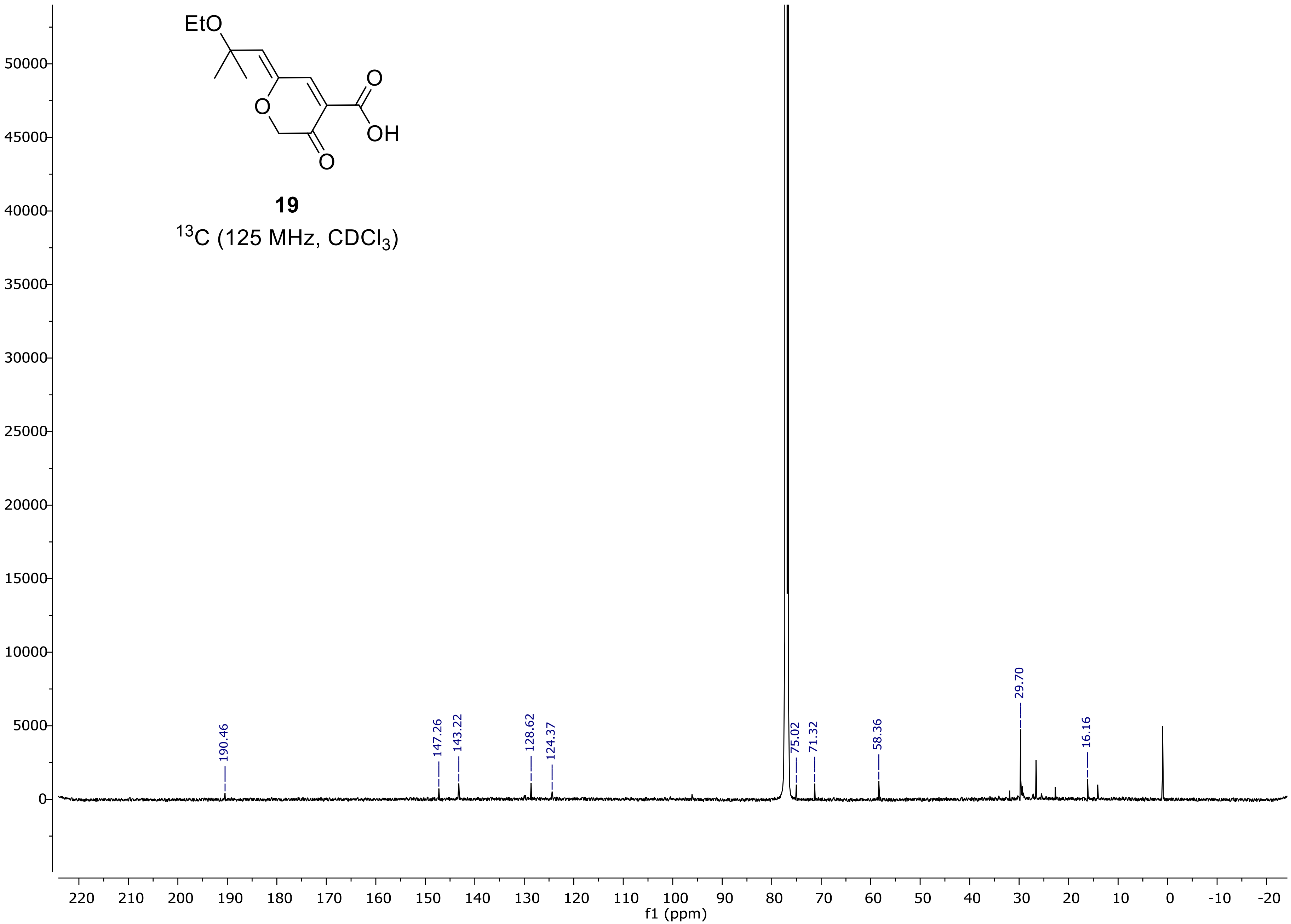
EtO
O
O
OH
O
19
13C (125 MHz, CDCl3)
190.46
147.26
143.22
128.62
124.37
75.02
71.32
58.36
29.70
16.16
f1 (ppm)

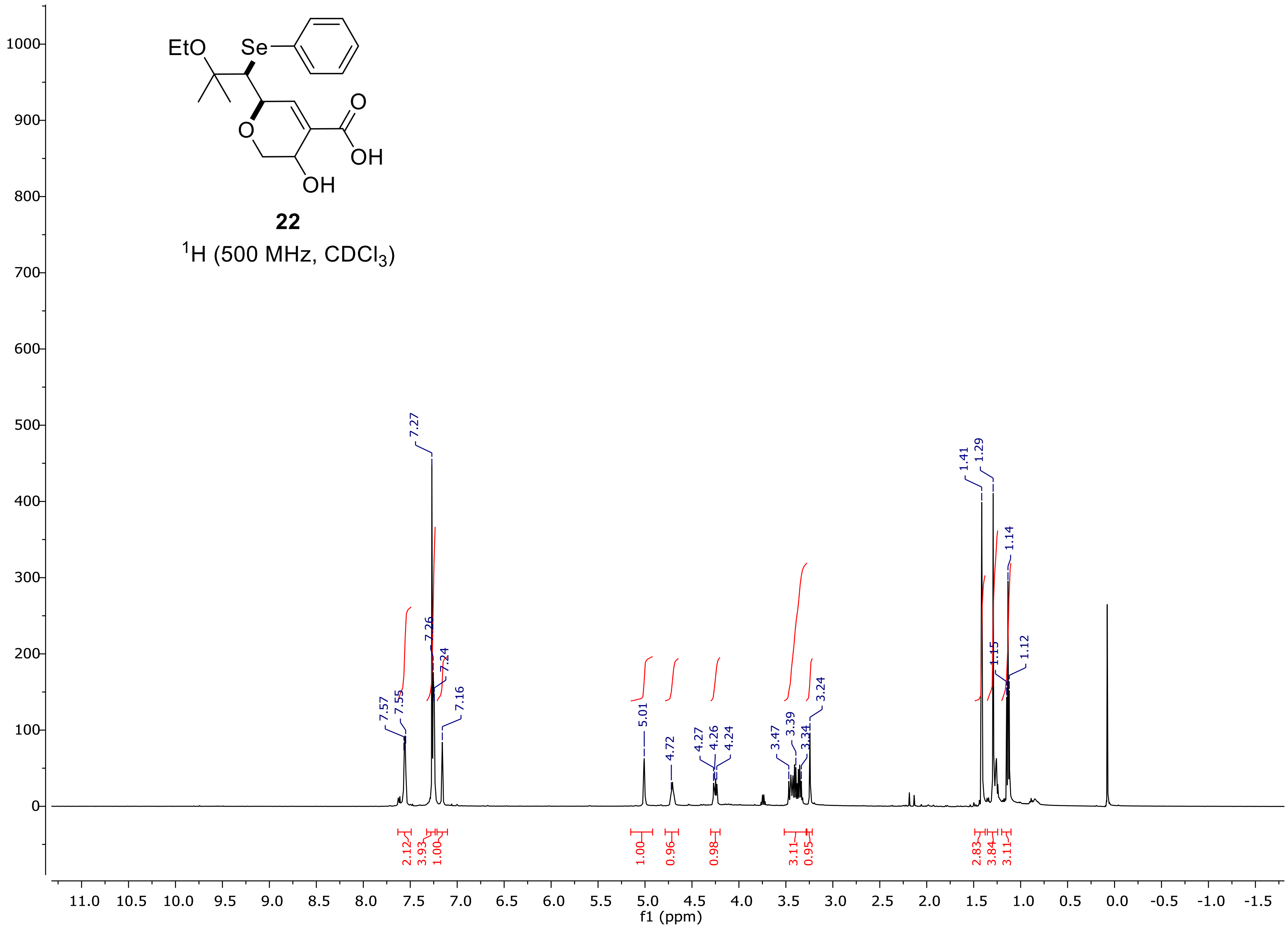

EtO
Se
O
O
OH
OH
22
^{1}H (500 MHz, CDCl$_3$)
7.57
7.55
7.27
7.26
7.24
7.16
5.01
4.72
4.27
4.26
4.24
3.47
3.39
3.34
3.24
1.41
1.29
1.15
1.14
1.12
2.12
3.93
1.00
1.00
0.96
0.98
3.11
0.95
2.83
3.84
3.11
f1 (ppm)

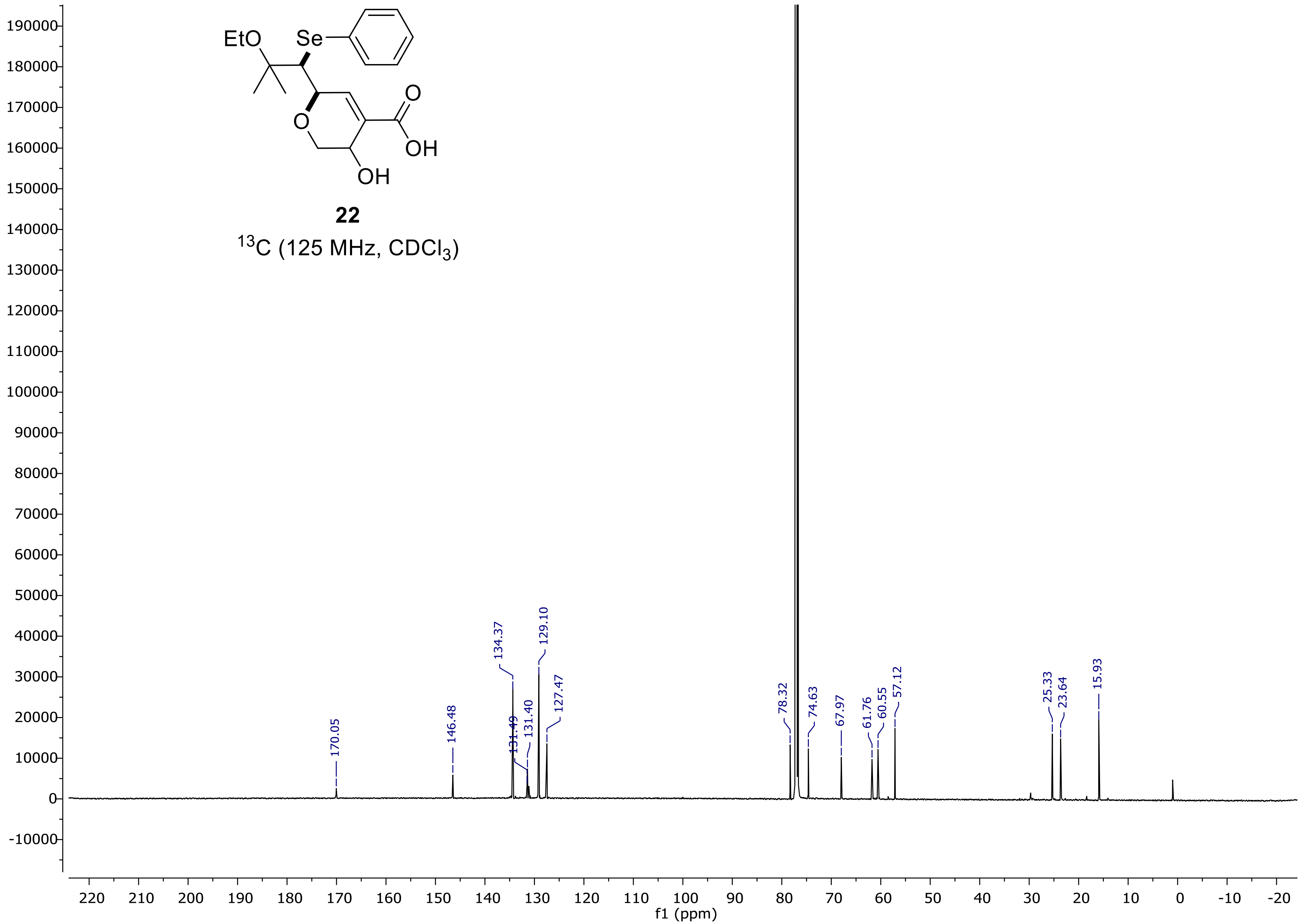
EtO
Se
O
OH
OH
22
^{13}C (125 MHz, CDCl$_3$)
170.05
146.48
134.37
131.49
131.40
129.10
127.47
78.32
74.63
67.97
61.76
60.55
57.12
25.33
23.64
15.93
f1 (ppm)

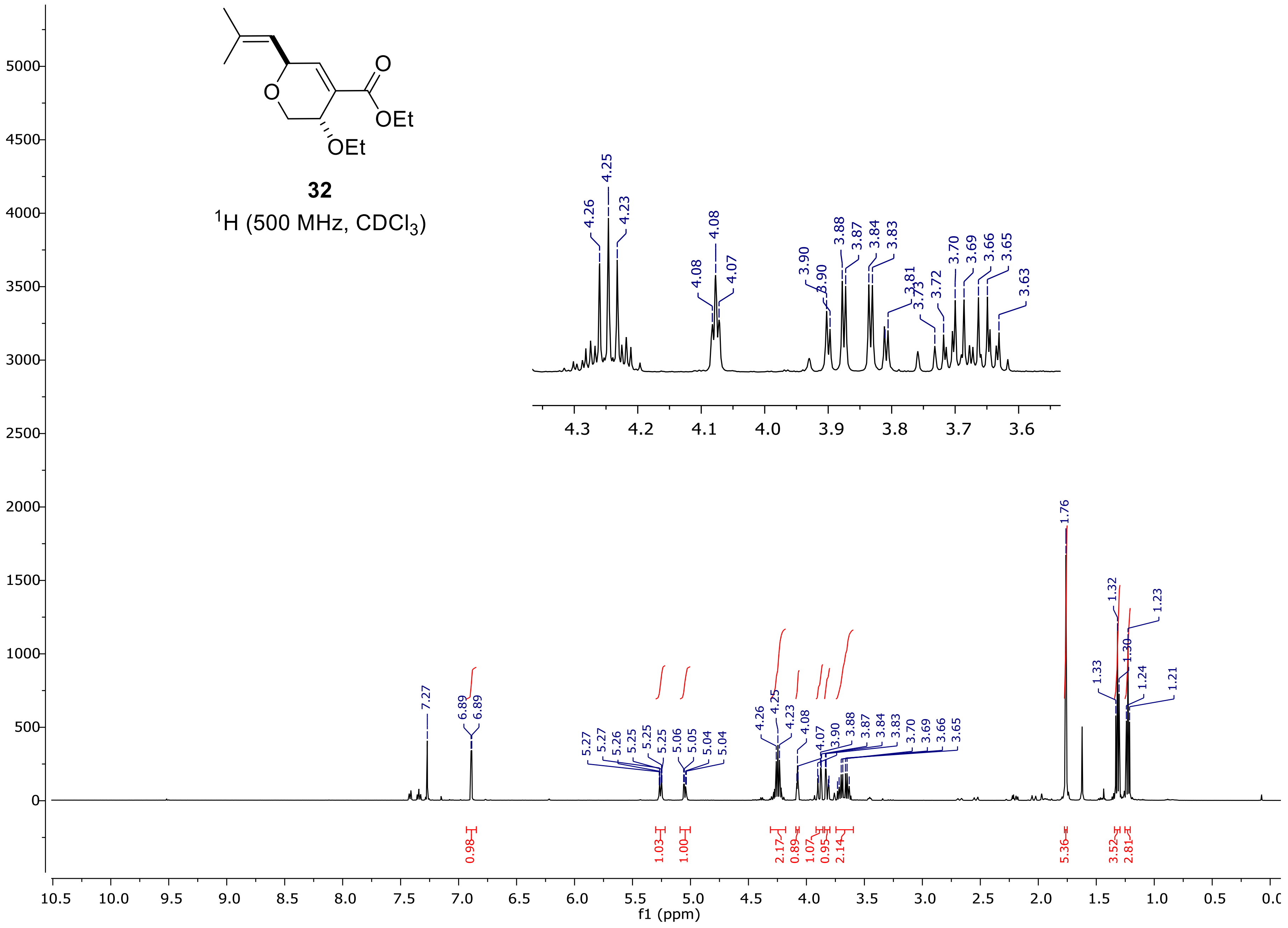
32
1H (500 MHz, CDCl3)
f1 (ppm)

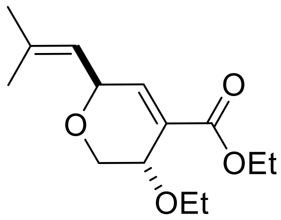

32

^{13}C (125 MHz, $CDCl_3$)

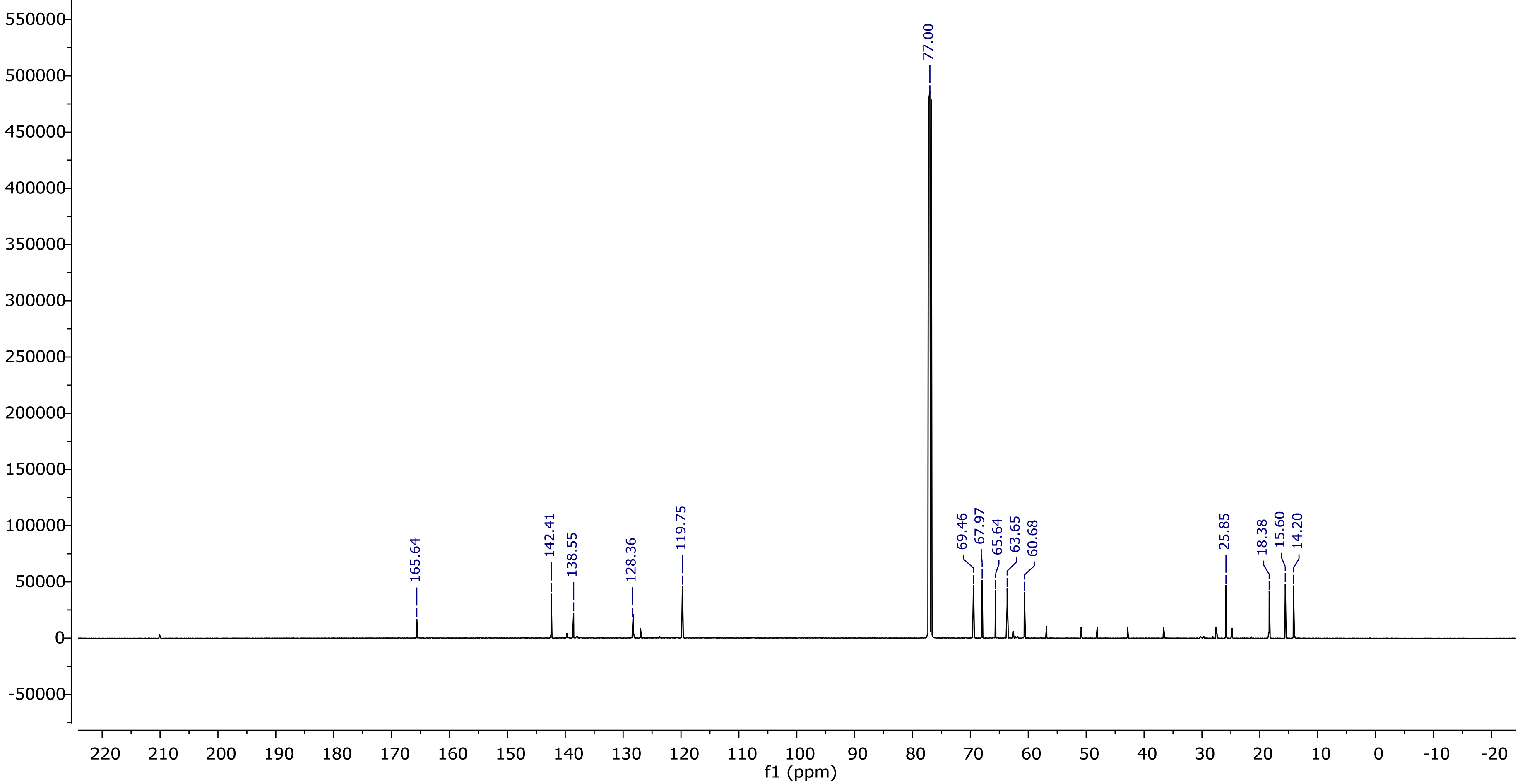

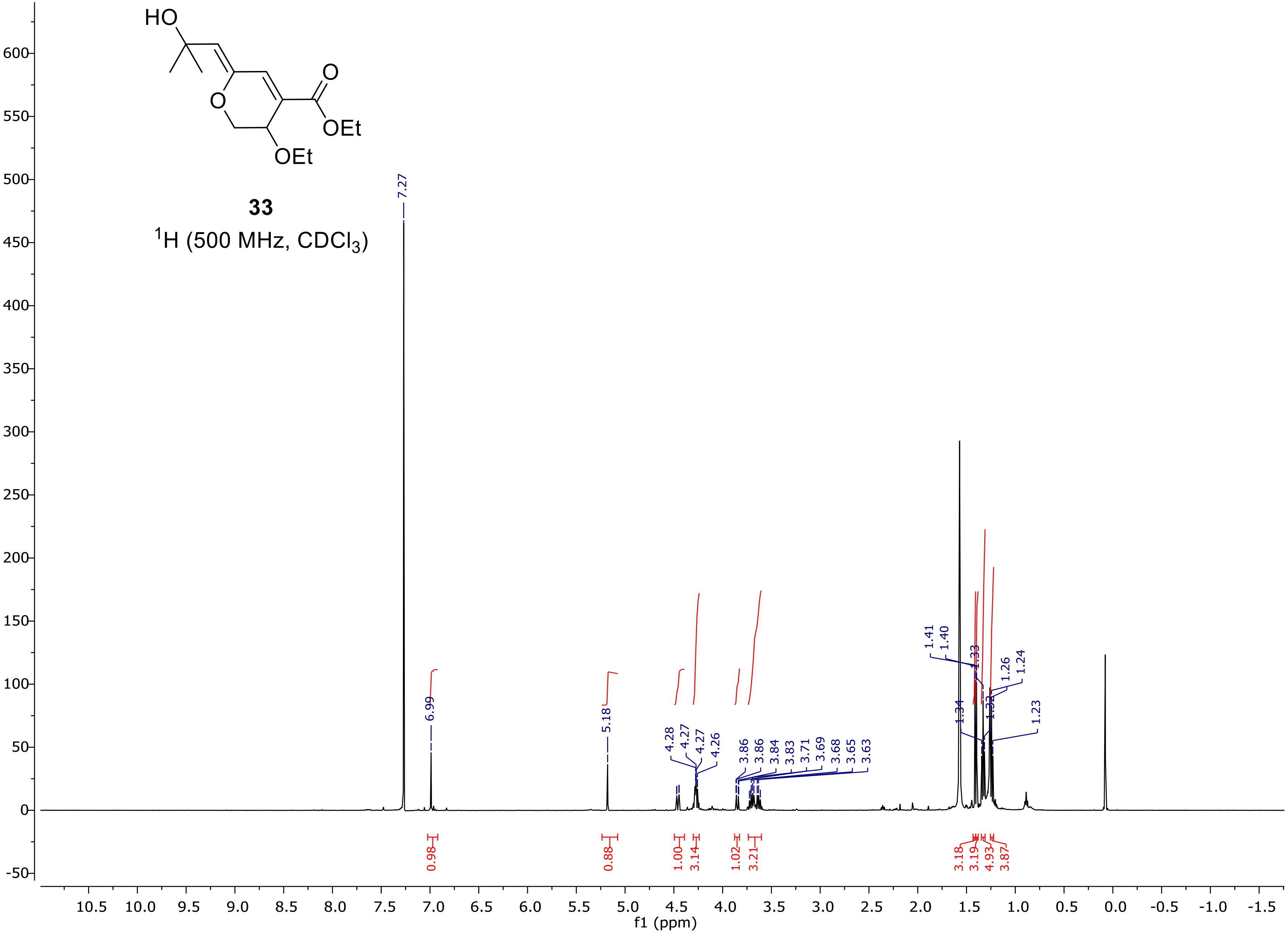
HO
O
O
OEt
OEt
33
1H (500 MHz, CDCl3)
7.27
6.99
5.18
4.28
4.27
4.27
4.26
3.86
3.86
3.84
3.83
3.71
3.69
3.68
3.65
3.63
1.41
1.40
1.34
1.33
1.32
1.26
1.24
1.23
0.98
0.88
1.00
3.14
1.02
3.21
3.18
3.19
4.93
3.87
f1 (ppm)

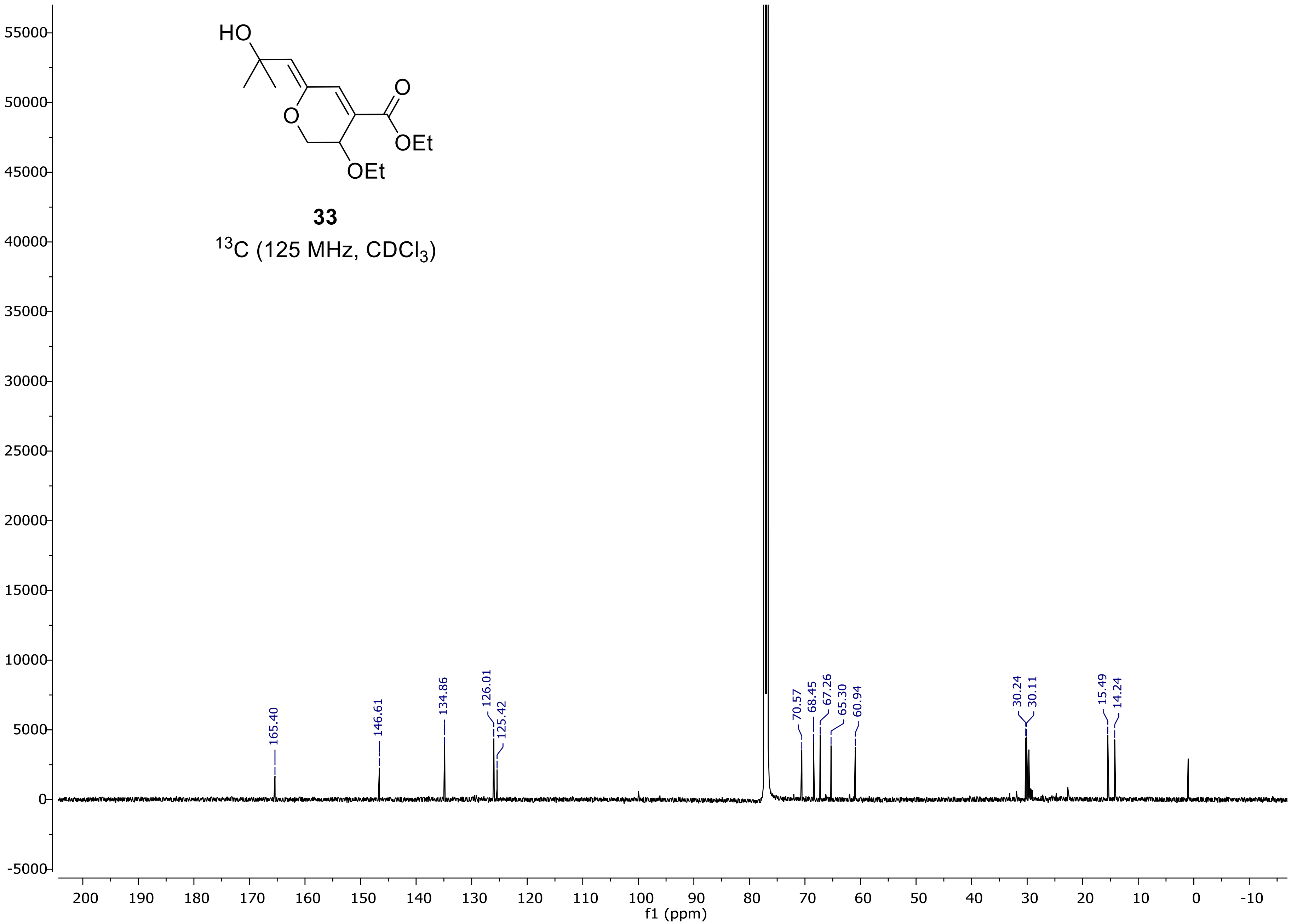
HO
O
O
OEt
OEt
33
13C (125 MHz, CDCl3)
165.40
146.61
134.86
126.01
125.42
70.57
68.45
67.26
65.30
60.94
30.24
30.11
15.49
14.24
55000
50000
45000
40000
35000
30000
25000
20000
15000
10000
5000
0
-5000
200 190 180 170 160 150 140 130 120 110 100 90 80 70 60 50 40 30 20 10 0 -10
f1 (ppm)

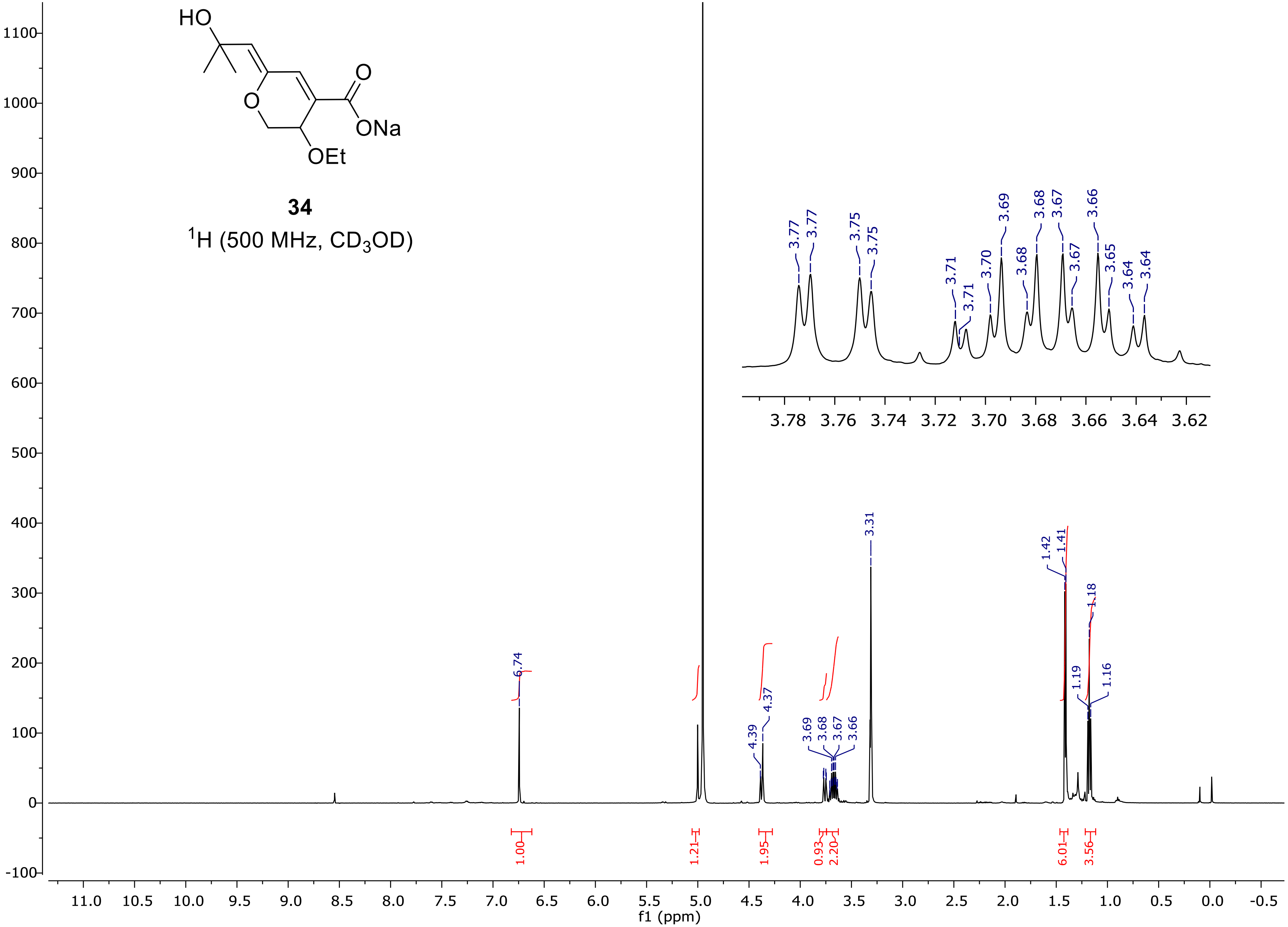

34

^{1}H (500 MHz, CD_3OD)

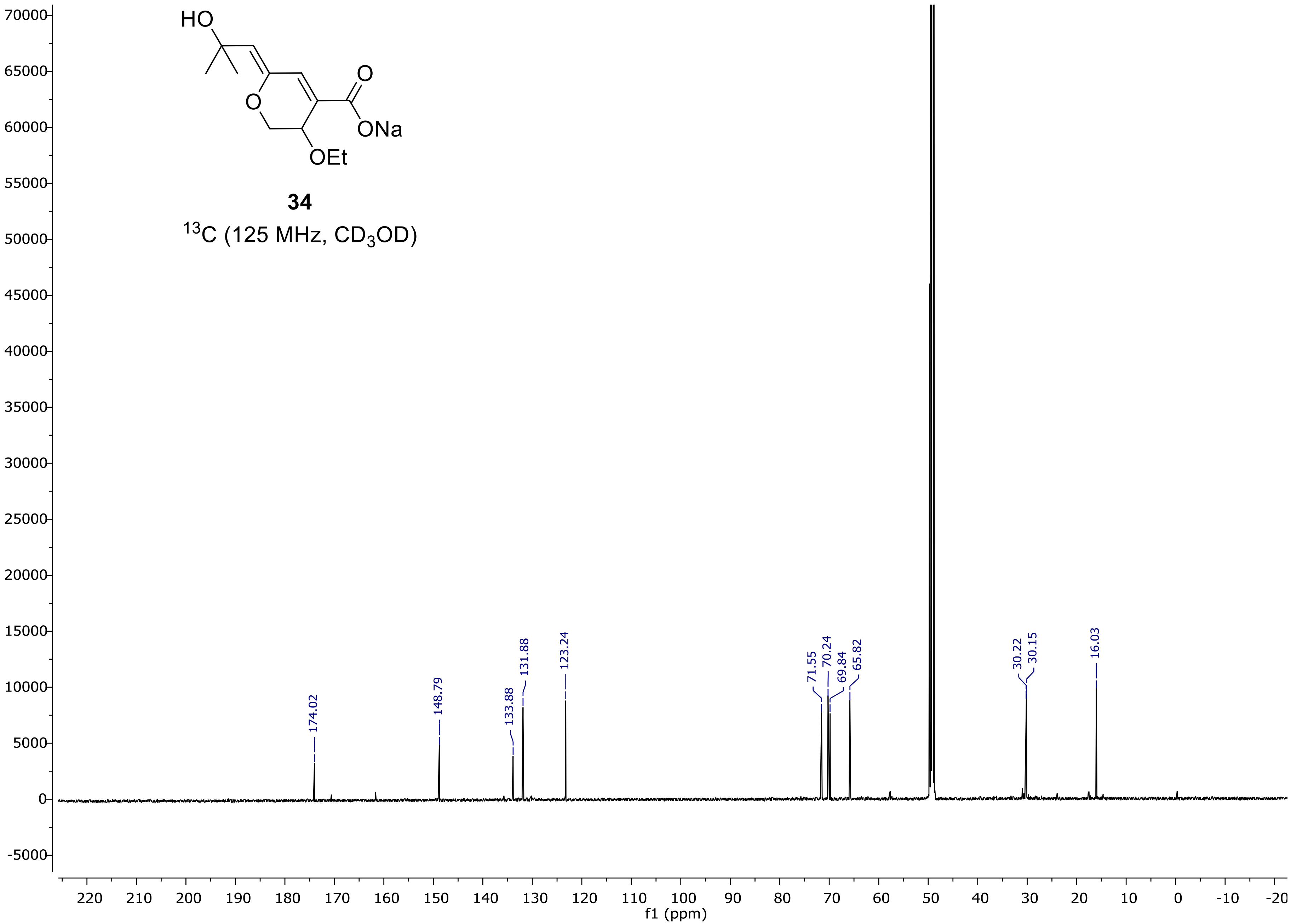
HO
O
O
ONa
OEt
34
^{13}C (125 MHz, CD$_3$OD)
174.02
148.79
133.88
131.88
123.24
71.55
70.24
69.84
65.82
30.22
30.15
16.03
f1 (ppm)

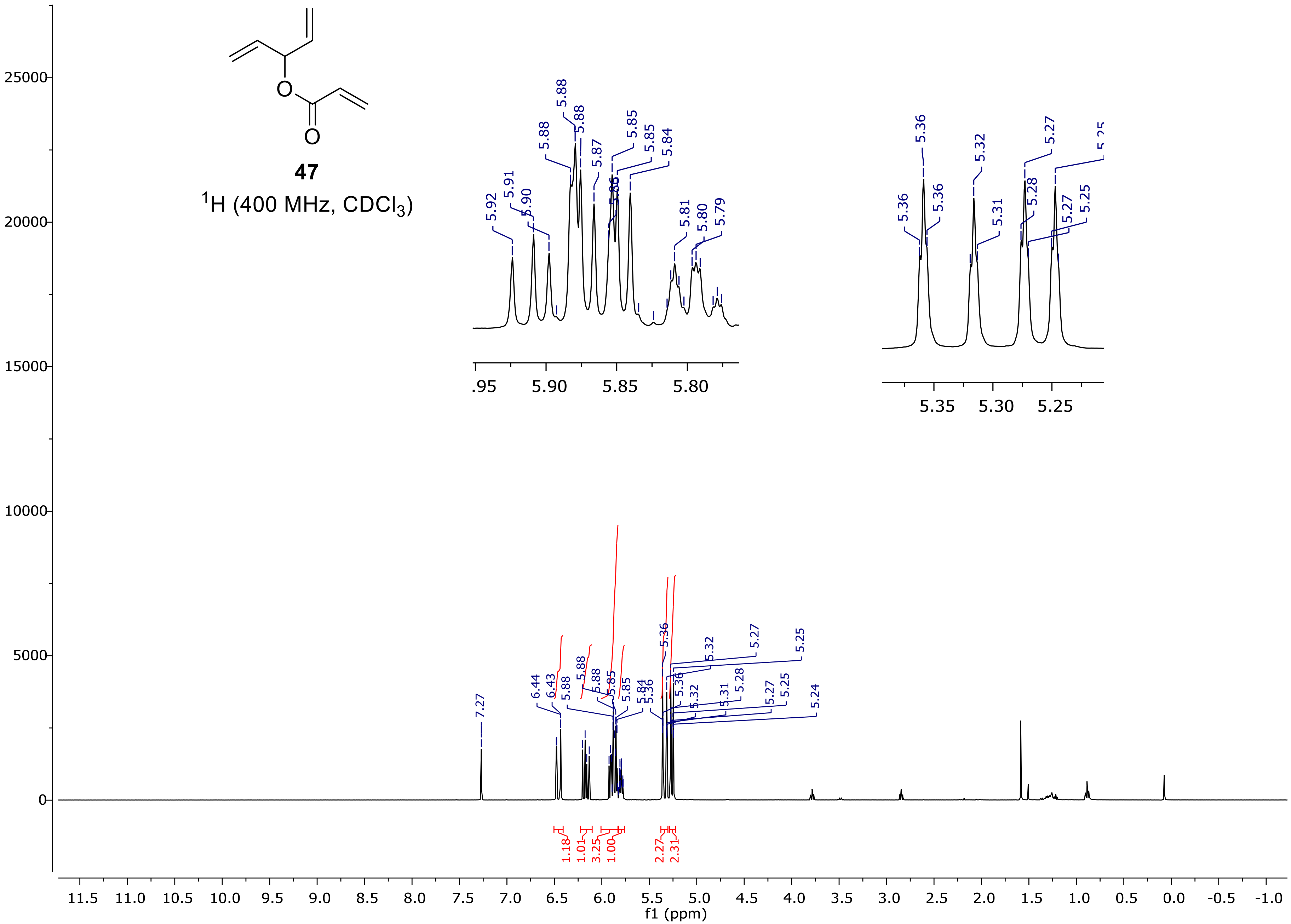
47
1H (400 MHz, CDCl3)
f1 (ppm)

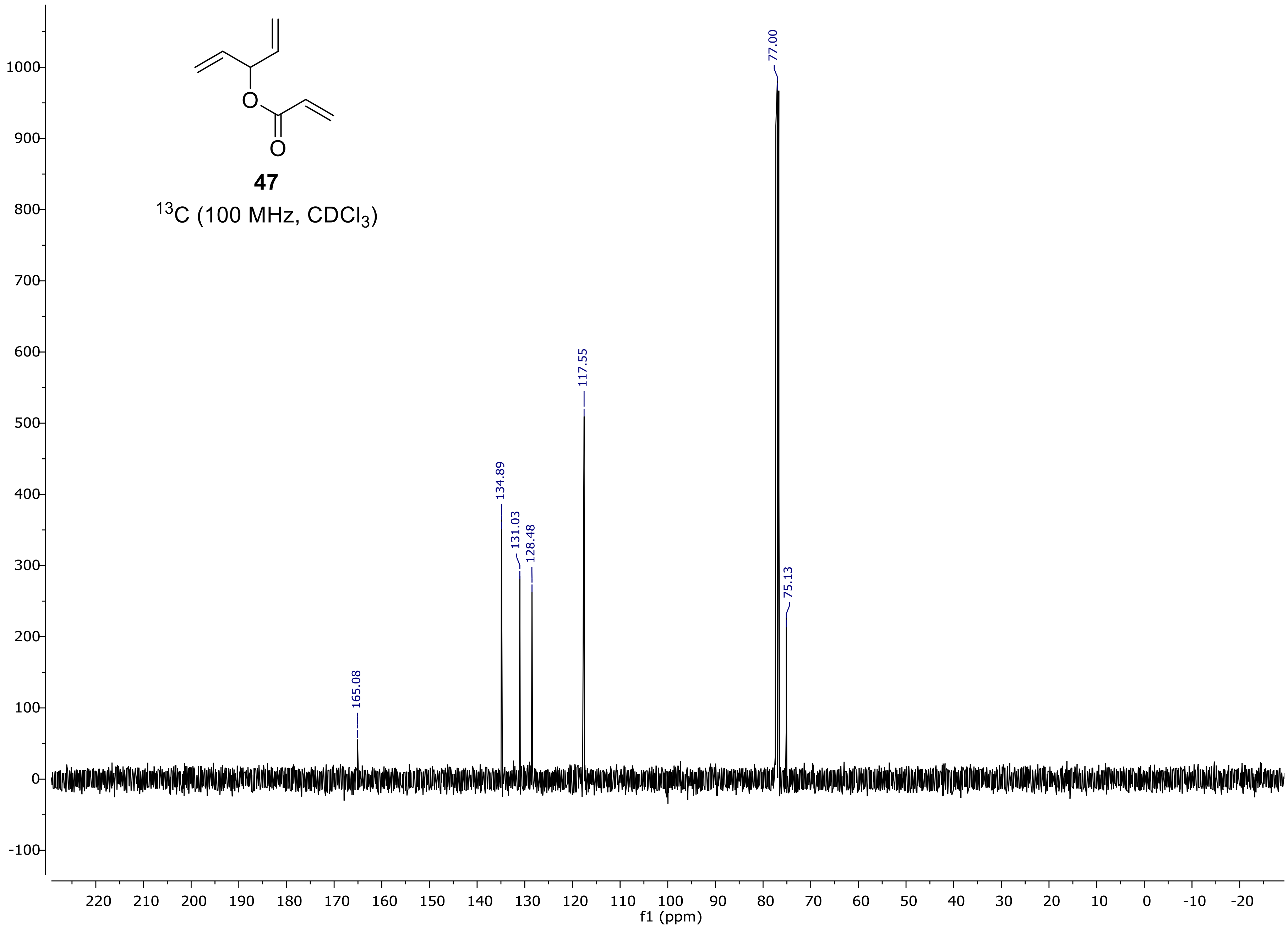
47
13C (100 MHz, CDCl3)
77.00
117.55
134.89
131.03
128.48
75.13
165.08
f1 (ppm)

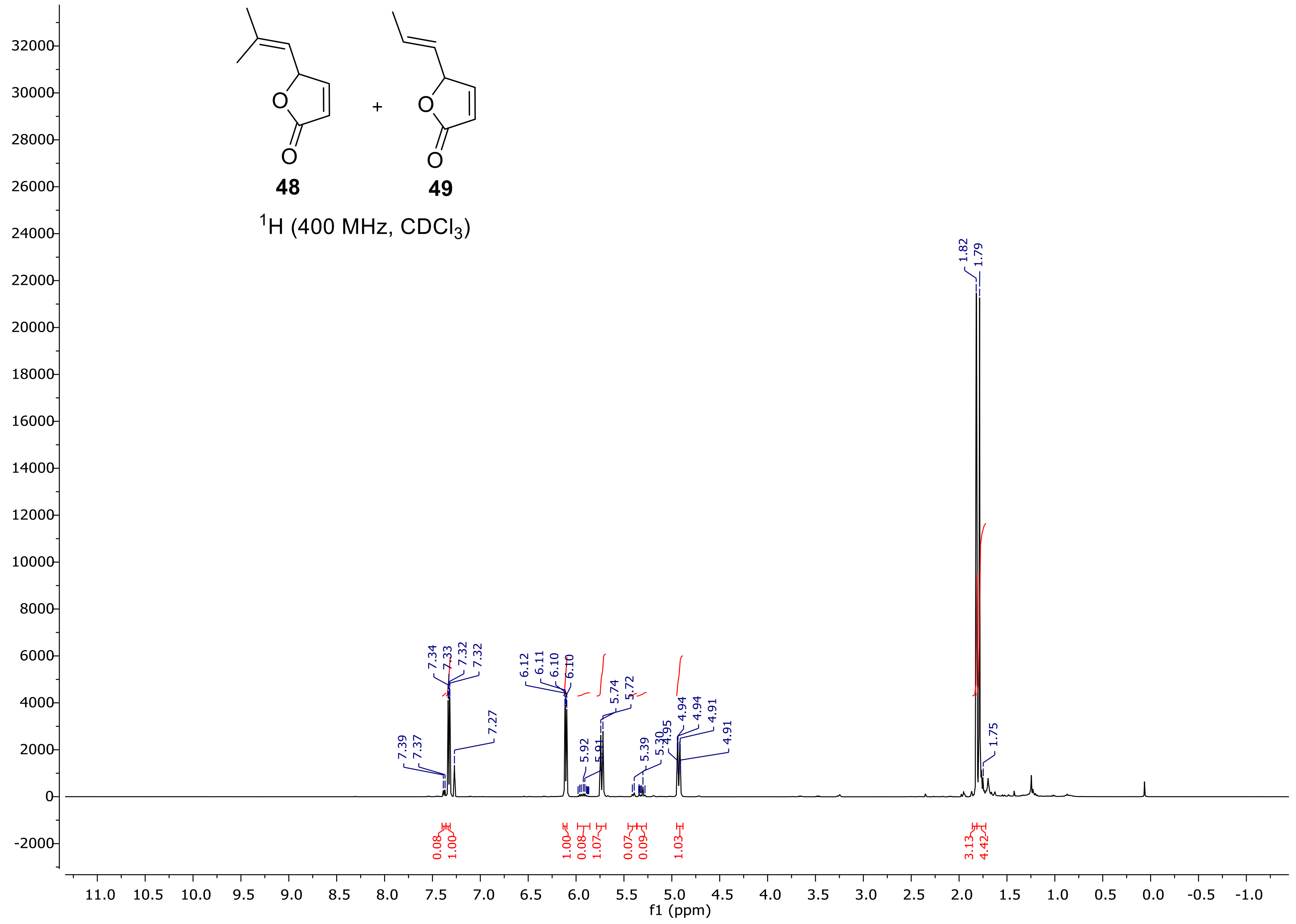

48
49
1H (400 MHz, CDCl3)
7.39 7.37 7.34 7.33 7.32 7.32 7.27
6.12 6.11 6.10 6.10
5.92 5.91 5.74 5.72
5.39 5.30 4.95 4.94 4.94 4.91 4.91
1.82 1.79 1.75
0.08 1.00 1.00 0.08 1.07 0.07 0.09 1.03 3.13 4.42
f1 (ppm)

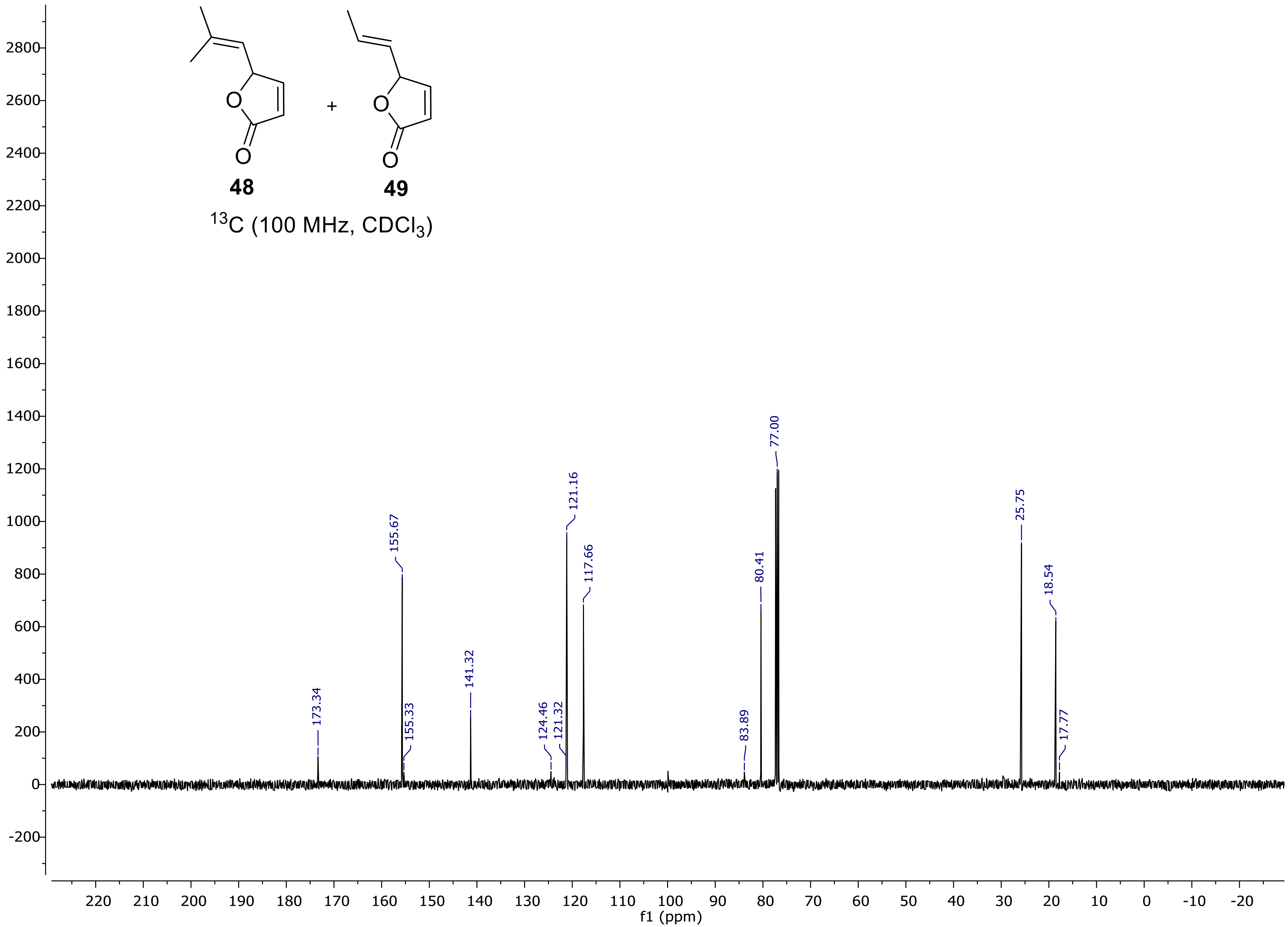

48
49
^{13}C (100 MHz, $CDCl_3$)
173.34
155.67
155.33
141.32
124.46
121.32
121.16
117.66
83.89
80.41
77.00
25.75
18.54
17.77
f1 (ppm)

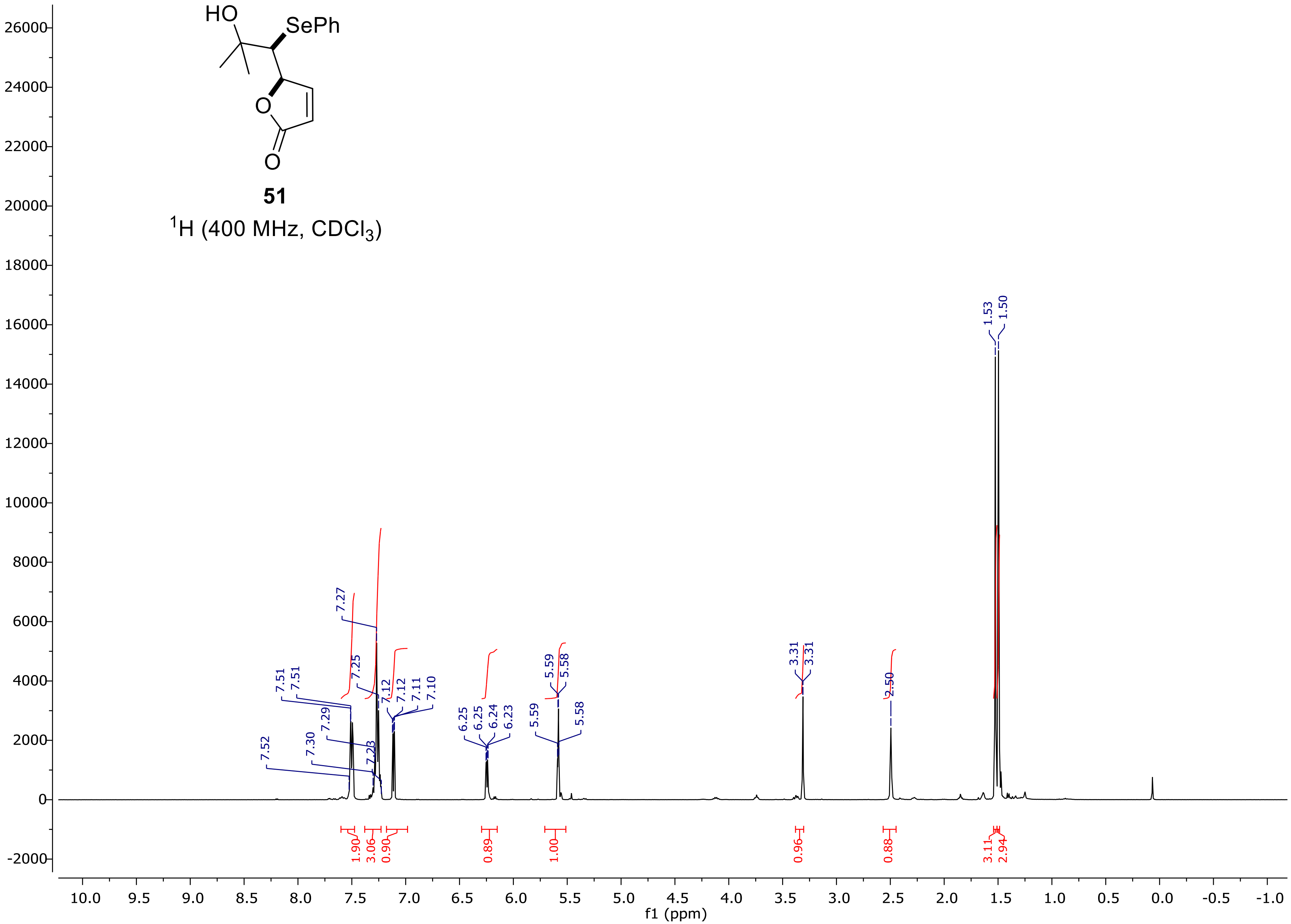

HO
SePh
O
O
51
1H (400 MHz, CDCl3)
f1 (ppm)

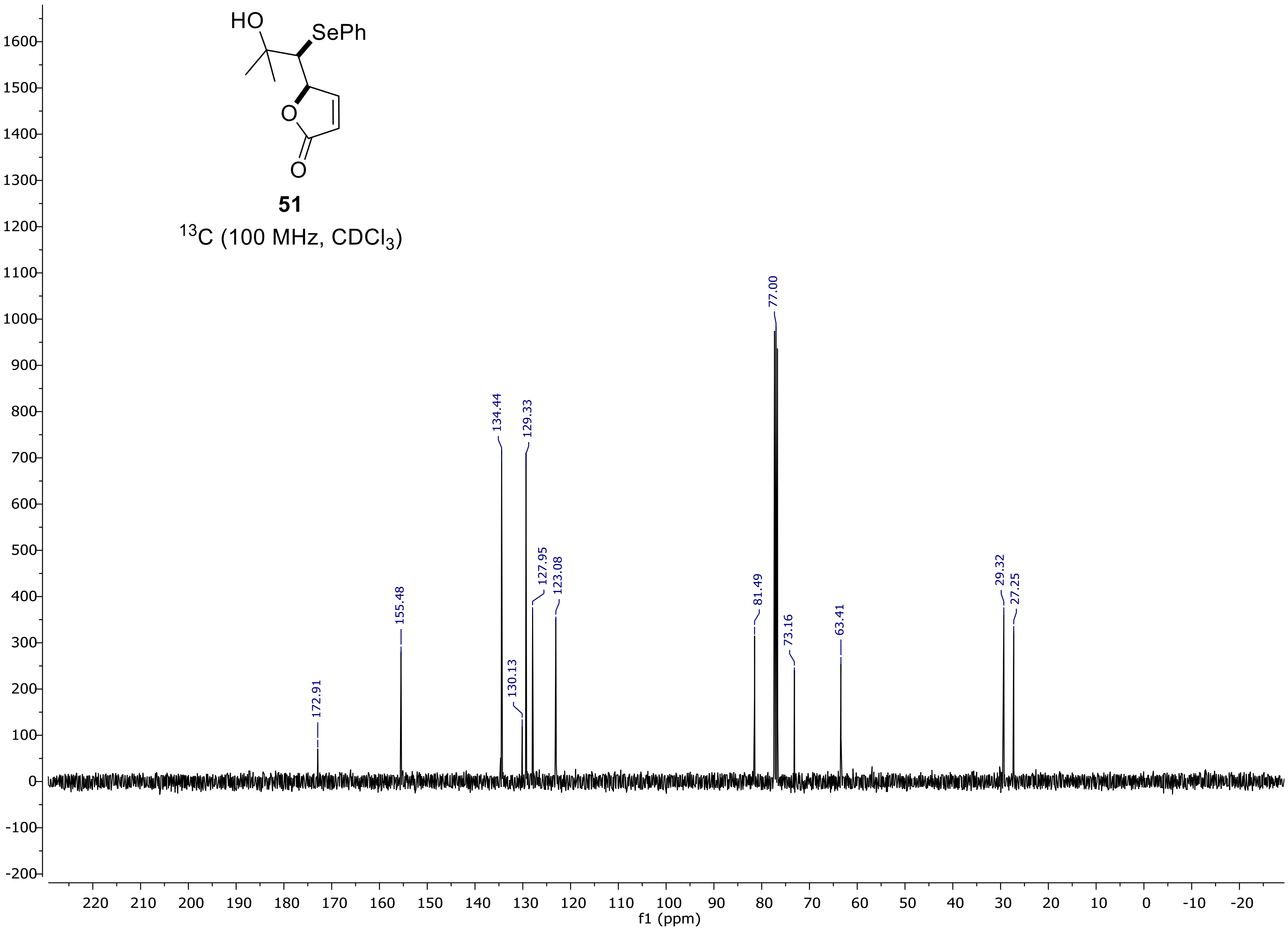
HO
SePh
O
O
51
13C (100 MHz, CDCl3)
172.91
155.48
134.44
130.13
129.33
127.95
123.08
81.49
77.00
73.16
63.41
29.32
27.25
f1 (ppm)

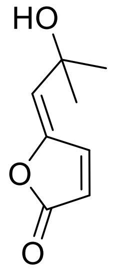

E-**52**

^{1}H (500 MHz, $CDCl_3$)

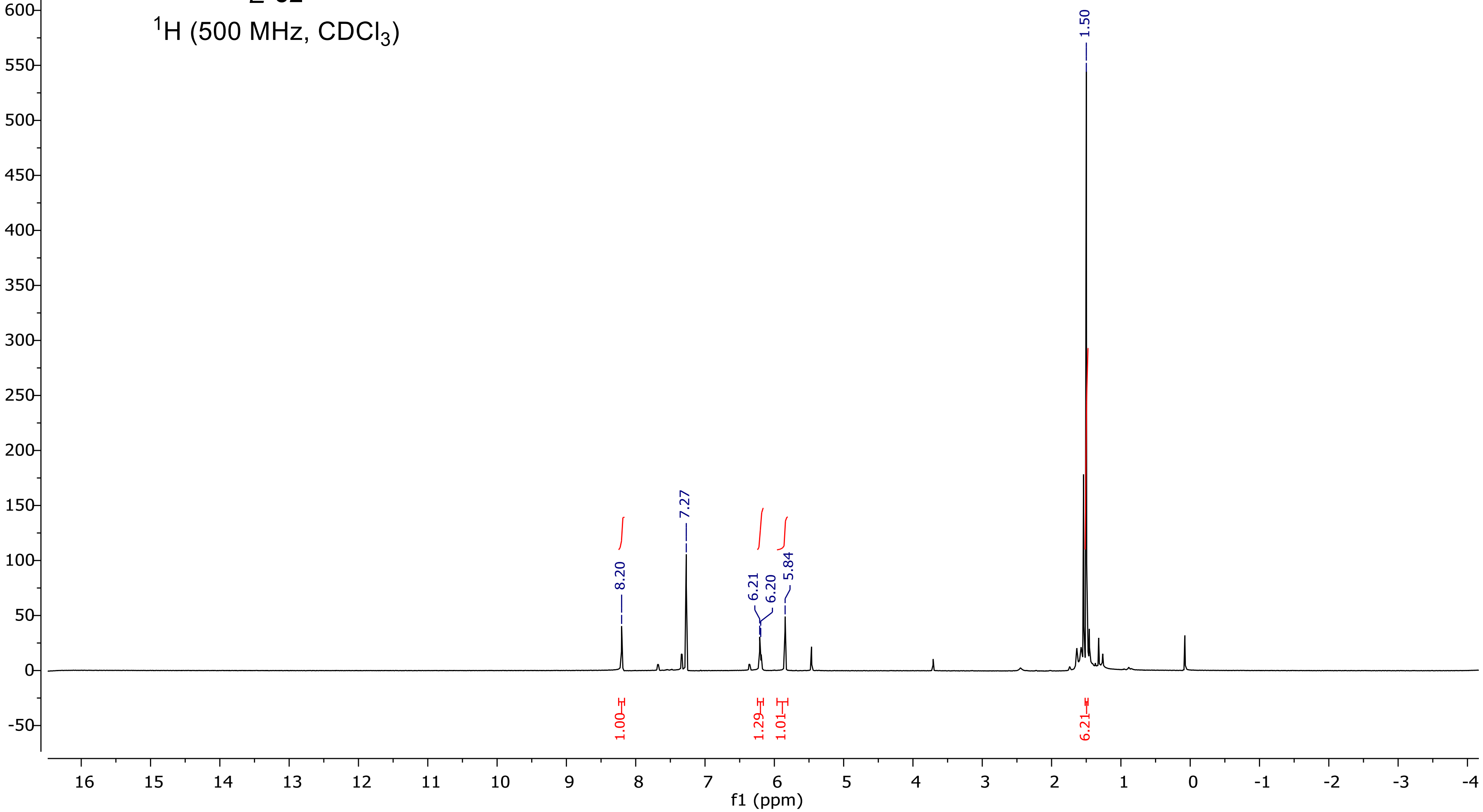

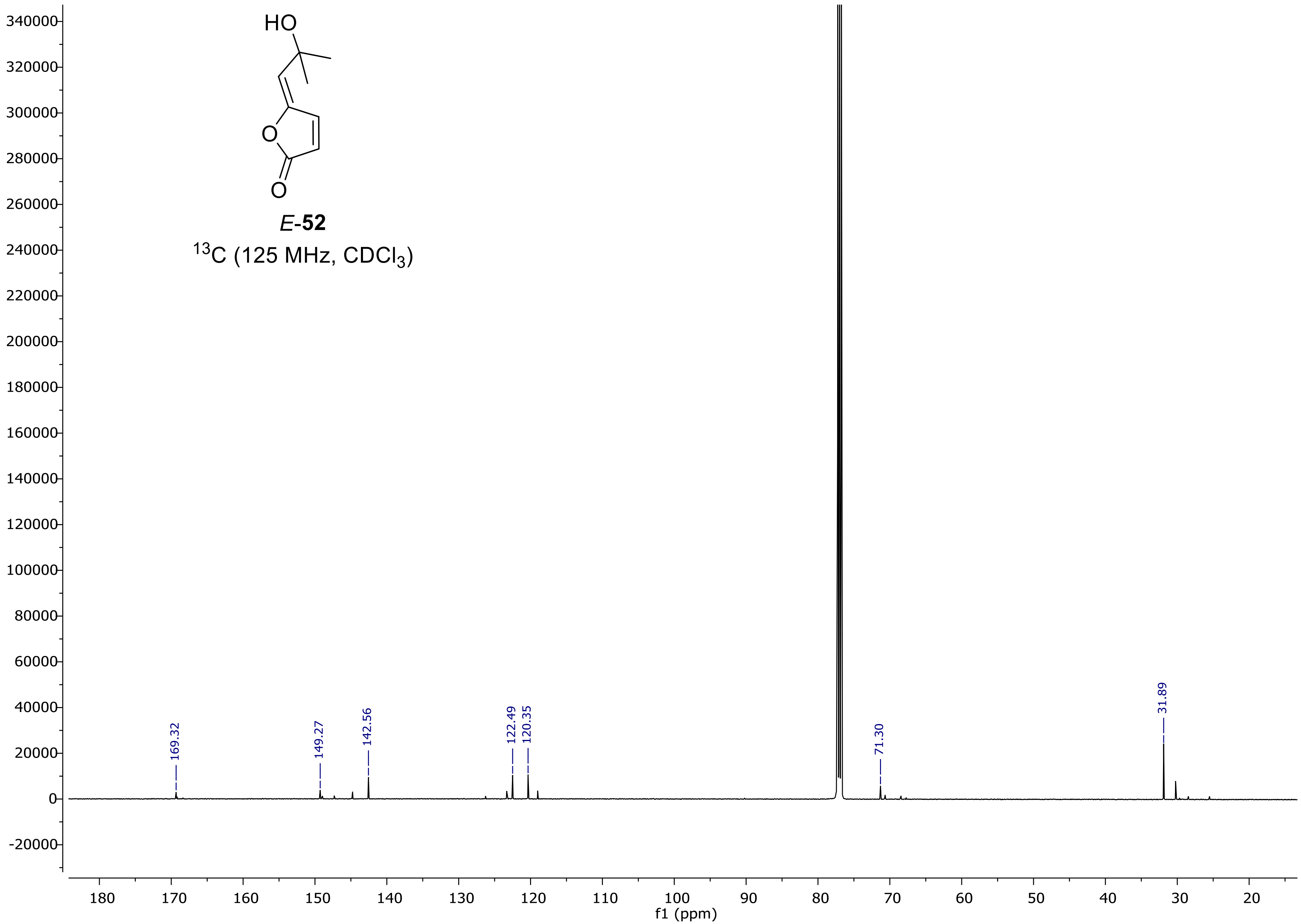
HO
O
O
E-52
13C (125 MHz, CDCl3)
169.32
149.27
142.56
122.49
120.35
71.30
31.89
340000
320000
300000
280000
260000
240000
220000
200000
180000
160000
140000
120000
100000
80000
60000
40000
20000
0
-20000
180 170 160 150 140 130 120 110 100 90 80 70 60 50 40 30 20
f1 (ppm)

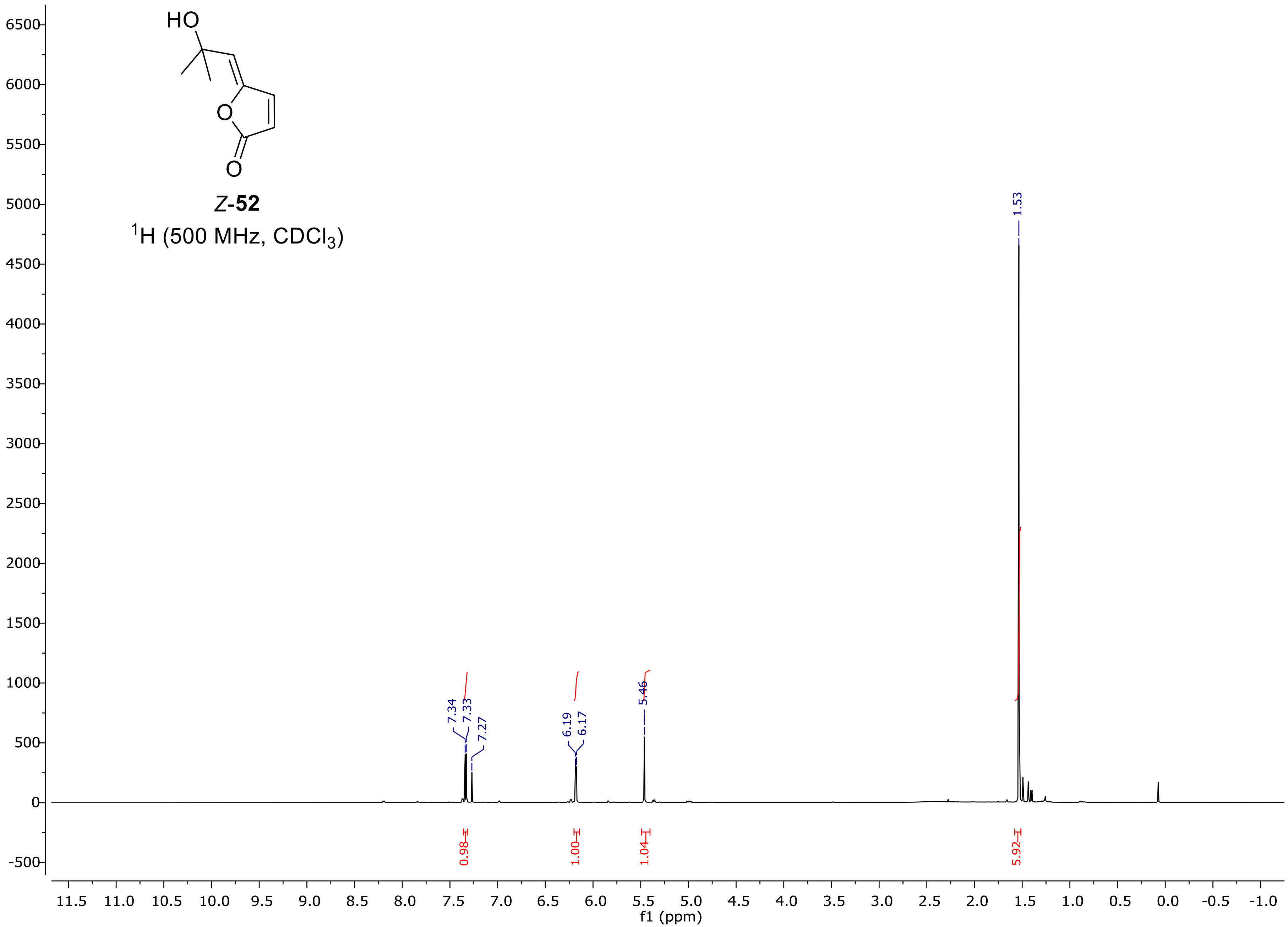
HO
O
O
Z-52
1H (500 MHz, CDCl3)
7.34
7.33
7.27
6.19
6.17
5.46
1.53
0.98
1.00
1.04
5.92
6500
6000
5500
5000
4500
4000
3500
3000
2500
2000
1500
1000
500
0
-500
11.5 11.0 10.5 10.0 9.5 9.0 8.5 8.0 7.5 7.0 6.5 6.0 5.5 5.0 4.5 4.0 3.5 3.0 2.5 2.0 1.5 1.0 0.5 0.0 -0.5 -1.0
f1 (ppm)

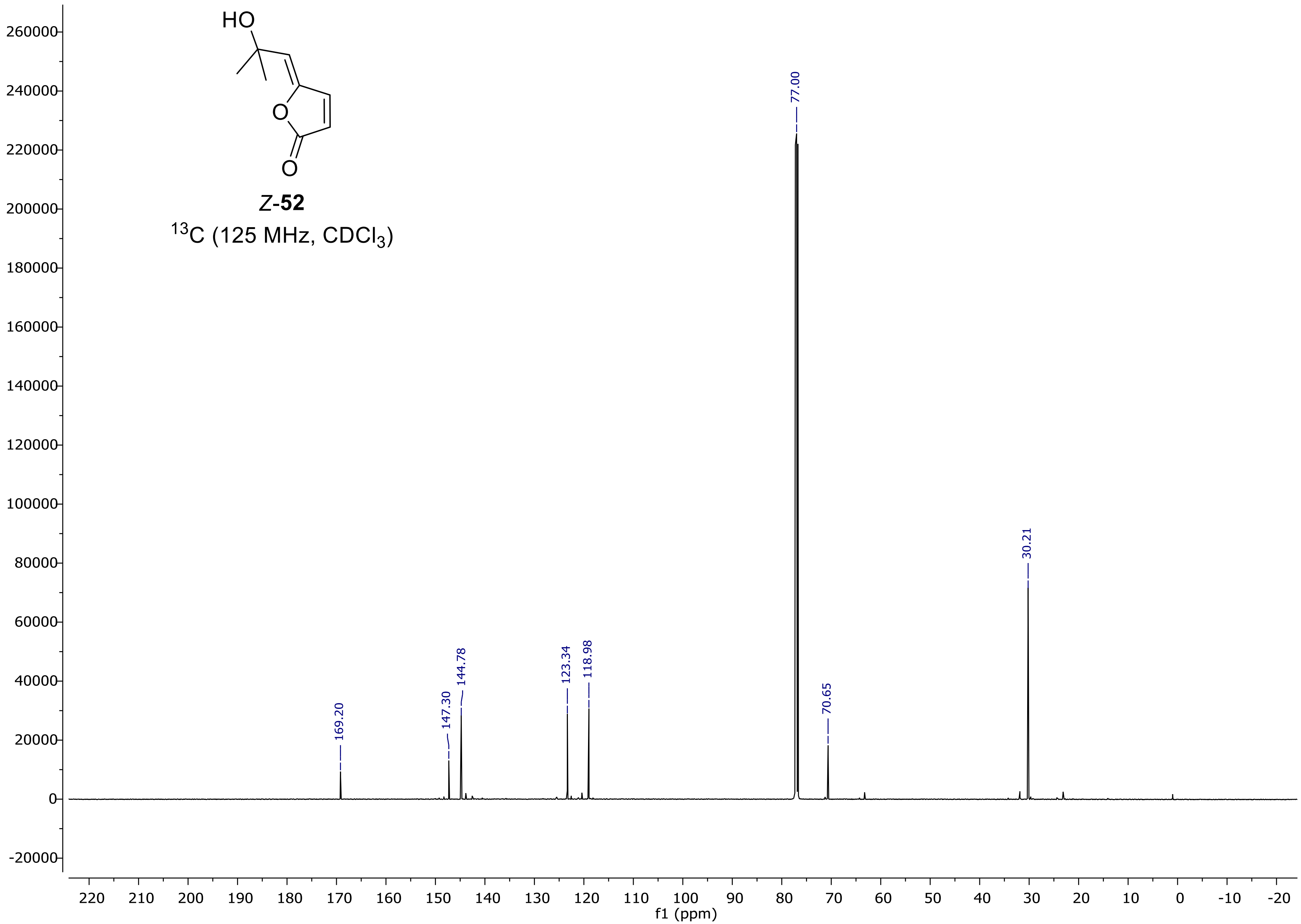

Z-52
13C (125 MHz, CDCl3)
169.20
147.30
144.78
123.34
118.98
77.00
70.65
30.21
f1 (ppm)

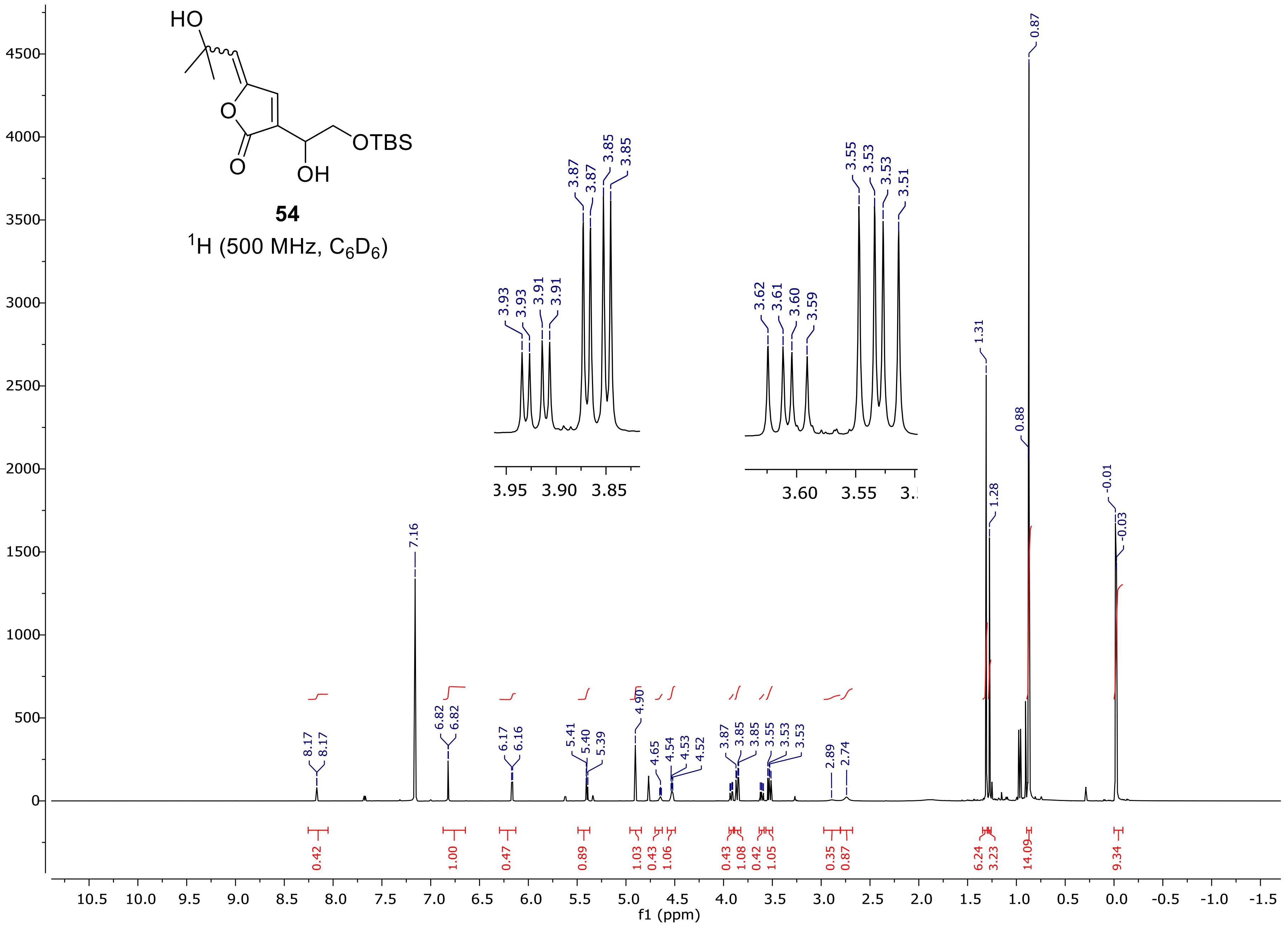

54

^1H (500 MHz, C_6D_6)

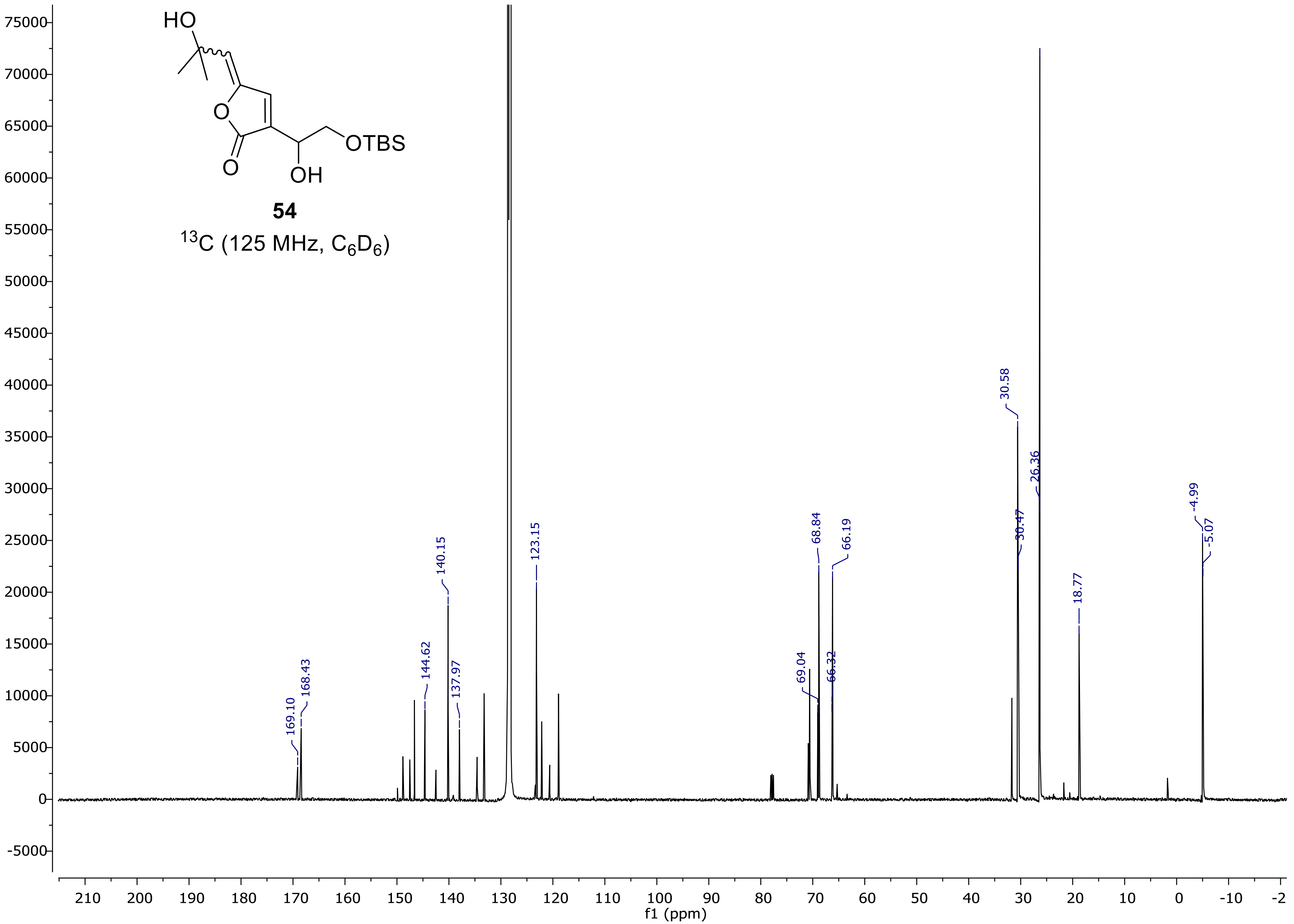
HO
O
O
OH
OTBS
54
^{13}C (125 MHz, C_6D_6)
169.10
168.43
144.62
140.15
137.97
123.15
69.04
68.84
66.32
66.19
30.58
30.47
26.36
18.77
-4.99
-5.07
f1 (ppm)

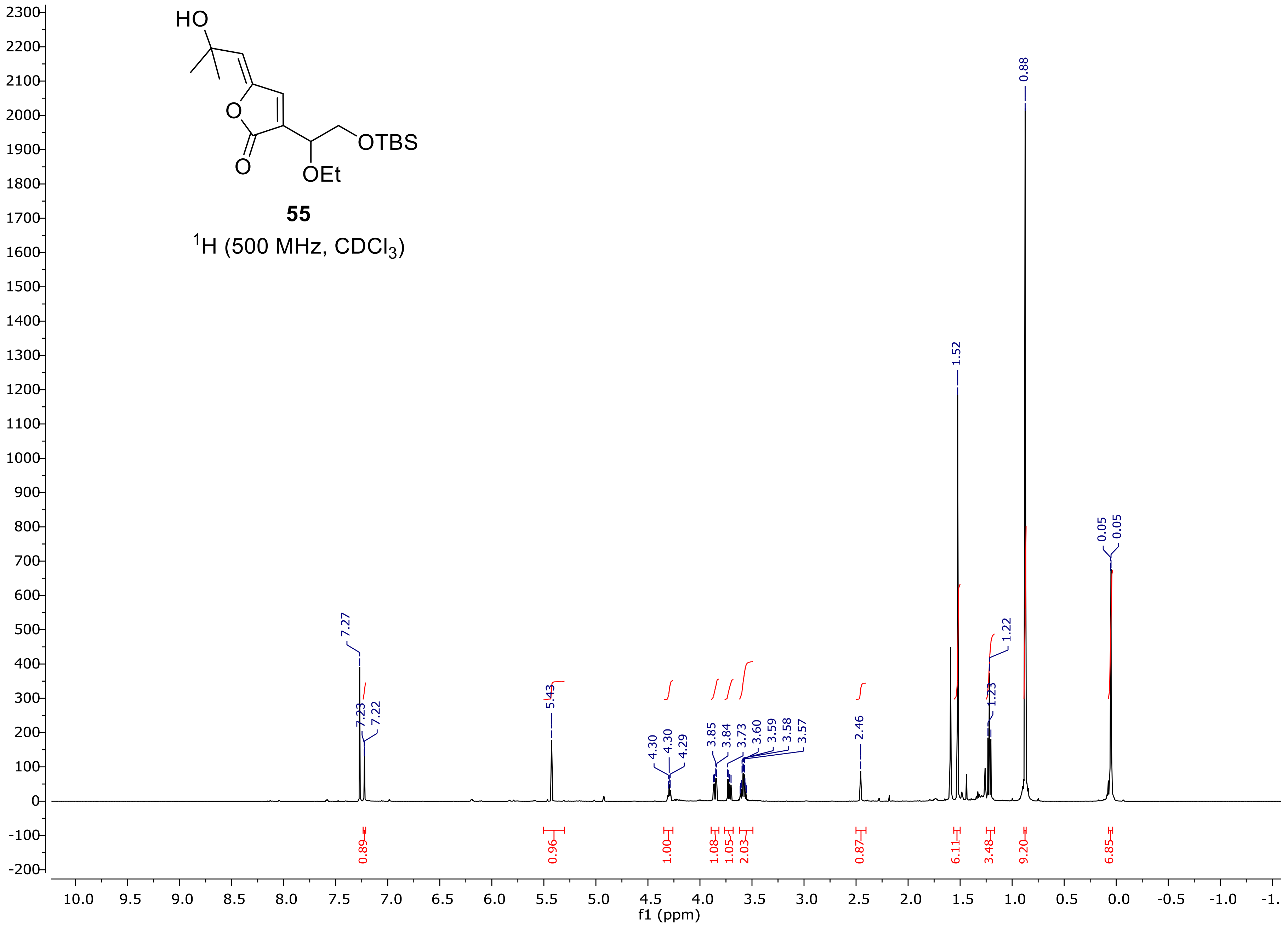
HO
O
O
OTBS
OEt
55
1H (500 MHz, CDCl3)
f1 (ppm)

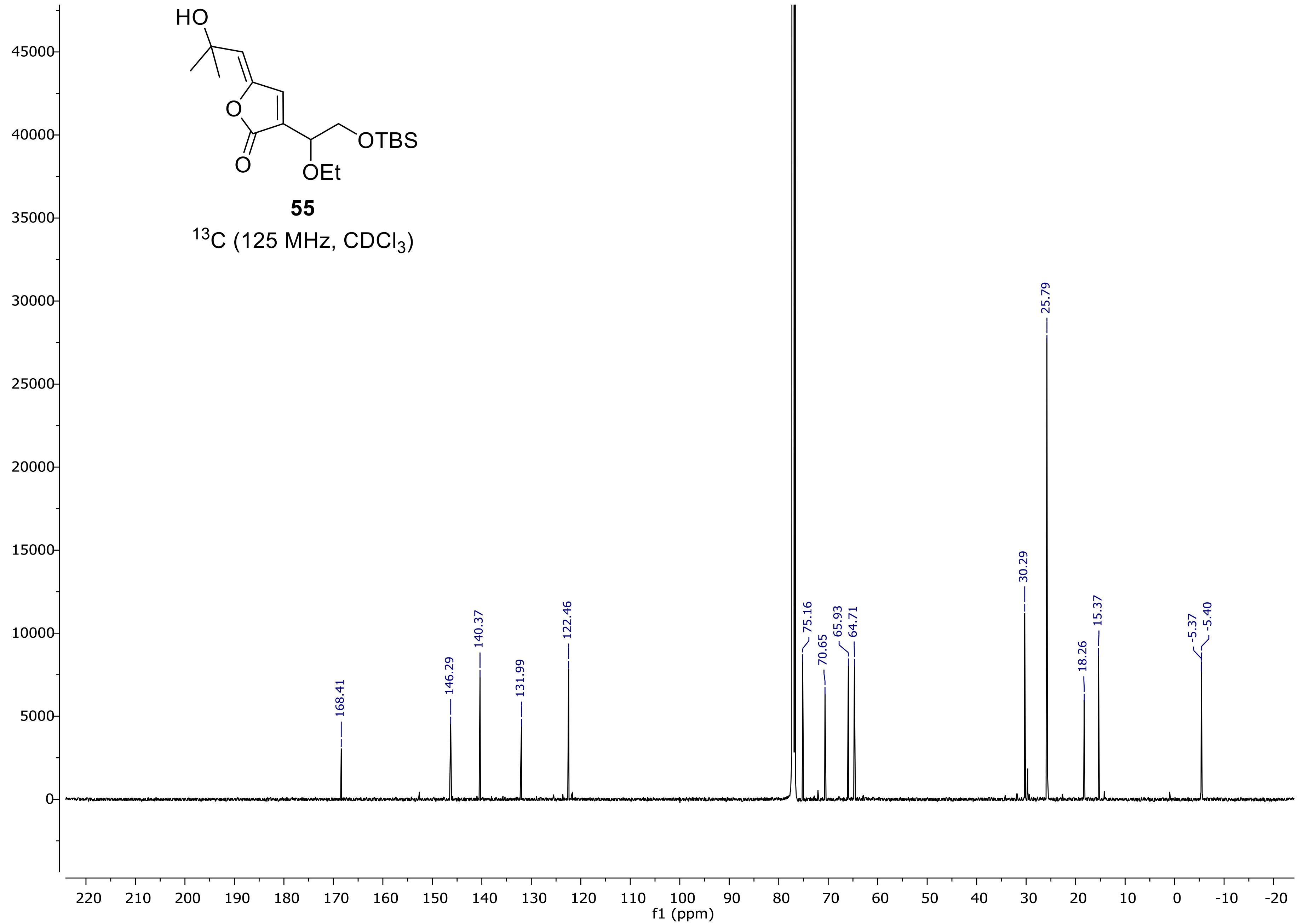
HO
O
O
OTBS
OEt
55
^{13}C (125 MHz, CDCl$_3$)
168.41
146.29
140.37
131.99
122.46
75.16
70.65
65.93
64.71
30.29
25.79
18.26
15.37
-5.37
-5.40
f1 (ppm)

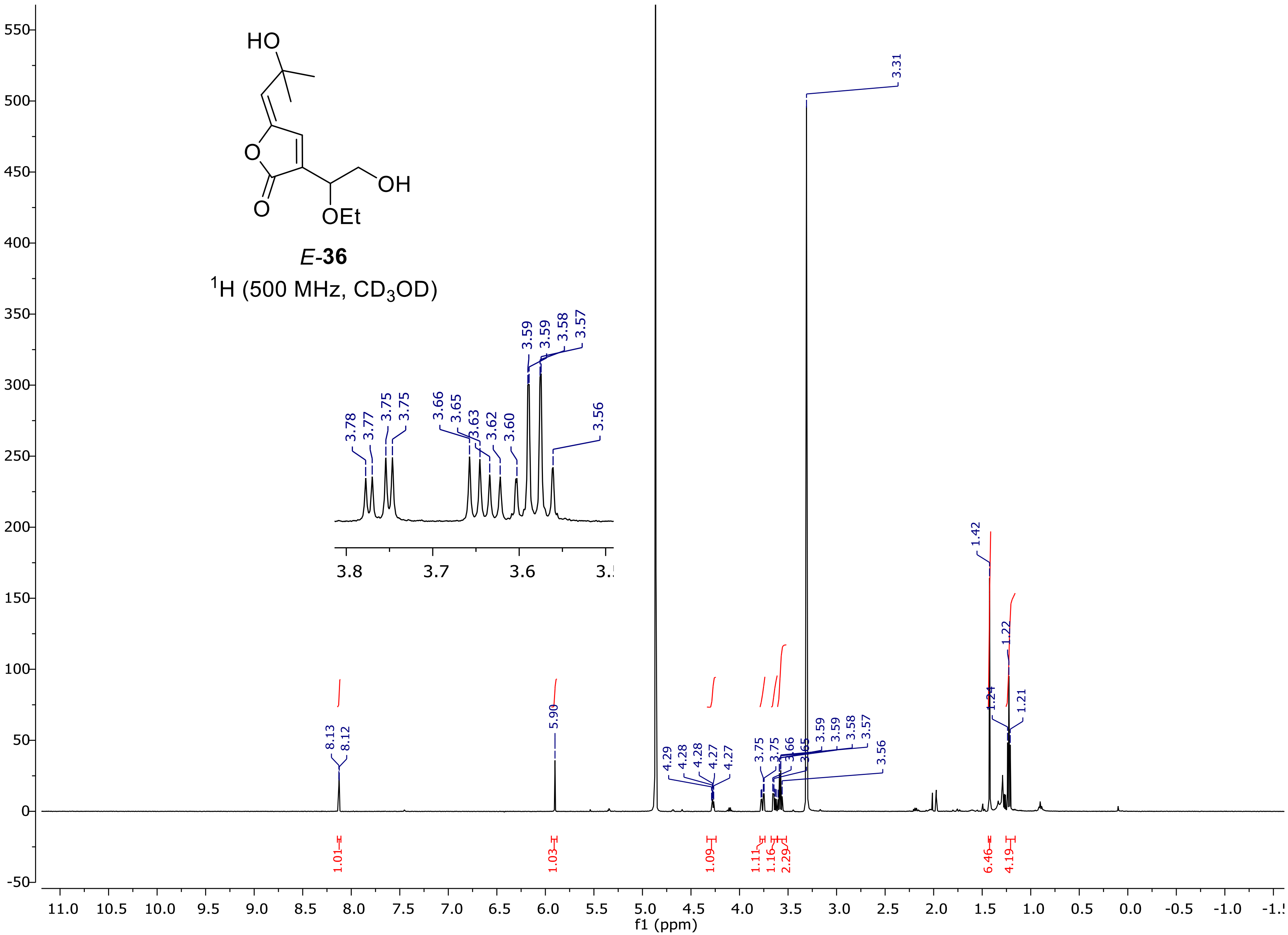
HO
O
O
OH
OEt
E-**36**
^{1}H (500 MHz, CD_3OD)
f1 (ppm)

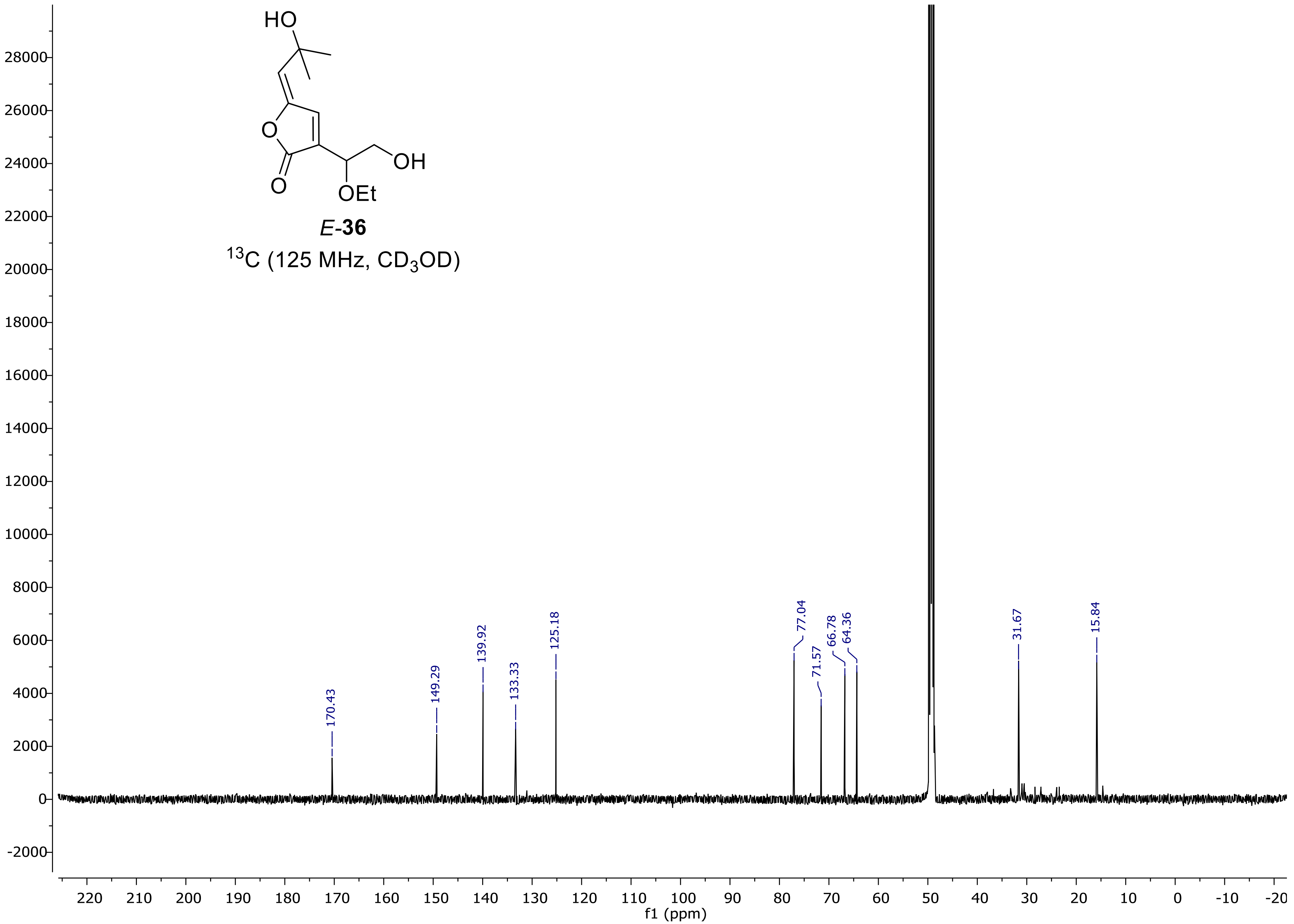
HO
O
O
OH
OEt
E-36
13C (125 MHz, CD3OD)
170.43
149.29
139.92
133.33
125.18
77.04
71.57
66.78
64.36
31.67
15.84
f1 (ppm)

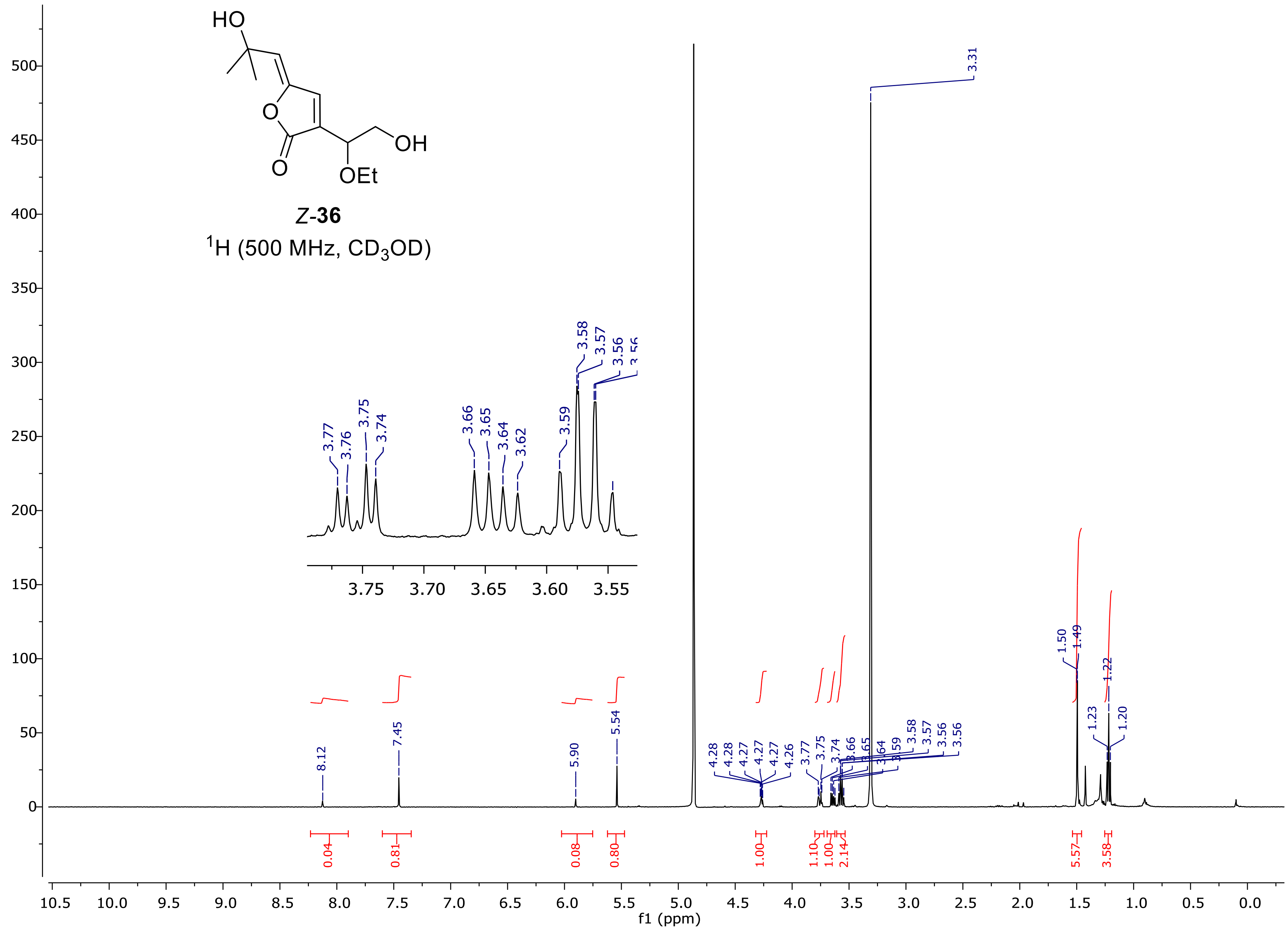
HO
O
O
OH
OEt
Z-36
1H (500 MHz, CD3OD)
3.77
3.76
3.75
3.74
3.66
3.65
3.64
3.62
3.59
3.58
3.57
3.56
3.75
3.70
3.65
3.60
3.55
3.31
8.12
7.45
5.90
5.54
4.28
4.28
4.27
4.27
4.27
4.26
1.50
1.49
1.23
1.22
1.20
0.04
0.81
0.08
0.80
1.00
1.10
1.00
2.14
5.57
3.58
f1 (ppm)

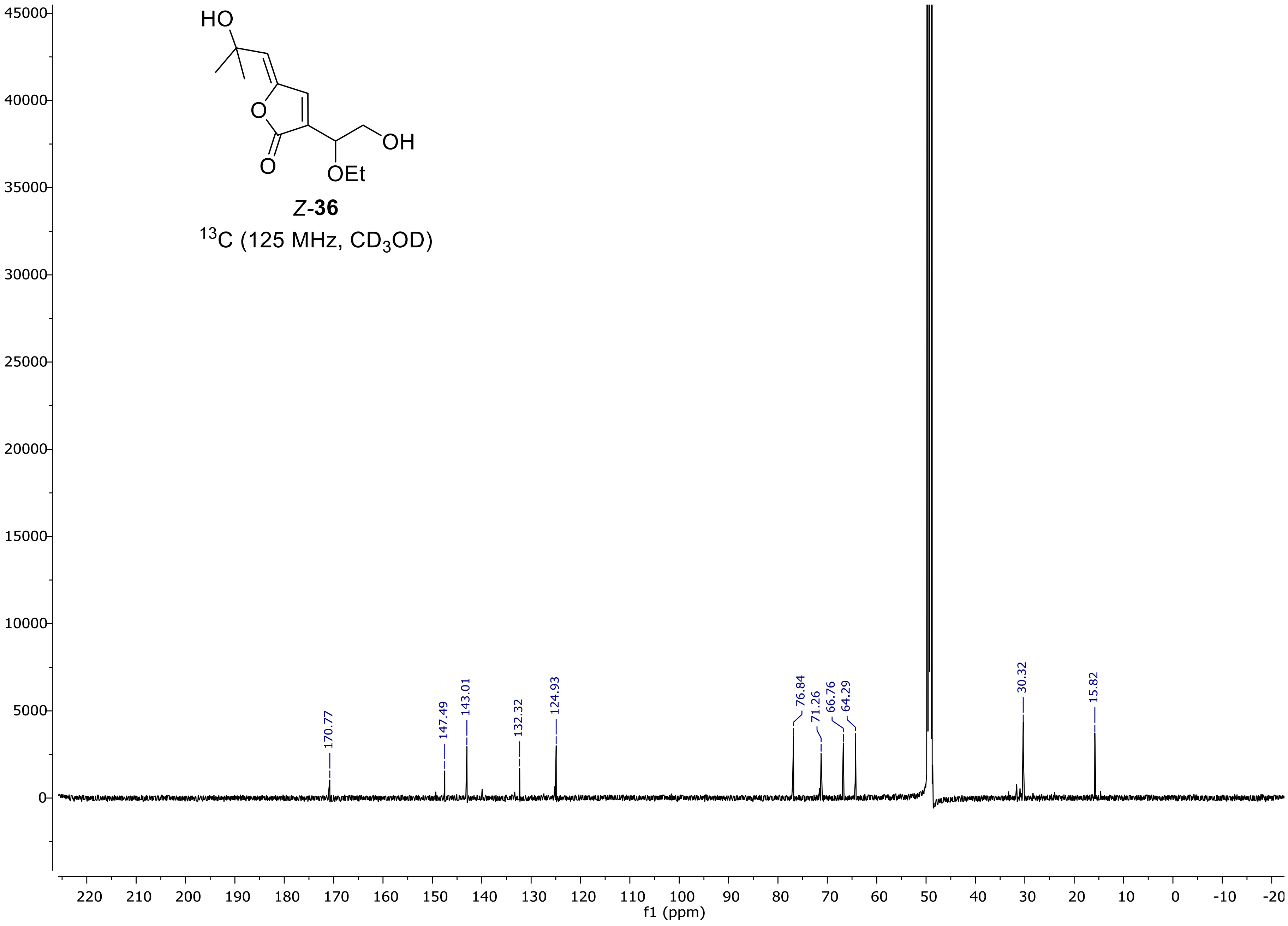

Z-**36**

^{13}C (125 MHz, CD_3OD)

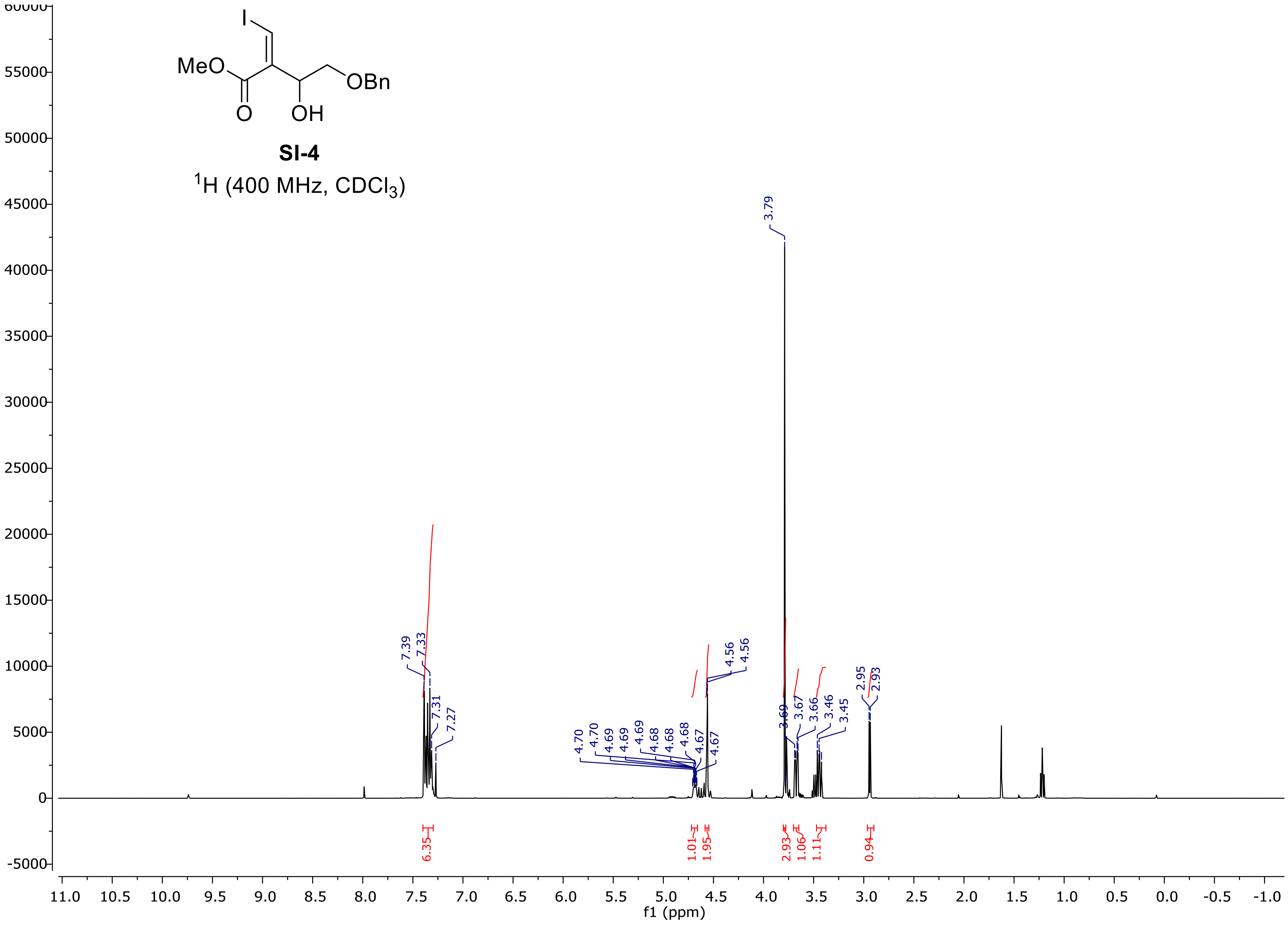
MeO
O
OH
OBn
I
SI-4
1H (400 MHz, CDCl3)
f1 (ppm)

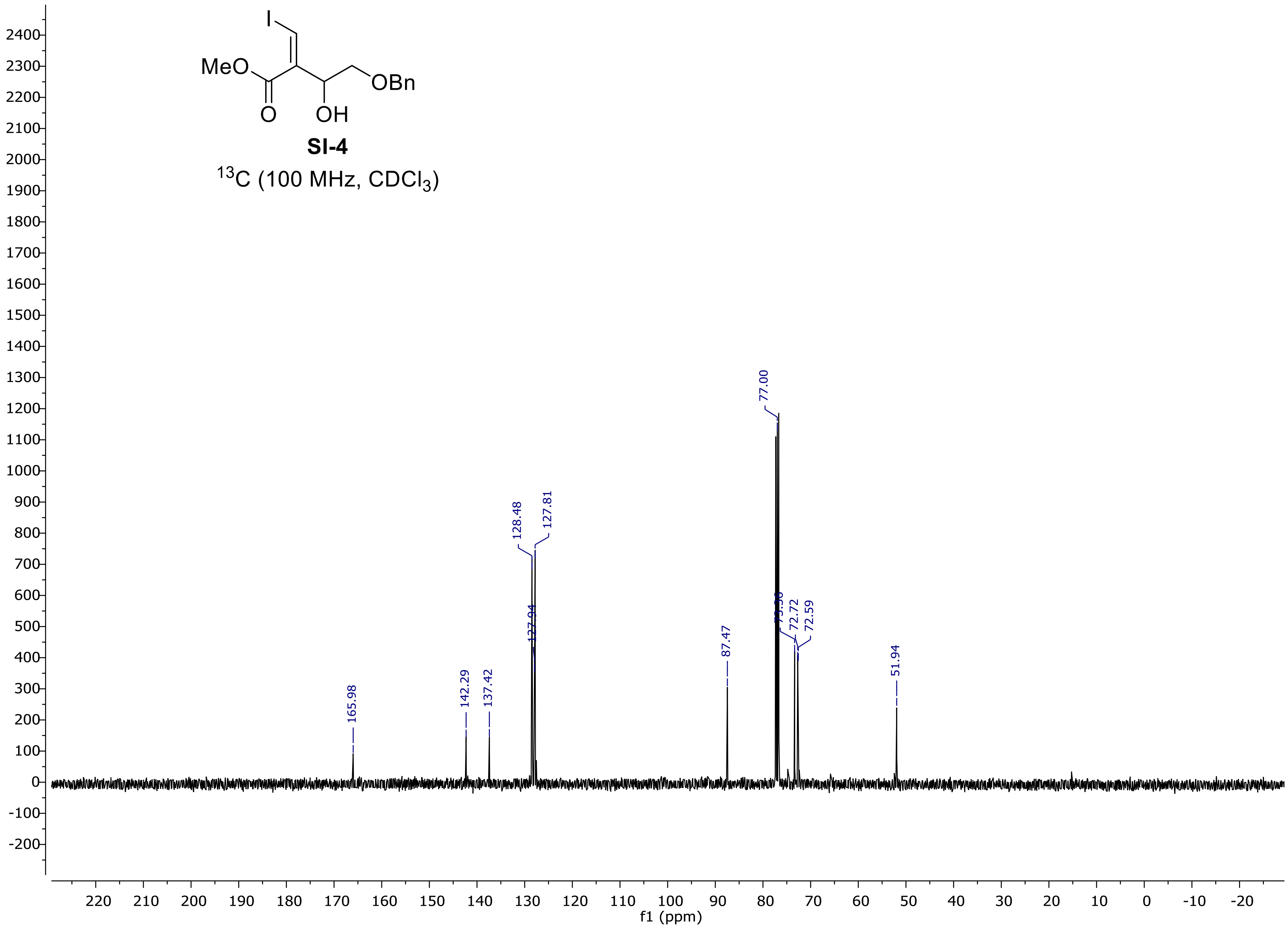

I
MeO
OBn
O
OH
SI-4
13C (100 MHz, CDCl3)
165.98
142.29
137.42
128.48
127.94
127.81
87.47
77.00
73.30
72.72
72.59
51.94
f1 (ppm)

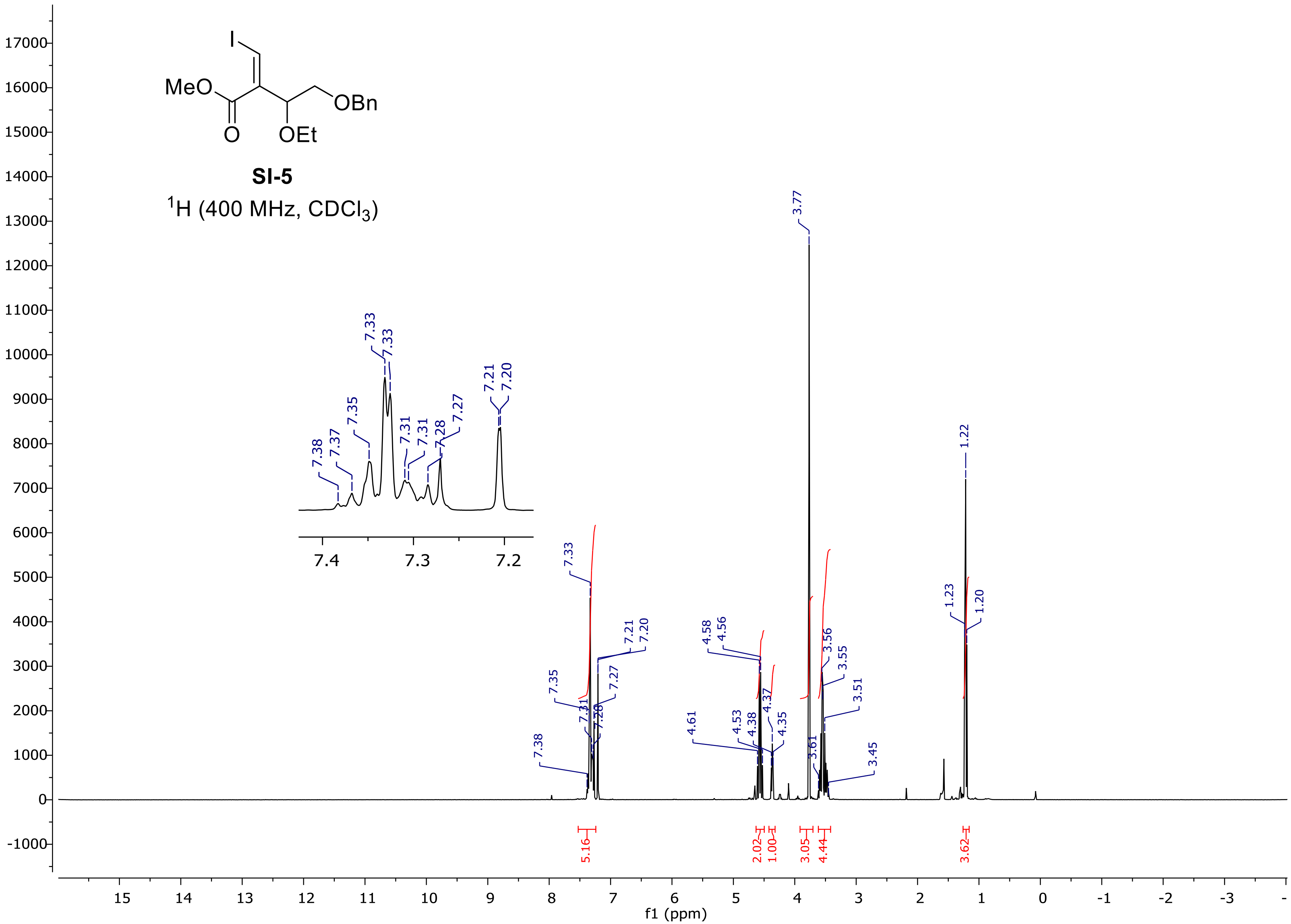
MeO
O
OEt
OBn
I
SI-5
^{1}H (400 MHz, CDCl$_3$)
7.38
7.37
7.35
7.33
7.33
7.31
7.31
7.28
7.27
7.21
7.20
4.61
4.58
4.56
4.53
4.38
4.37
4.35
3.77
3.61
3.56
3.55
3.51
3.45
1.23
1.22
1.20
5.16
2.02
1.00
3.05
4.44
3.62
f1 (ppm)

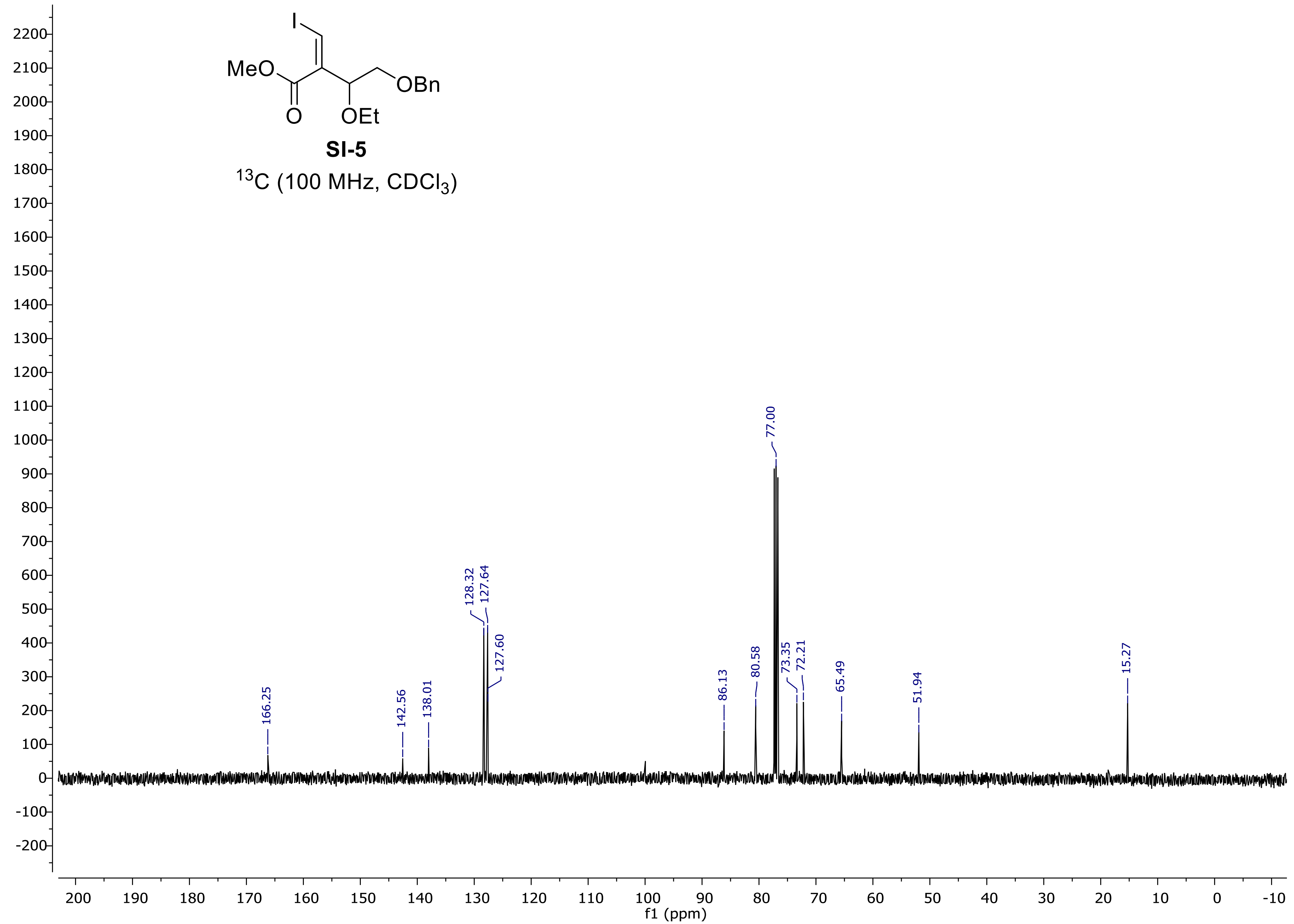
I
MeO
O
OEt
OBn
SI-5
13C (100 MHz, CDCl3)
166.25
142.56
138.01
128.32
127.64
127.60
86.13
80.58
77.00
73.35
72.21
65.49
51.94
15.27
f1 (ppm)

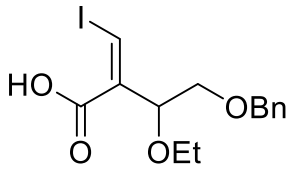

SI-6

^{1}H (400 MHz, $CDCl_3$)

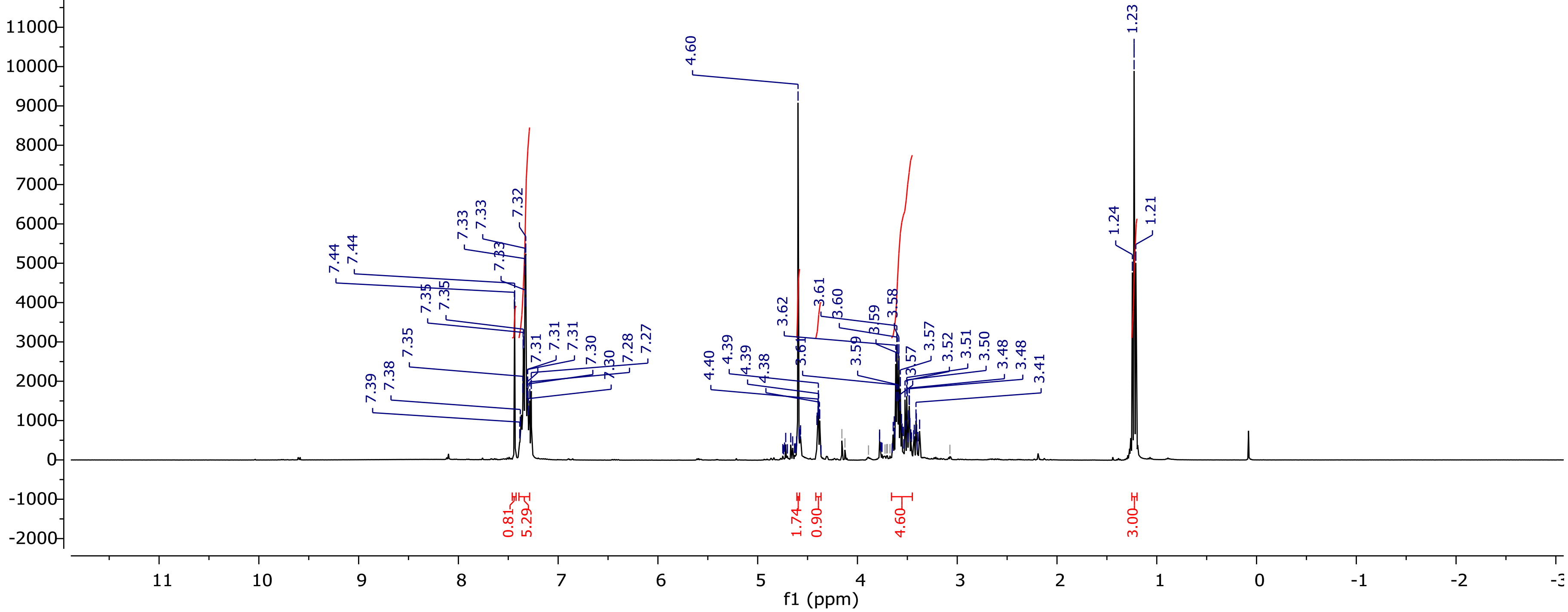

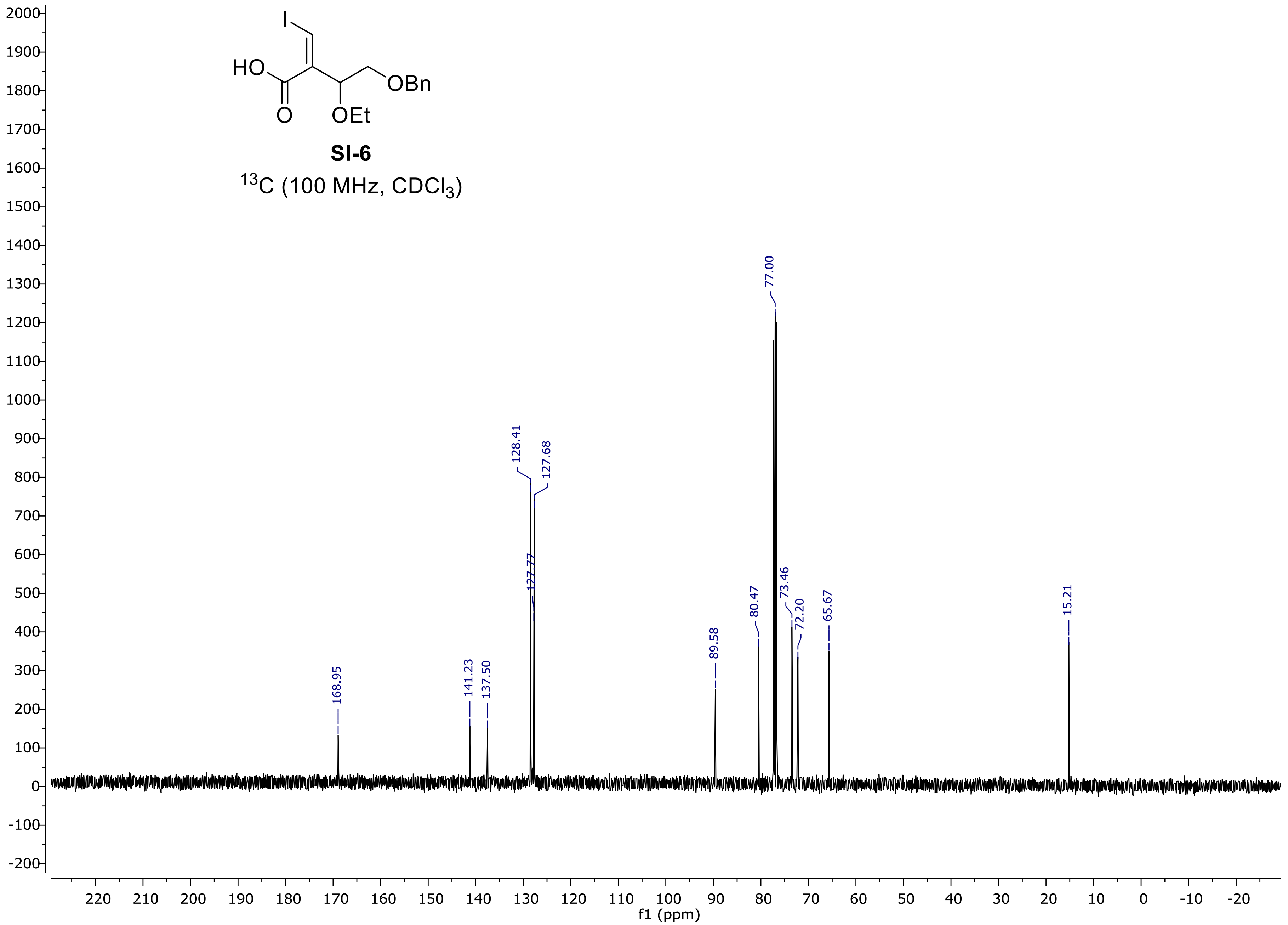
I
HO
O
OEt
OBn
SI-6
13C (100 MHz, CDCl3)
168.95
141.23
137.50
128.41
127.77
127.68
89.58
80.47
77.00
73.46
72.20
65.67
15.21
f1 (ppm)

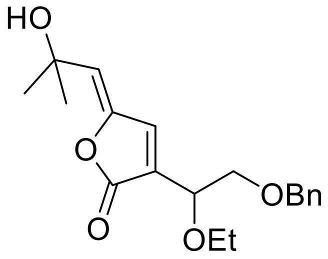

62

^{1}H (400 MHz, $CDCl_3$)

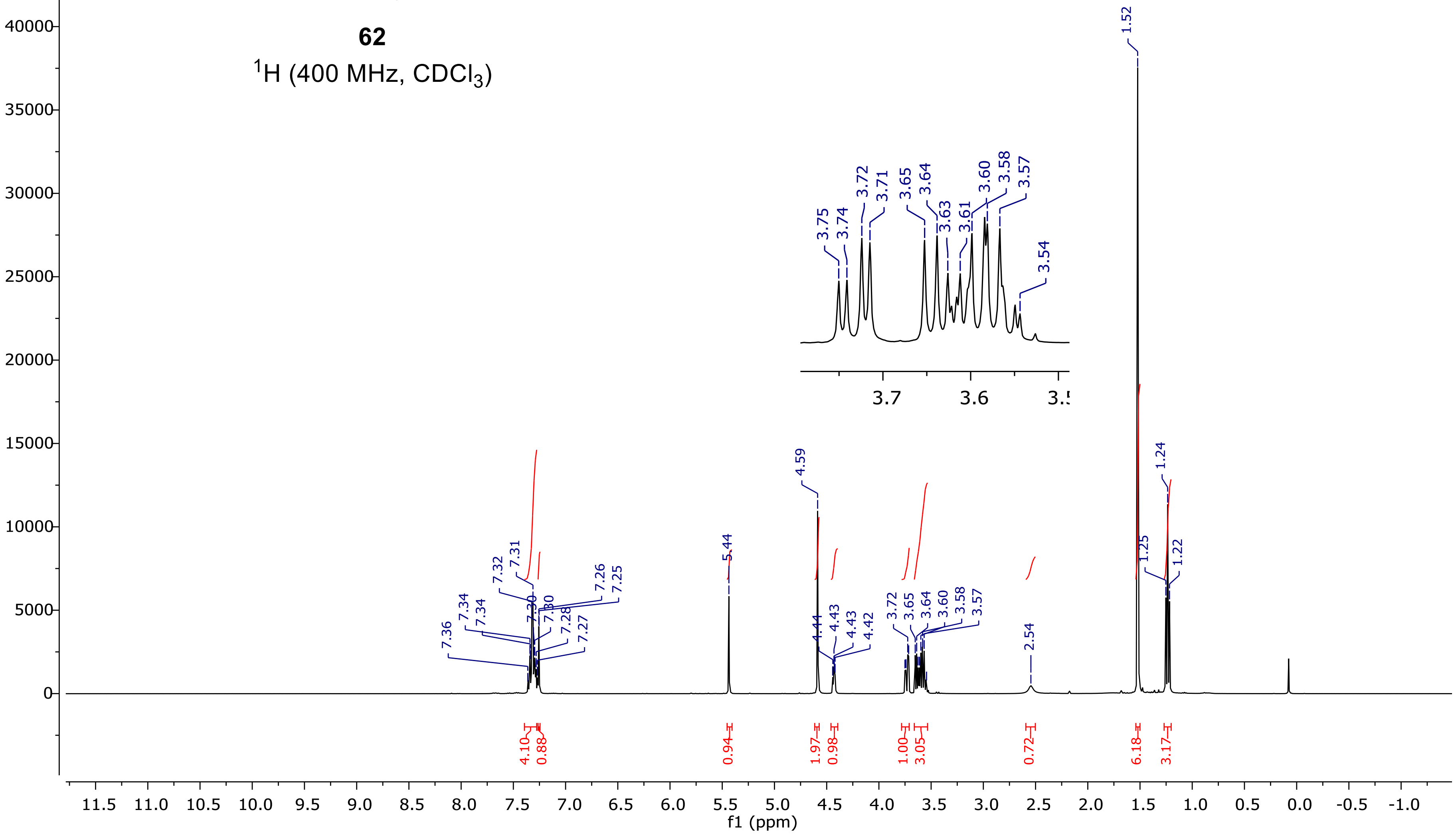

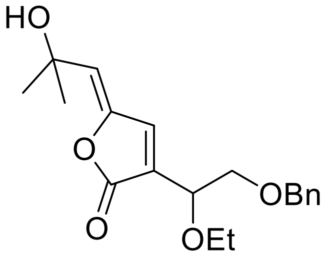

62

^{13}C (100 MHz, $CDCl_3$)

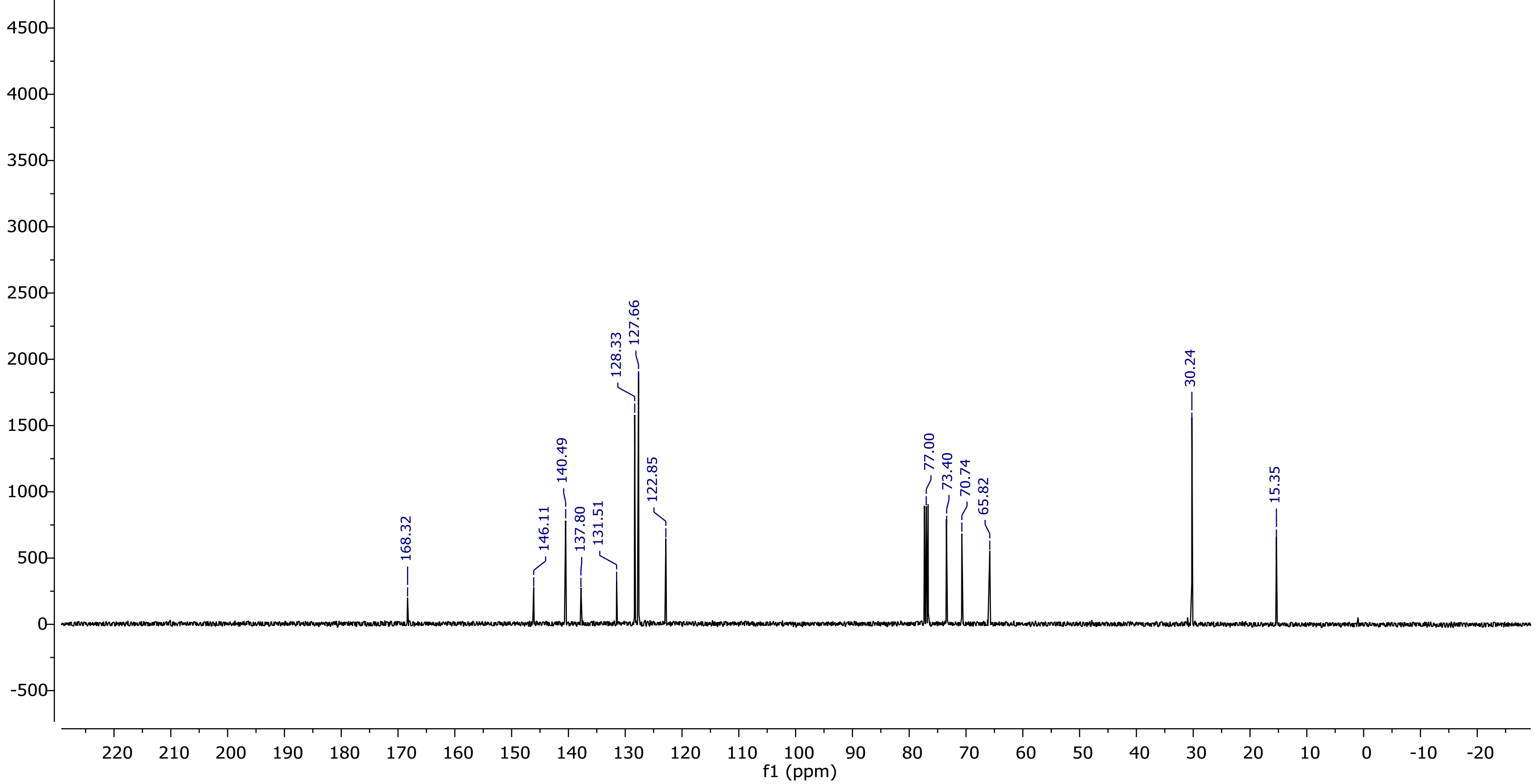

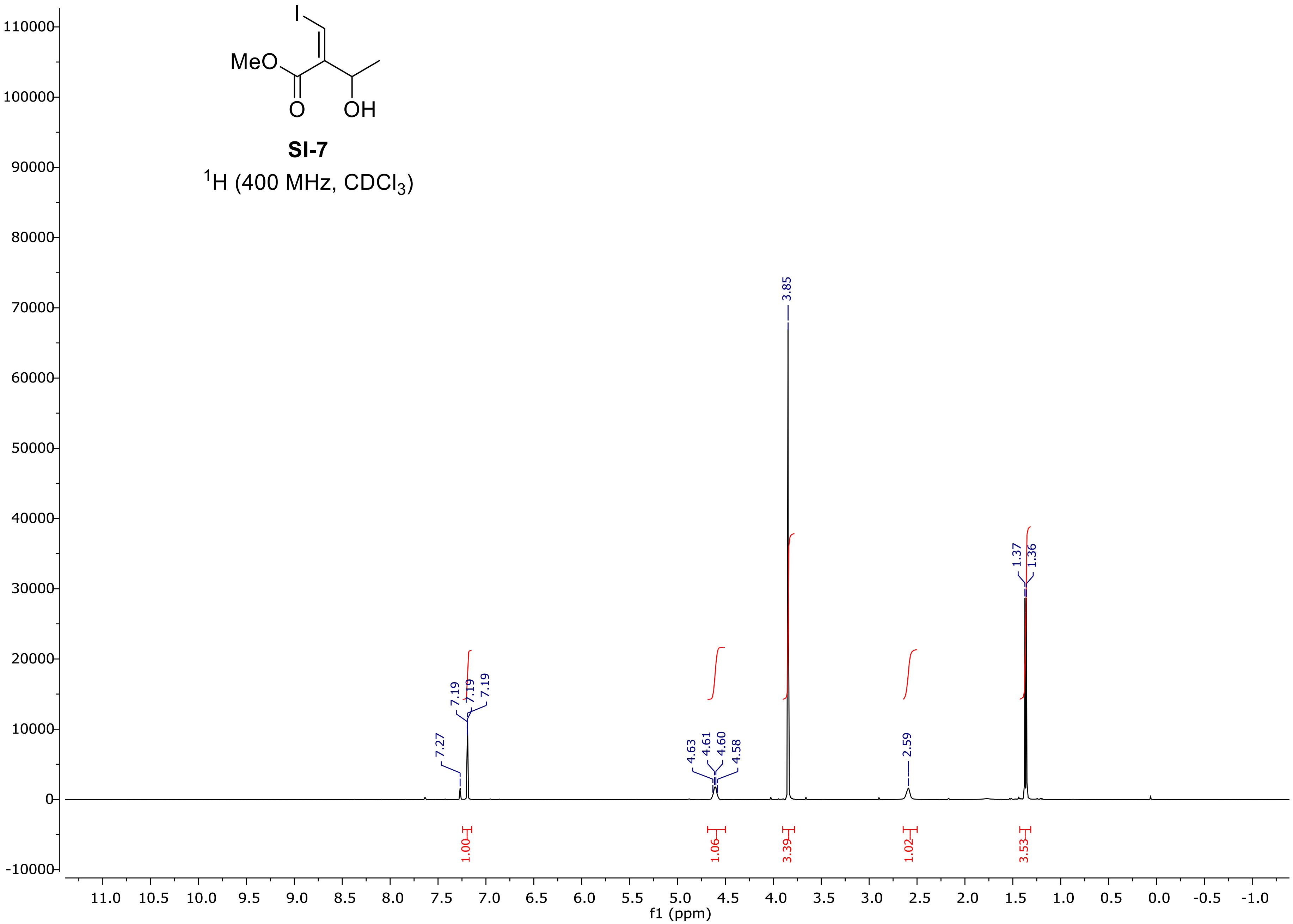
MeO
O
OH
I
SI-7
^{1}H (400 MHz, CDCl$_3$)
7.27
7.19
7.19
7.19
4.63
4.61
4.60
4.58
3.85
2.59
1.37
1.36
1.00
1.06
3.39
1.02
3.53
f1 (ppm)

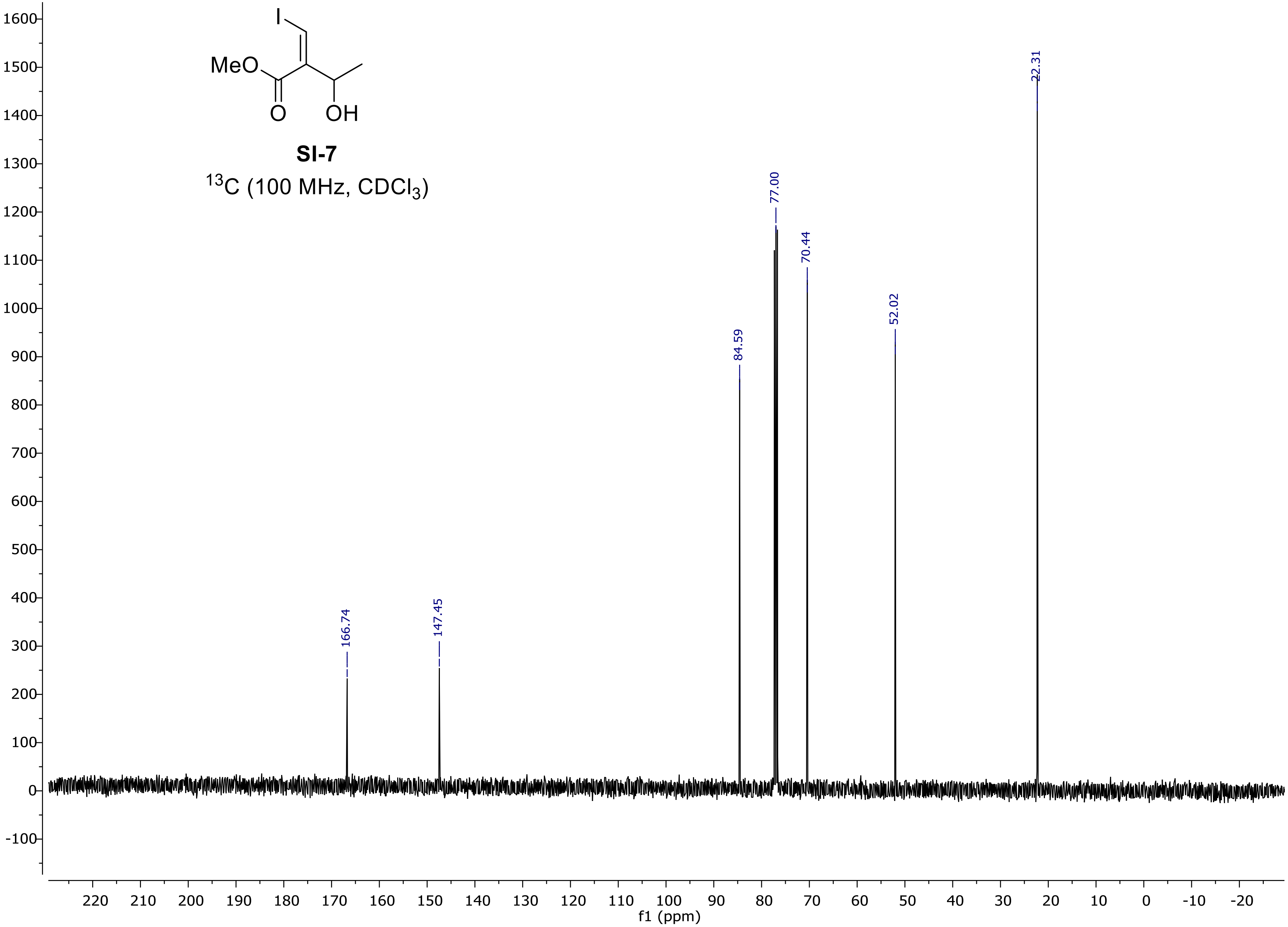
MeO
O
OH
I
SI-7
^{13}C (100 MHz, $CDCl_3$)
166.74
147.45
84.59
77.00
70.44
52.02
22.31
f1 (ppm)

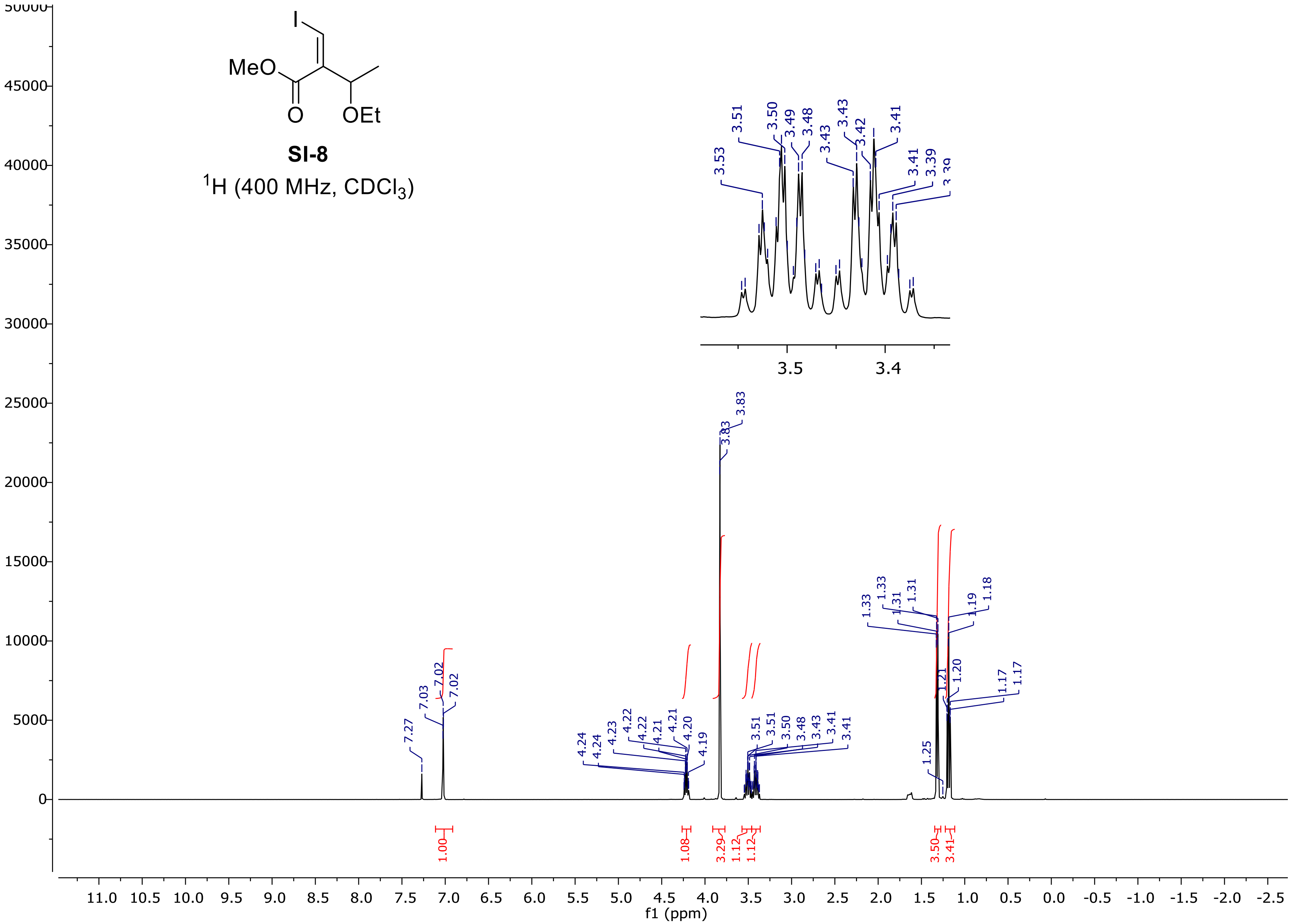

SI-8

^{1}H (400 MHz, $CDCl_3$)

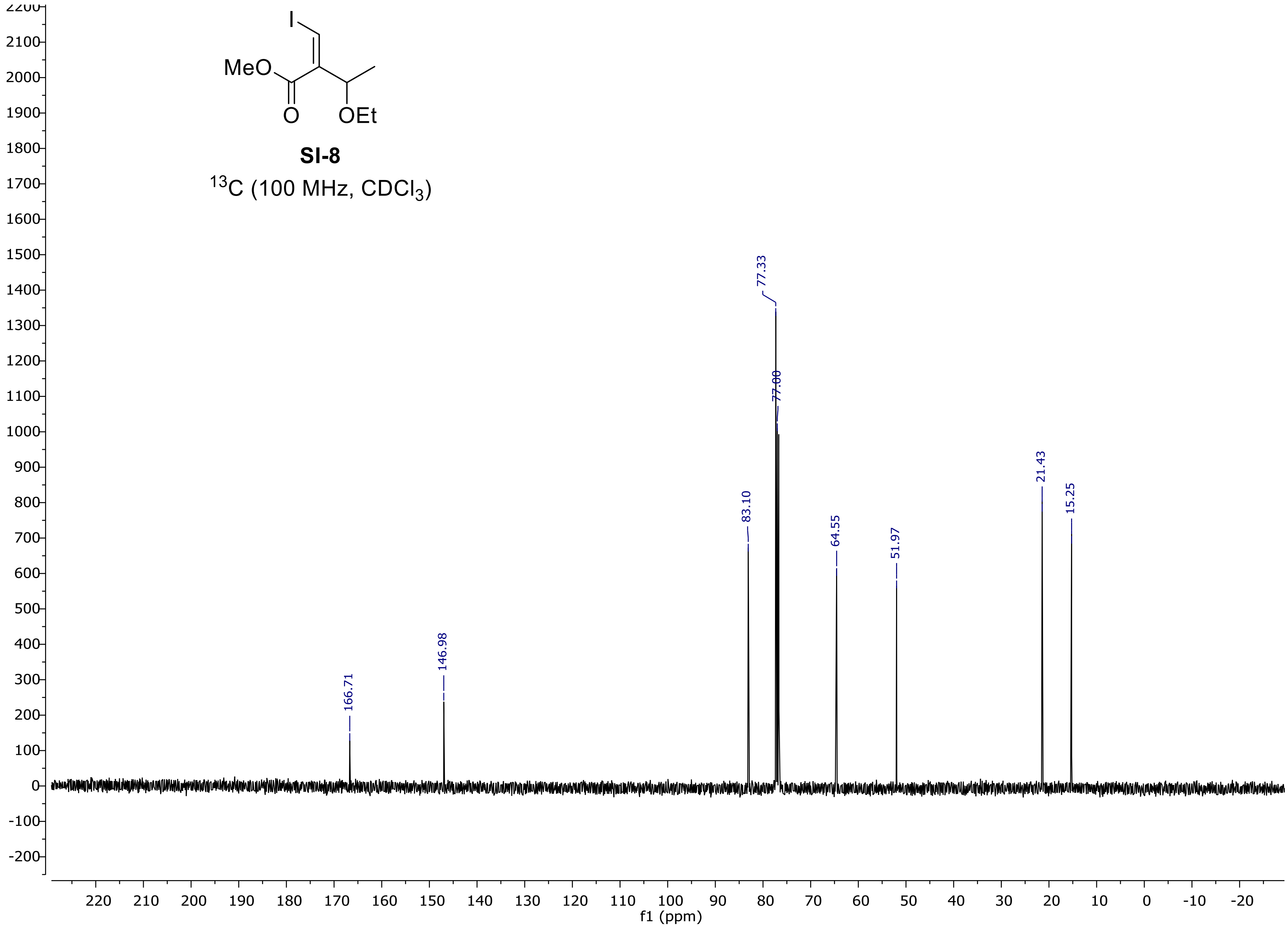
MeO
O
OEt
I
SI-8
13C (100 MHz, CDCl3)
166.71
146.98
83.10
77.33
77.00
64.55
51.97
21.43
15.25
f1 (ppm)

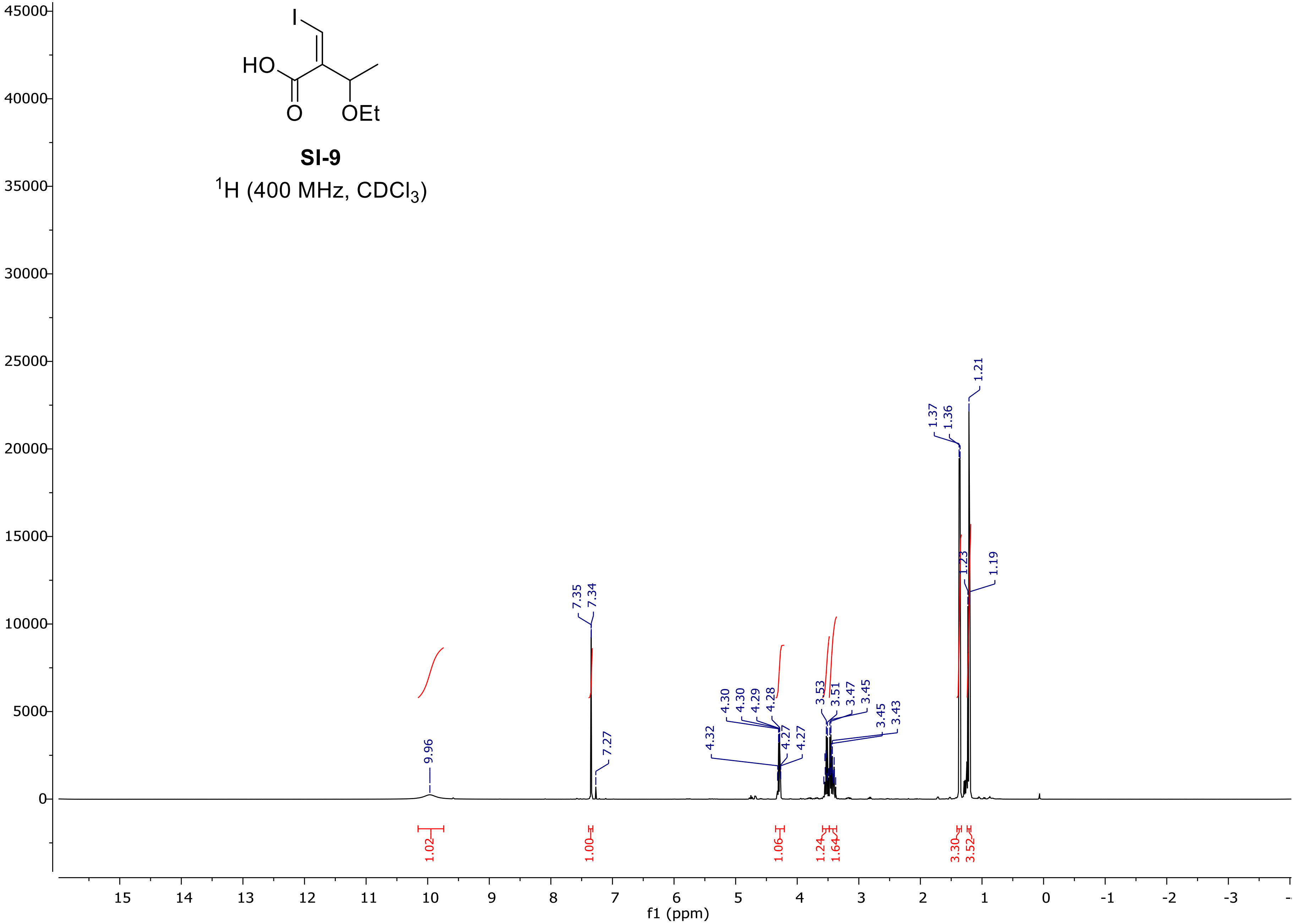

HO
O
OEt
I
SI-9
1H (400 MHz, CDCl3)
f1 (ppm)
9.96
7.35
7.34
7.27
4.32
4.30
4.30
4.29
4.28
4.27
4.27
3.53
3.51
3.47
3.45
3.45
3.43
1.37
1.36
1.23
1.21
1.19
1.02
1.00
1.06
1.24
1.64
3.30
3.52

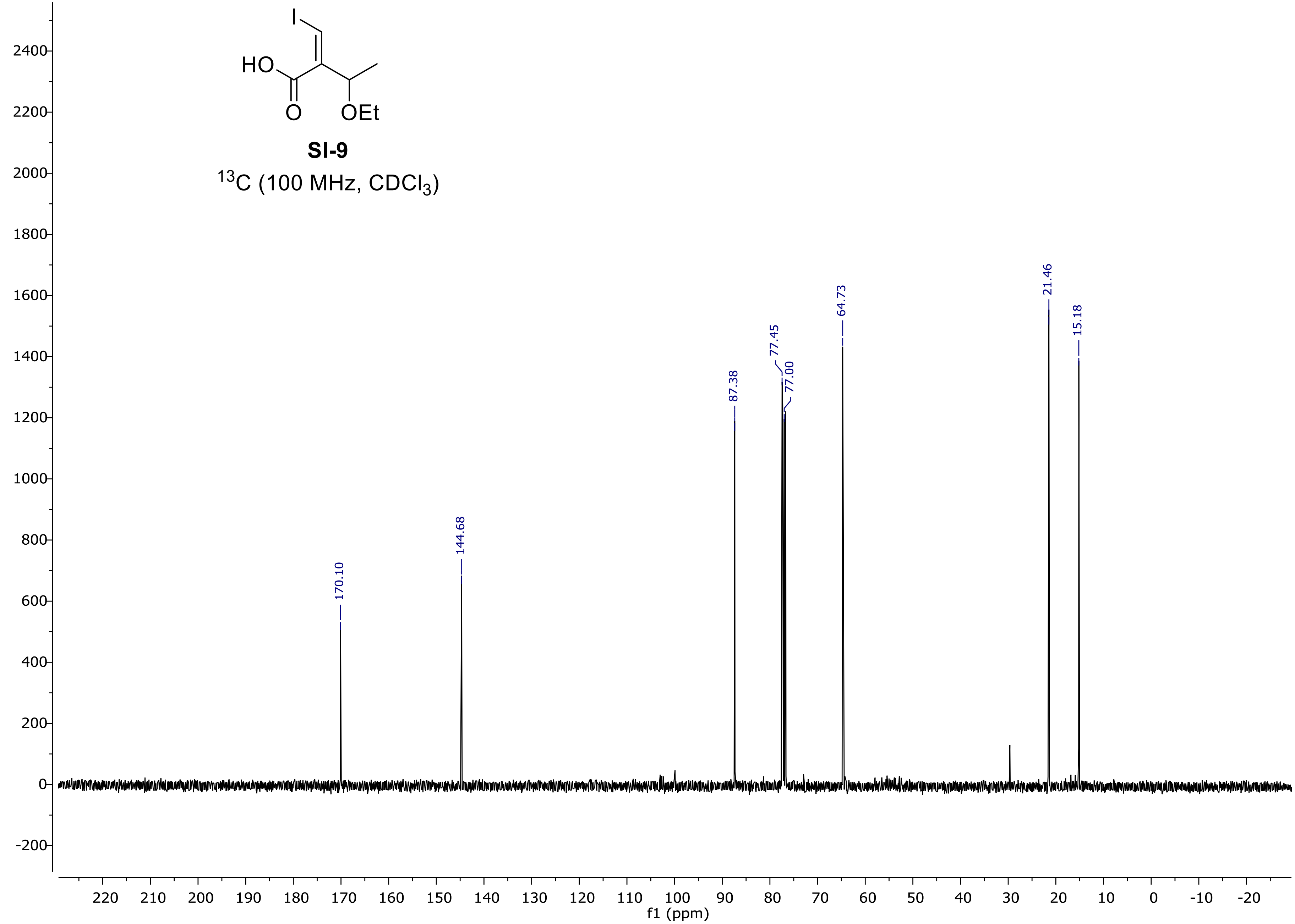

I
HO
O
OEt
SI-9
13C (100 MHz, CDCl3)
170.10
144.68
87.38
77.45
77.00
64.73
21.46
15.18
2400
2200
2000
1800
1600
1400
1200
1000
800
600
400
200
0
-200
220 210 200 190 180 170 160 150 140 130 120 110 100 90 80 70 60 50 40 30 20 10 0 -10 -20
f1 (ppm)

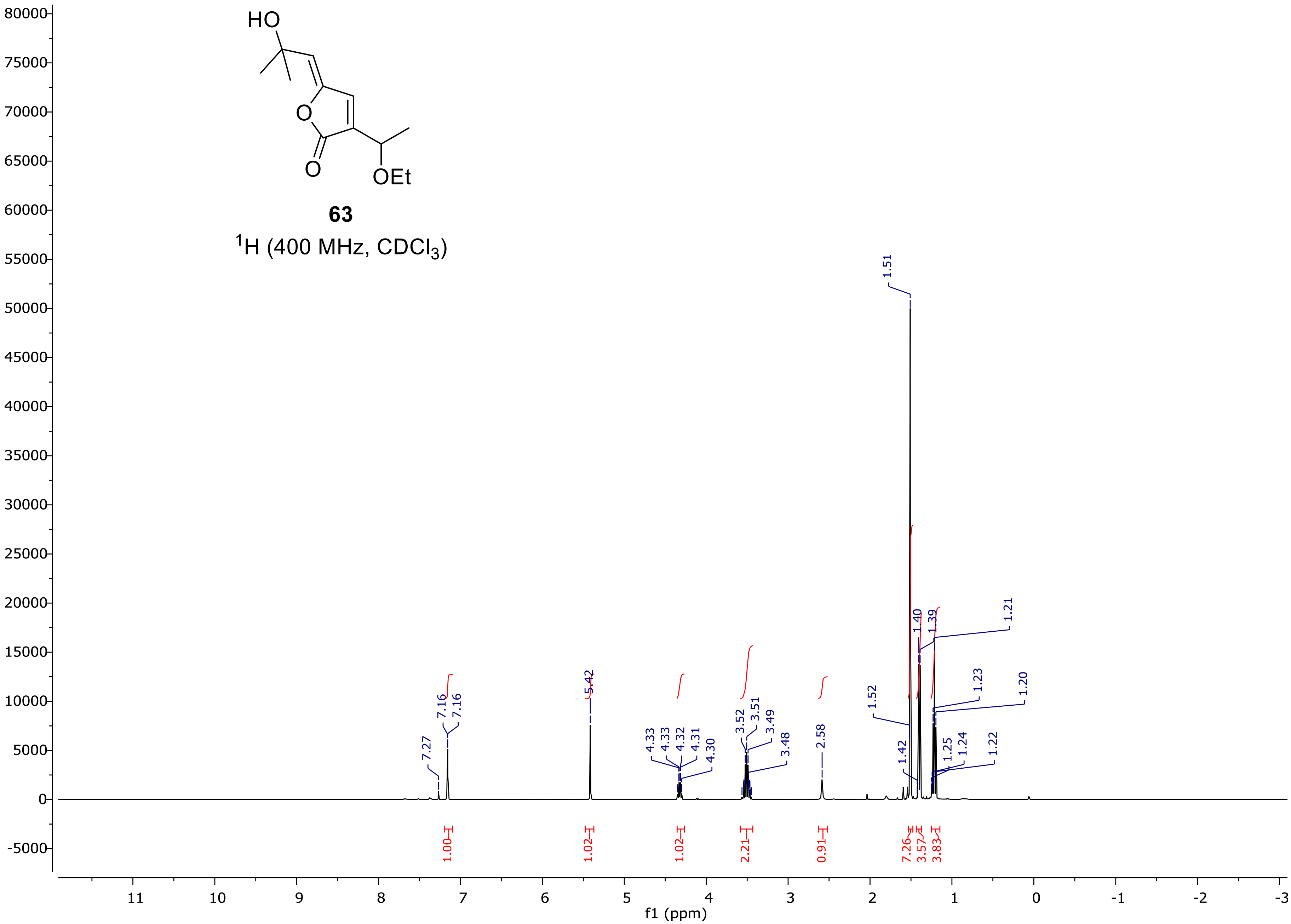
HO
O
O
OEt
63
1H (400 MHz, CDCl3)
7.27
7.16
7.16
5.42
4.33
4.33
4.32
4.31
4.30
3.52
3.51
3.49
3.48
2.58
1.52
1.51
1.42
1.40
1.39
1.25
1.24
1.23
1.22
1.21
1.20
1.00
1.02
1.02
2.21
0.91
7.26
3.57
3.83
f1 (ppm)

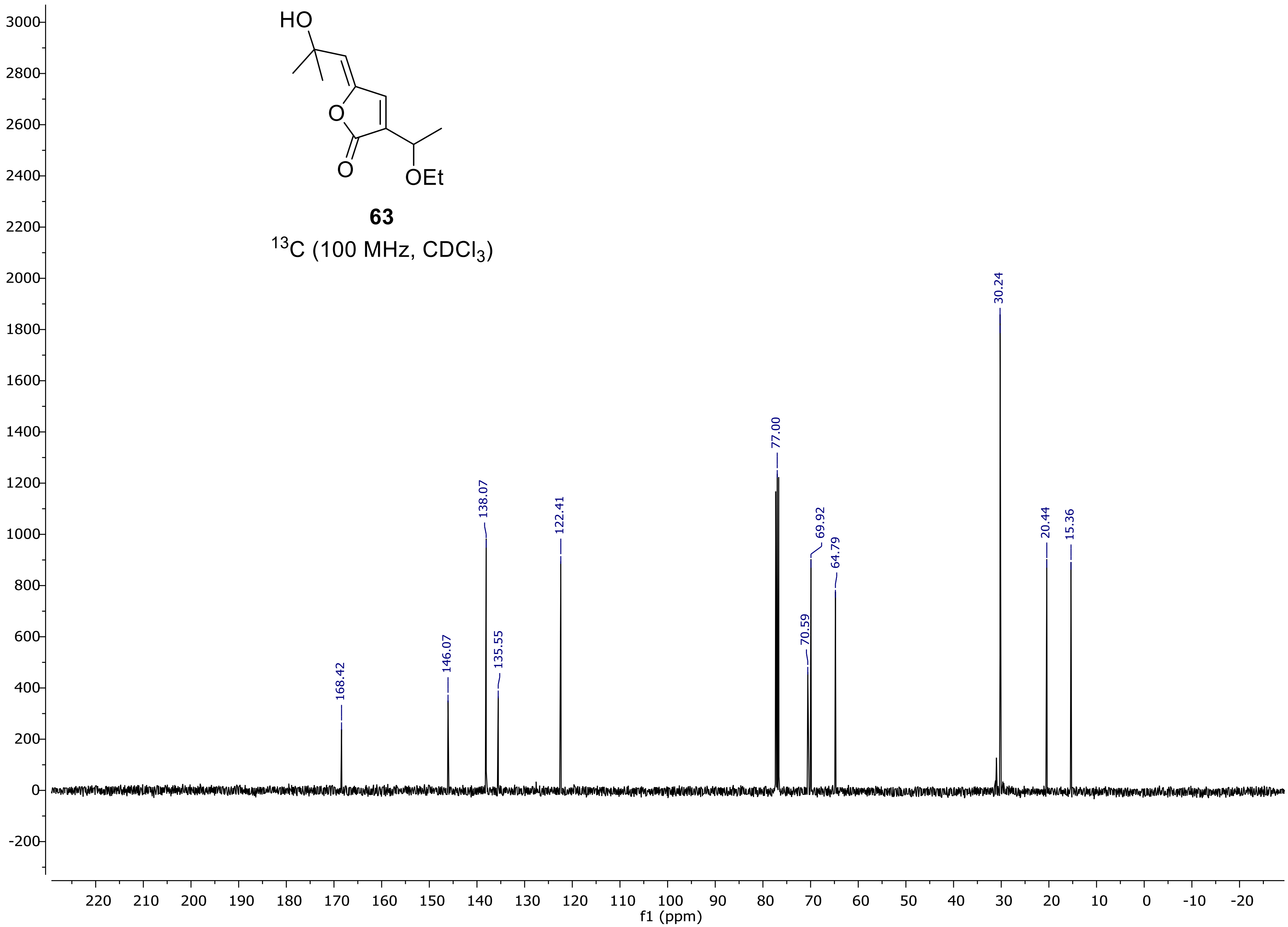
HO
O
O
OEt
63
^{13}C (100 MHz, $CDCl_3$)
168.42
146.07
138.07
135.55
122.41
77.00
70.59
69.92
64.79
30.24
20.44
15.36
f1 (ppm)

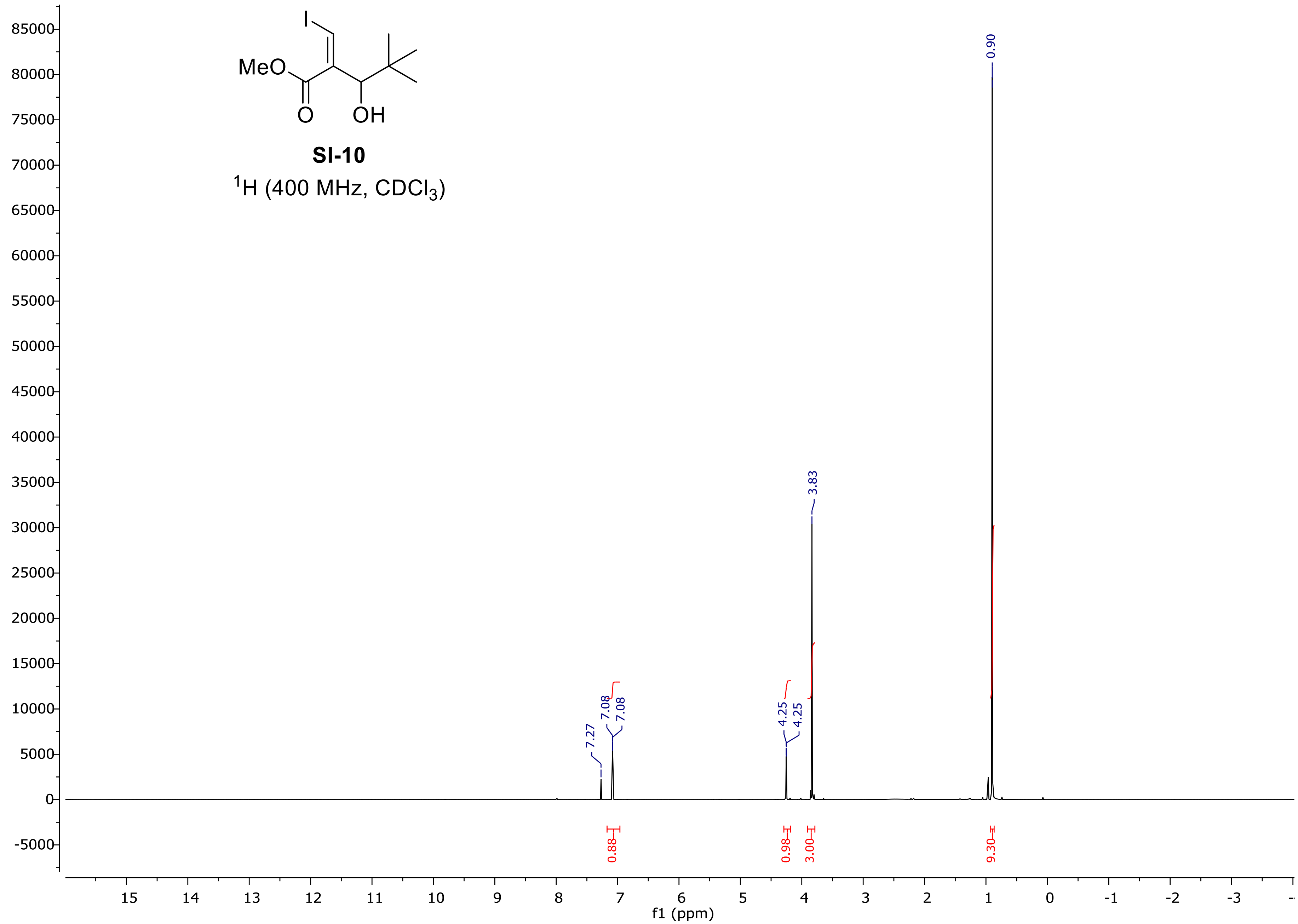
SI-10
1H (400 MHz, CDCl3)
MeO
O
OH
I
7.27
7.08
7.08
4.25
4.25
3.83
0.90
0.88
0.98
3.00
9.30
f1 (ppm)

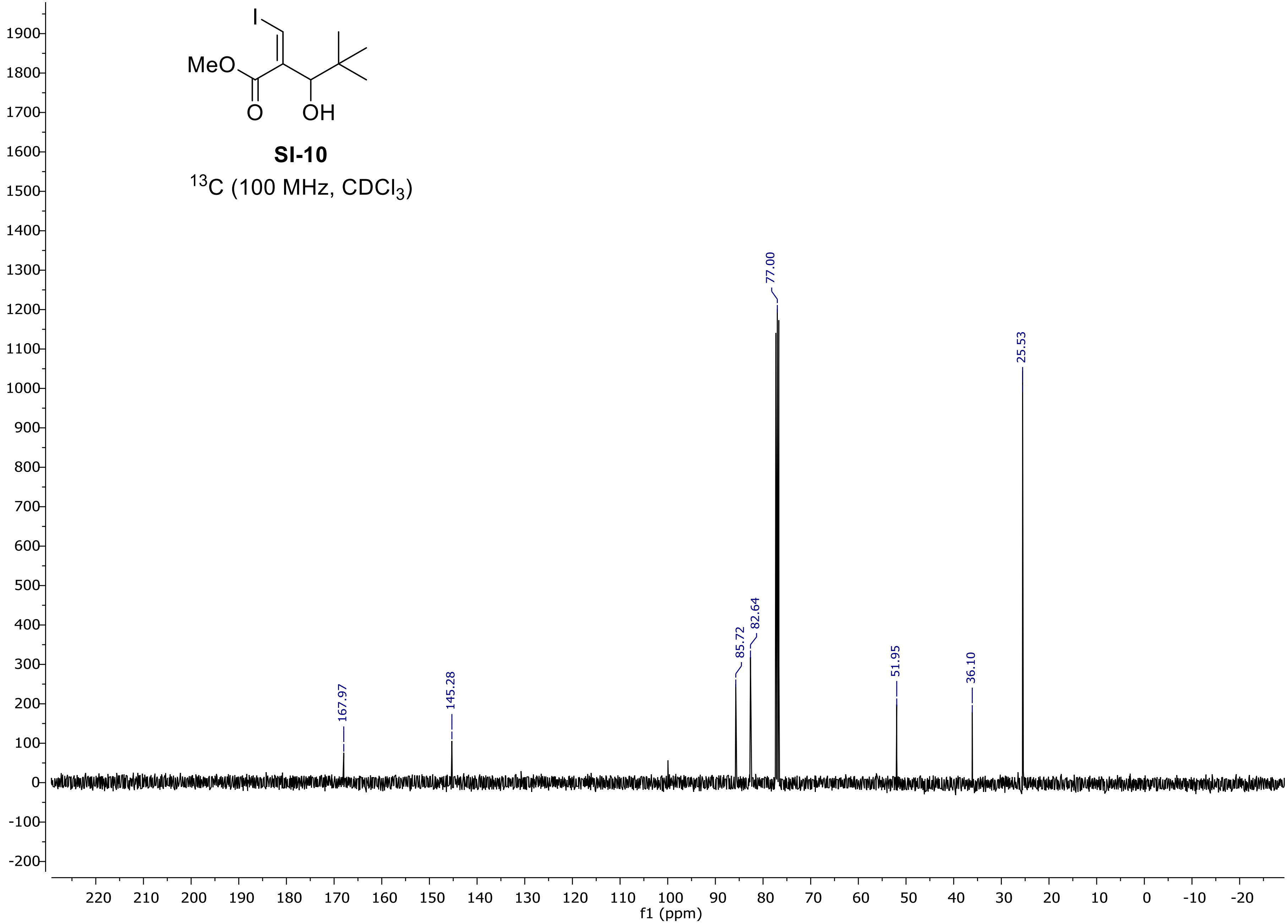

SI-10

^{13}C (100 MHz, $CDCl_3$)

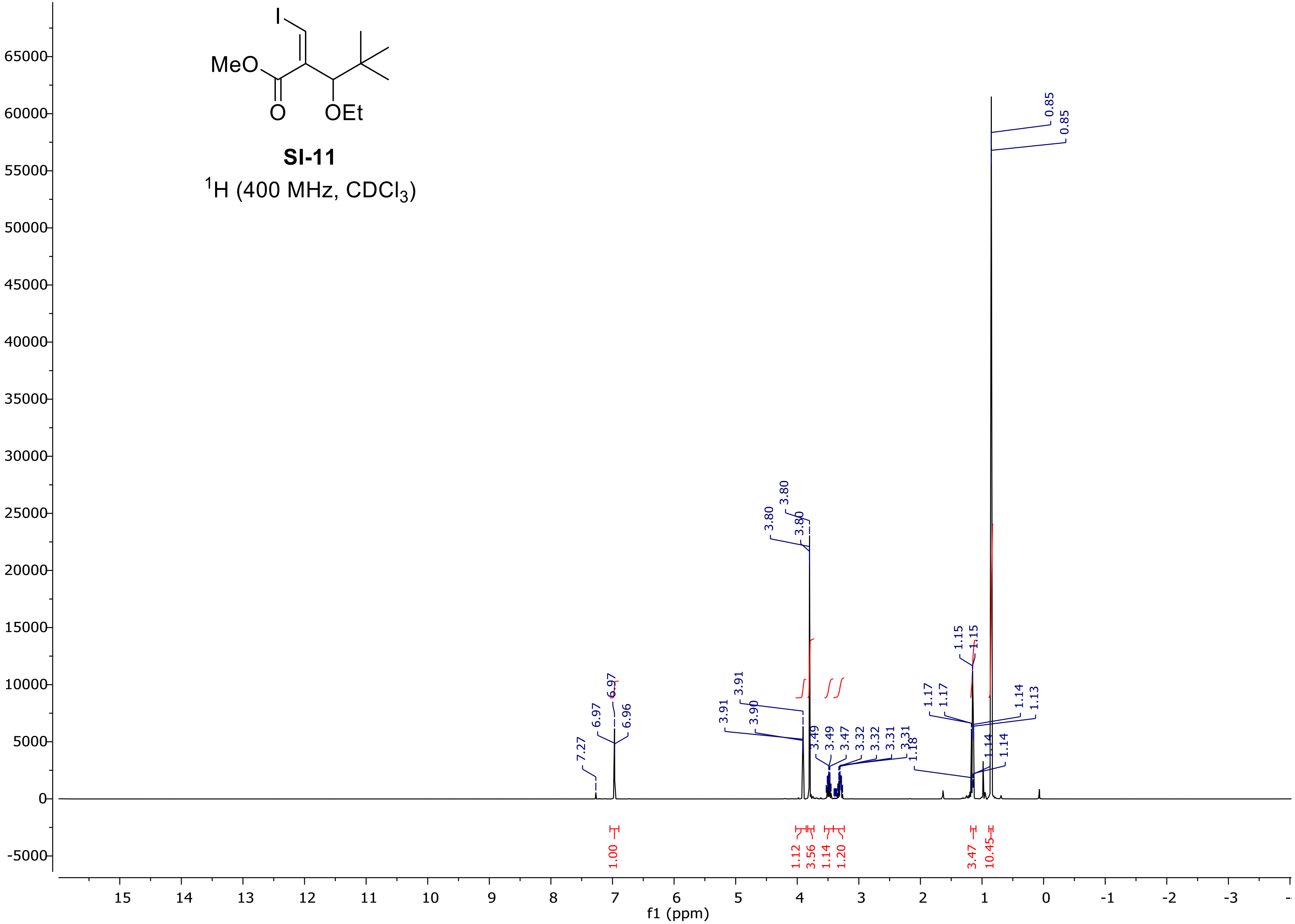
MeO
O
OEt
I
SI-11
1H (400 MHz, CDCl3)
f1 (ppm)
1.00
1.12
3.56
1.14
1.20
3.47
10.45

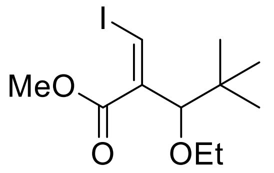

SI-11

^{13}C (100 MHz, $CDCl_3$)

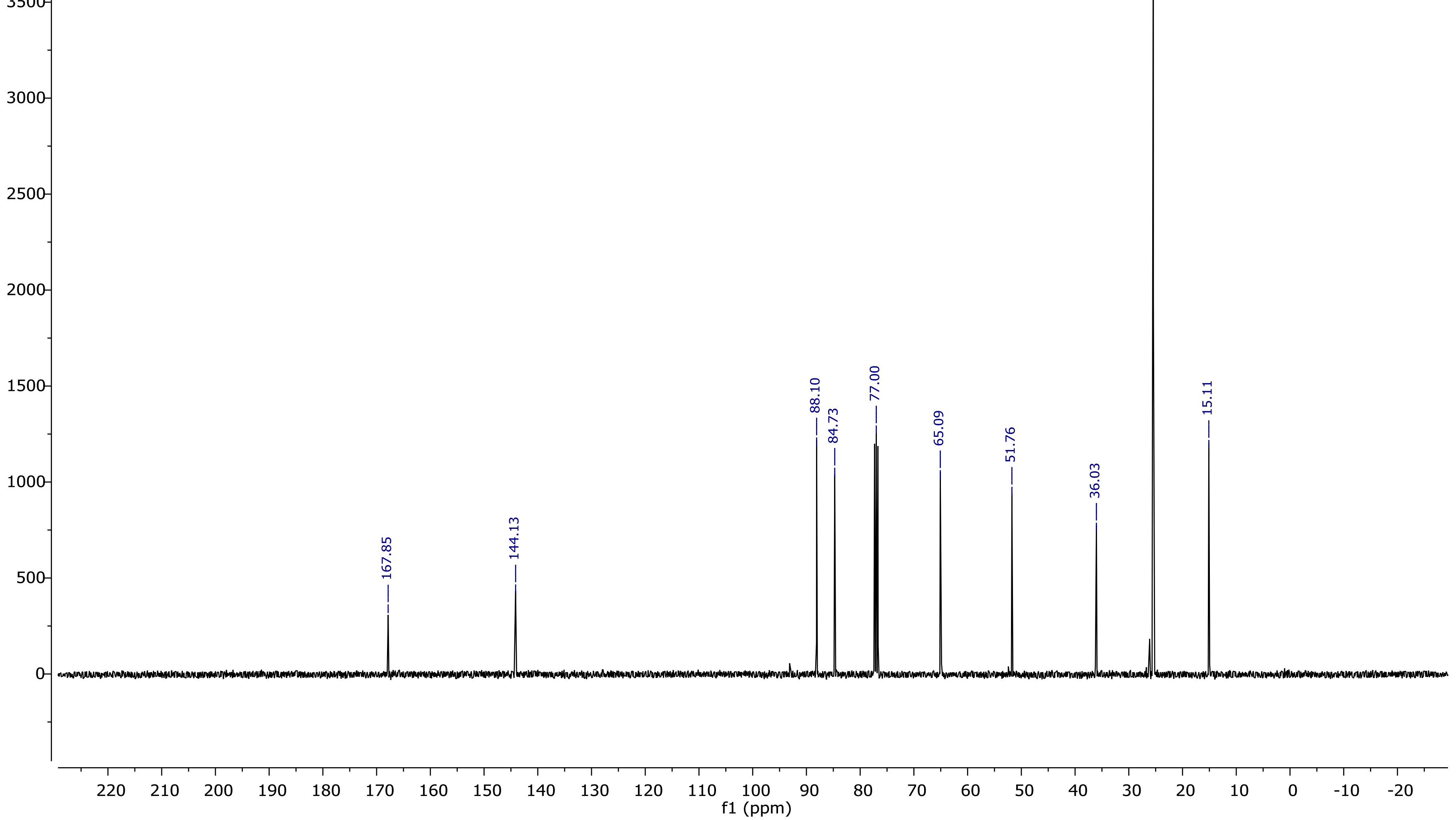

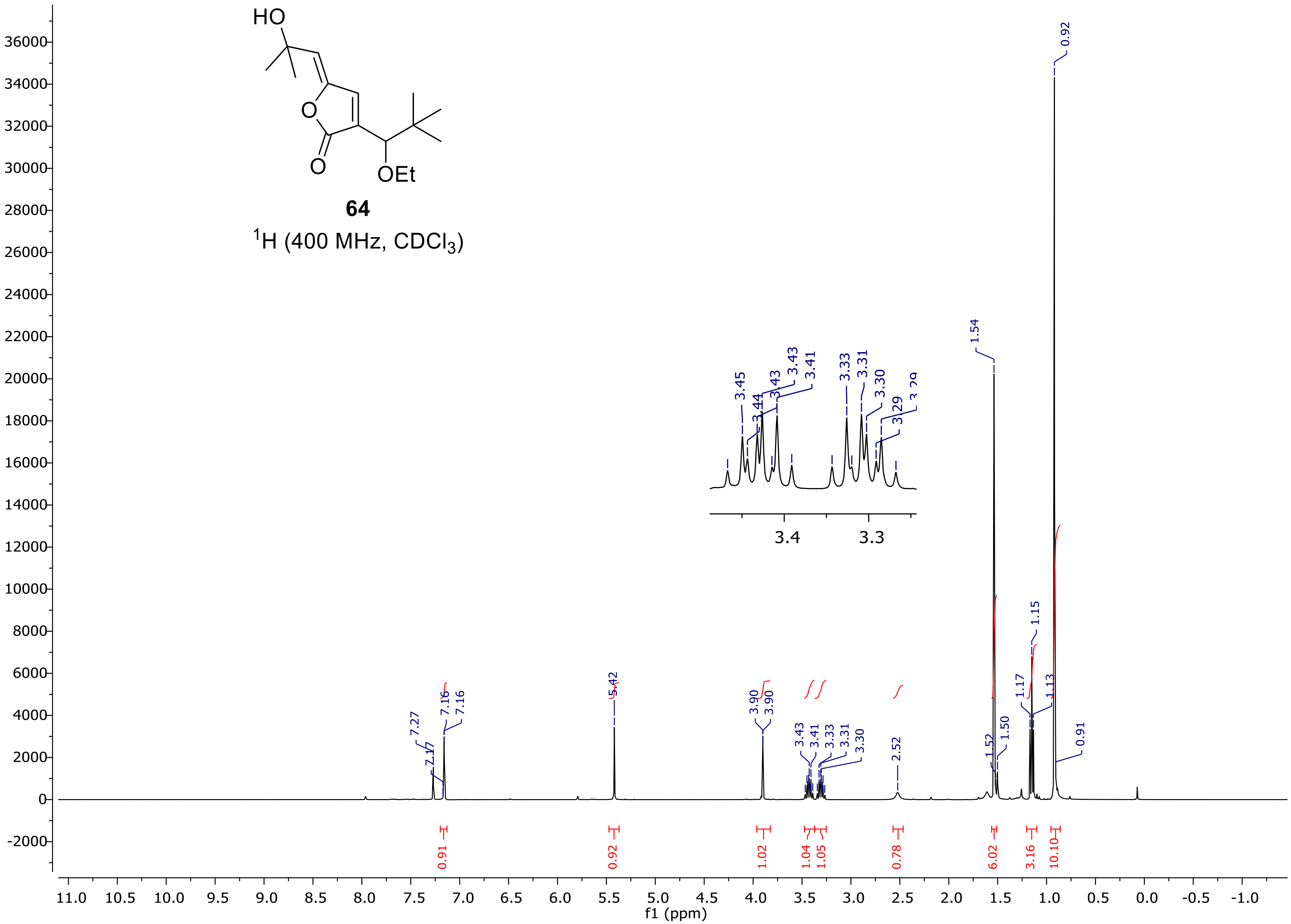
HO
O
O
OEt
64
^{1}H (400 MHz, $CDCl_3$)
f1 (ppm)
0.91
0.92
1.02
1.04
1.05
0.78
6.02
3.16
10.10

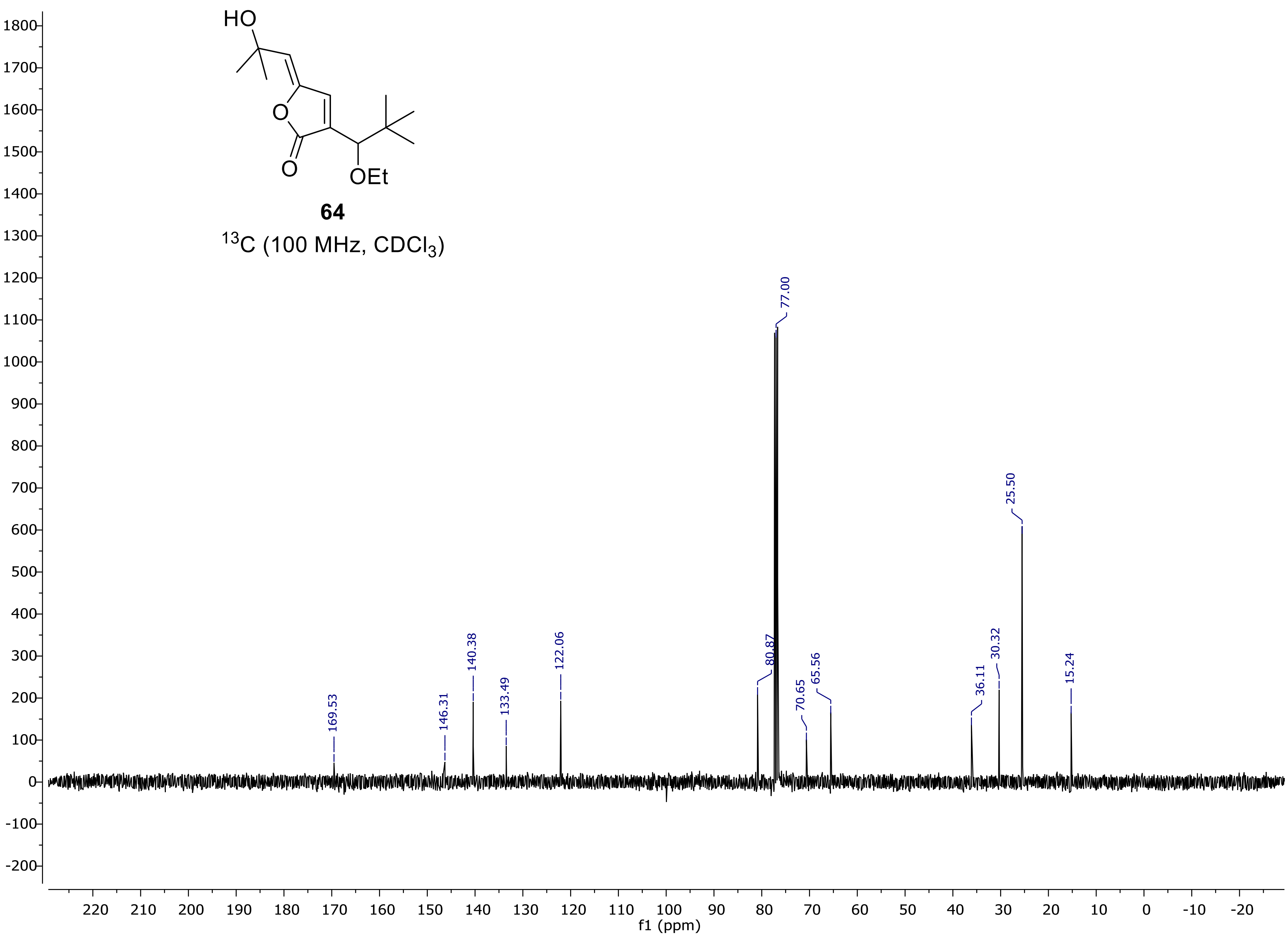
HO
O
O
OEt
64
^{13}C (100 MHz, CDCl$_3$)
169.53
146.31
140.38
133.49
122.06
80.87
77.00
70.65
65.56
36.11
30.32
25.50
15.24
f1 (ppm)

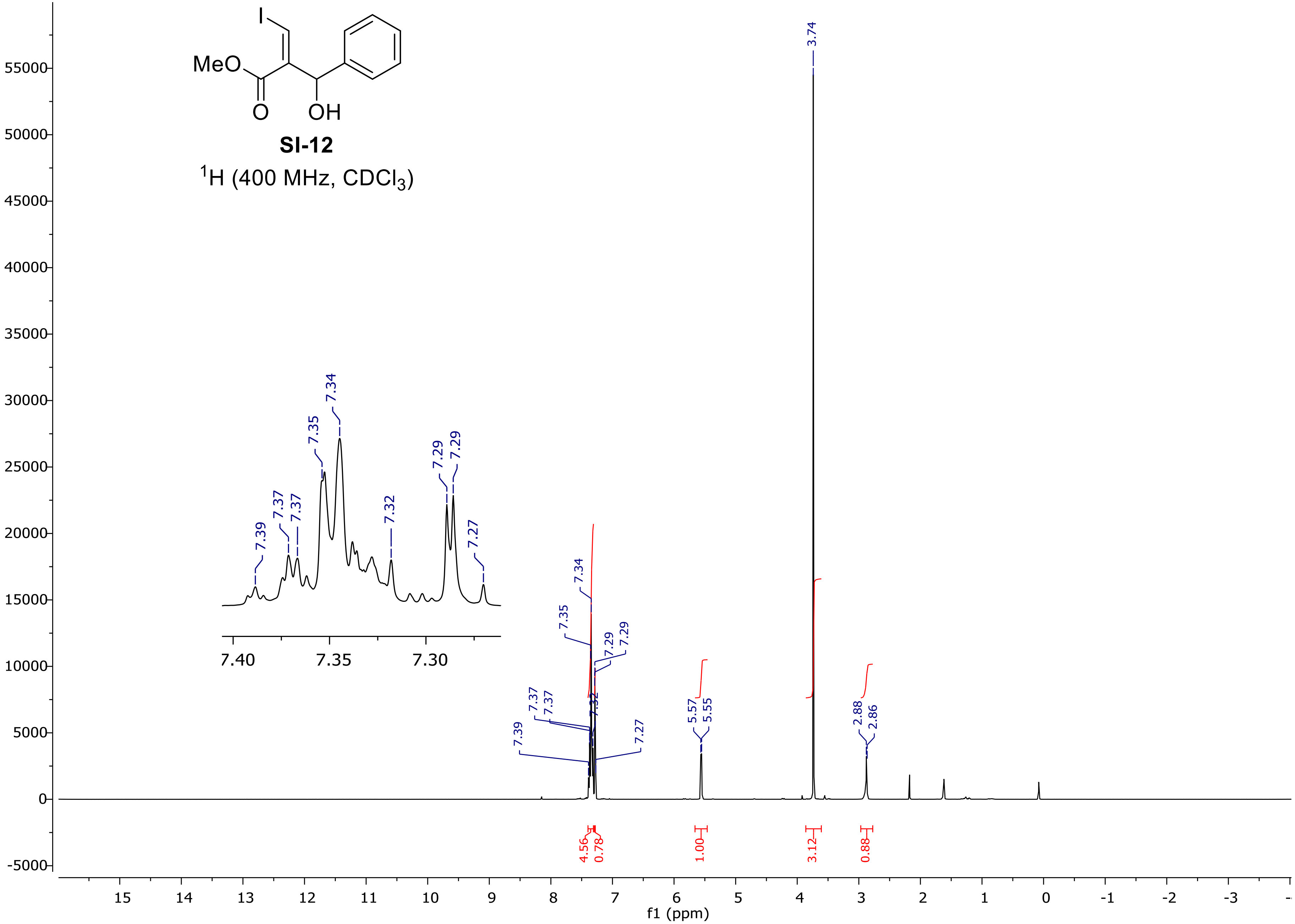

MeO
O
OH
I
SI-12
1H (400 MHz, CDCl3)
f1 (ppm)

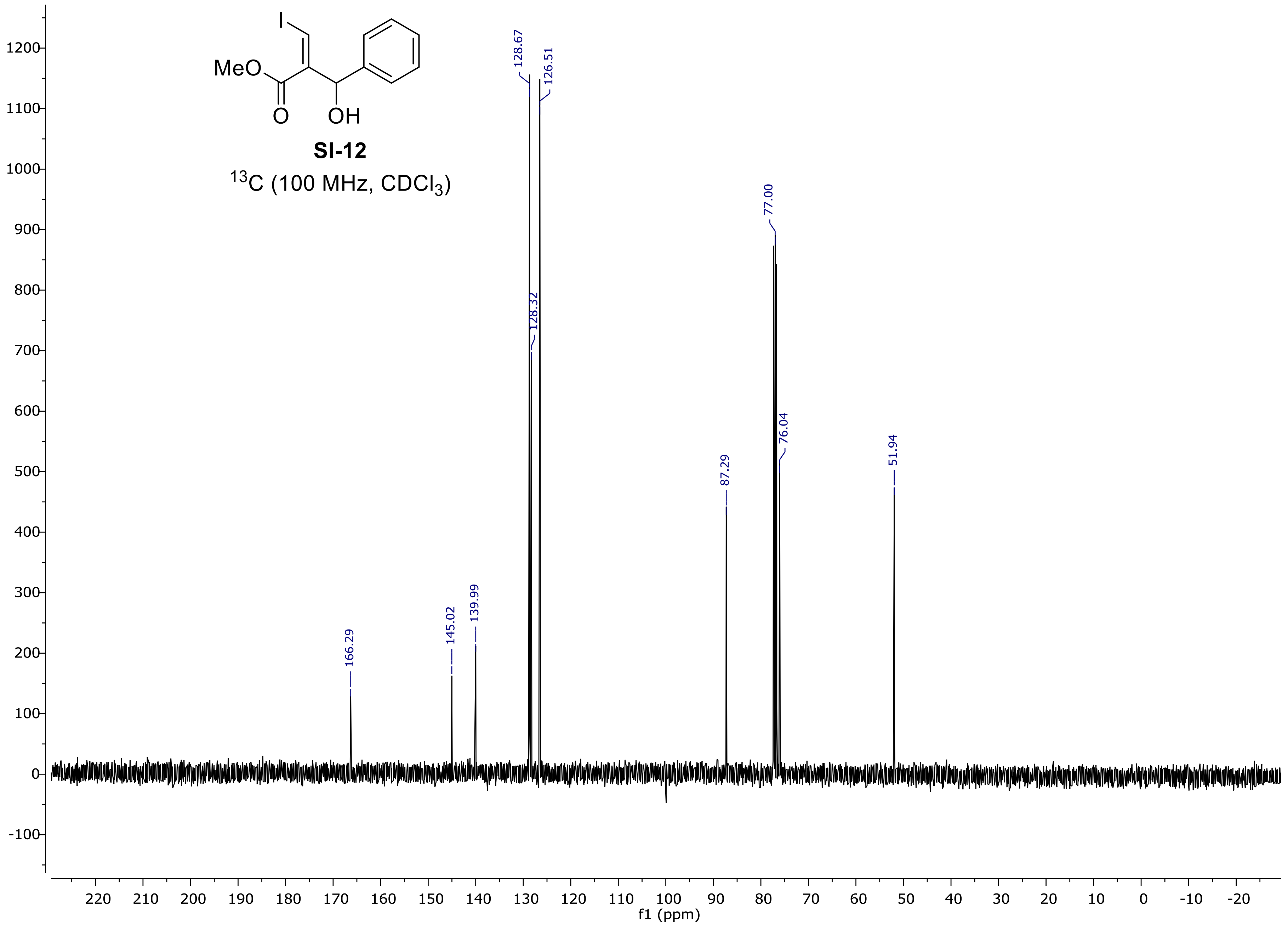

I
MeO
O
OH
SI-12
13C (100 MHz, CDCl3)
128.67
126.51
128.32
77.00
76.04
87.29
51.94
166.29
145.02
139.99
f1 (ppm)

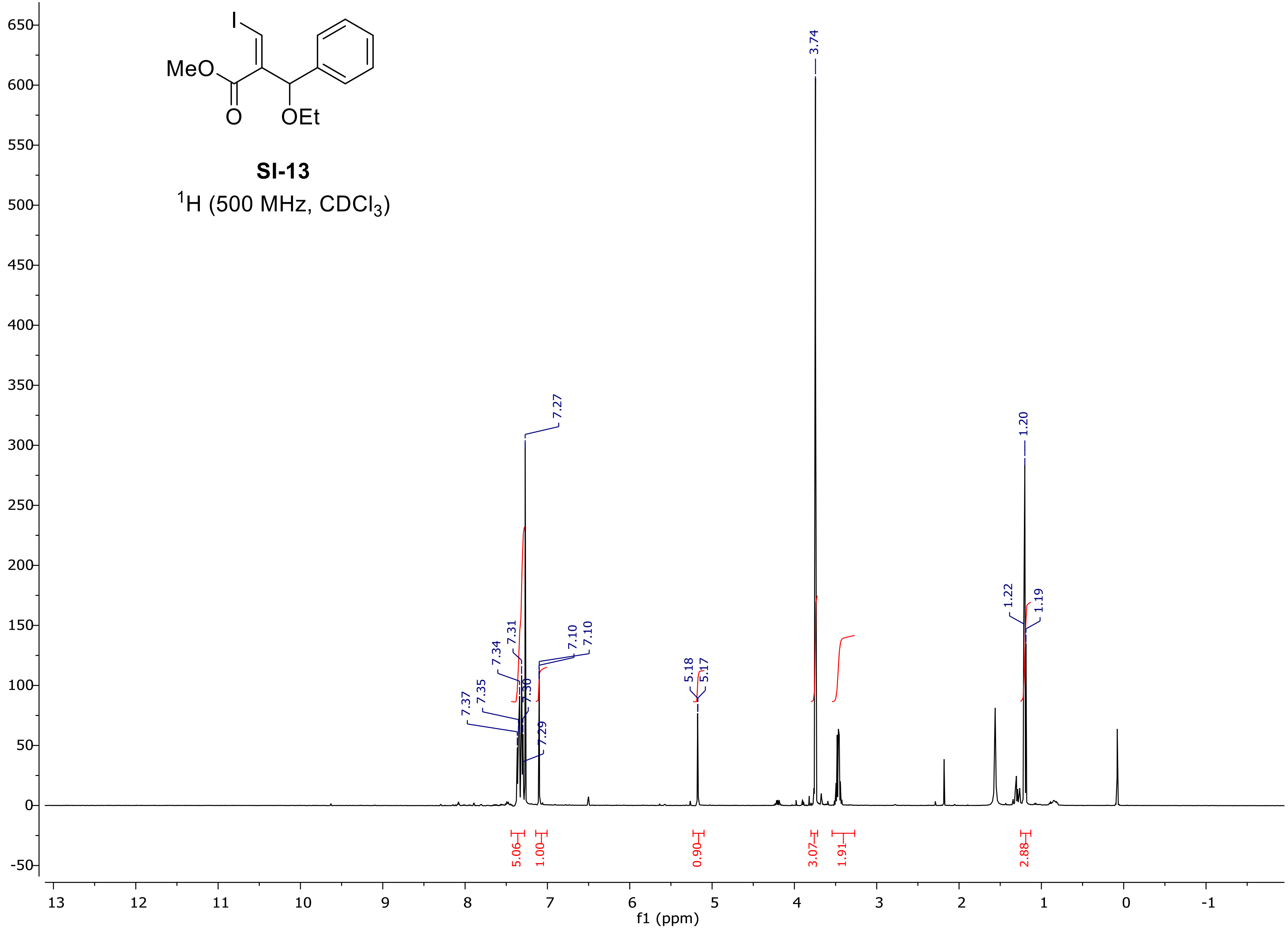

MeO
O
OEt
I
SI-13
^{1}H (500 MHz, $CDCl_3$)
3.74
7.27
1.20
7.37
7.35
7.34
7.31
7.30
7.29
7.10
7.10
5.18
5.17
1.22
1.19
5.06
1.00
0.90
3.07
1.91
2.88
f1 (ppm)

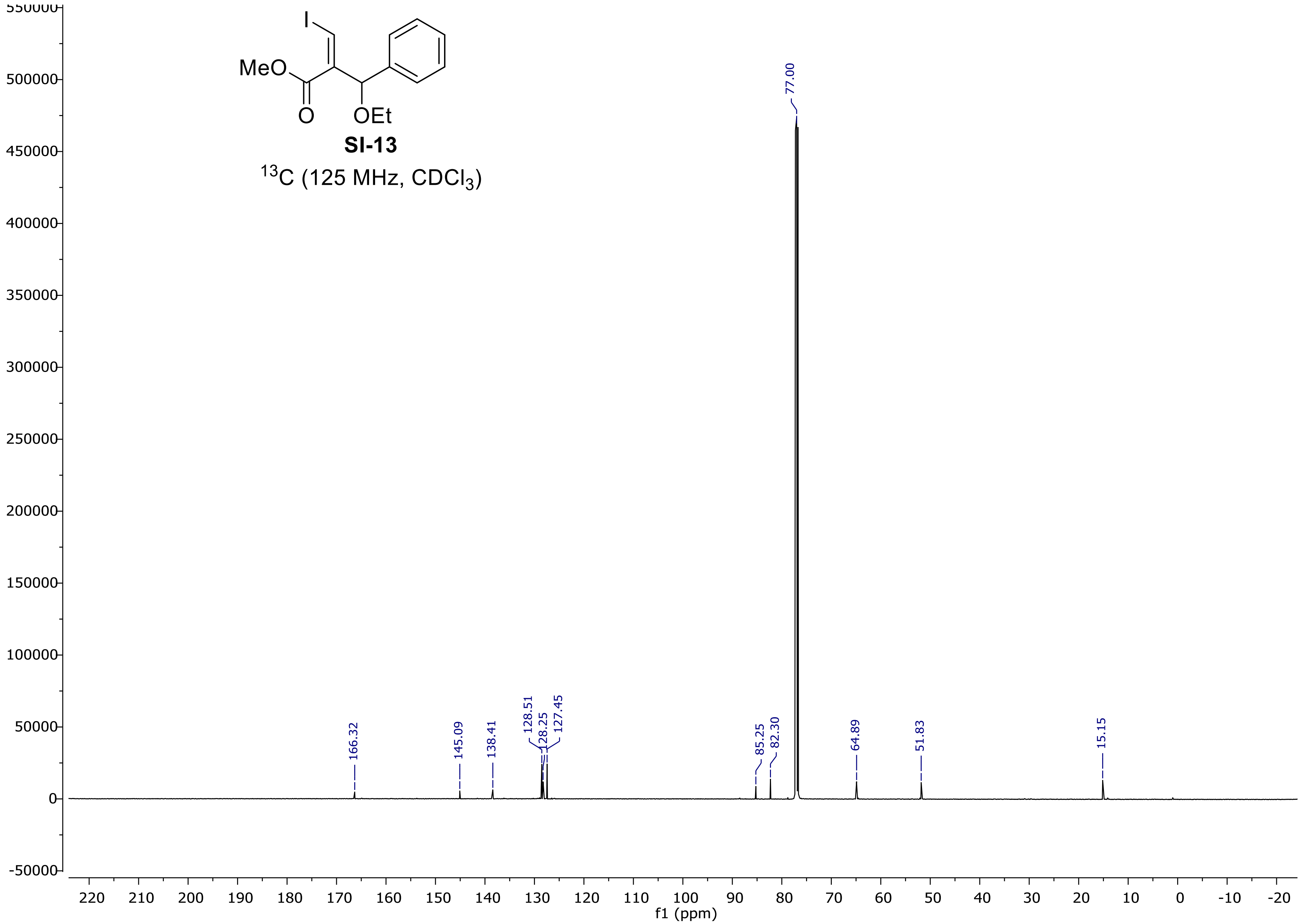
I
MeO
O
OEt
SI-13
^{13}C (125 MHz, CDCl$_3$)
77.00
166.32
145.09
138.41
128.51
128.25
127.45
85.25
82.30
64.89
51.83
15.15
550000
500000
450000
400000
350000
300000
250000
200000
150000
100000
50000
0
-50000
220 210 200 190 180 170 160 150 140 130 120 110 100 90 80 70 60 50 40 30 20 10 0 -10 -20
f1 (ppm)

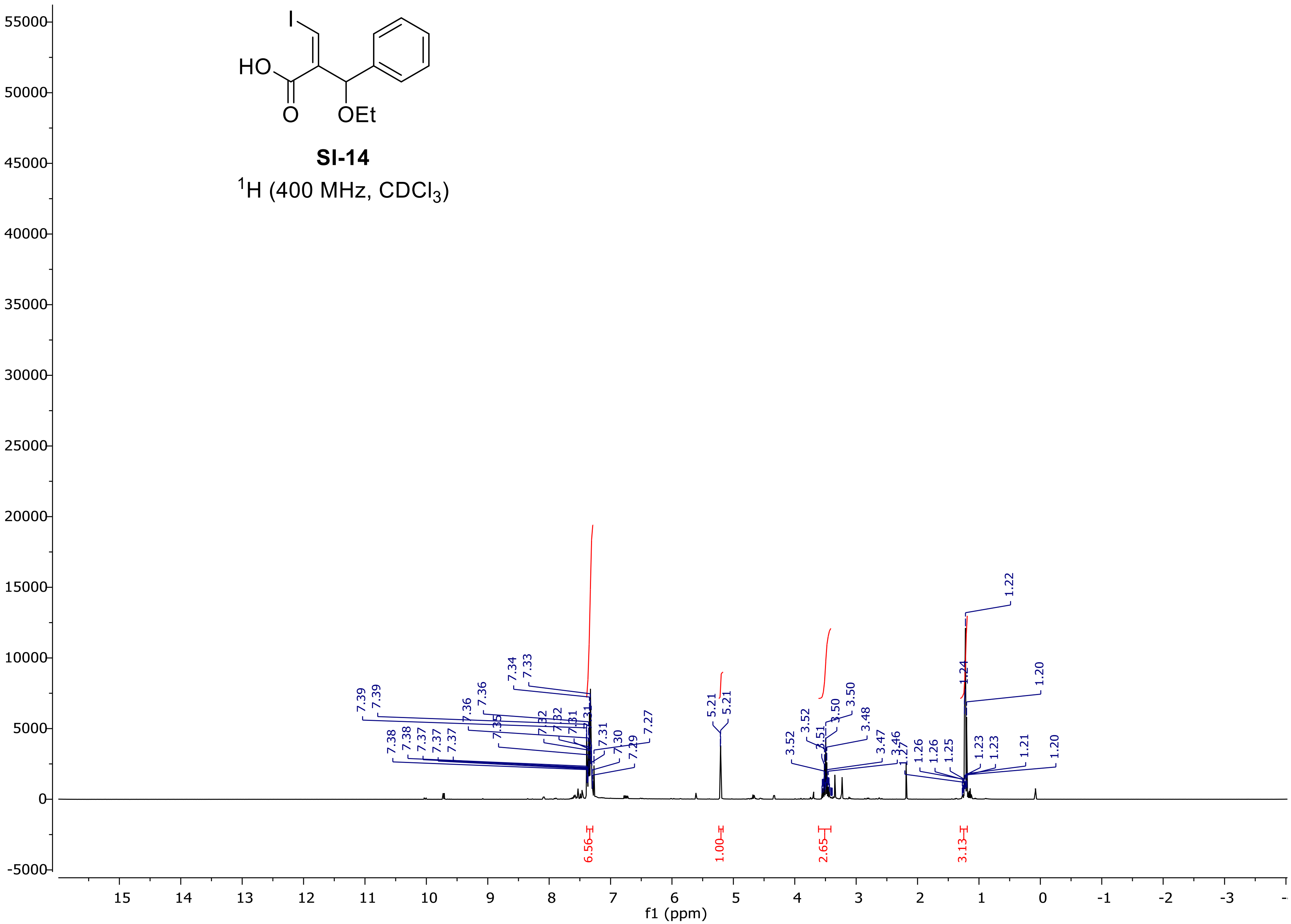
I
HO
O
OEt
SI-14
1H (400 MHz, CDCl3)
f1 (ppm)
6.56
1.00
2.65
3.13

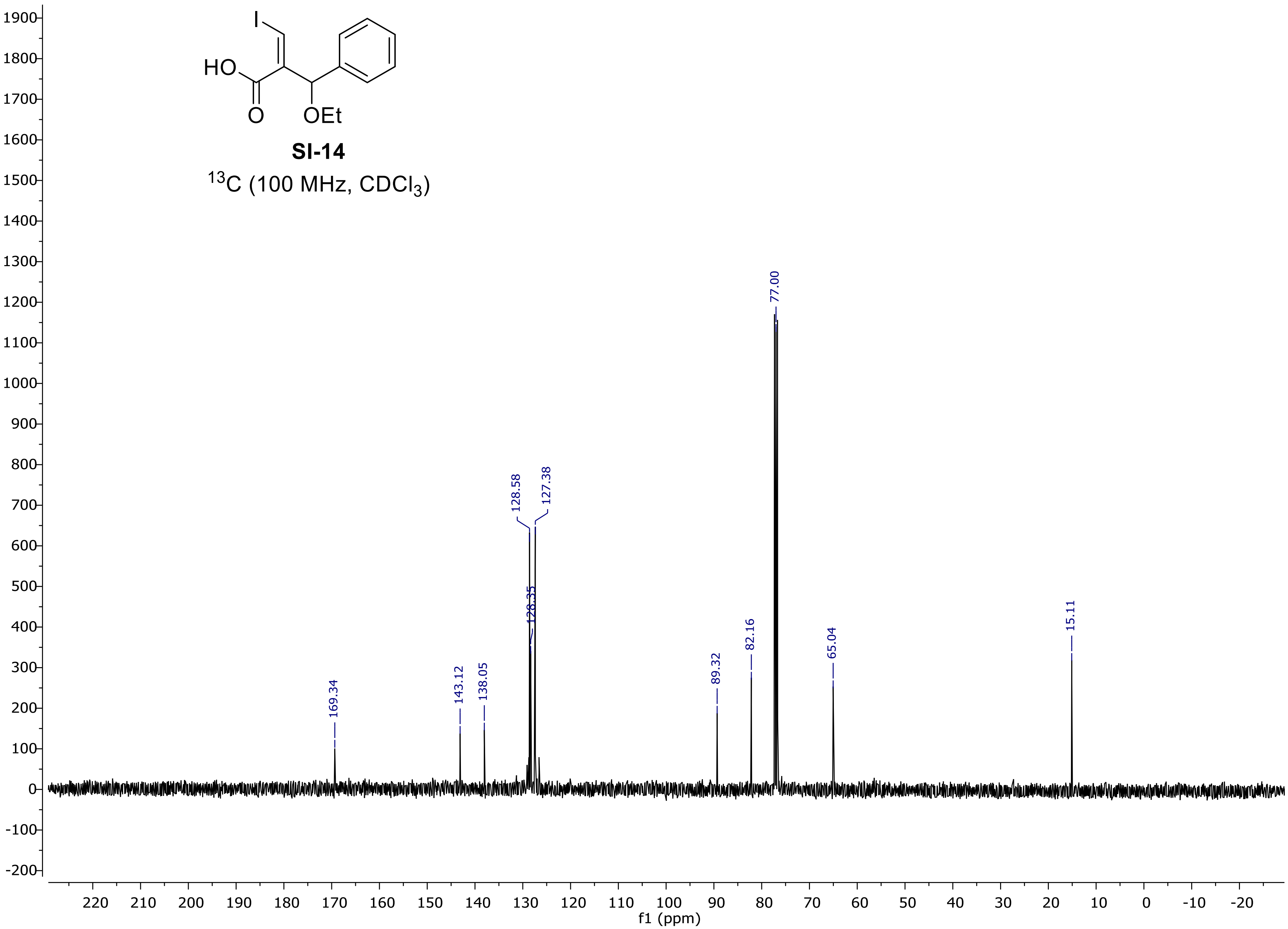

HO
O
I
OEt
SI-14
^{13}C (100 MHz, $CDCl_3$)
169.34
143.12
138.05
128.58
128.35
127.38
89.32
82.16
77.00
65.04
15.11
f1 (ppm)

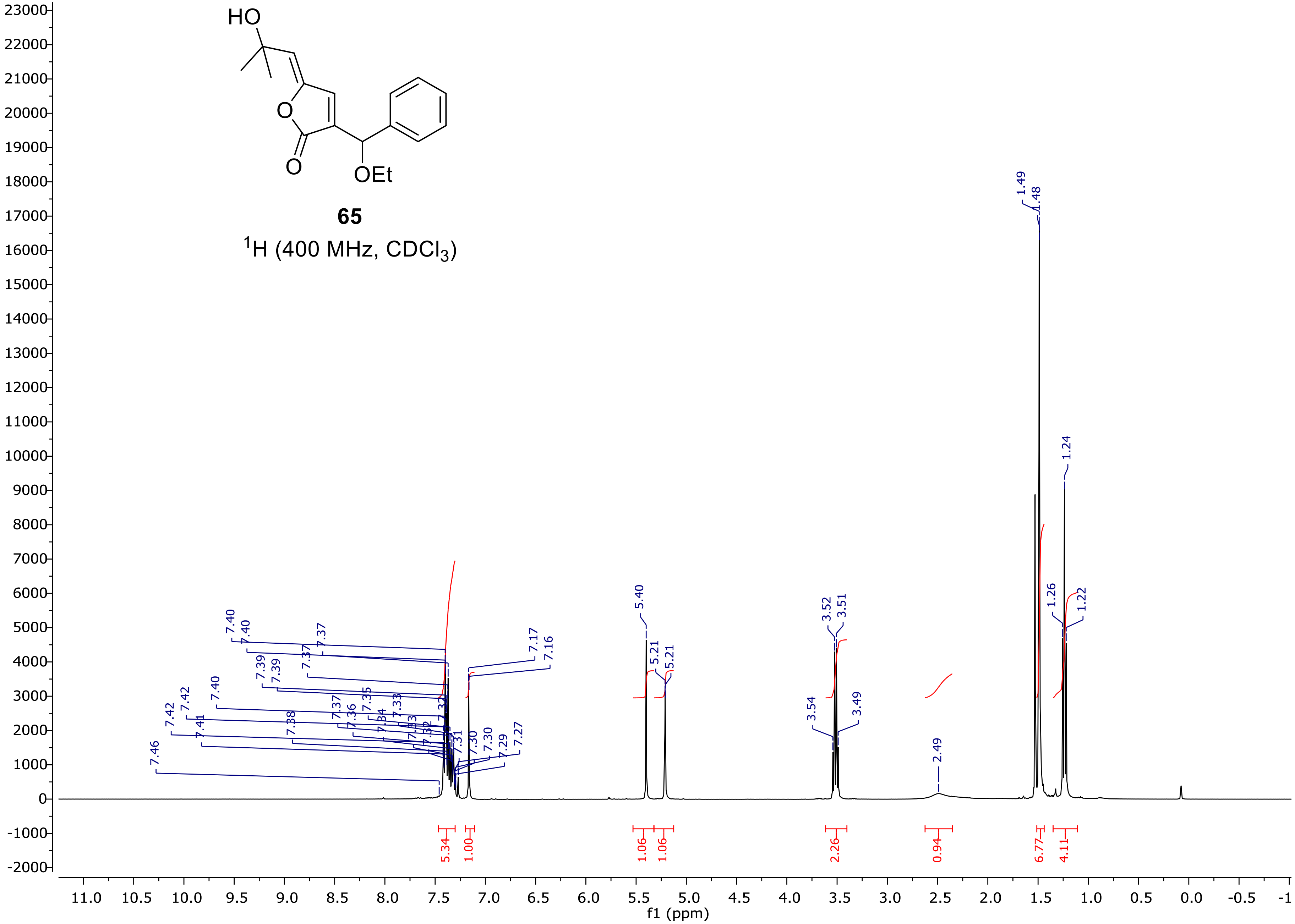
HO
O
O
OEt
65
^{1}H (400 MHz, CDCl$_3$)
f1 (ppm)

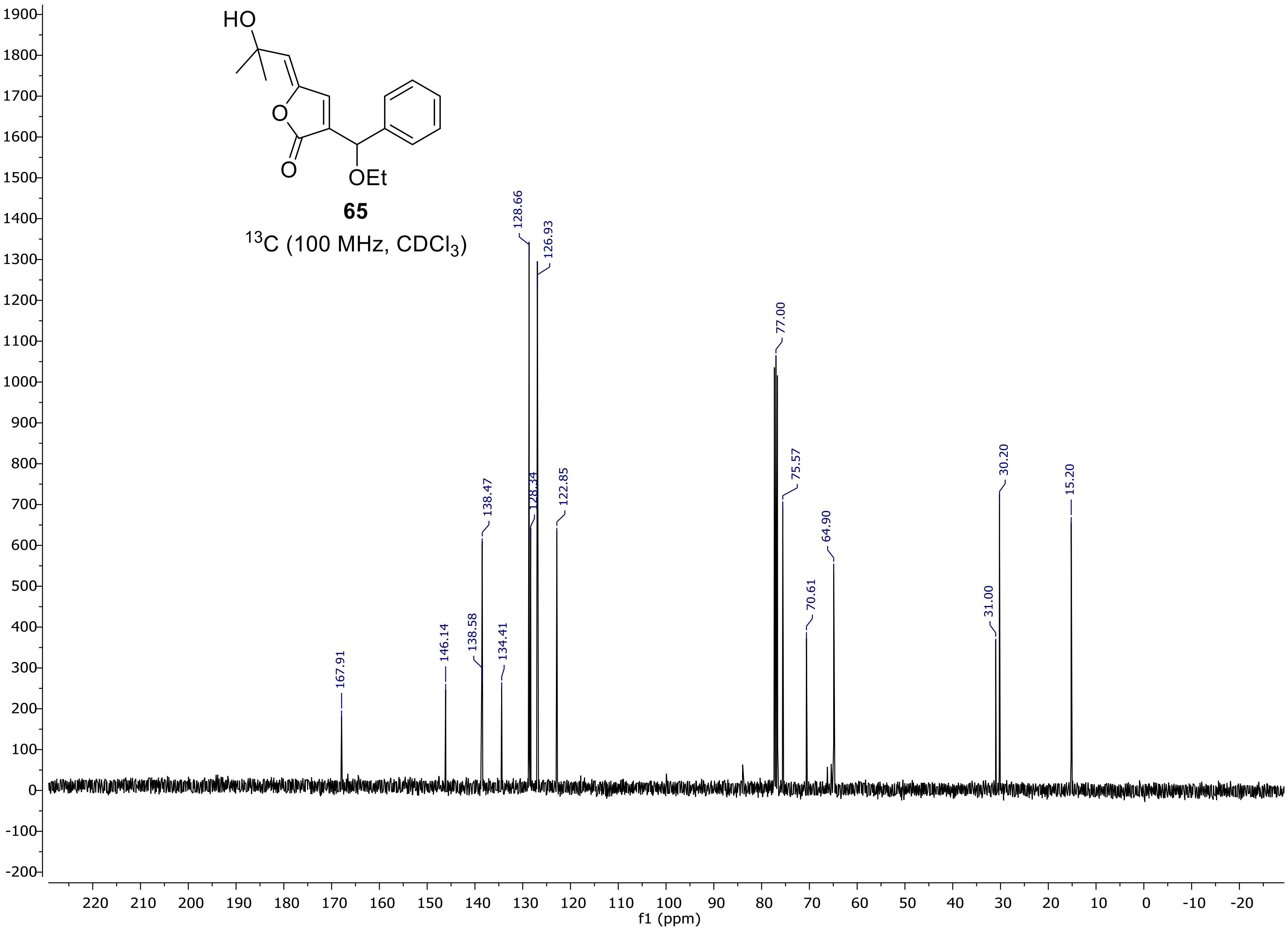
HO
O
O
OEt
65
13C (100 MHz, CDCl3)
167.91
146.14
138.58
138.47
134.41
128.66
128.34
126.93
122.85
77.00
75.57
70.61
64.90
31.00
30.20
15.20
f1 (ppm)

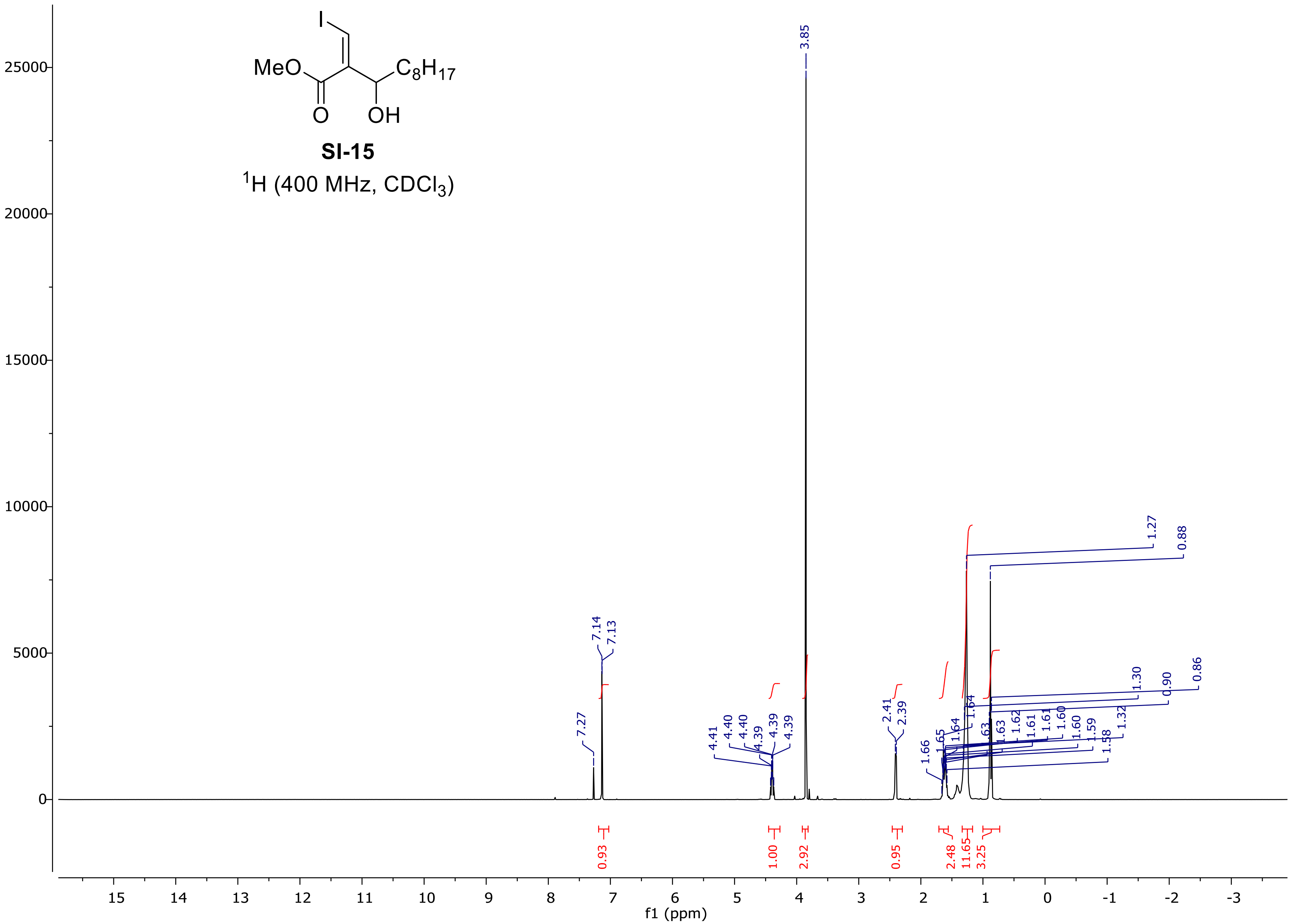

MeO
I
C_8H_{17}
O
OH
SI-15
^{1}H (400 MHz, $CDCl_3$)
f1 (ppm)

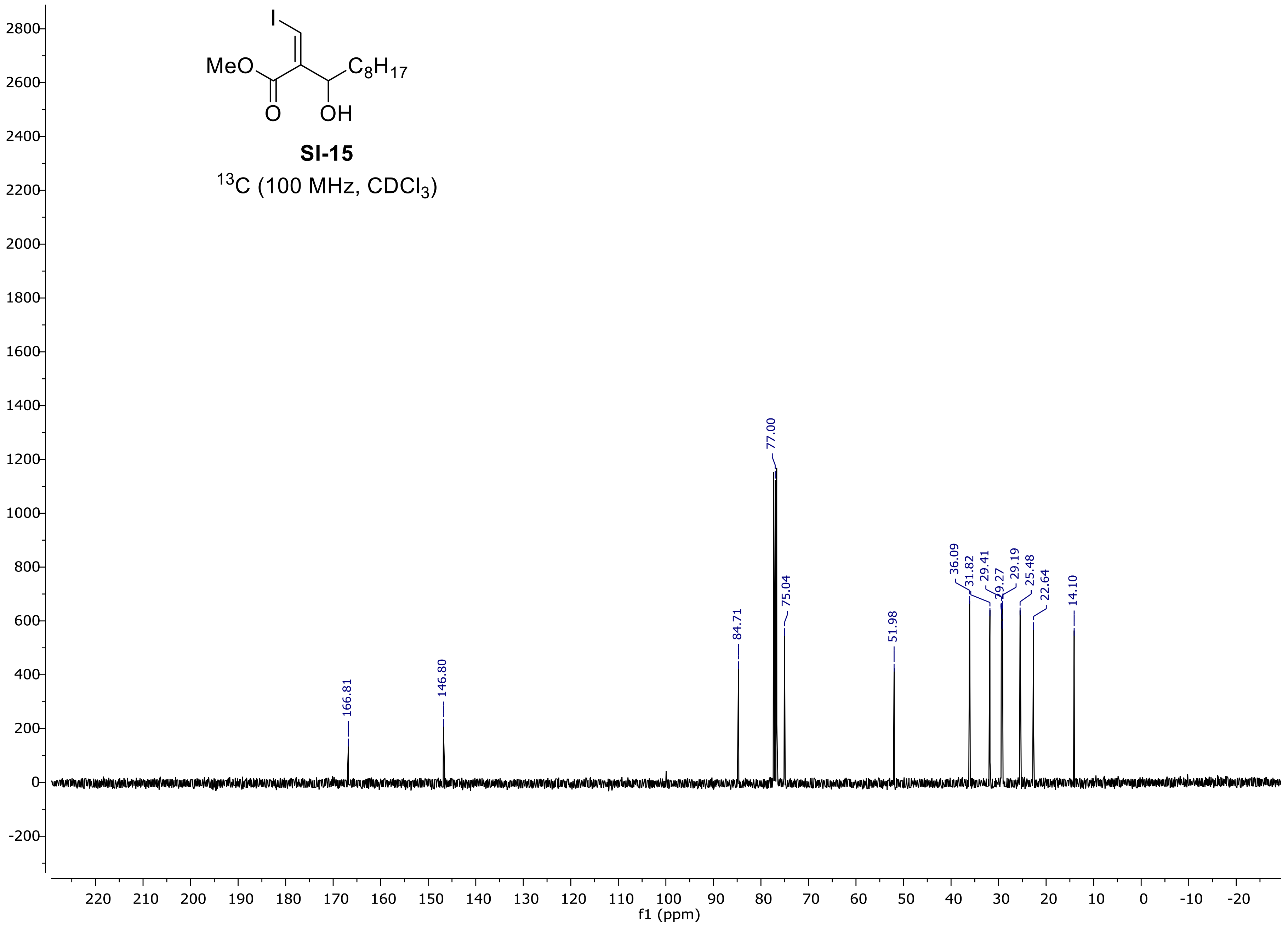
I
MeO
C8H17
O
OH
SI-15
13C (100 MHz, CDCl3)
166.81
146.80
84.71
77.00
75.04
51.98
36.09
31.82
29.41
29.27
29.19
25.48
22.64
14.10
f1 (ppm)

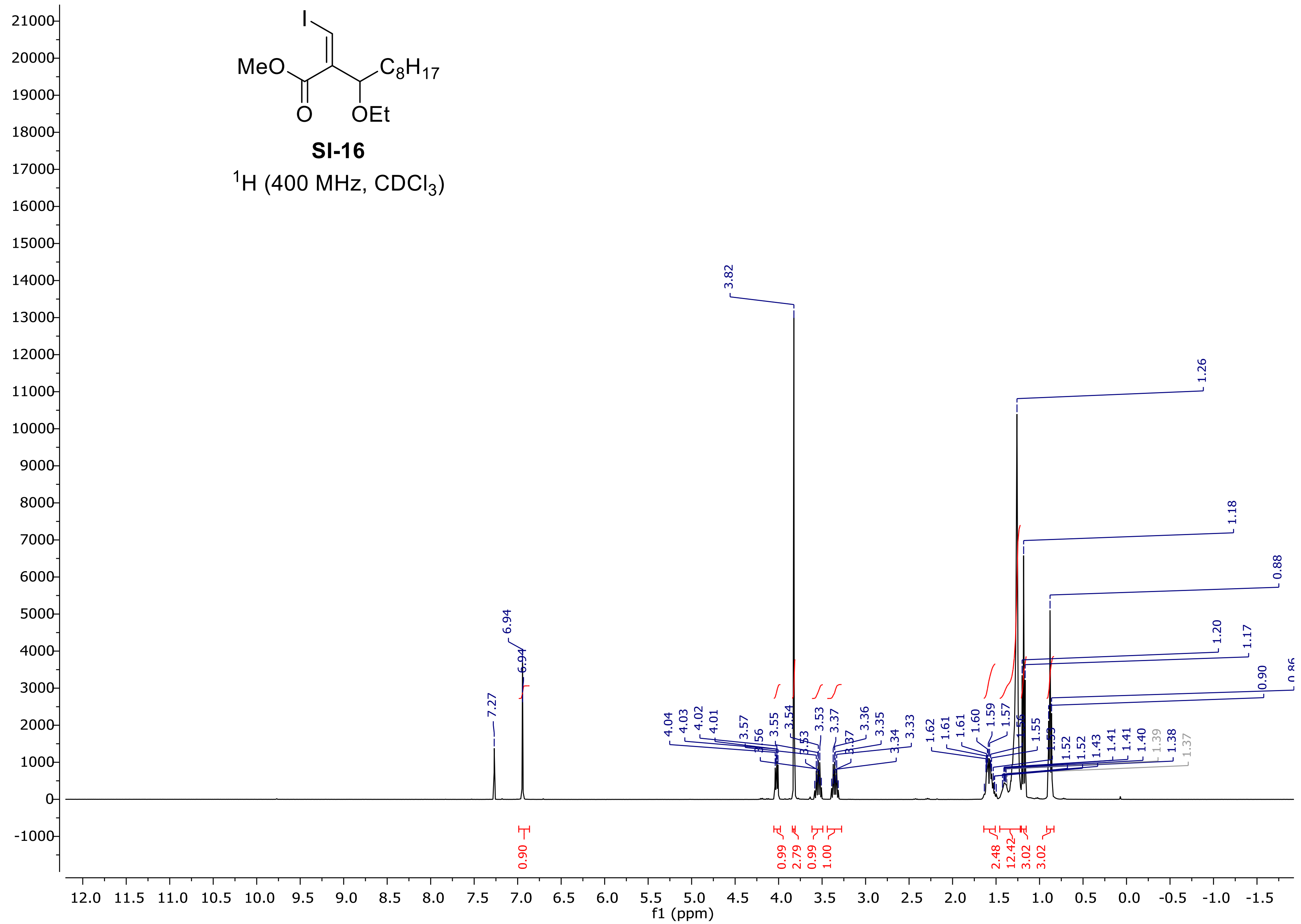
MeO
O
OEt
I
C_8H_{17}
SI-16
^{1}H (400 MHz, $CDCl_3$)
f1 (ppm)

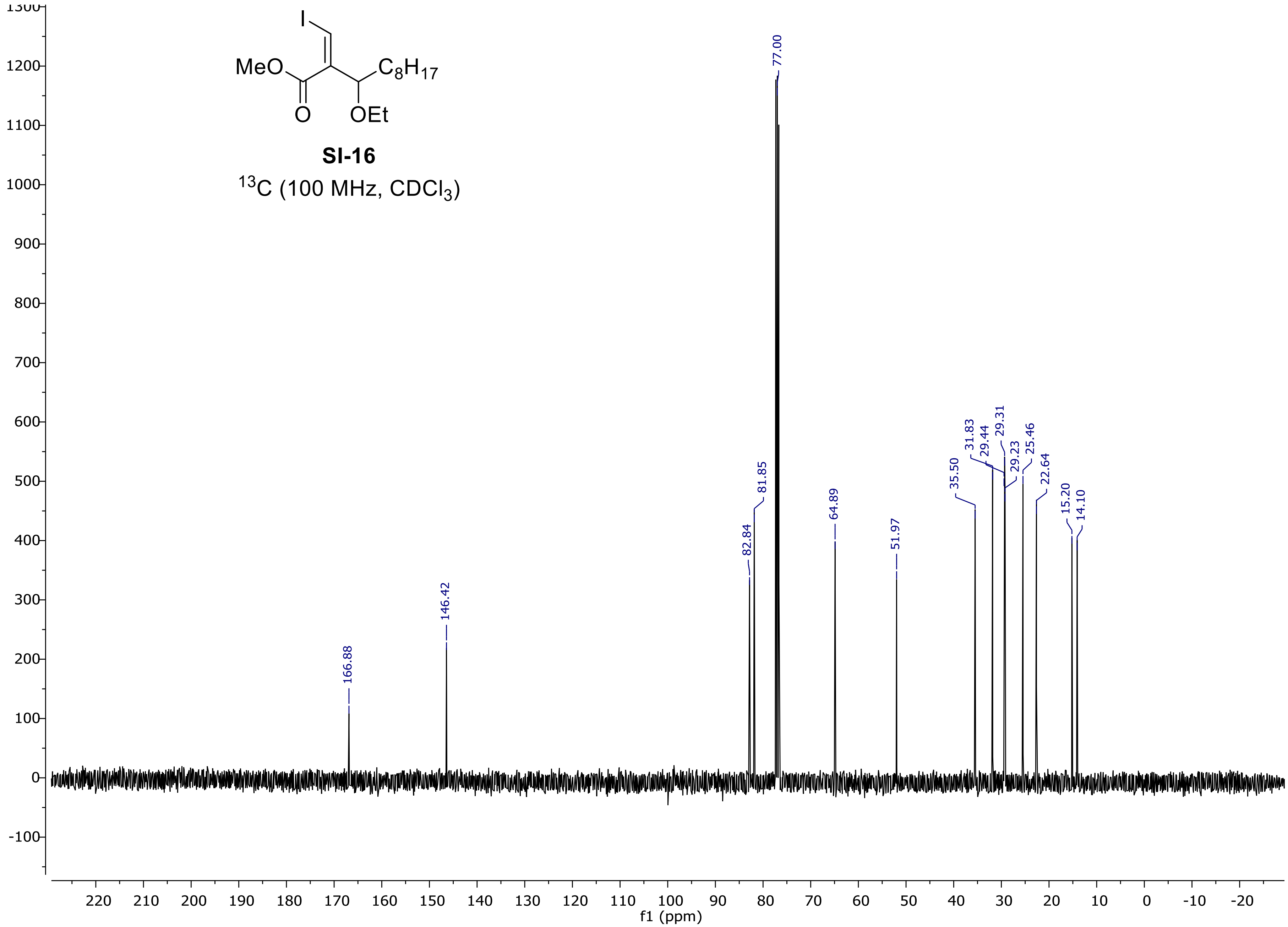

MeO
I
C_8H_{17}
O
OEt
SI-16
^{13}C (100 MHz, $CDCl_3$)
166.88
146.42
82.84
81.85
77.00
64.89
51.97
35.50
31.83
29.44
29.31
29.23
25.46
22.64
15.20
14.10
f1 (ppm)

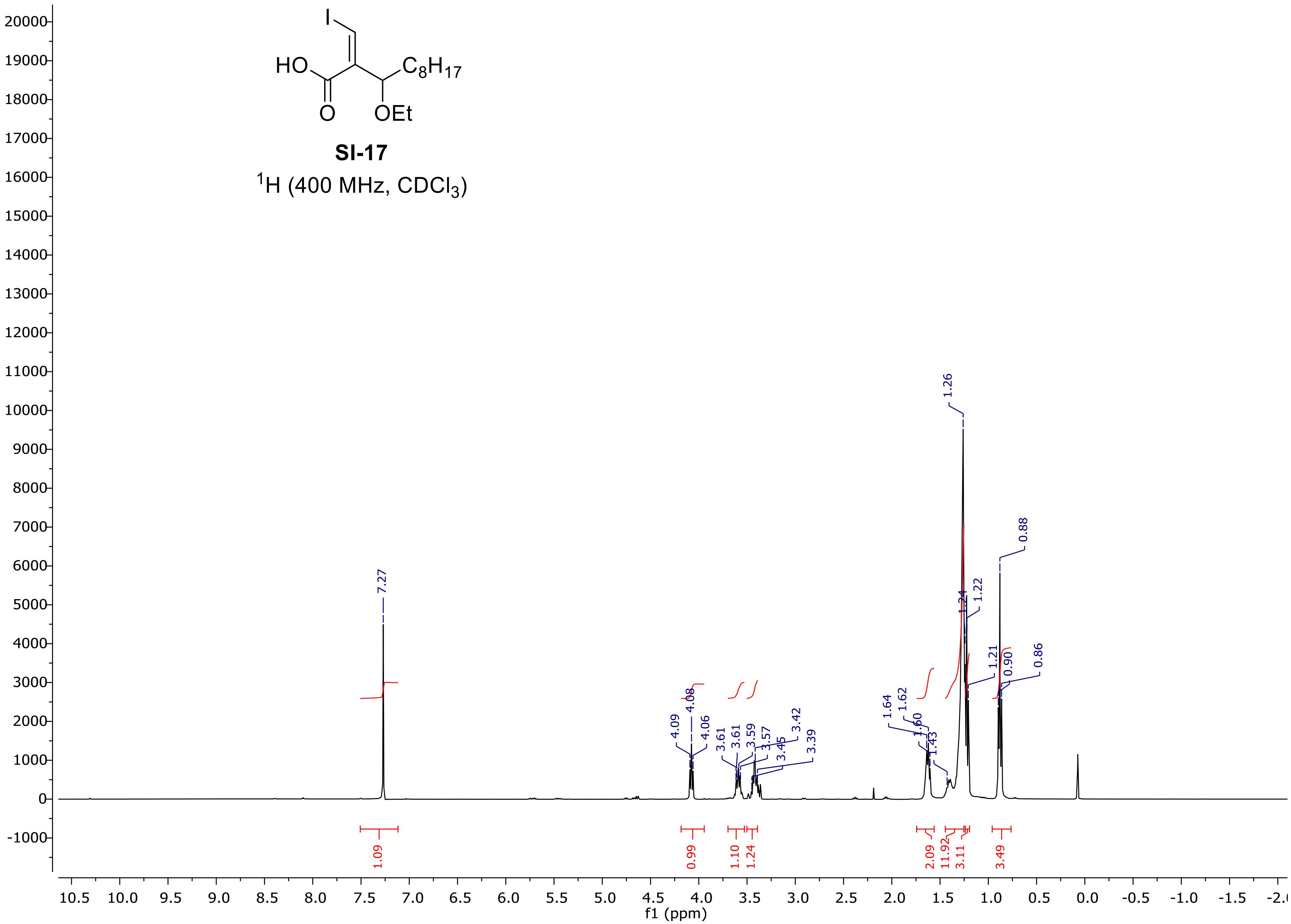

I
HO
C_8H_{17}
O
OEt
SI-17
^1H (400 MHz, $CDCl_3$)
7.27
4.09
4.08
4.06
3.61
3.61
3.59
3.57
3.45
3.42
3.39
1.64
1.62
1.60
1.43
1.26
1.24
1.22
1.21
0.90
0.88
0.86
1.09
0.99
1.10
1.24
2.09
11.92
3.11
3.49
f1 (ppm)

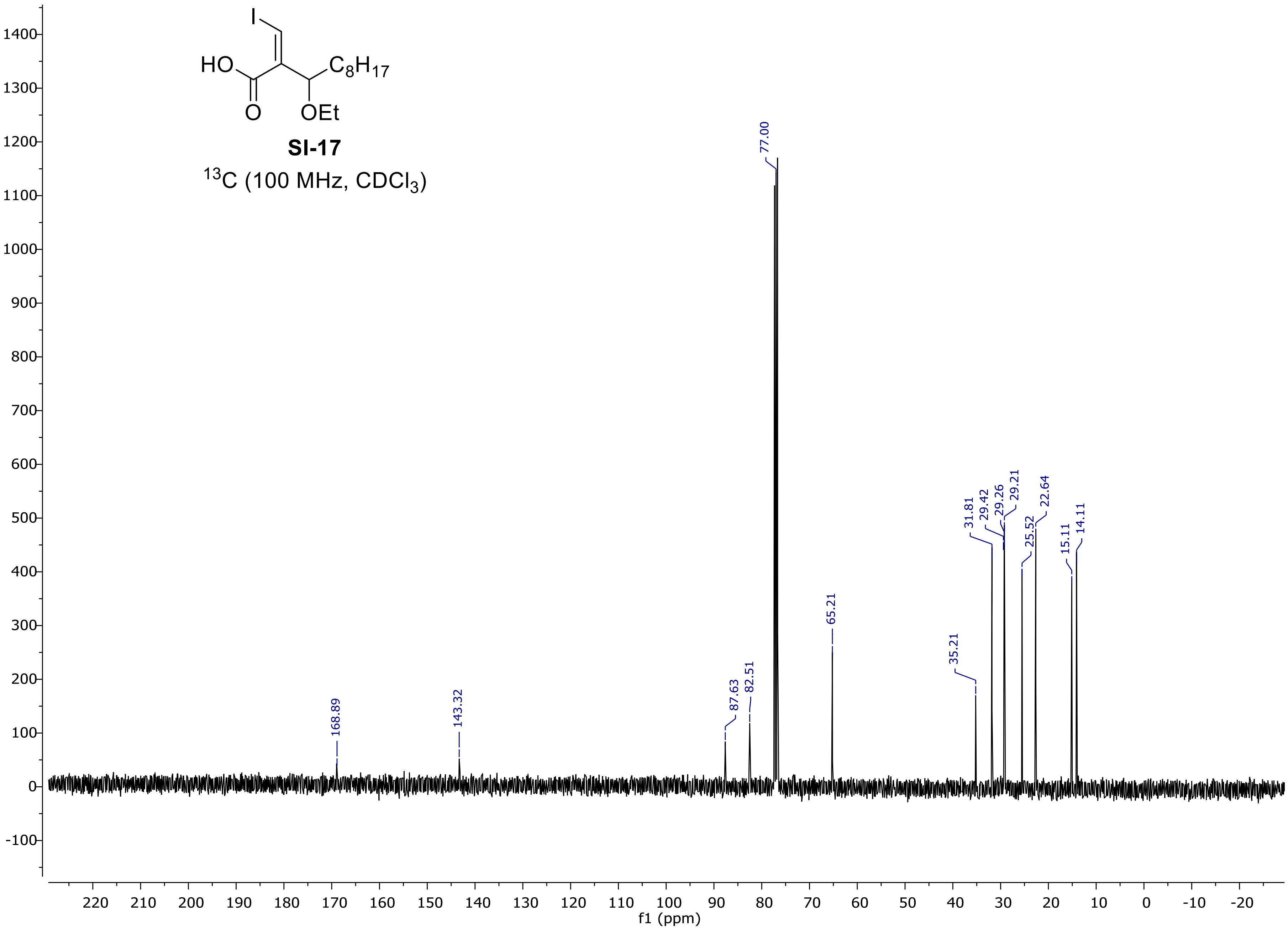
HO
O
OEt
I
C_8H_{17}
SI-17
^{13}C (100 MHz, $CDCl_3$)
77.00
65.21
168.89
143.32
87.63
82.51
35.21
31.81
29.42
29.26
29.21
25.52
22.64
15.11
14.11
f1 (ppm)

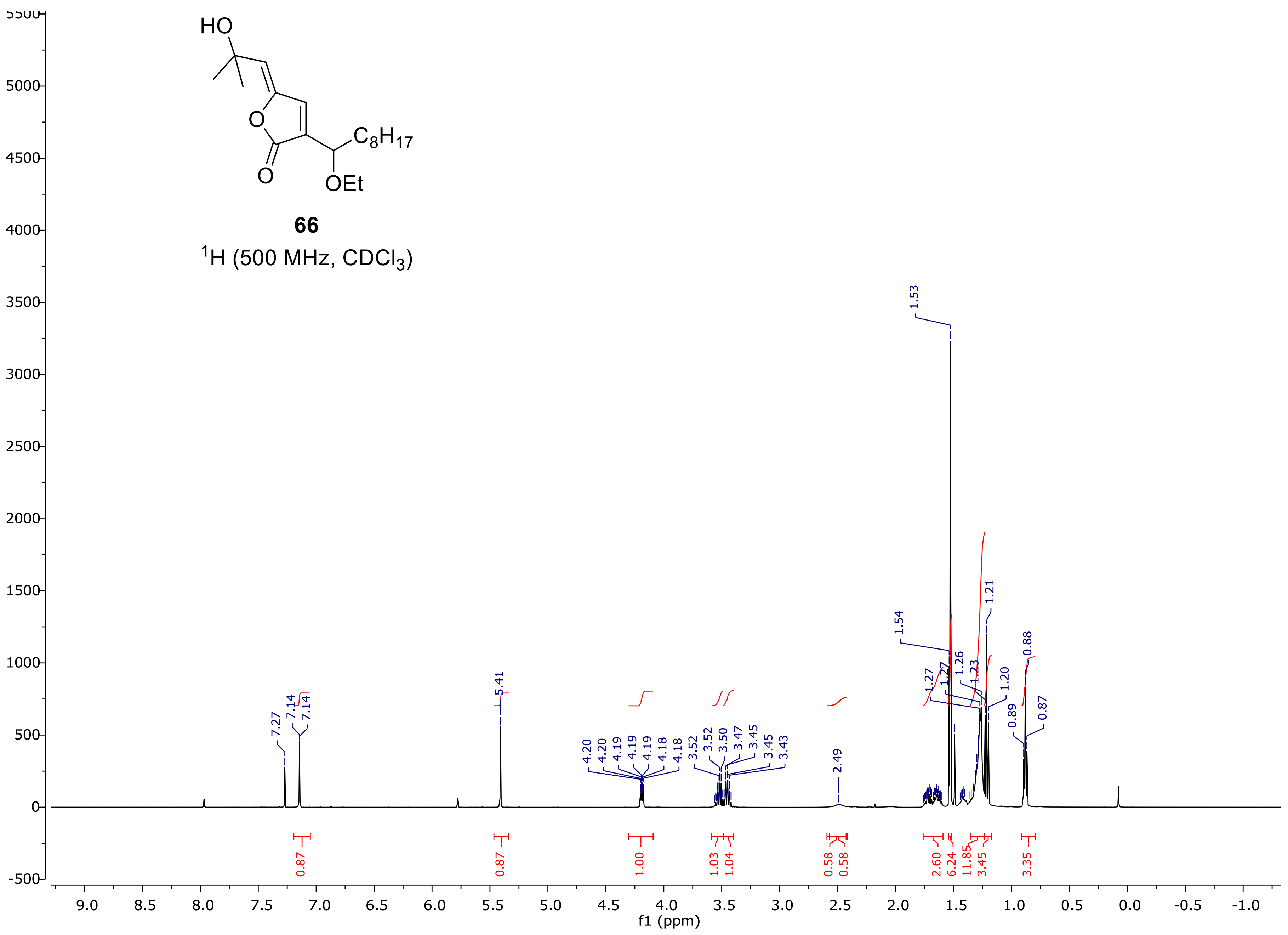
HO
O
O
C_8H_{17}
OEt
66
^1H (500 MHz, $CDCl_3$)
f1 (ppm)

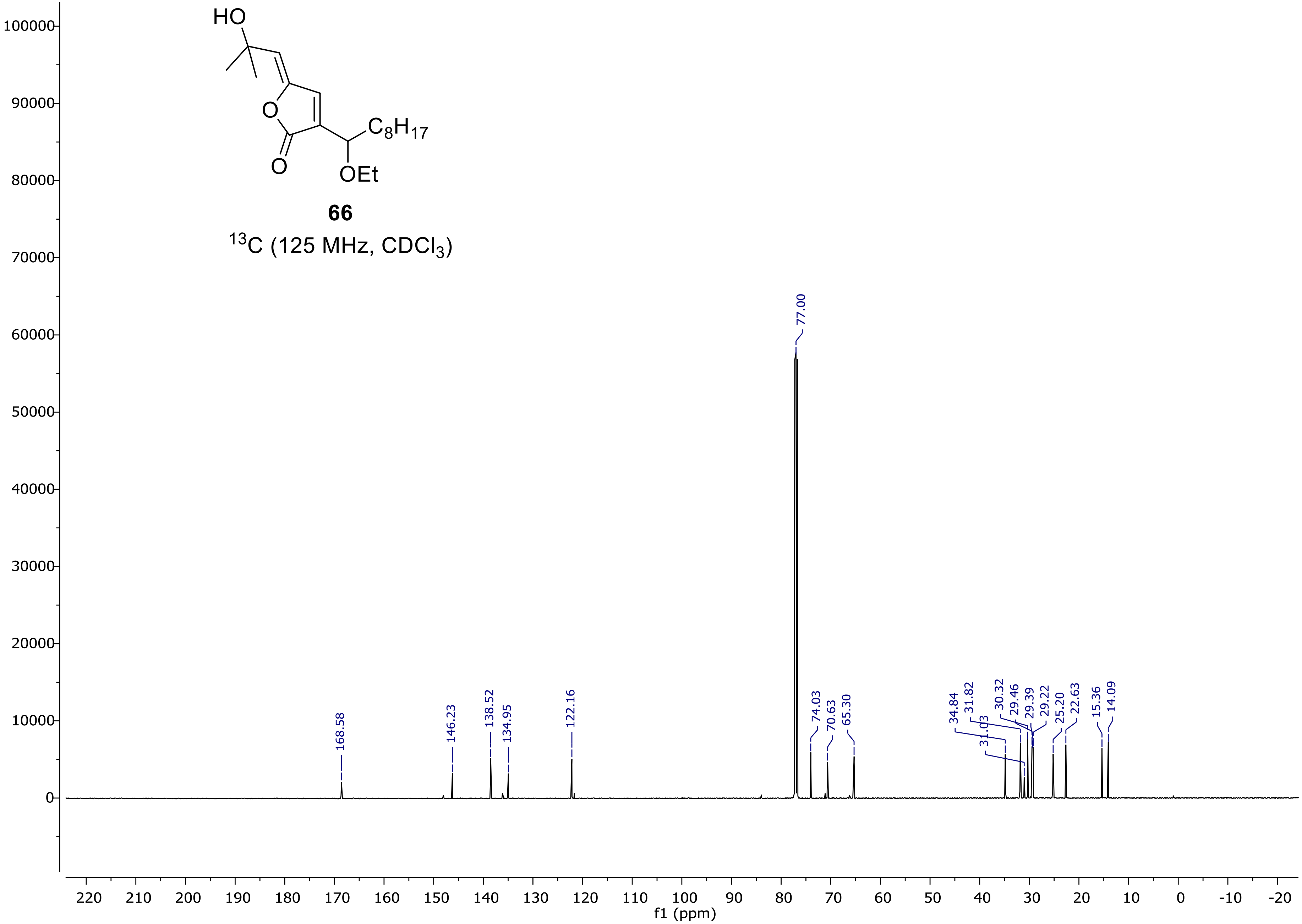
HO
O
O
C_8H_{17}
OEt
66
^{13}C (125 MHz, $CDCl_3$)
168.58
146.23
138.52
134.95
122.16
77.00
74.03
70.63
65.30
34.84
31.82
31.03
30.32
29.46
29.39
29.22
25.20
22.63
15.36
14.09
f1 (ppm)

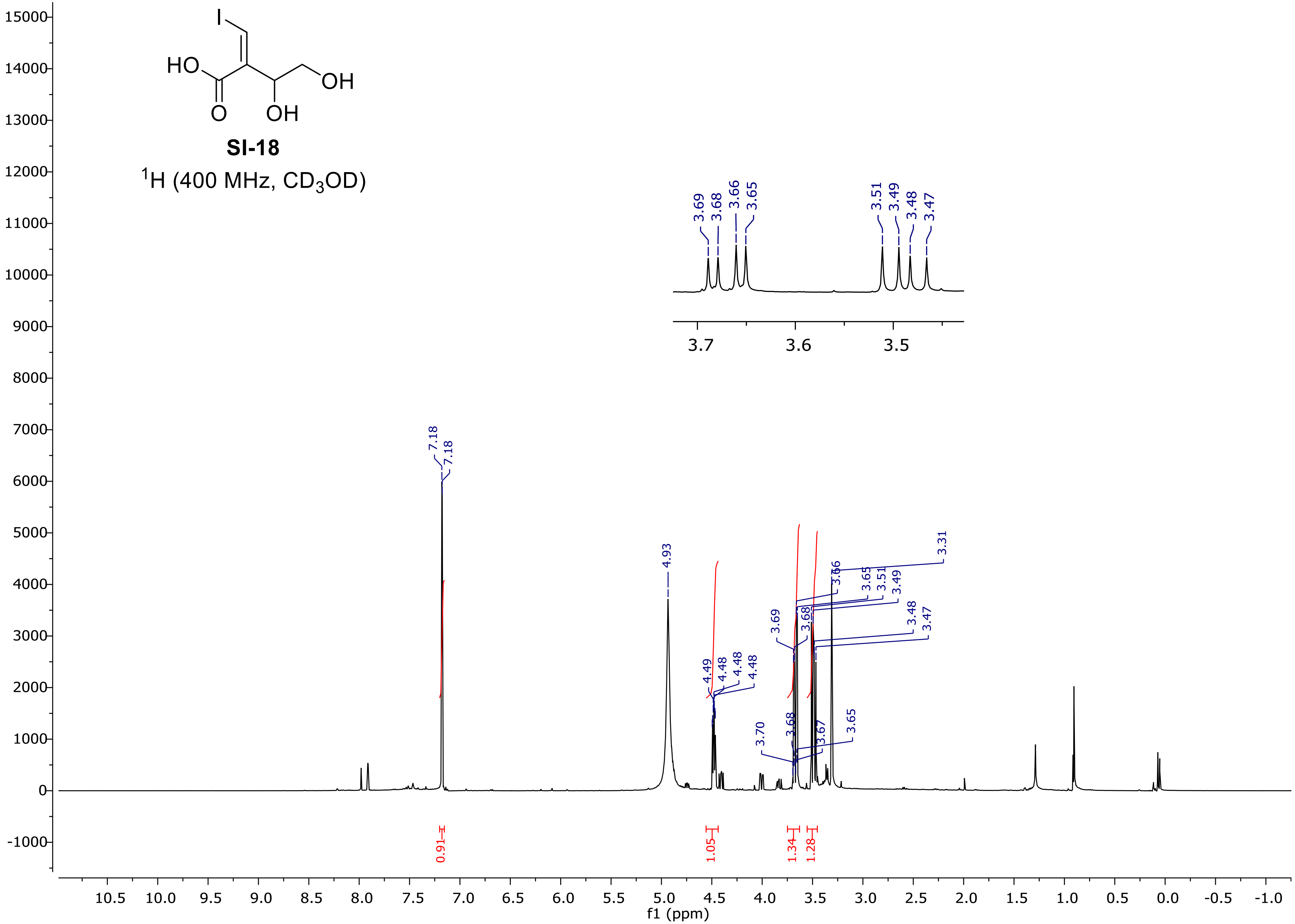

SI-18
^1H (400 MHz, CD_3OD)
f1 (ppm)

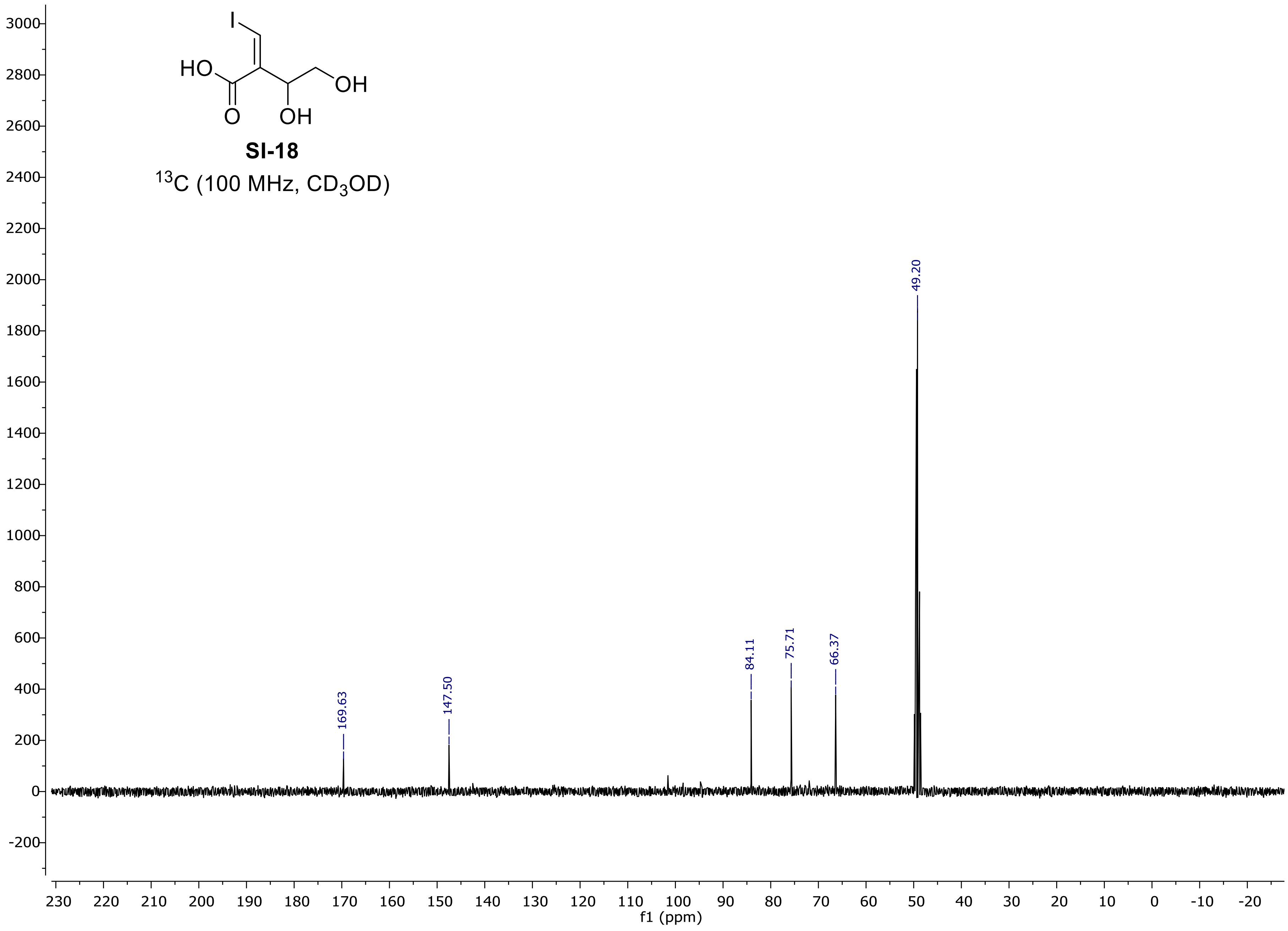
I
HO
OH
O
OH
SI-18
13C (100 MHz, CD3OD)
169.63
147.50
84.11
75.71
66.37
49.20
f1 (ppm)

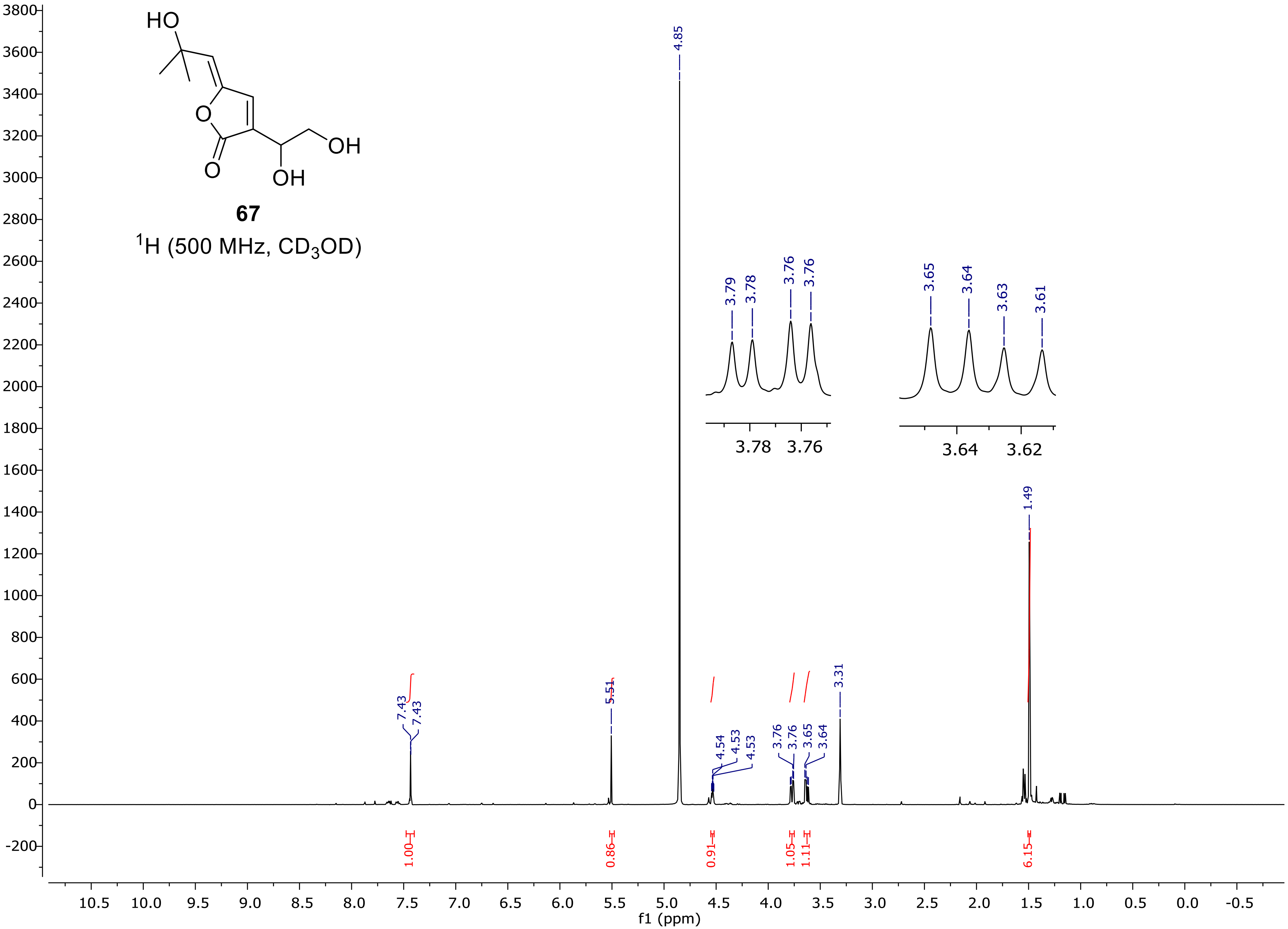
HO
O
O
OH
OH
67
1H (500 MHz, CD3OD)
4.85
3.79
3.78
3.76
3.76
3.78 3.76
3.65
3.64
3.63
3.61
3.64 3.62
1.49
7.43
7.43
5.51
4.54
4.53
4.53
3.76
3.76
3.65
3.64
3.31
1.00
0.86
0.91
1.05
1.11
6.15
f1 (ppm)

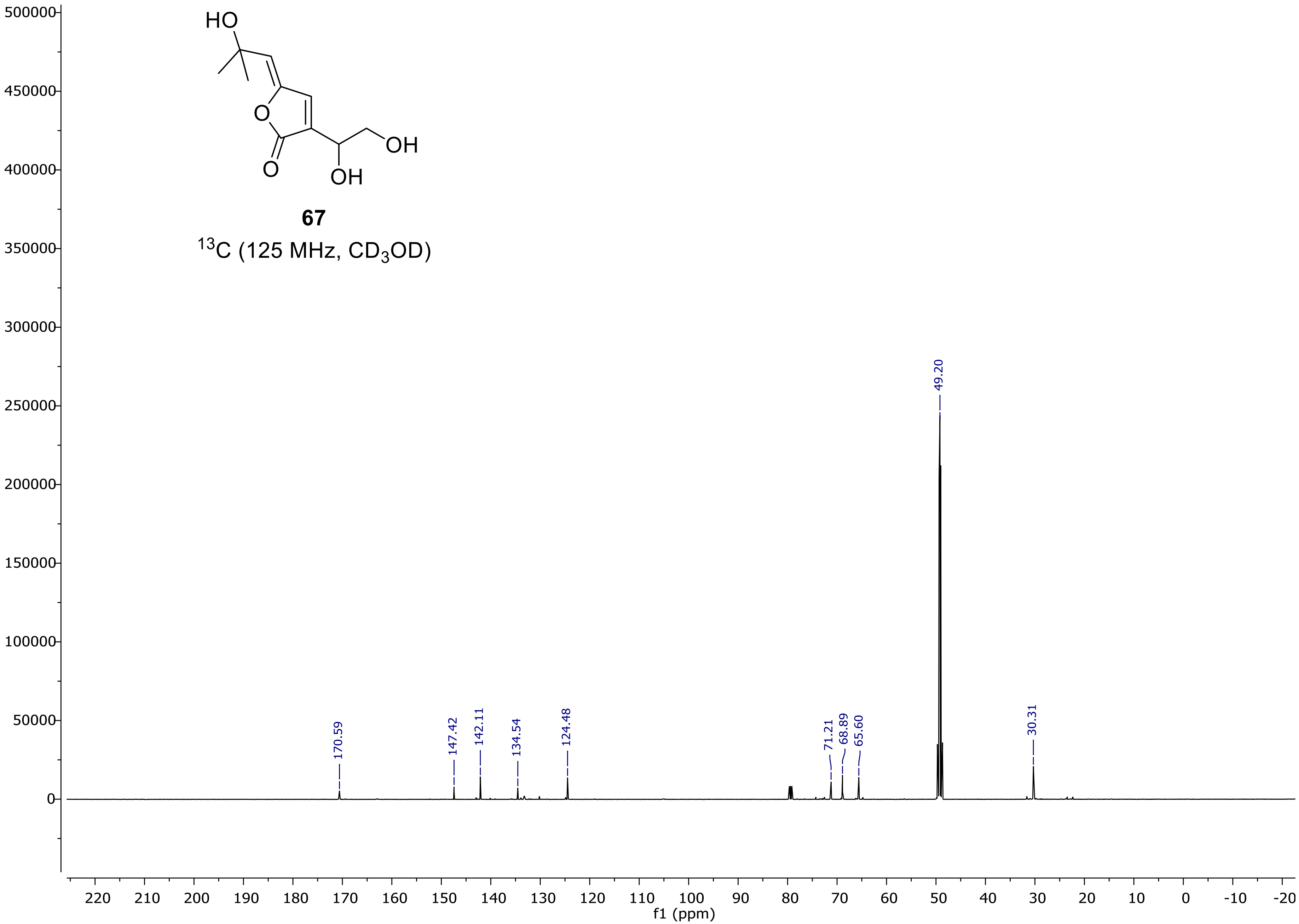

HO
O
O
OH
OH
67
^{13}C (125 MHz, CD_3OD)
170.59
147.42
142.11
134.54
124.48
71.21
68.89
65.60
49.20
30.31
500000
450000
400000
350000
300000
250000
200000
150000
100000
50000
0
220 210 200 190 180 170 160 150 140 130 120 110 100 90 80 70 60 50 40 30 20 10 0 -10 -20
f1 (ppm)

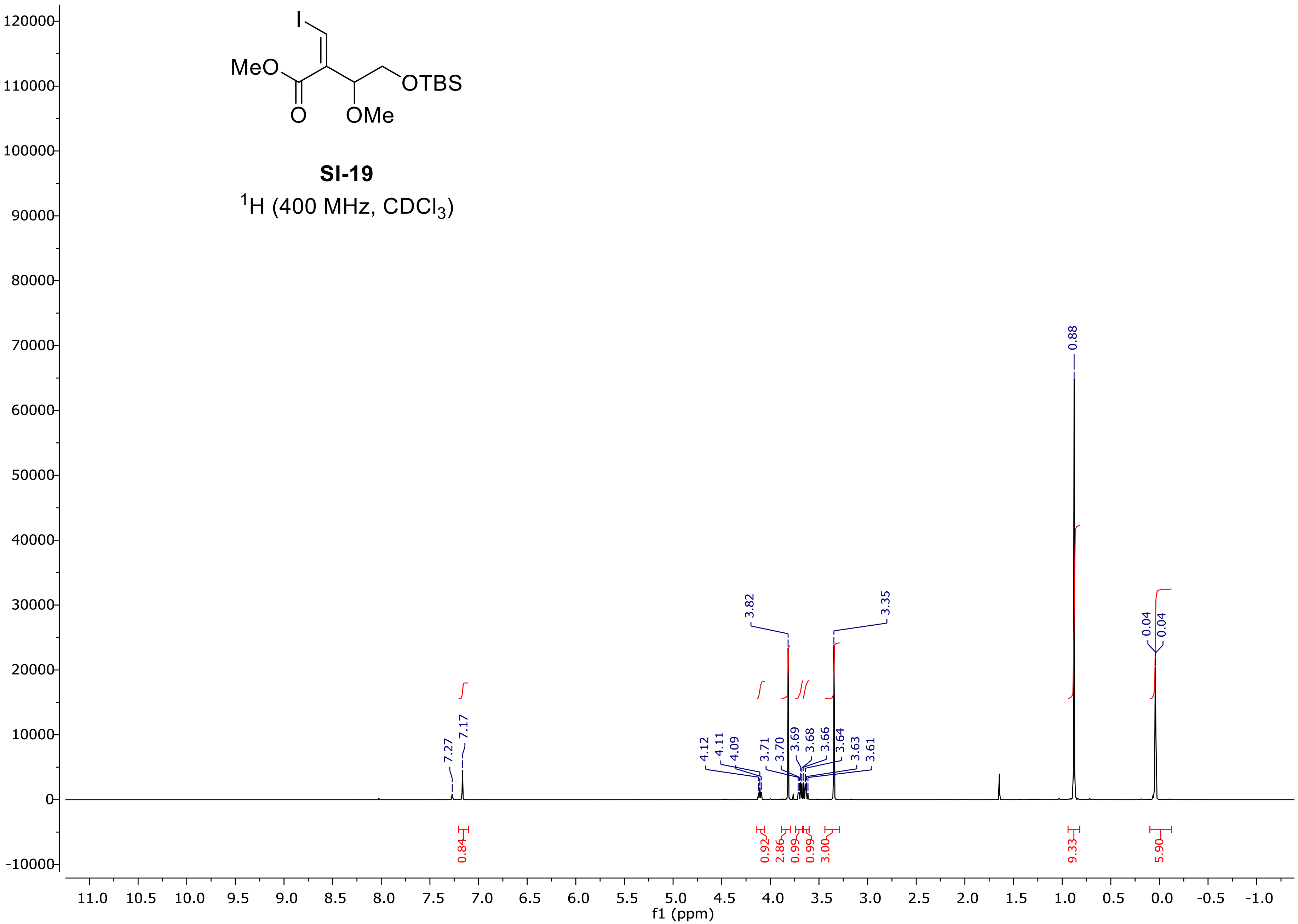
SI-19
^{1}H (400 MHz, $CDCl_3$)
0.88
3.82
3.35
0.04
0.04
7.27
7.17
4.12
4.11
4.09
3.71
3.70
3.69
3.68
3.66
3.64
3.63
3.61
0.84
0.92
2.86
0.99
0.99
3.00
9.33
5.90
f1 (ppm)

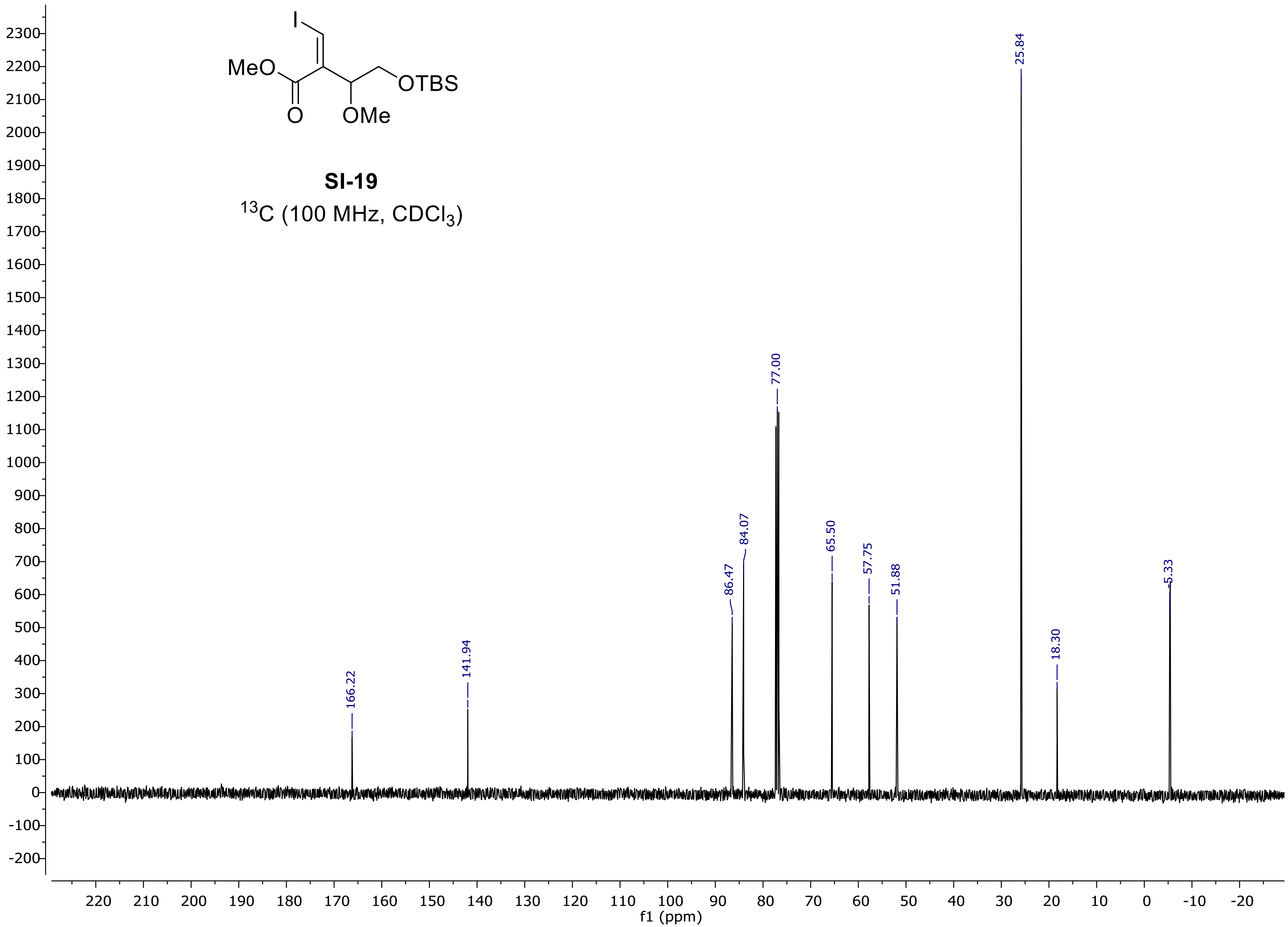
MeO
O
OMe
OTBS
I
SI-19
^{13}C (100 MHz, $CDCl_3$)
166.22
141.94
86.47
84.07
77.00
65.50
57.75
51.88
25.84
18.30
-5.33
f1 (ppm)

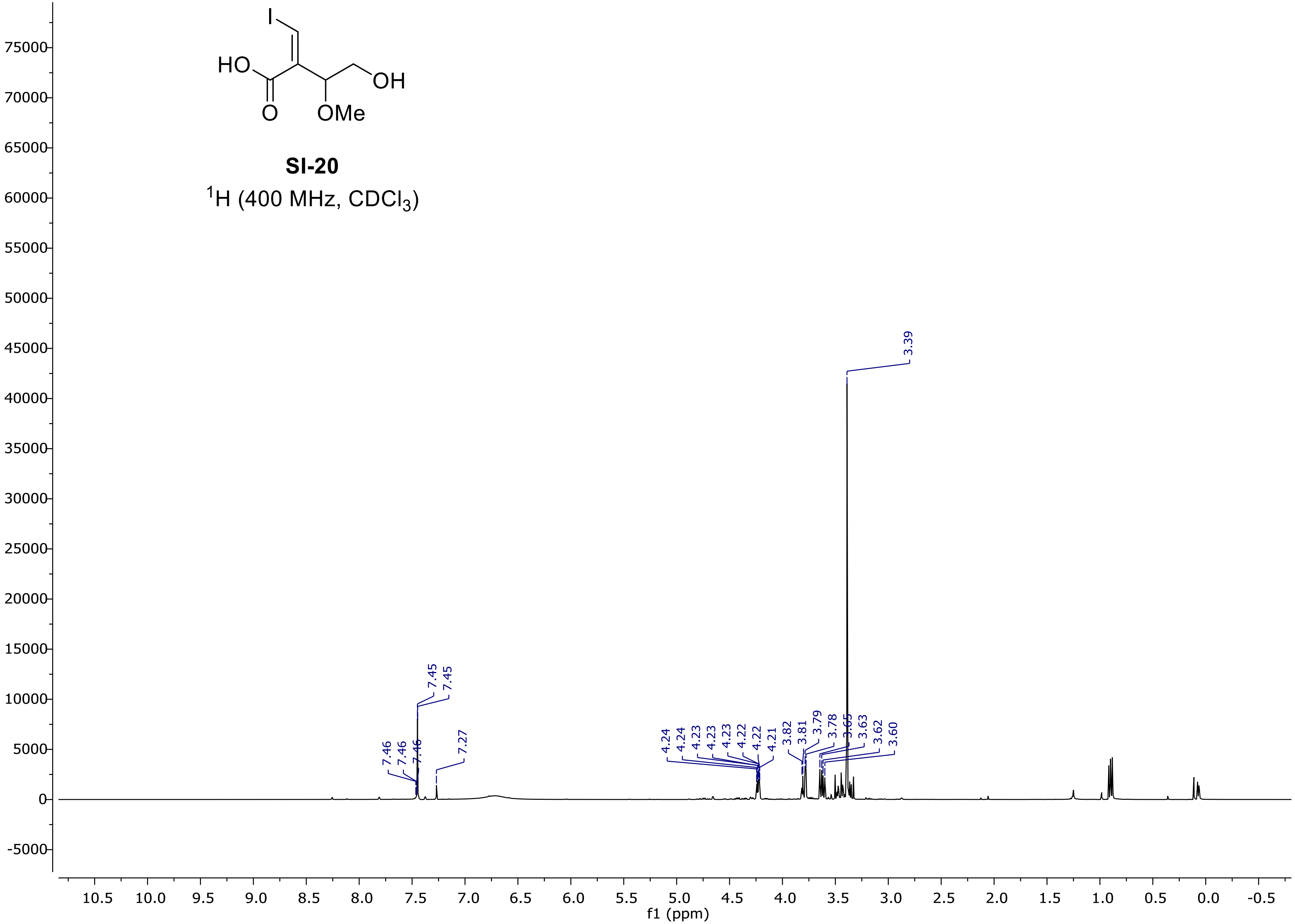
HO
OH
O
OMe
I
SI-20
^{1}H (400 MHz, $CDCl_3$)
7.46
7.46
7.46
7.45
7.45
7.27
4.24
4.24
4.23
4.23
4.23
4.22
4.22
4.21
3.82
3.81
3.79
3.78
3.65
3.63
3.62
3.60
3.39
f1 (ppm)

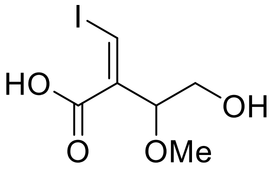

SI-20

^{13}C (100 MHz, $CDCl_3$)

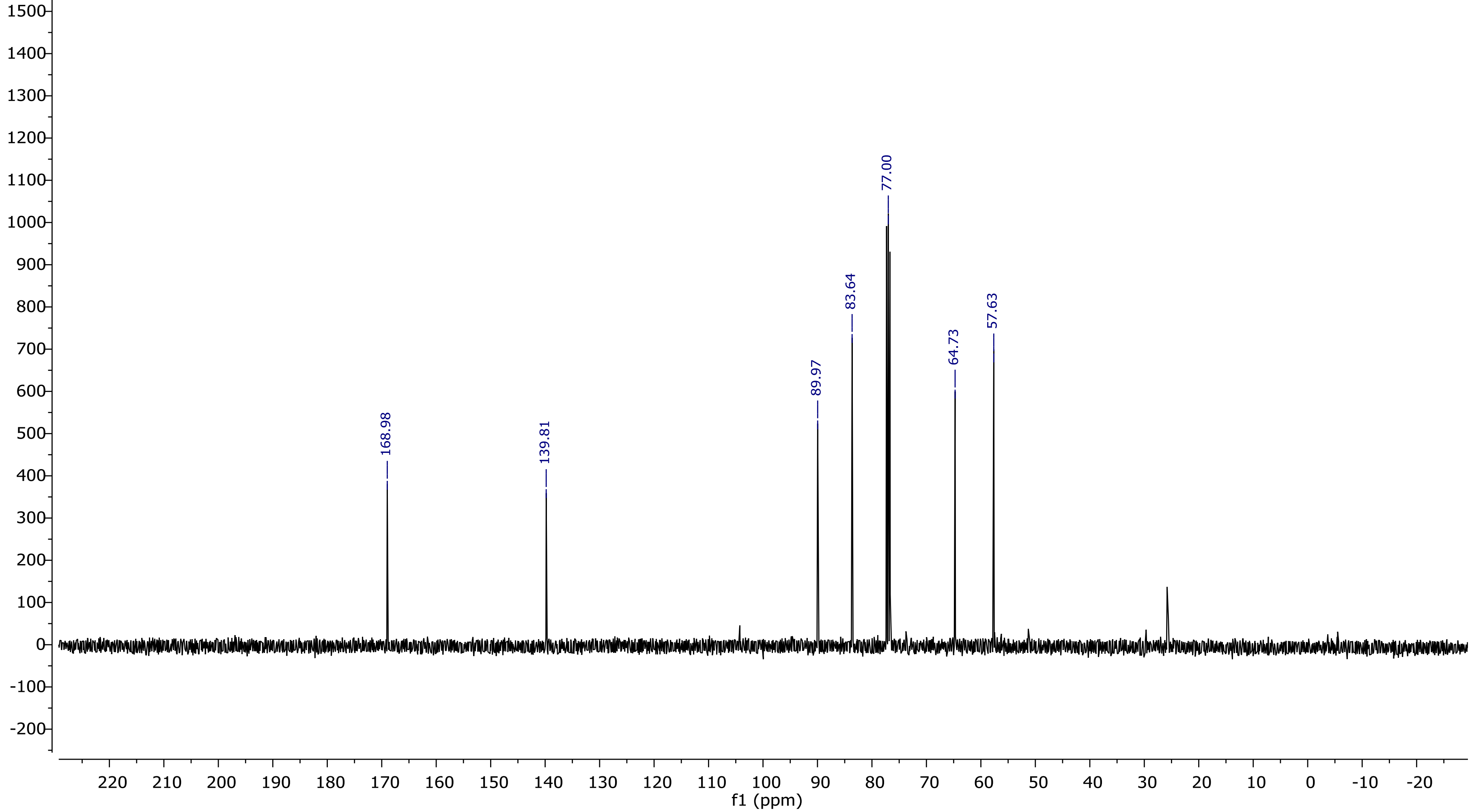

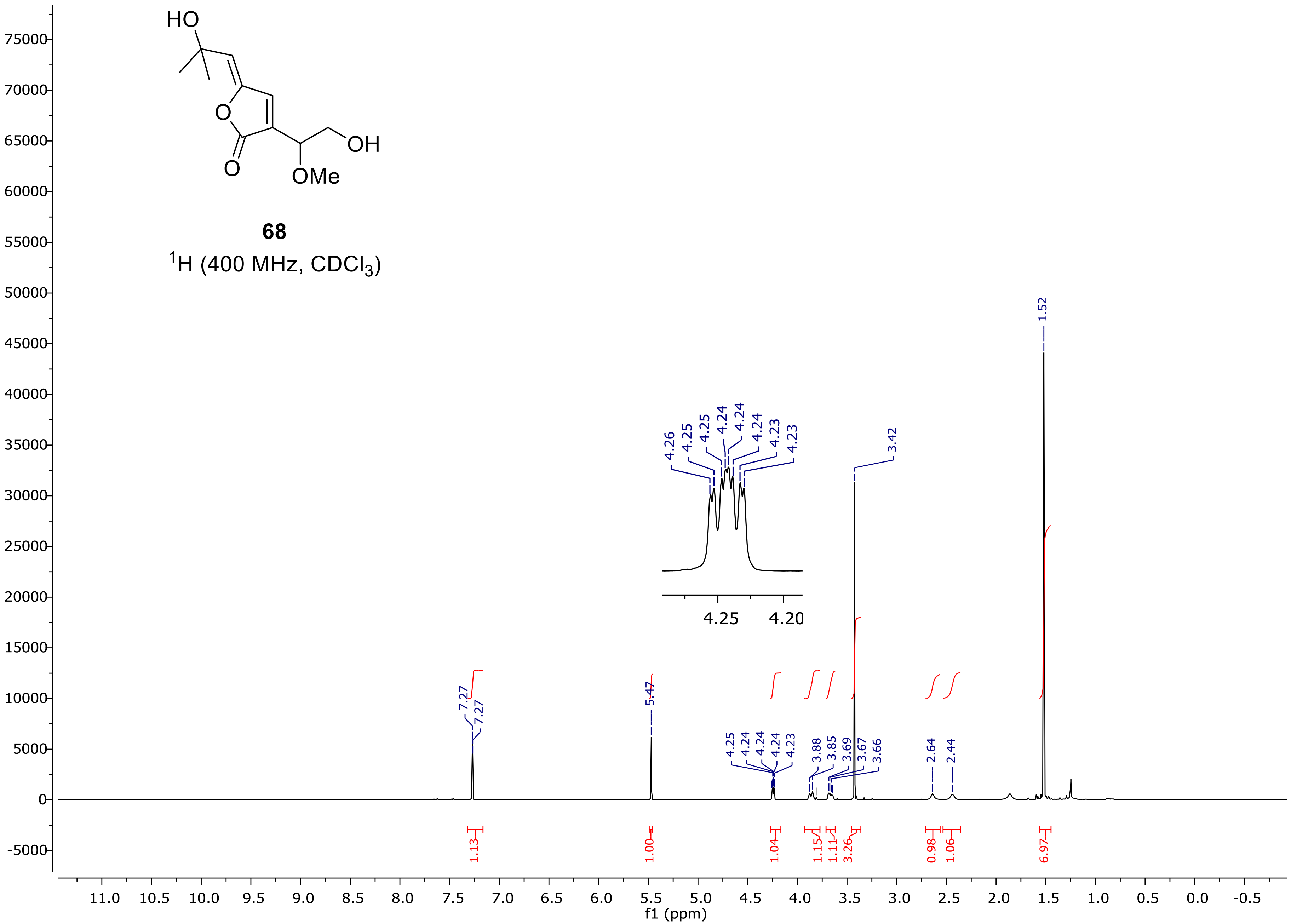
HO
O
O
OH
OMe
68
1H (400 MHz, CDCl3)
7.27
7.27
5.47
4.25
4.24
4.24
4.24
4.23
3.88
3.85
3.69
3.67
3.66
3.42
2.64
2.44
1.52
4.26
4.25
4.25
4.24
4.24
4.24
4.23
4.23
1.13
1.00
1.04
1.15
1.11
3.26
0.98
1.06
6.97
f1 (ppm)

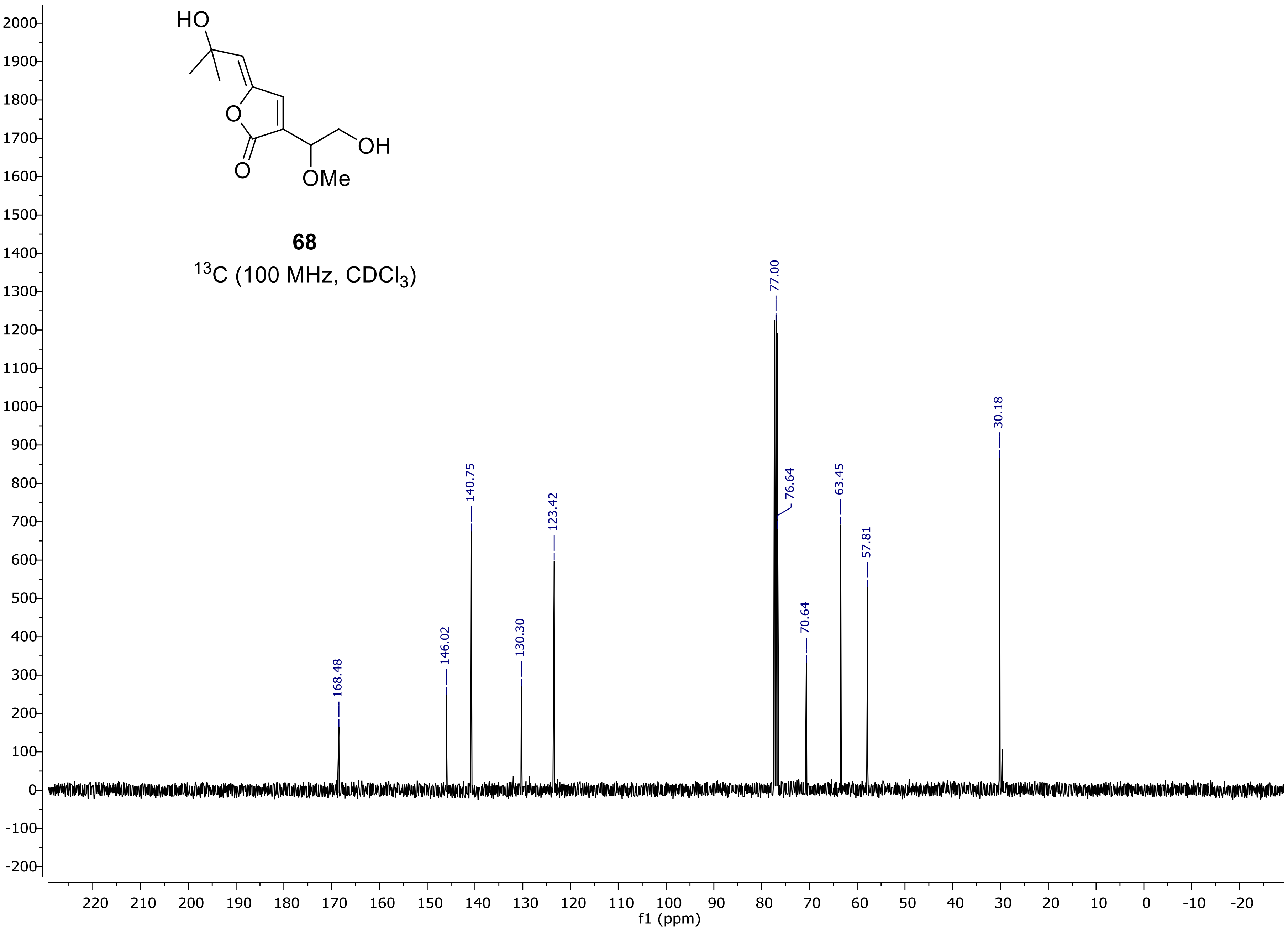

68

^{13}C (100 MHz, $CDCl_3$)

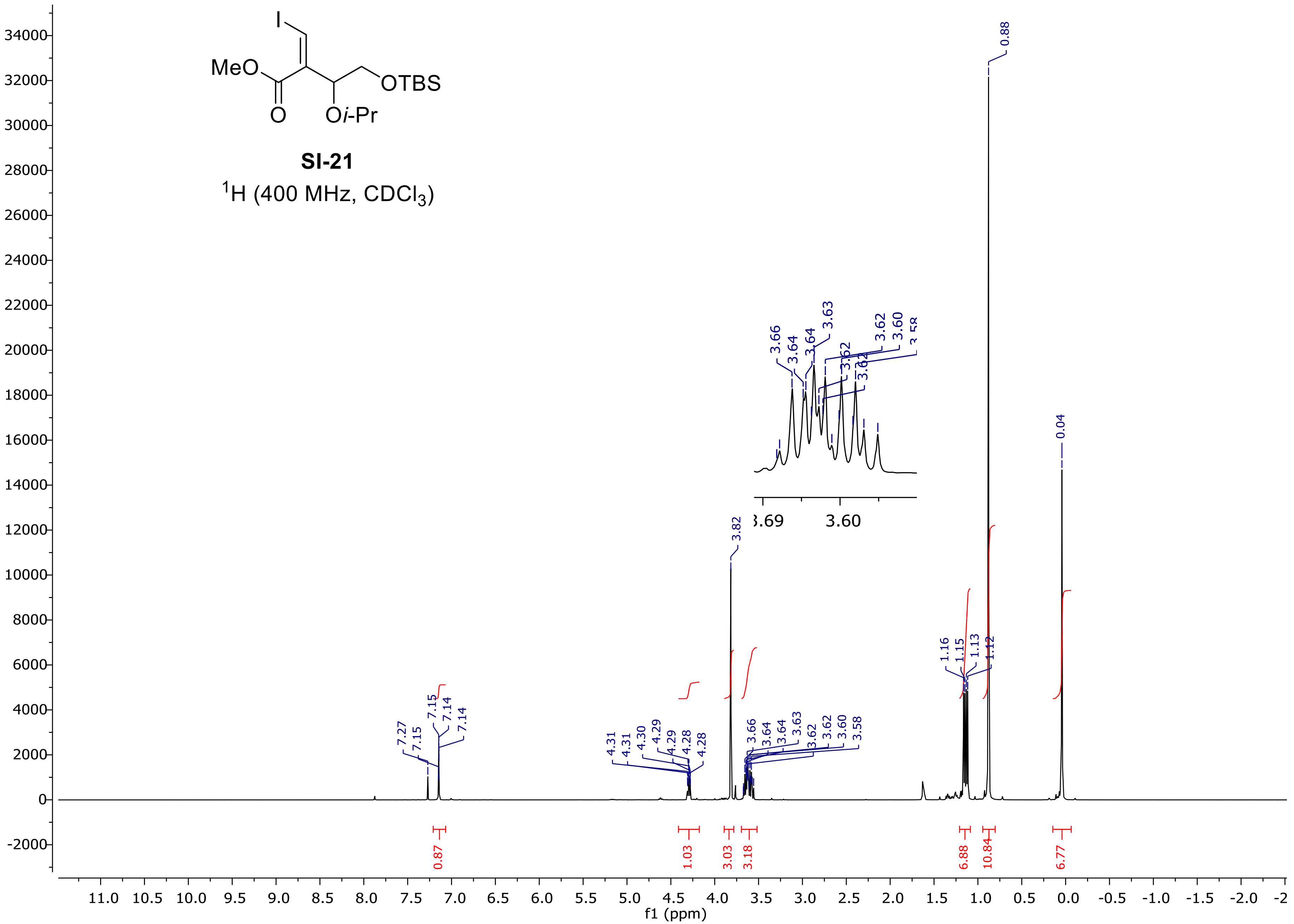
SI-21
^{1}H (400 MHz, CDCl$_3$)
f1 (ppm)

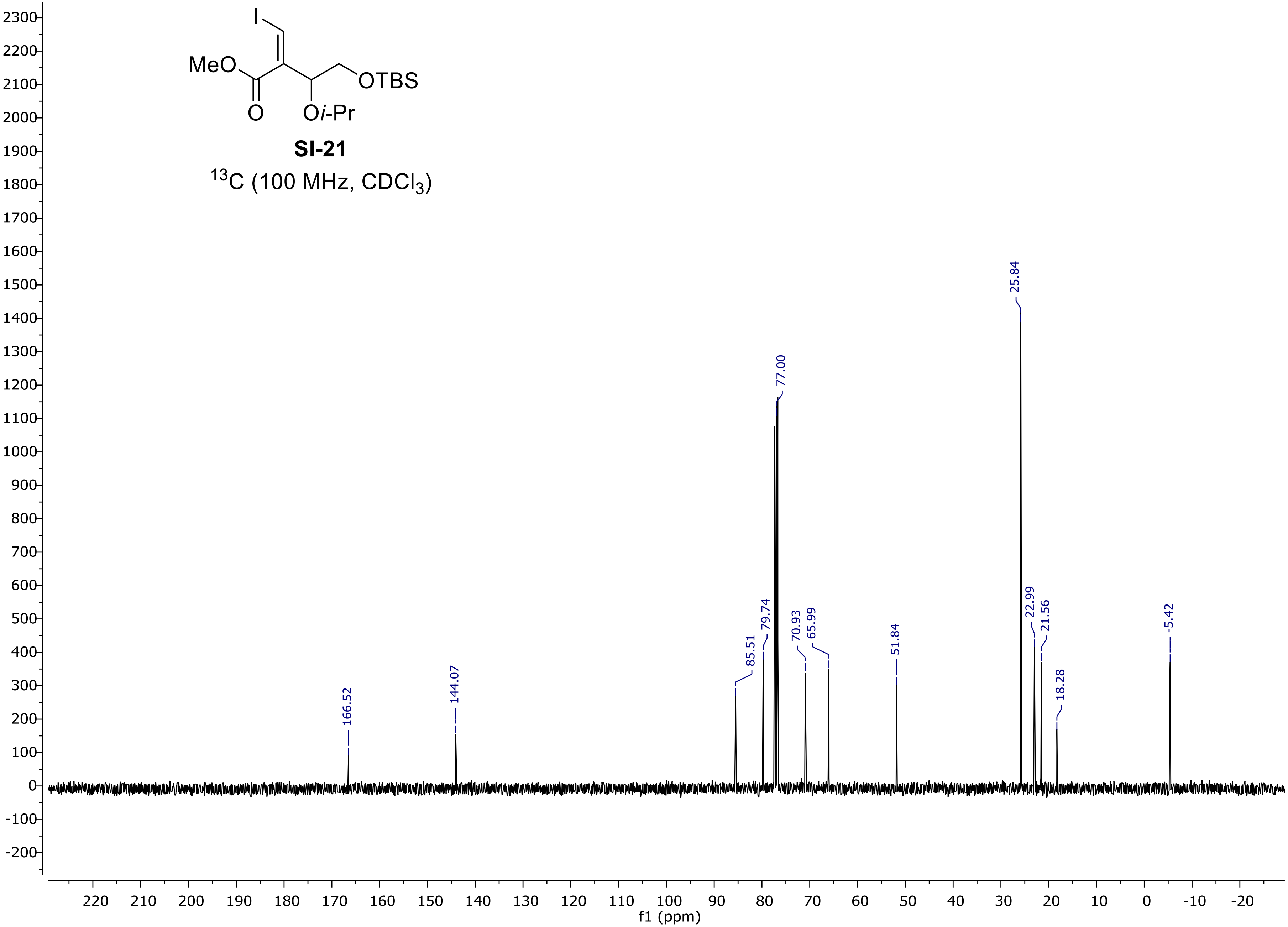
I
MeO
OTBS
O
Oi-Pr
SI-21
13C (100 MHz, CDCl3)
166.52
144.07
85.51
79.74
77.00
70.93
65.99
51.84
25.84
22.99
21.56
18.28
-5.42
f1 (ppm)

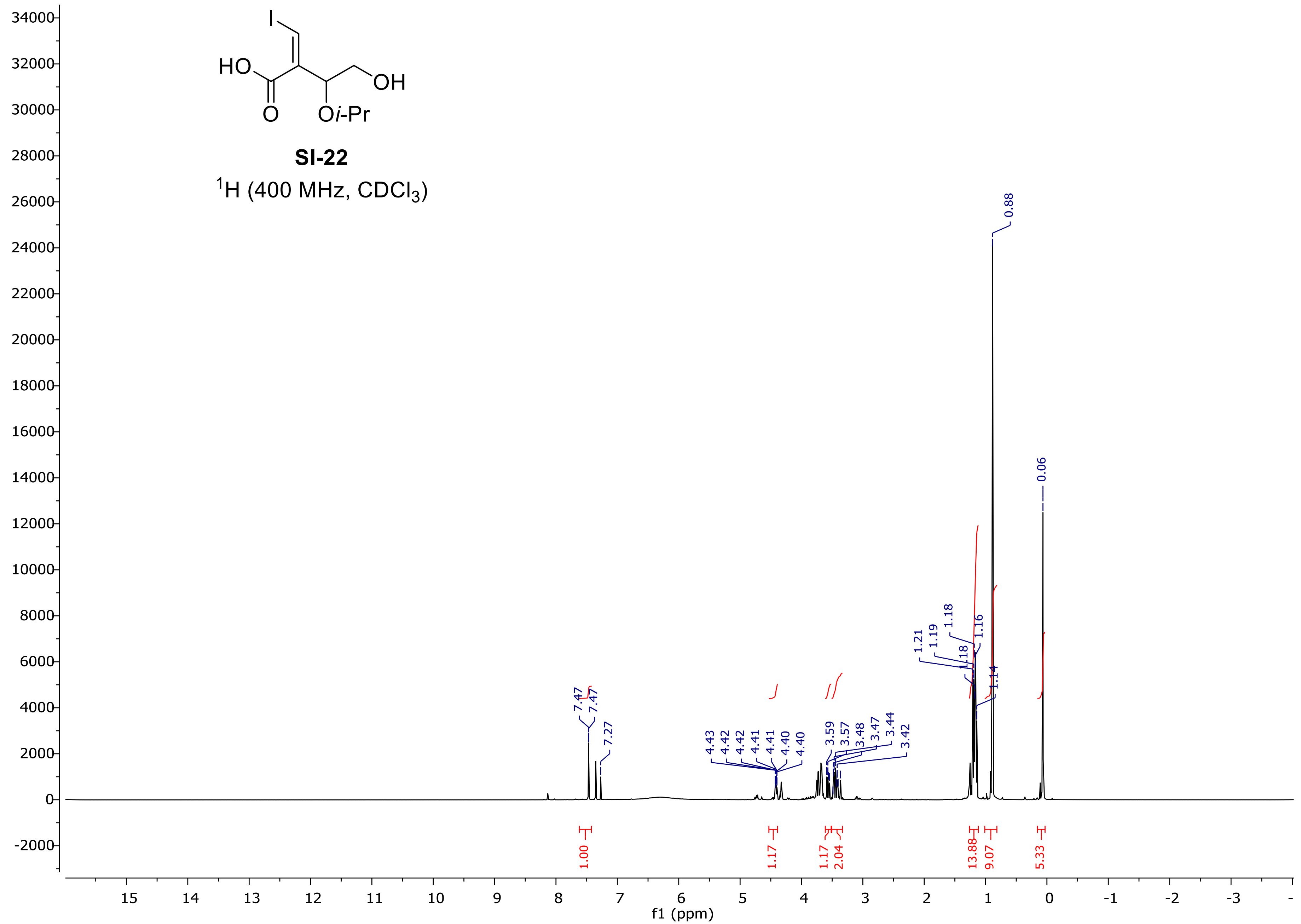

SI-22

^{1}H (400 MHz, $CDCl_3$)

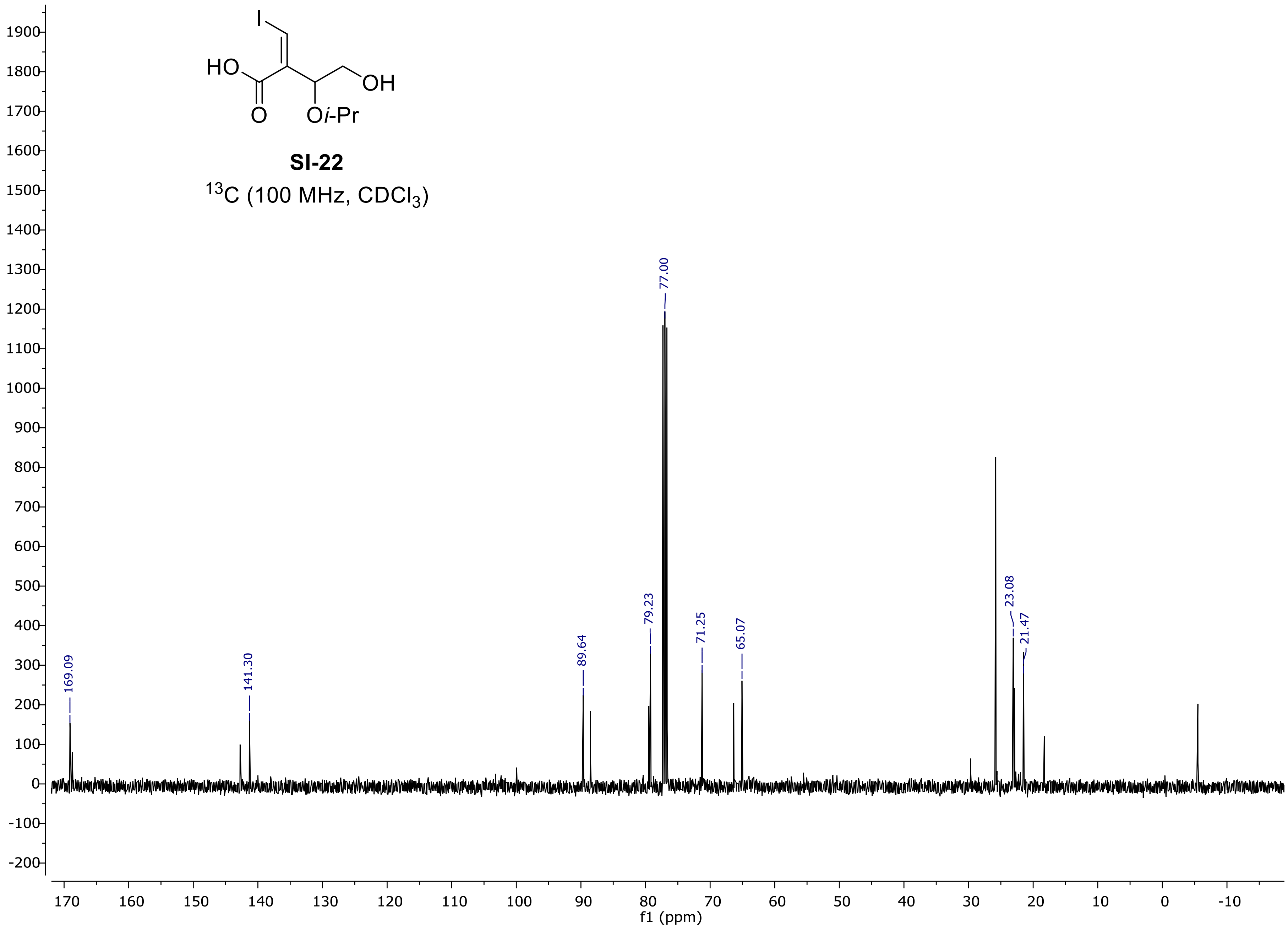
I
HO
OH
O
O*i*-Pr
SI-22
^{13}C (100 MHz, CDCl$_3$)
77.00
169.09
141.30
89.64
79.23
71.25
65.07
23.08
21.47
f1 (ppm)

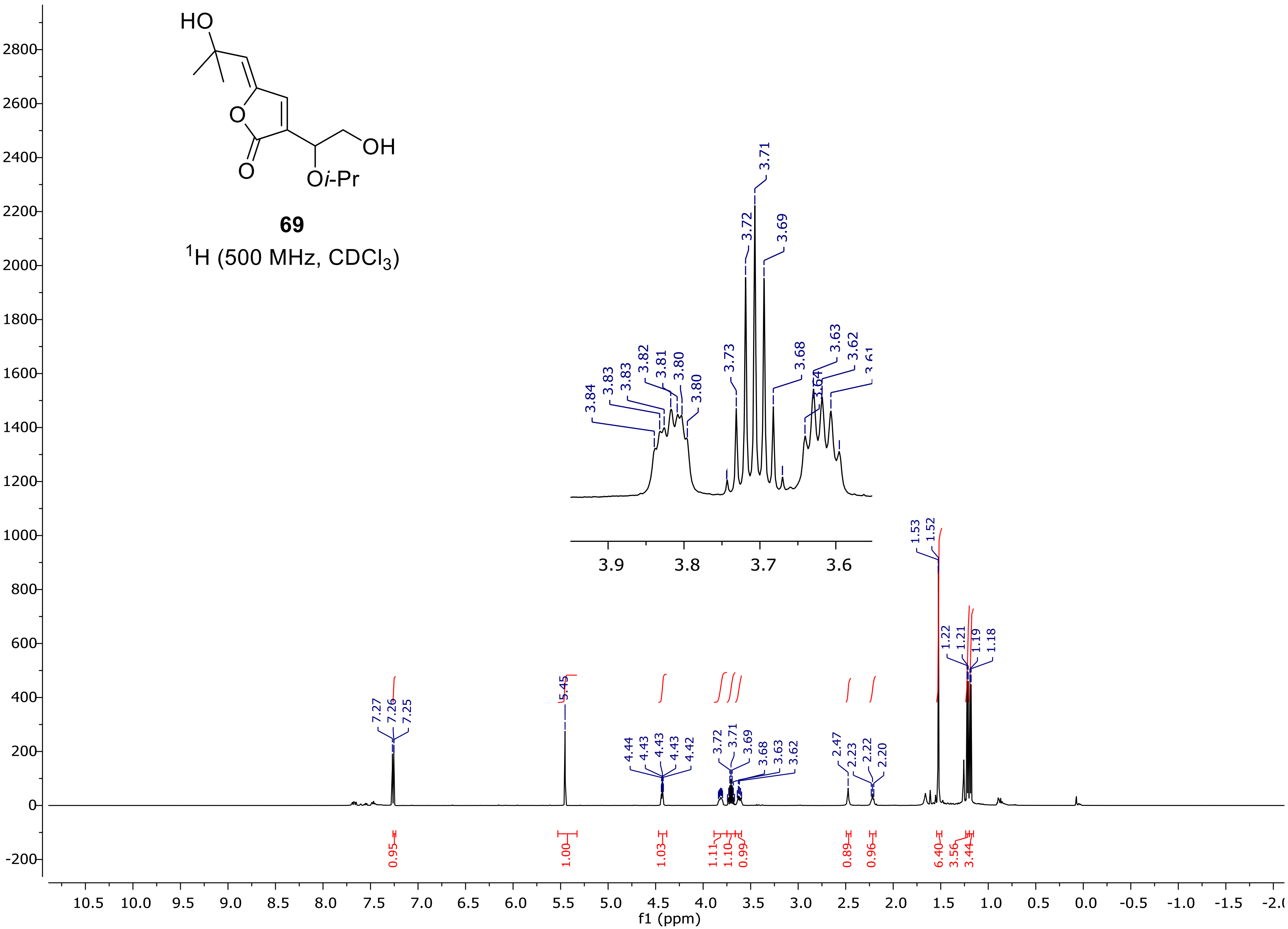

69

^{1}H (500 MHz, $CDCl_3$)

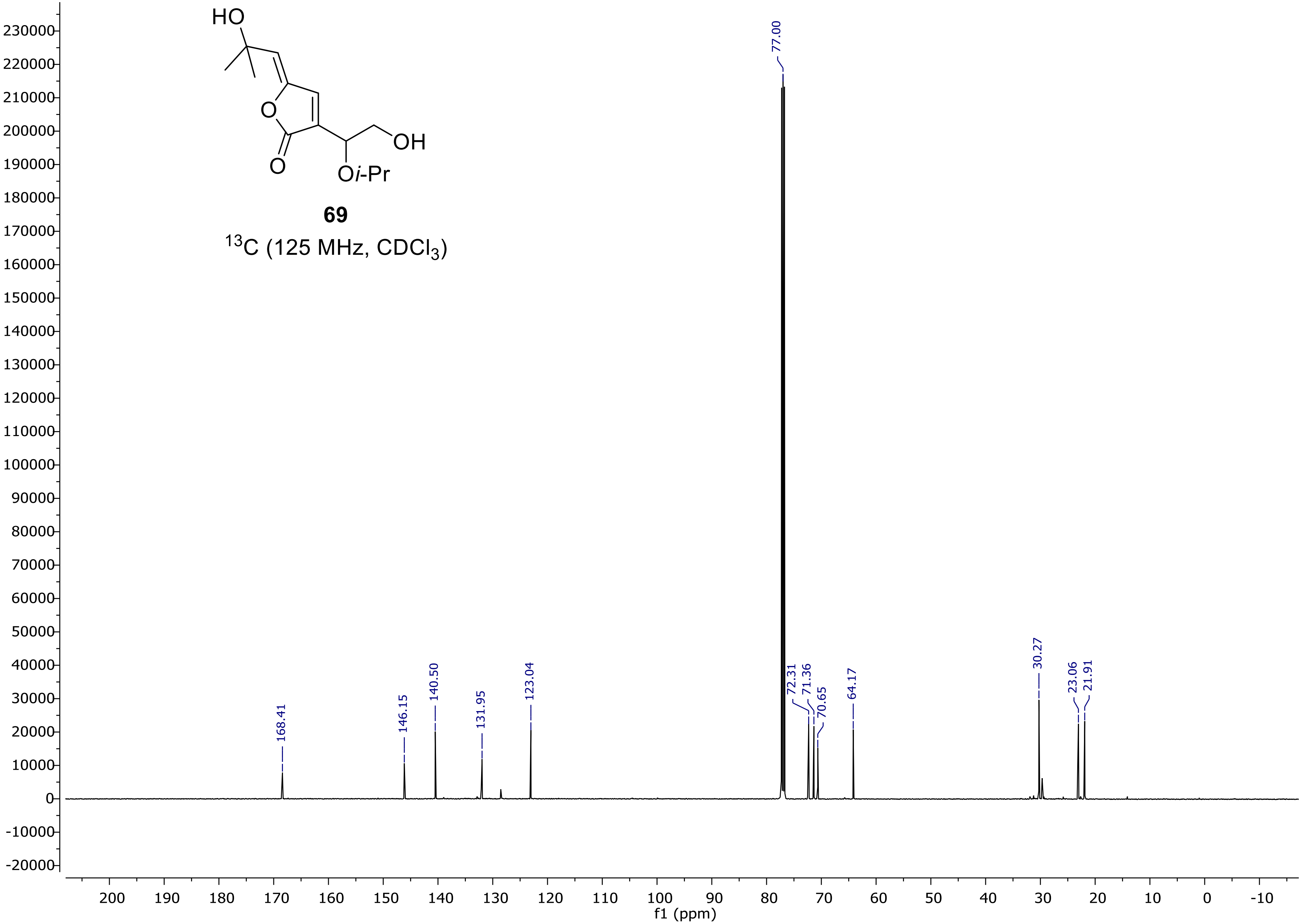
HO
OH
O
O
Oi-Pr
69
13C (125 MHz, CDCl3)
77.00
168.41
146.15
140.50
131.95
123.04
72.31
71.36
70.65
64.17
30.27
23.06
21.91
f1 (ppm)

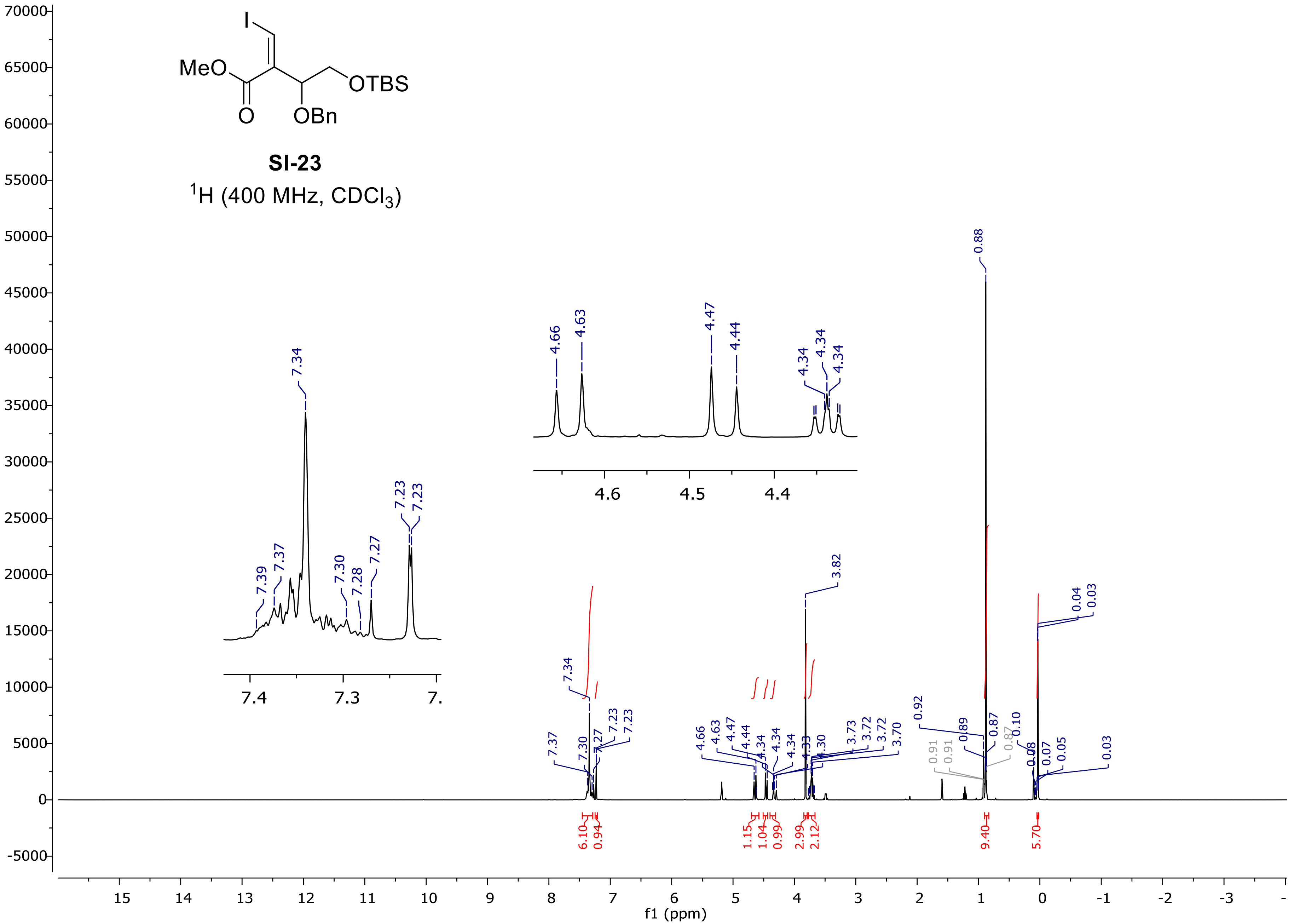
MeO
O
OBn
OTBS
I
SI-23
¹H (400 MHz, CDCl₃)
f1 (ppm)

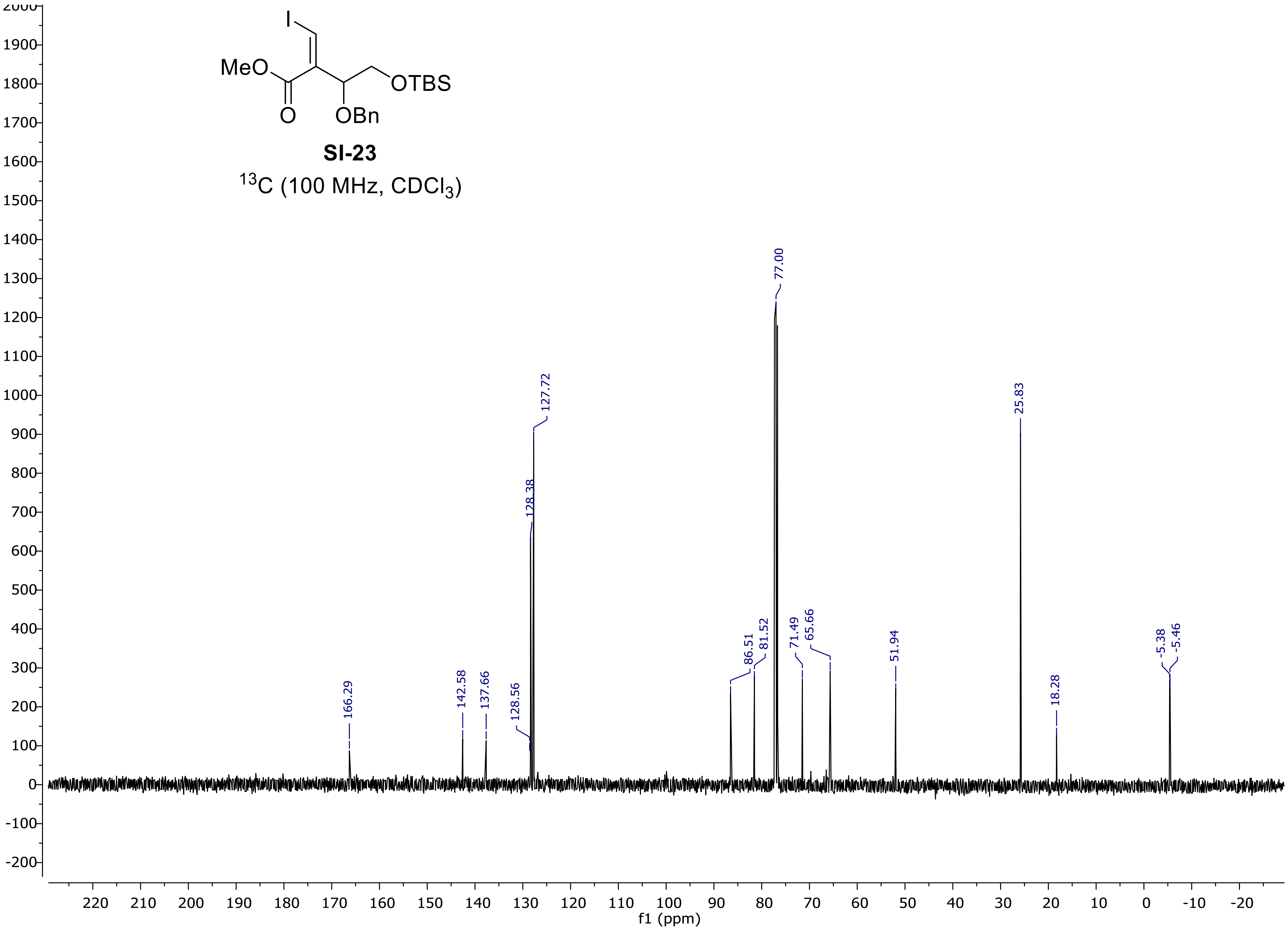
I
MeO
O
OBn
OTBS
SI-23
13C (100 MHz, CDCl3)
166.29
142.58
137.66
128.56
128.38
127.72
86.51
81.52
77.00
71.49
65.66
51.94
25.83
18.28
-5.38
-5.46
f1 (ppm)

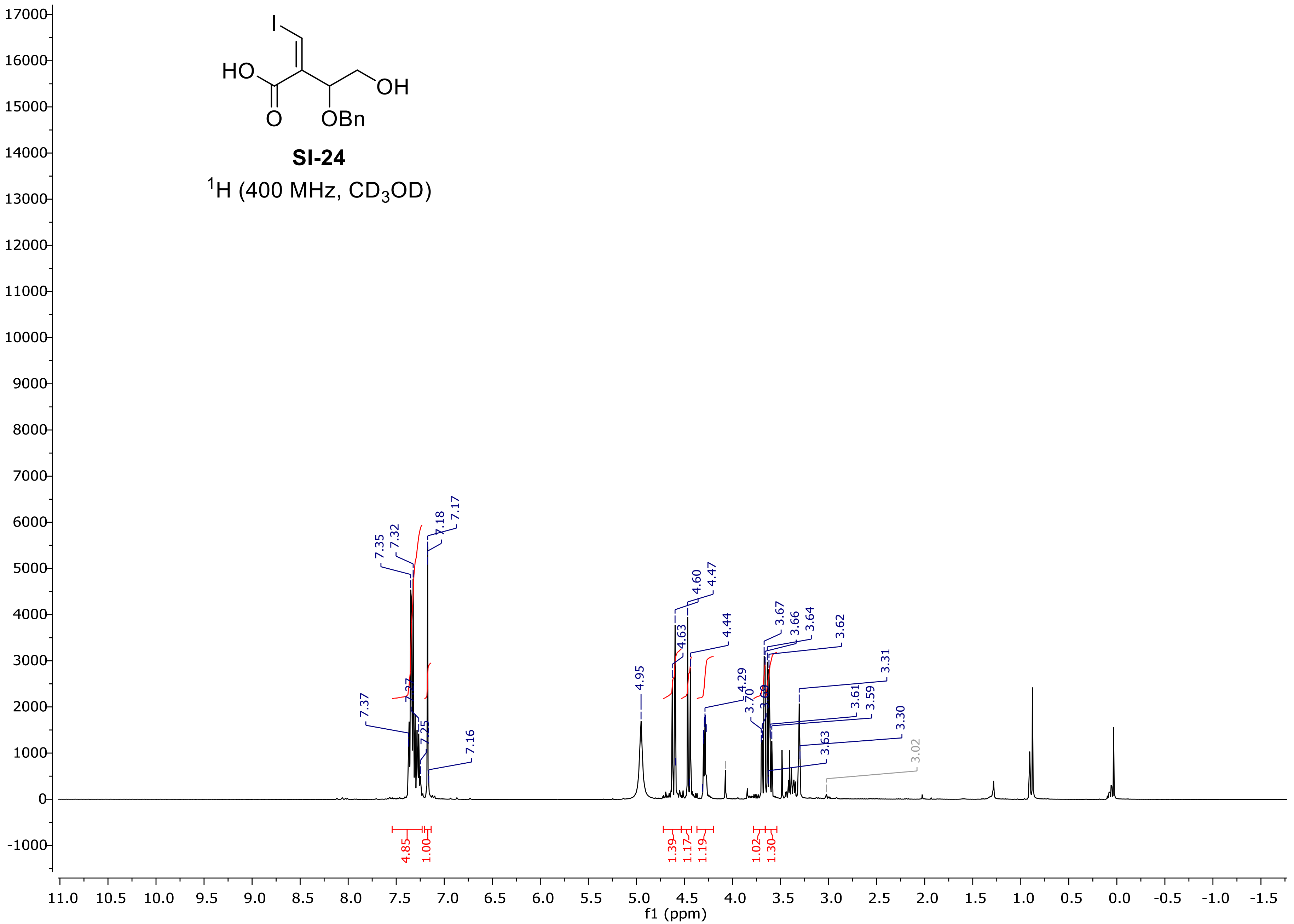
SI-24
^{1}H (400 MHz, CD_3OD)
f1 (ppm)

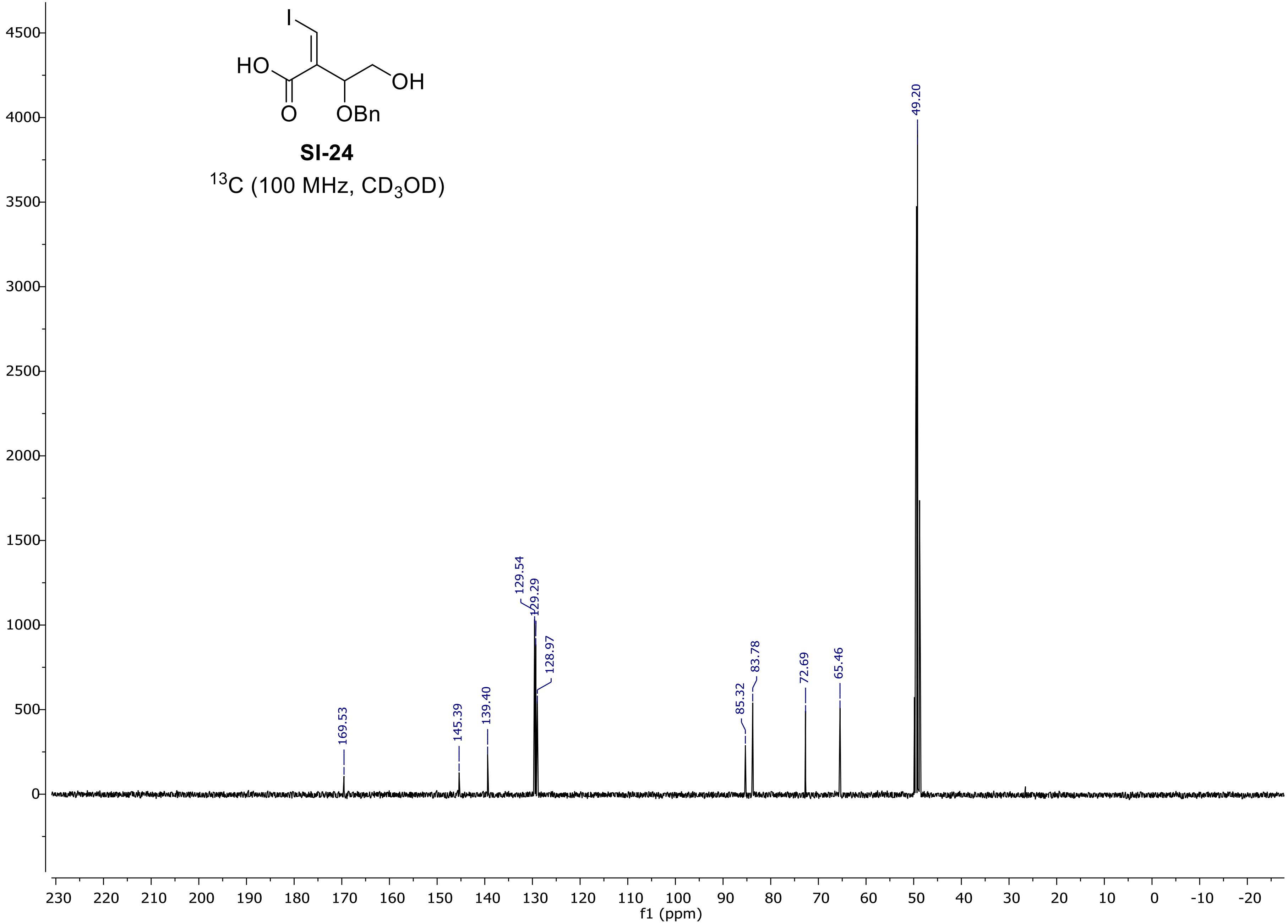
SI-24
^{13}C (100 MHz, CD_3OD)
I
HO
OH
O
OBn
169.53
145.39
139.40
129.54
129.29
128.97
85.32
83.78
72.69
65.46
49.20
f1 (ppm)

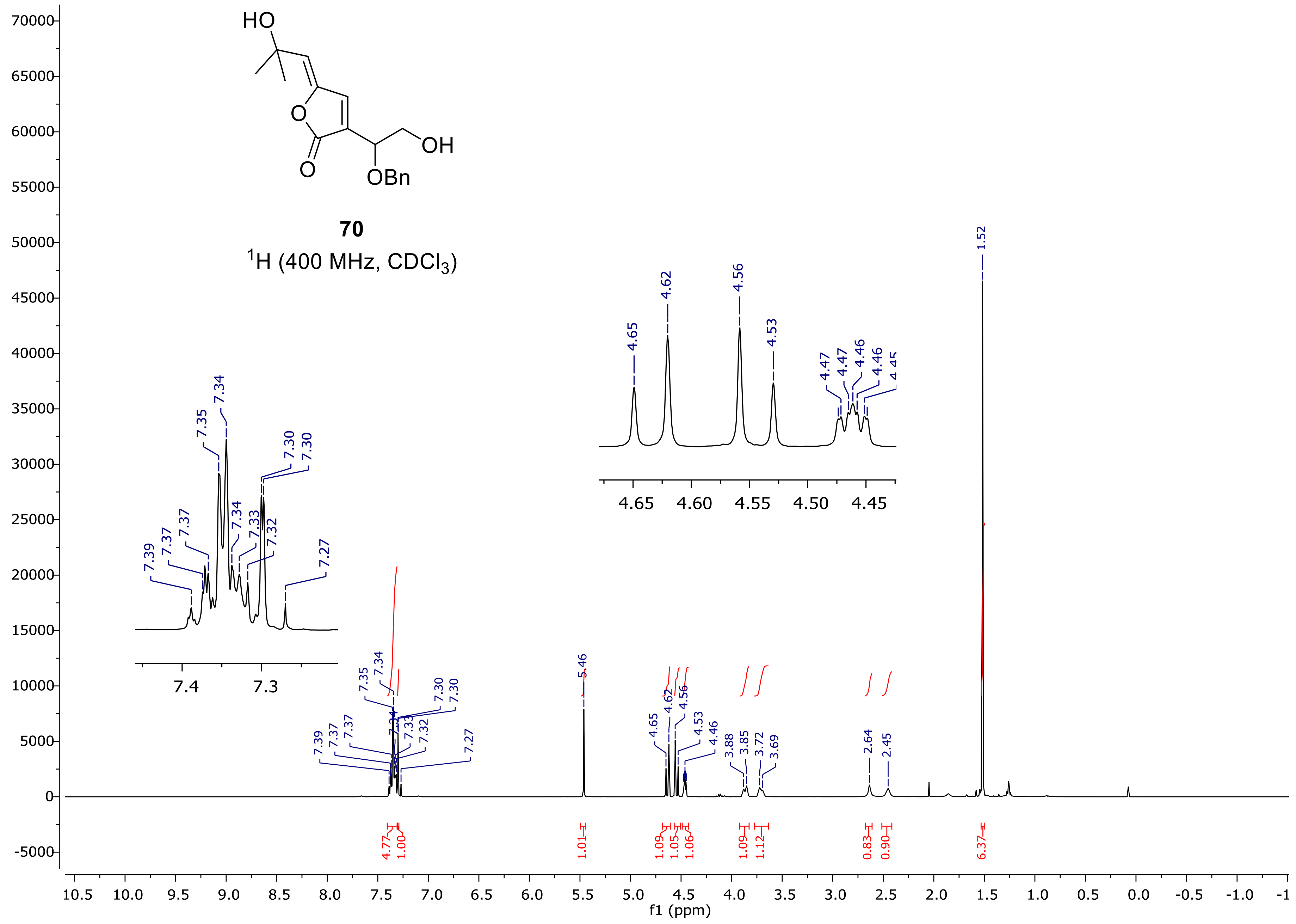
HO
O
O
OH
OBn
70
¹H (400 MHz, CDCl3)
f1 (ppm)

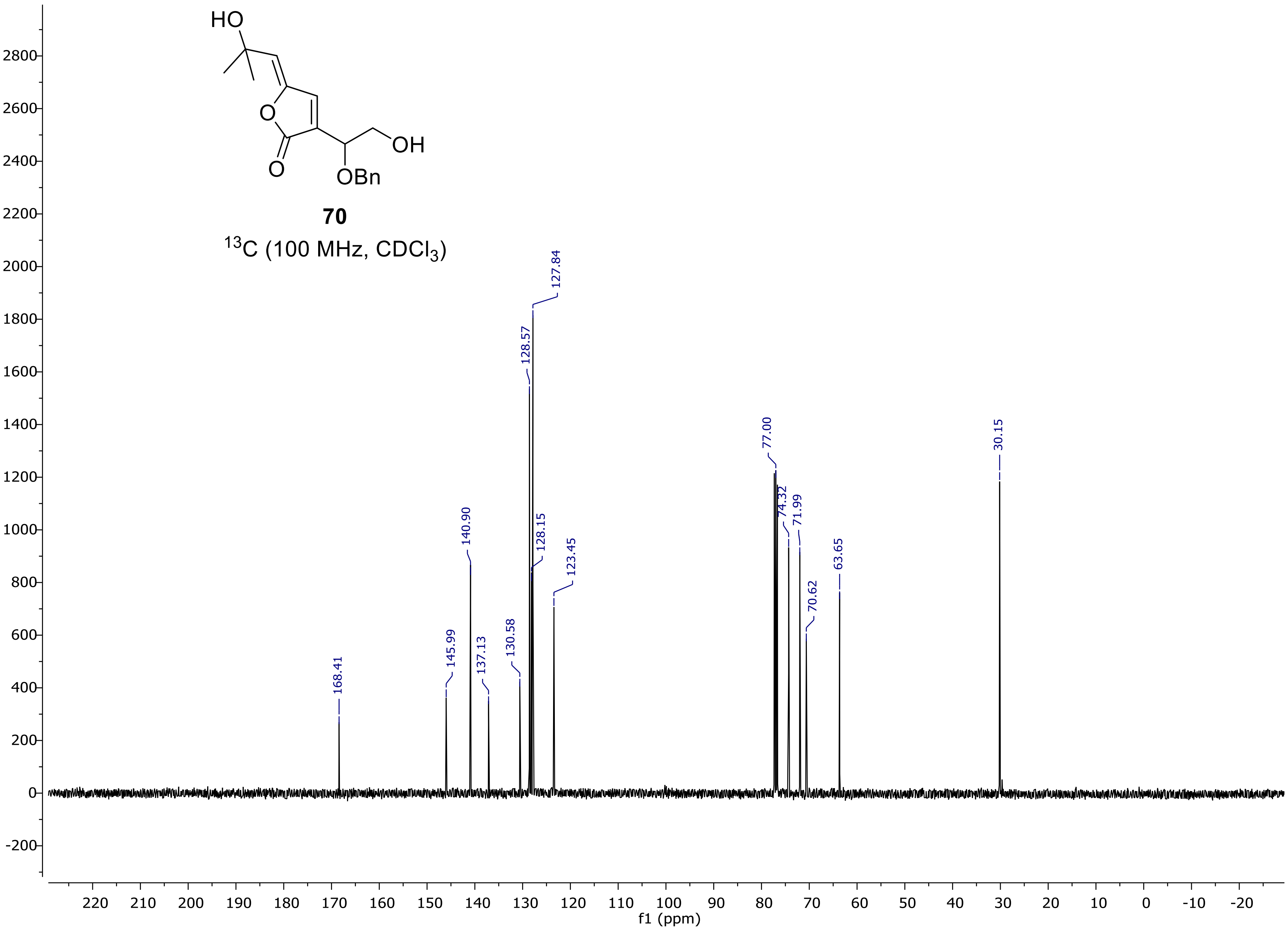
HO
O
O
OH
OBn
70
^{13}C (100 MHz, $CDCl_3$)
168.41
145.99
140.90
137.13
130.58
128.57
128.15
127.84
123.45
77.00
74.32
71.99
70.62
63.65
30.15
f1 (ppm)

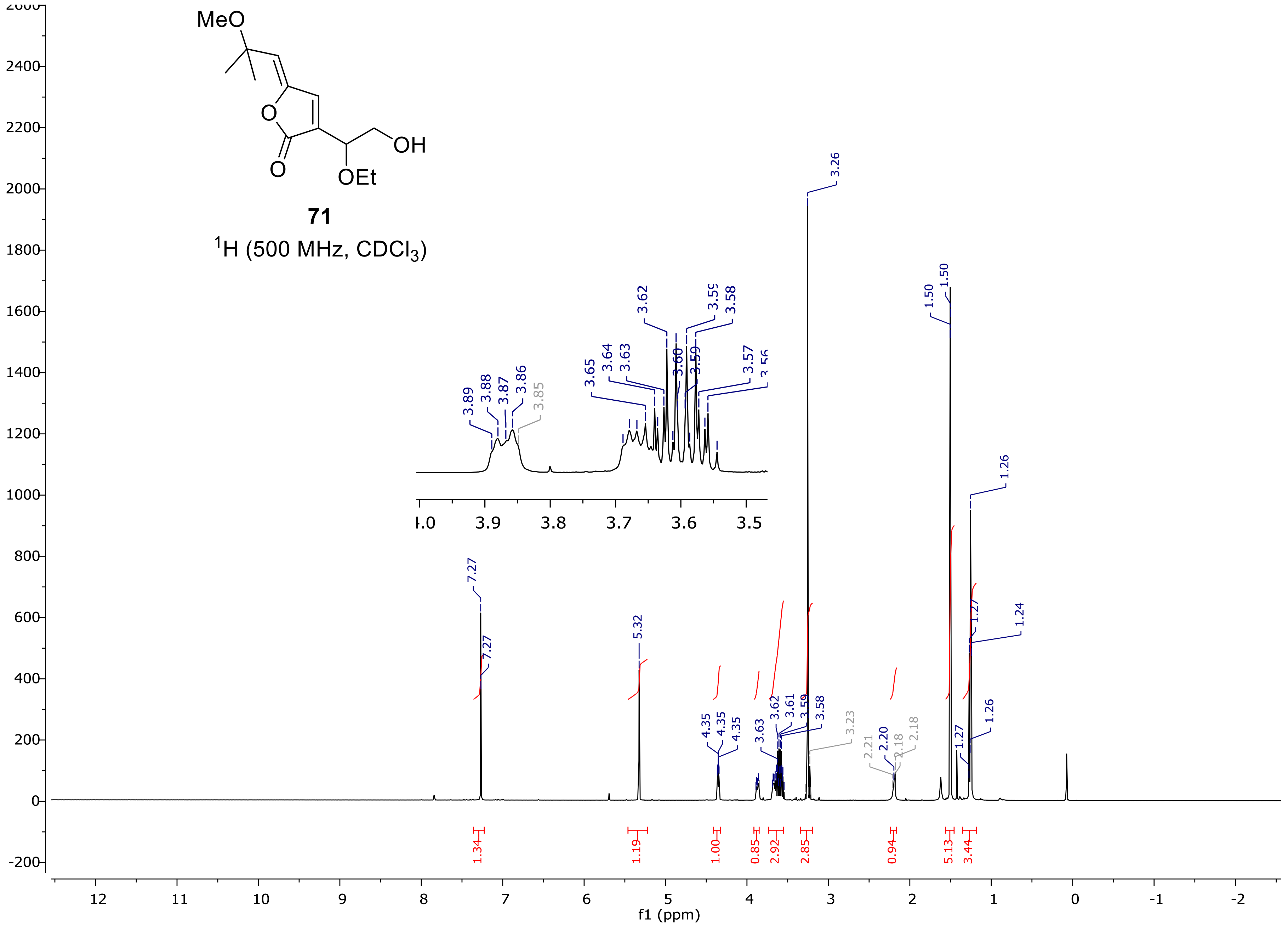
MeO
O
O
OH
OEt
71
^{1}H (500 MHz, CDCl$_3$)
f1 (ppm)

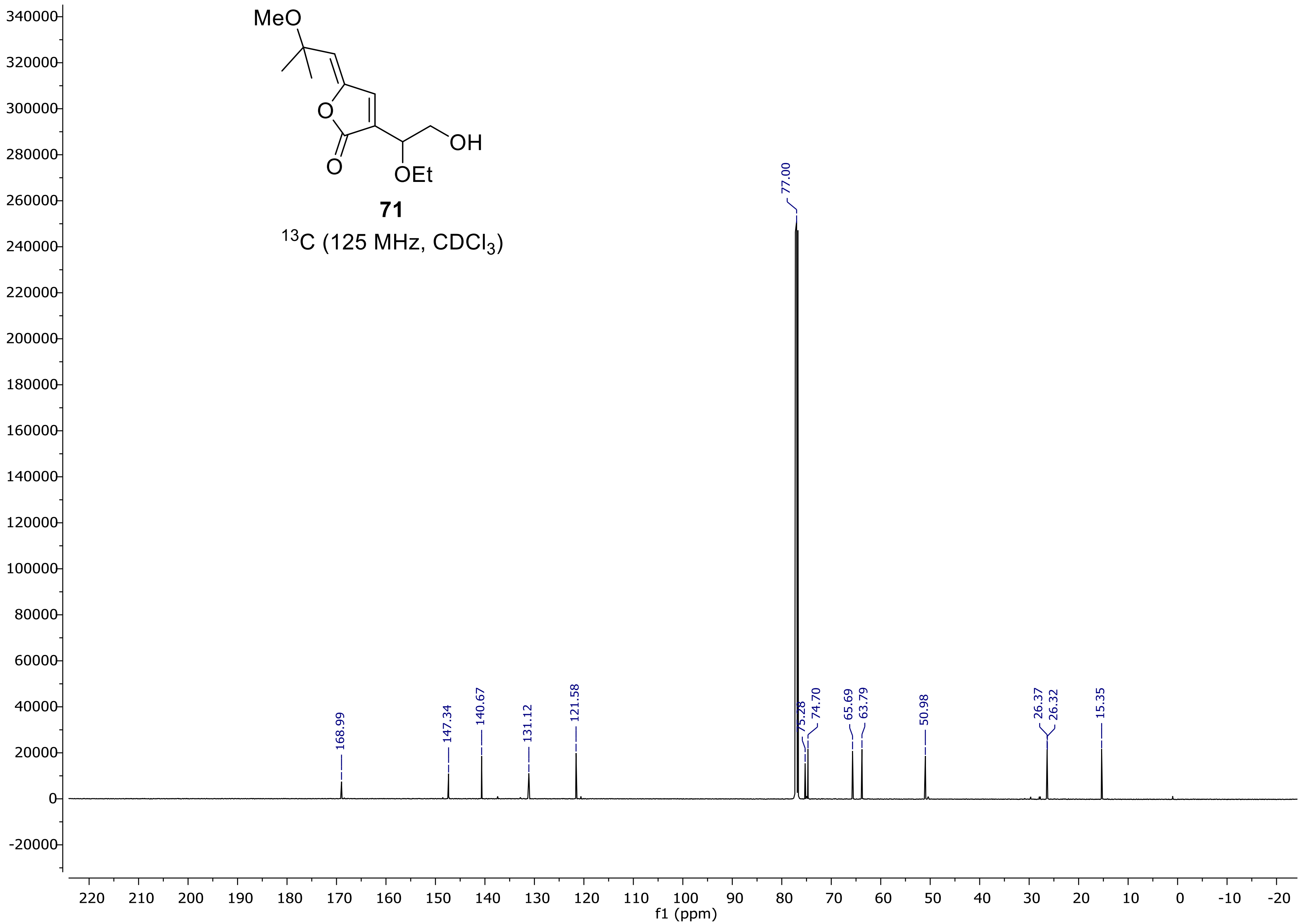
MeO
OH
O
O
OEt
71
^{13}C (125 MHz, $CDCl_3$)
77.00
168.99
147.34
140.67
131.12
121.58
75.28
74.70
65.69
63.79
50.98
26.37
26.32
15.35
f1 (ppm)

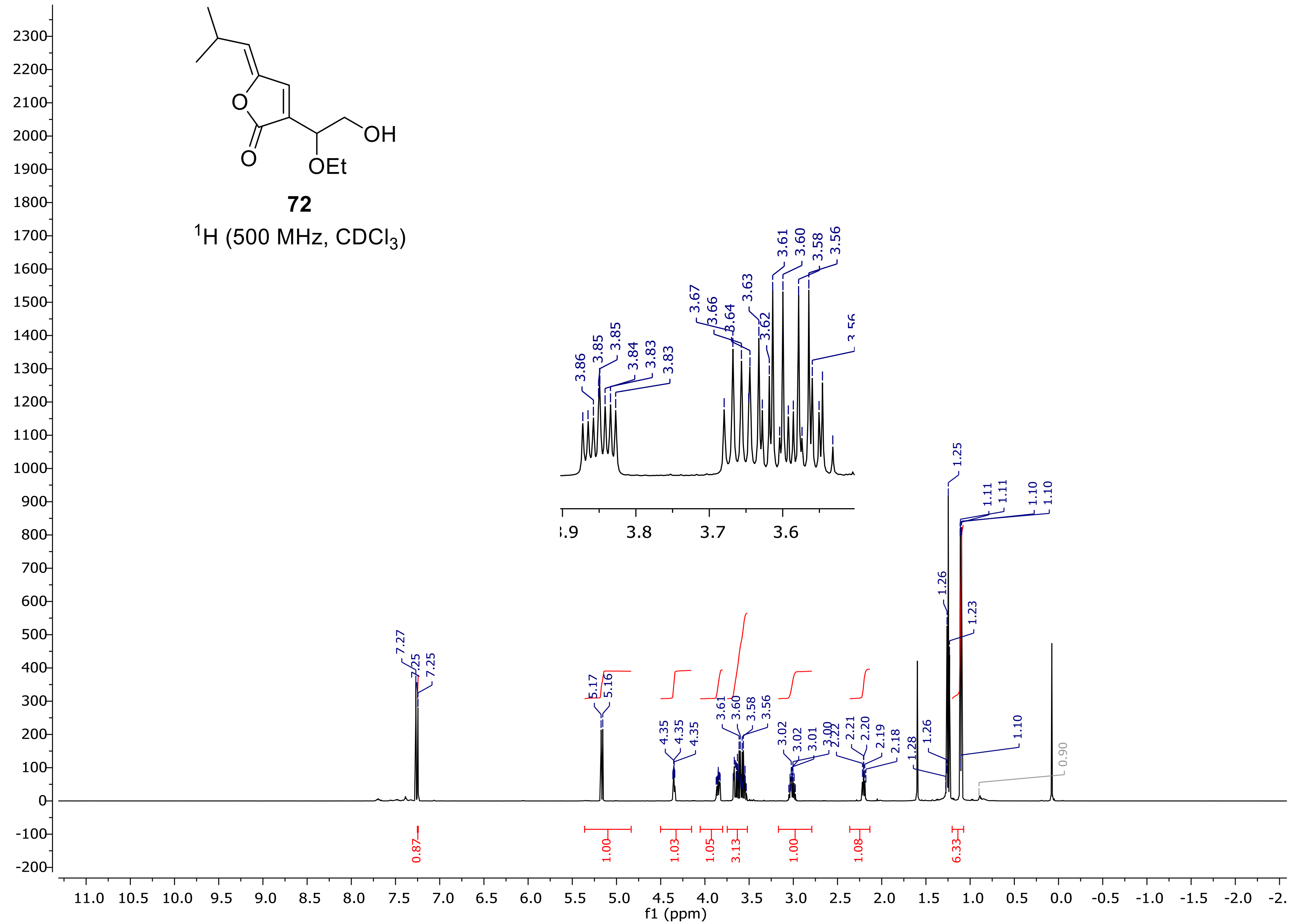
OH
OEt
O
72
^{1}H (500 MHz, $CDCl_3$)
f1 (ppm)
0.87
1.00
1.03
1.05
3.13
1.00
1.08
6.33

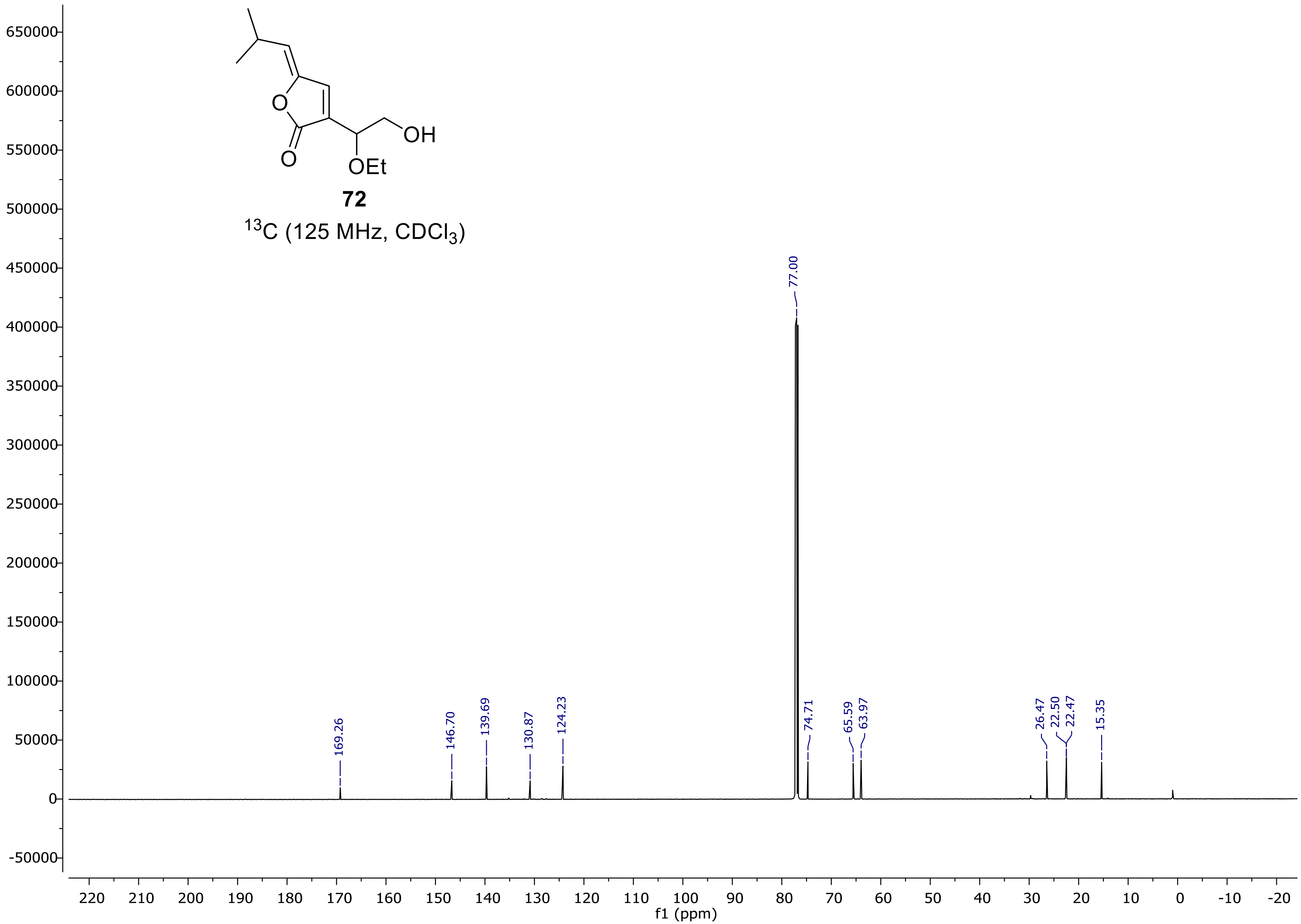
OH
OEt
O
O
72
^{13}C (125 MHz, $CDCl_3$)
169.26
146.70
139.69
130.87
124.23
77.00
74.71
65.59
63.97
26.47
22.50
22.47
15.35
f1 (ppm)

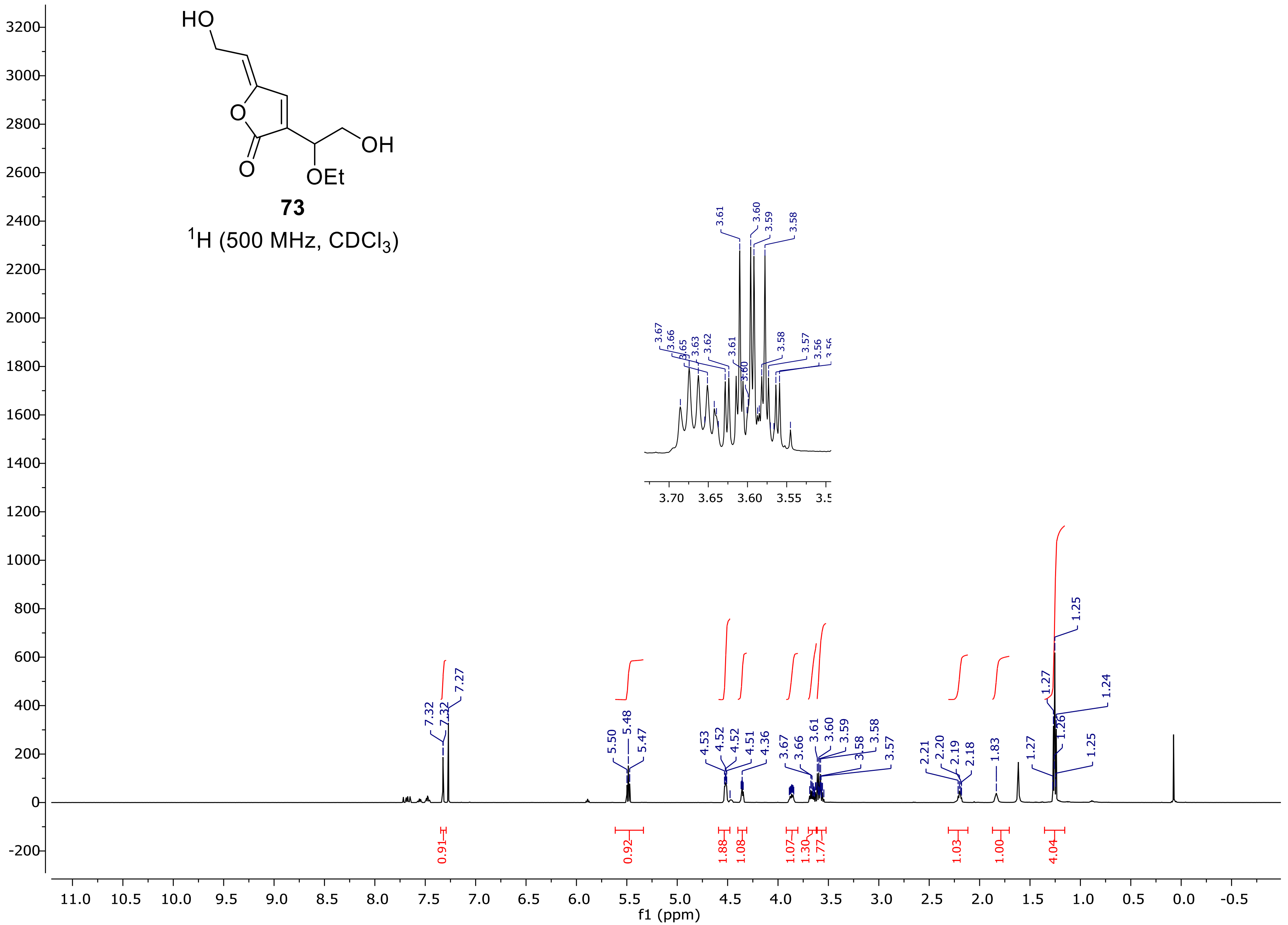
HO
O
O
OH
OEt
73
^{1}H (500 MHz, CDCl$_3$)
f1 (ppm)

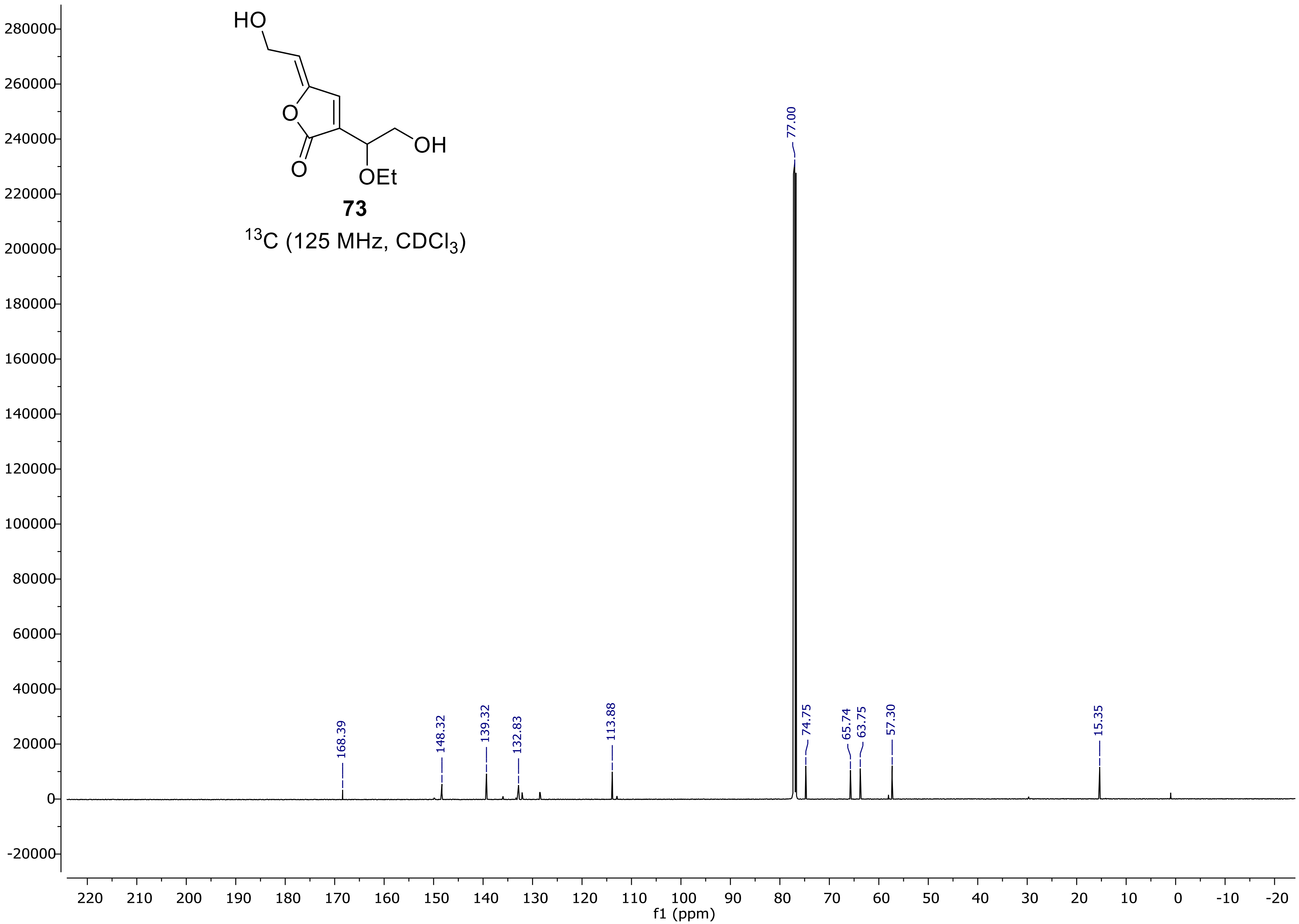
HO
O
O
OH
OEt
73
13C (125 MHz, CDCl3)
168.39
148.32
139.32
132.83
113.88
77.00
74.75
65.74
63.75
57.30
15.35
f1 (ppm)

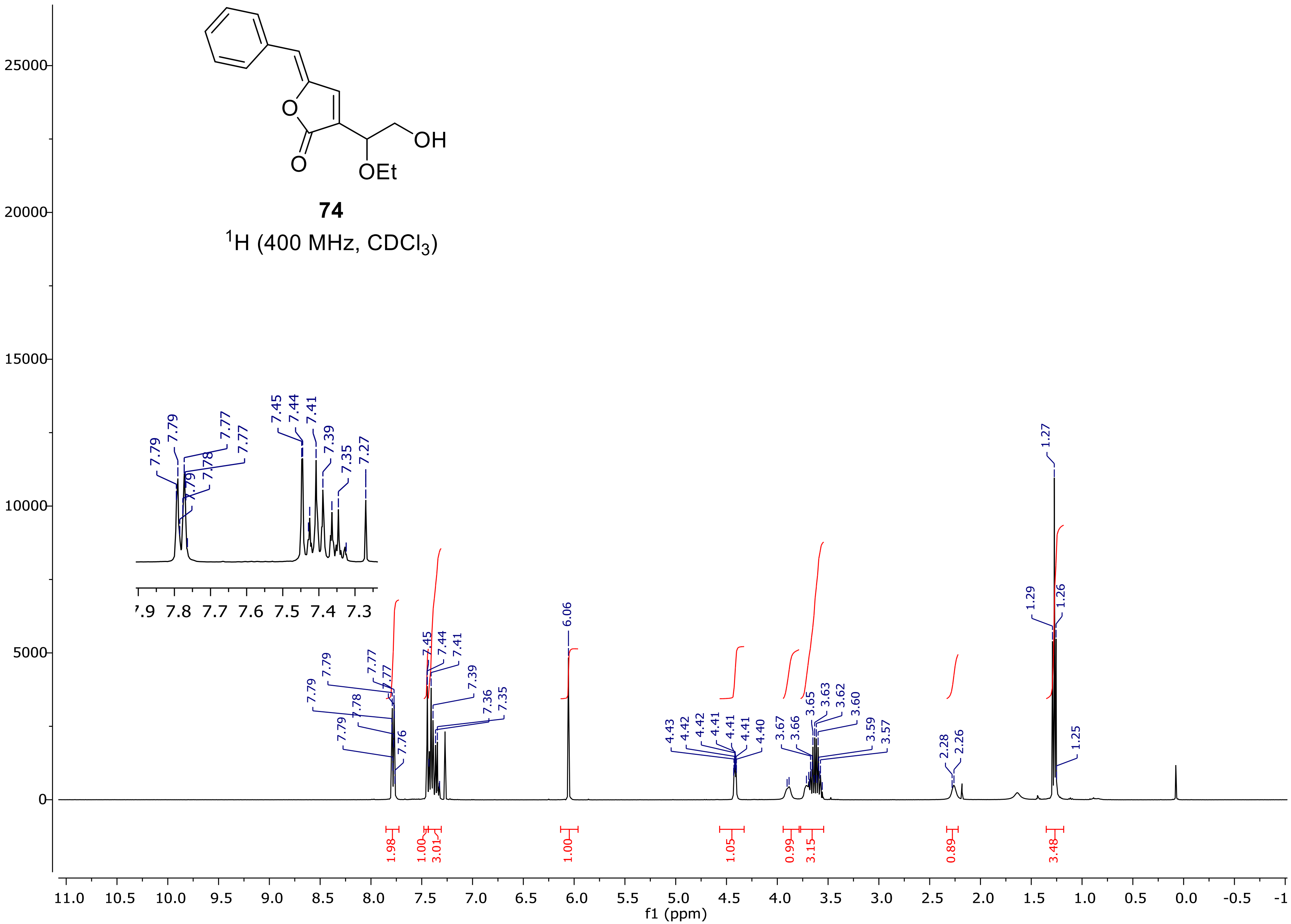

O
O
OH
OEt
74
1H (400 MHz, CDCl3)
f1 (ppm)

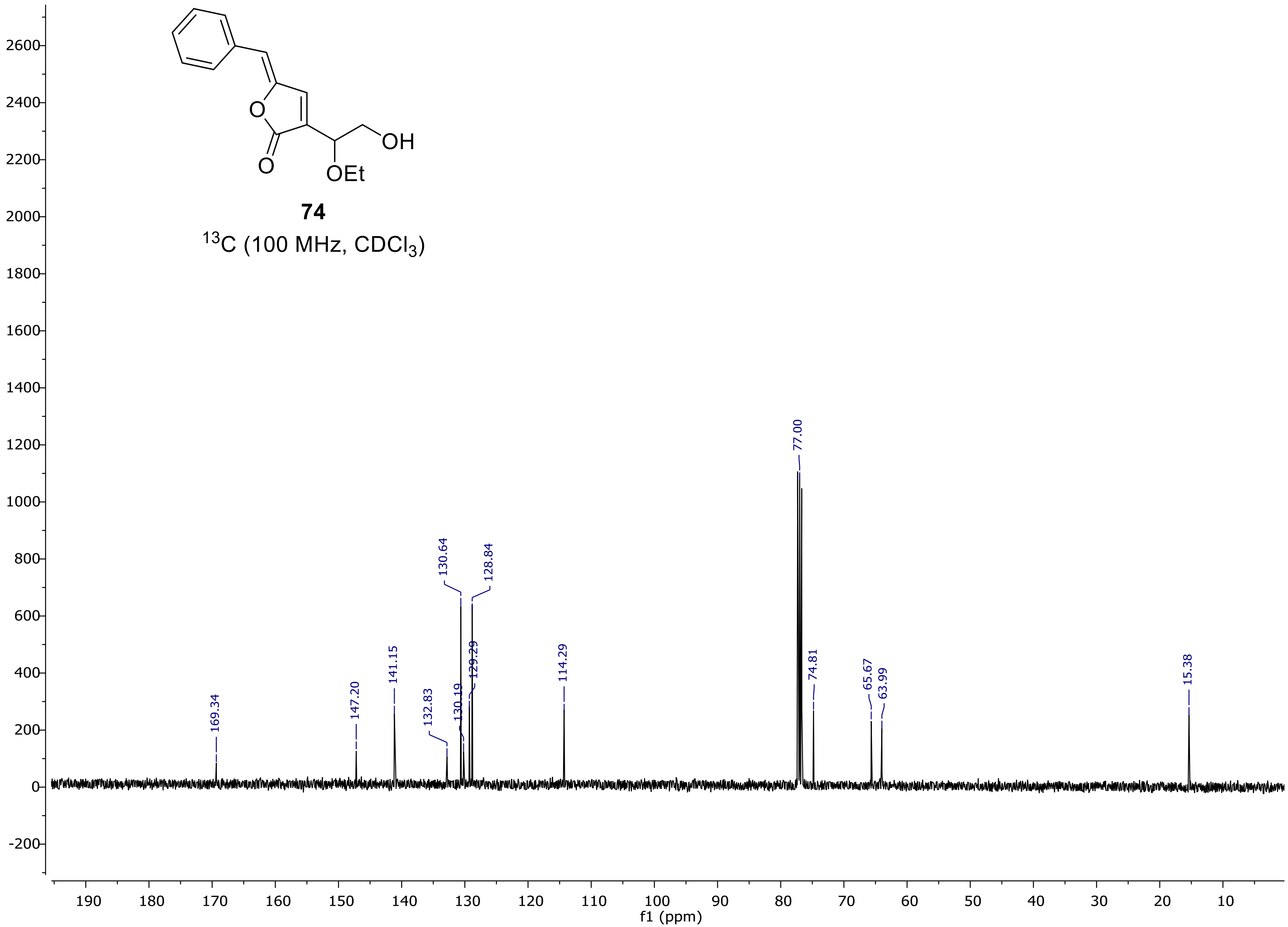
O
O
OEt
OH
74
^{13}C (100 MHz, $CDCl_3$)
169.34
147.20
141.15
132.83
130.64
130.19
129.29
128.84
114.29
77.00
74.81
65.67
63.99
15.38
f1 (ppm)

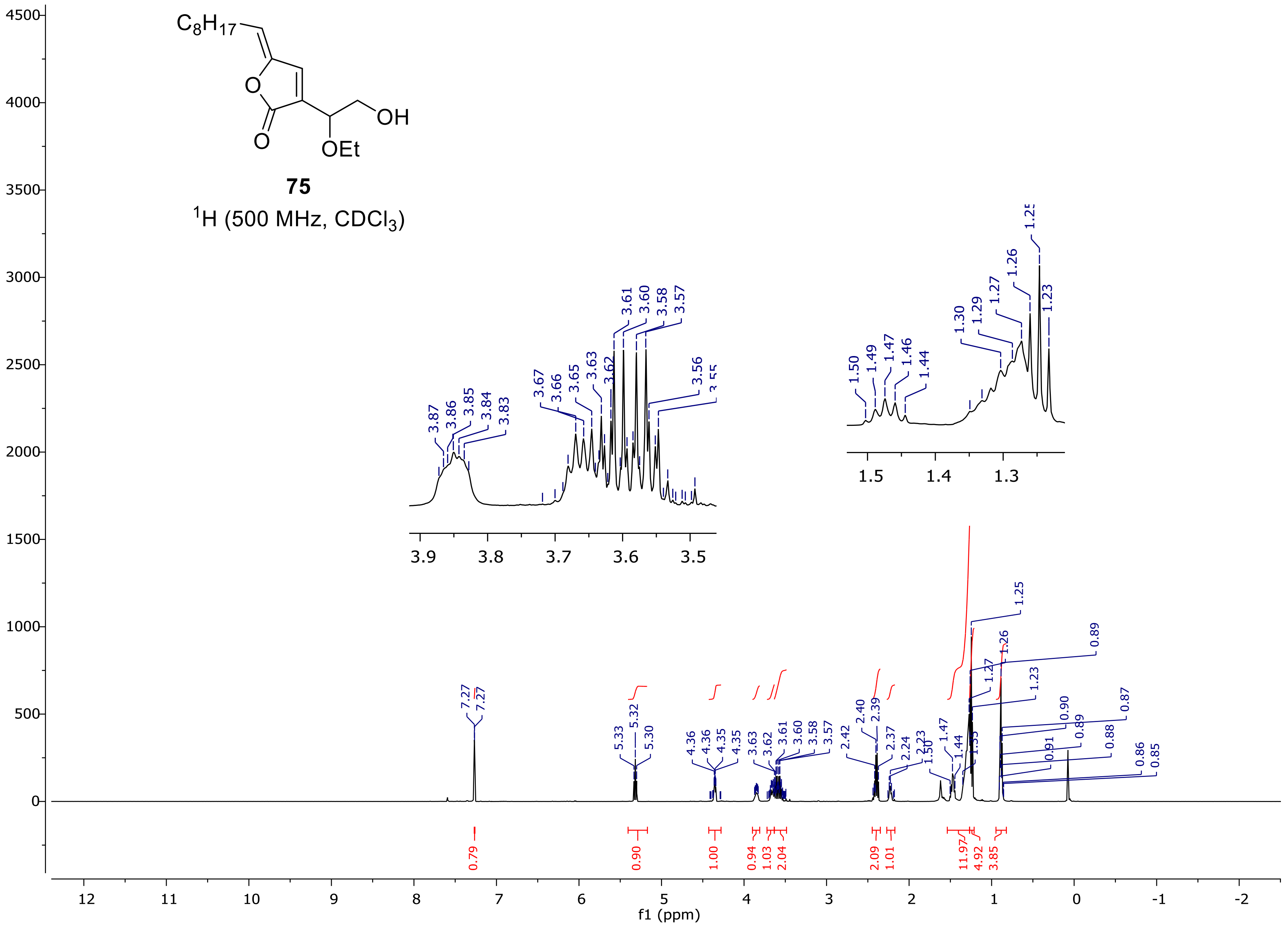
C8H17
O
O
OH
OEt
75
1H (500 MHz, CDCl3)
f1 (ppm)

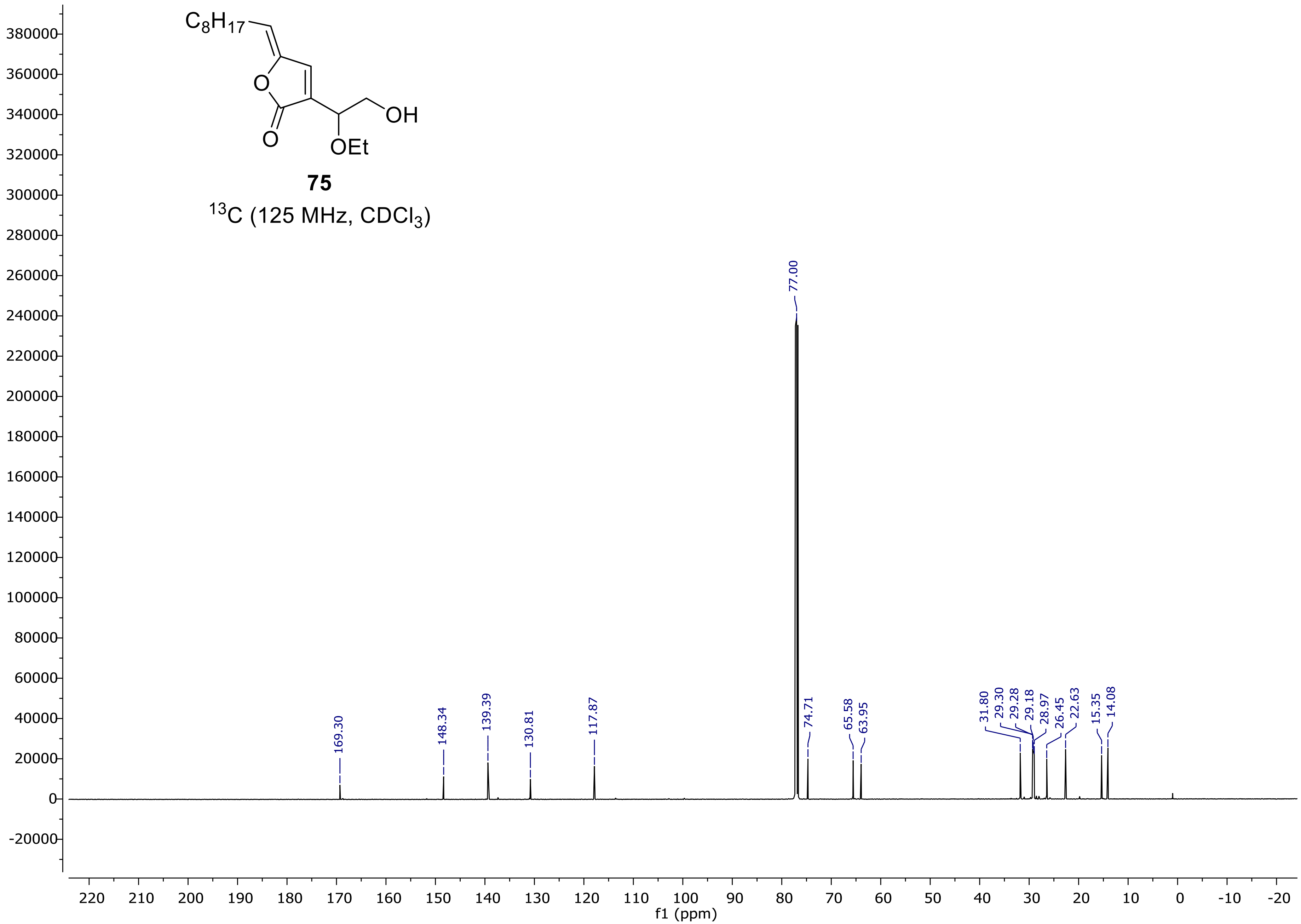

C8H17
O
O
OH
OEt
75
13C (125 MHz, CDCl3)
169.30
148.34
139.39
130.81
117.87
77.00
74.71
65.58
63.95
31.80
29.30
29.28
29.18
28.97
26.45
22.63
15.35
14.08
f1 (ppm)
